# Exploring perceptions of low risk behaviour and drivers to test for HIV among South African youth

**DOI:** 10.1371/journal.pone.0245542

**Published:** 2021-01-22

**Authors:** Tshifhiwa Muravha, Christopher J. Hoffmann, Claire Botha, Wellington Maruma, Salome Charalambous, Candice M. Chetty-Makkan

**Affiliations:** 1 The Aurum Institute, Johannesburg, South Africa; 2 John Hopkins University, Baltimore, Maryland, United States of America; 3 Division of Public Health Surveillance and Response, National Institute for Communicable Diseases, Johannesburg, South Africa; 4 School of Public Health, Faculty of Health Sciences, University of the Witwatersrand, Johannesburg, South Africa; 5 Health Economics and Epidemiology Research Office, Johannesburg, South Africa; 1. IRCCS Neuromed 2. Doctors with Africa CUAMM, ITALY

## Abstract

Human Immunodeficiency Virus (HIV) prevalence among South African youth is high, yet HIV testing remains suboptimal. We explored how perceptions of HIV risk and behaviours informed decisions to test for HIV. This study was conducted from April 2018 to March 2019 in Ekurhuleni district, Gauteng Province with males and females aged between 15–24 years. Twenty-five youth with unknown HIV status participated in in-depth interviews (IDIs); while four focus group discussions (FGDs) were conducted with those that previously tested for HIV. Probes used in the guides included types of incentives that youth would value when testing for HIV or receiving treatment; barriers and motivators to HIV testing; enablers and challenges to using cellphone technology and preferences on type of social media that could be used to create awareness about HIV testing services. IDIs and FGDs were audio-recorded, transcribed, and translated. QSR NVIVO 10 was used for the analysis. The majority of the youth perceived that their risk of HIV infection was low due to factors such as being young, lacking physical signs of HIV, being sexually inactive and parents not being HIV positive. However, youth identified high risk behaviours such as unprotected sex, multiple sexual partners, excessive drinking of alcohol, being victims of sexual abuse, road accidents and violent behaviour as increasing their vulnerability to HIV. Most youth highlighted cues to action that would motivate them to test for HIV such as support of parents, receiving incentives, improved confidentiality during HIV testing and receiving information about HIV via social media (Facebook, Twitter and Whatsapp). Despite perceptions of low risk to HIV, youth remain vulnerable to HIV. Disseminating HIV information via digital platforms; giving youth options to choose between testing locations that they consider to be private; providing incentives and equipping parents/guardians to encourage youth to test could optimise HIV testing.

## Introduction

Human Immunodeficiency Virus (HIV) infection among the youth remains a global health burden. Of 1.7 million new HIV infections, youth aged between 15–24 years accounted for 32% of global infections [1] and youth from Eastern and Southern Africa regions have the highest number of new HIV infections [2]. It is concerning that up to 6000 adolescent girls and young women (AGYW) are infected weekly with HIV globally [1]. In Sub-Saharan Africa (SSA) and South Africa (SA), AGYW aged between 15–24 years contributed up to 282 000 and 66 200 annual new HIV infections respectively [1, 3]. Of the 231 100 new HIV infections reported in SA in 2017, youth (15–24 years) contributed to the majority (38%) of new HIV infections in 2017 [3]. Youth often engage in sexual activities without using a condom [3, 4], have multiple sexual partners [5], and early sexual debut [3, 6] that place them at high risk for HIV. Despite their high risk behaviour, youth often perceive low vulnerability to HIV [6]. Partly for this reason, utilisation of HIV testing remains low among this population and a high proportion of youth are not aware of their HIV status [5, 7, 8].

Between 2012 to 2018, HIV testing utilisation among adolescents and young adults was below 20% [2]. The same trend is seen in the Southern Africa region, where a Zambian study reported that 42% of youth tested for HIV [9]. HIV testing among youth is insufficient for national infection control goals; a national survey from SA showed that only 58.8% had ever tested for HIV [3]. Another South African study reported that less than half (47%) of youth who participated in a study had ever tested for HIV [10]. Knowledge of HIV infection is essential to assessing appropriate medical care and initiation of antiretroviral therapy (ART) for those living with HIV.

There are several barriers that hinder youth from accessing HIV testing. Reported barriers include: fear of receiving positive HIV test results [11], disclosing HIV test results to family and peers [12], fear of facing rejection should they test HIV positive, having to travel long distances to HIV testing centres and inconvenient testing centre operating hours [13]. Other studies highlighted youth being concerned with stigma, discrimination and lack of confidentiality at HIV testing centres [12, 14]. Effectiveness of current interventions may have not met testing goals, due to limited understanding on youth preferences for HIV testing services.

Over the years multiple approaches have been used to increase HIV testing among youth (aged 15–24 years). In SA, campaigns aimed at broadening HIV awareness through youth focused HIV prevention programmes include LoveLife, a youth focused HIV prevention strategy, Soul City TV drama series, and advertisements on television [3]. Peer navigators (PNs) have also been used with some demonstrated success. For example, use of PN programme to promote HIV testing was utilised by at least 88.8% participants among street-connected children and youth in Kenya. PNs were youth (18–24 years old), who tested for HIV and were known in the community. They spent time building rapport with street-connected children and youth who either lived entirely or partly on the street [15]. HIV self-testing [16], particularly unsupervised and home-based testing [17] methods have also been found to be acceptable to youth. SA was also part of the DREAMS project which was initiated to provide multifaceted interventions such as HIV testing options aimed at optimizing HIV testing among AGYW [18, 19]. However, there is still a gap on understanding what choices youth prefer in relation to HIV testing. Here we used in depth interviews and focus group discussions to explore youth’s perceptions of HIV risk and behaviours that could motivate them to test for HIV.

## Methods

### Description of the study area

This study took place from April 2018 to March 2019 in Ekurhuleni, a peri-urban area of the Gauteng Province, South Africa. Recruitment took place in the community, at school or college events; door-to-door community recruitment and at public health clinics. Potential participants were individually invited to take part in the study. Interested participants gave contact information to the research staff that included names and phone numbers.

### Study design

This was a qualitative study where participants were invited to take part in an in-depth interview (IDI) or focus group discussion (FGD) to understand preferences for HIV testing. For IDIs, participants with unknown HIV status were eligible (had not undergone HIV testing), while those who tested for HIV previously were invited to be part of the FGDs. Locator information was recorded and used to telephonically contact participants to schedule appointments at a time that was convenient for them. In South Africa, research participants <18 years do not provide written informed consent. Participants aged 15–17 years old were eligible if they agreed to sign the assent form; their parent or legal guardian agreed to provide parental consent; showed proof of valid identification; agreed to be interviewed with digital recording of the session; and were able to communicate in one of the study languages. Participants aged 18–24 years were eligible if they provided written informed consent to participate; showed proof of valid identification; agreed to be interviewed with digital recording of the session, and were able to communicate in one of the study languages. Illiterate participants provided a thumbprint to acknowledge understanding in the presence of a witness. Participants were either interviewed or participated in an FGD once.

In order to assess saturation, a method for ensuring that the themes that emerged from the transcripts started to recur, so no new information was being identified. A preliminary analysis was conducted on 12 IDIs and one FGD. We noticed that the themes on types of incentives to encourage HIV testing started to recur thus saturation was reached. However, reasons for selecting a specific type of incentive needed to be explored further. Probes were revised, research assistants re-trained and detailed probing implemented for the remaining IDIs and FGDs. Based on the number of interviews and FGDs conducted and revision of probes to fully describe themes, saturation was reached by observing recurrence in themes from all transcripts. The authors (CMC and TM) qualitatively assessed the transcripts for saturation and when no new themes emerged from the transcripts we deemed that saturation was reached [20].

### Data collection

We invited 28 participants for the IDI and excluded three (10.7%) due to them reporting at enrolment that they previously tested for HIV. Twenty-five males and females aged between 15–24 years that had never tested for HIV were interviewed. Each IDI lasted approximately 45 minutes to an hour. A summary of demographics (Table 1) of the IDI participants that took part is shown. We conducted four FGDs with a total of 18 males and females with history of HIV testing, where participants were divided according to two age categories (15–17 years and 18–24 years). Each FGD took approximately 1–2 hours long per session. The IDI (Table 2) and FGD (Table 3) guides explored the types of incentives that youth would value when testing for HIV or receiving treatment; barriers and motivators to HIV testing; enablers and challenges to using cell phone technology and preferences on type of social media to be used to create awareness about HIV testing services. Interviews were conducted by two males and one female; all interviewers had prior experience on qualitative data collection and were trained for purposes of the study. The interviewers were young adults who were able to identify with the participants and speak local languages used during interviews. IDIs and FGDs were conducted in preferred languages of participants, which were either English, isiZulu, Setswana, or Sepedi.

**Table 1:** Demographic characteristics of participants in in-depth interviews.

		Total		Males		Females	
		n = 25	%	n = 11	%	n = 14	%
**Age category**	15–17 years	11	44.0	5	45.5	6	42.9
	18–24 years	14	56.0	6	54.5	8	57.1
**Education**	High school	23	92.0	9	81.8	14	100.0
	Tertiary	2	8.0	2	18.2	0	0.0
**Currently Employed**	Yes	1	4.0	1	9.1	0	0.0
	No	24	96.0	10	90.9	14	100.0
**In a relationship**	Yes	12	48.0	6	54.5	6	42.9
	No	13	52.0	5	45.5	8	57.1
**Currently sexually active**	Yes	13	52.0	6	54.5	7	50.0
	No	12	48.0	5	45.5	7	50.0
**Access to cellphone**	Yes	16	64.0	6	54.5	10	71.4
	No	9	36.0	5	45.5	4	28.6

**Table 2:** In-depth interview guide.

**Part 1: Structured questionnaire**
1. What is your gender?
2. How old are you?
3. What is your educational status?
4. Are you employed?
5. What is the name of the public health clinic that you attend?
6. How far is the clinic from your home?
7. Are you currently in a relationship? If yes, how long have you been in a relationship?
8. Are you sexually active?
9. Do you know your HIV status?
10. Do you have a cellphone?
**Part 2: In-depth Interview**
1. Please describe to me your thoughts on HIV?
a) How can you become infected with HIV?
b) Tell me about what places a person at risk for HIV?
c) Can you tell me about any situation when you felt that you may have been at risk for HIV?
2. Can you tell me about the HIV testing services that take place in your community?
a) Where are the HIV testing services for youth usually delivered?
b) Can you tell me about your experience accessing these services?
c) In your opinion what is positive about the current HIV testing services that are available to youth?
d) In your opinion what are negative aspects of the current HIV testing services that are available to youth?
3. How do you think incentives could be used to encourage youth to test for HIV and access treatment?
a) What is your understanding of incentives?
b) Please describe the types of incentives that youth value which could encourage them to test for HIV or access treatment?
c) How often do you think these incentives for HIV testing services should be provided?
d) What could be the challenges of providing these incentives for HIV testing services?
e) What would be the benefits of providing these incentives for HIV testing services?
4. Please describe to me your thoughts about being contacted via telephone or social media for HIV testing services?
a) Can you please describe some examples of how you have been informed about HIV testing services?
b) How would you feel about being informed and registering for HIV testing services using your cellphone?
c) How could cellphones be used to inform youth of HIV testing services?
d) Please describe any challenges that youth might experience if they are contacted on their cellphone for HIV testing services.
e) Please describe the benefits of contacting youth on their cellphones for HIV testing services
f) In your opinion what types of other social media should be used to contact youth for HIV testing?
g) What are the challenges for using social media to contact youth for HIV testing services?
h) What would be the benefits of using social media to contact youth for HIV testing services?
i) How do you think your parents/legal guardians would feel about you receiving information on HIV testing on your cellphone or social media?
5. Can you tell me about any other suggestions that you may have which would encourage youth to test for HIV?

**Table 3:** Focus group discussion guide.

1. Please describe your positive and negative experiences of being tested for HIV at an adolescent friendly clinic
2. Tell us about suggestions that you may have on how to improve HIV testing services at adolescent friendly clinics
3. How do you think we should inform youth about HIV testing and treatment?
4. Can you tell us what measures we could put in place to encourage youth to test for HIV?
5. What do you think about providing incentives to young people to access HIV testing services?
6. Please describe types of incentives that young people would value when testing for HIV or receiving treatment
7. Please tell us about the benefits and barriers to use of incentives for HIV testing services
8. How would you feel about being informed and registering for HIV testing services using your cellphone?
9. How could cellphones be used to inform youth of HIV testing services?
10. Describe the positive and negative of using cellphone technology when contacting adolescents or young adults for HIV testing and treatment
11. In your opinion, please tell us what type of social media should be used to inform adolescents or young adults about HIV testing services
12. In your opinion, how would your parent/legal guardian feel about you receiving information on HIV testing services on your cellphone or social media?
13. Can you tell us about other suggestions that you may have on how we could encourage youth to speak honestly about their sexual behaviours?
14. Are there any final thoughts you have about youth, accessing HIV testing services or the use of incentives?

Only the interviewer and the participant were present during the in-depth interview session. For FGDs, an interviewer, note taker and participants were present during each session. Research staff were trained to be objective during all contact with participants. All participants were reimbursed approximately $15 (ZAR150.00) to compensate them for their time and travel.

### Data analysis

All transcripts were anonymised for confidentiality (S1 File). IDIs and FGDs were transcribed verbatim, and translated into English where applicable. Most transcriptions and translations were outsourced to master’s level students based in South Africa who transcribed the digital recordings to the local language and then back-translated into English. Authors (CMC, TM and WM) quality checked the transcriptions. All transcripts were coded and analysed in English. Digital recordings were reviewed during the analysis. QSR NVIVO 10 was used for the analysis. Thematic analysis uses deductive and inductive approaches were used to develop the themes [20]. An iterative approach was used to develop the codebook and index the transcripts from which categories, themes and patterns developed from the data as a standard component of qualitative analysis in which codes are developed and then applied to transcripts for indexing related text for use in thematic analysis. Authors with doctorate level (CMC) and master’s levels (WM, TM) qualifications developed the coding scheme using the English transcripts. For reliability, the original transcript and codes were sent to independent reviewers. The independent reviewers (CJH, CB, SC) were part of the investigator team with medical and doctorate level qualifications. The codebook was discussed at a round table meeting where new codes were suggested [12] Initially our broad coding included categories of *HIV testing Services* and *Incentives*. During our first review, *cell phones* and *HIV information sharing* codes were later added because more information developed from transcripts. The authors agreed to particular codes such as *HIV testing services*, *incentives*, *cell phones*, *and HIV information sharing*. Codes that lacked intercoder reliability were dropped from the analysis [21]. For example, we moved sub-categories of *parental perceived opposition* and *parental perceived support* from the *HIV testing* code to another main code labelled as *parental roles* due to strong patterns that developed related specific to parental support. For the parent code of *incentive*s, we removed *games* and *Youtube* as sub-categories, as trends related to these did not develop during review of the transcripts. The codebook was revised by authors (CMC and TM) and the themes finalised which are displayed as direct quotes [20, 22]. Participants did not provide feedback on the findings. The COREQ checklist (S2 File) which guided the data collection and data analysis process is attached.

### Ethics statement

Approval was obtained from the University of the Witwatersrand Human Research Ethics Committee (180203) and from the Gauteng Province Ekurhuleni health district (GP_201804_010). Ethics approval was granted to use direct quotes for reporting.

## Results

The findings of this qualitative study are built on the three main themes and their corresponding sub-themes. Youth perceived that their risk to HIV infection was low. As a result, youth still engaged in behaviours that exposed them to HIV risk. Youth were able to describe some cues to action that they perceived could promote HIV testing among their age group. Table 4 demonstrates a quantitative breakdown of participants’ response rate:

**Table 4:** Response rate of participants based on the main themes and sub-themes.

Main Theme	Sub-themes	Number of participants	Number of times a statement was coded
**Behaviours related to susceptibility and exposure to HIV risk**		29	256
	Accident	3	4
	Emotional reactions	21	40
	Fear of HIV testing procedure	19	52
	High risk sexual activity	25	79
	Parent-child infection	6	9
	Physical reactions	5	8
	Physical, sexual or emotional violence	6	9
	Sexual reactions	6	11
**Cues to action**		20	127
	Confidentiality	7	12
	Education and awareness using social media	20	190
	HIV testing procedure	10	24
	Incentives	22	543
	Parental role	8	18
Perception of low risk to HIV infection		22	76
	Low risk	17	45
	Misconceptions	8	20
	Myths around HIV testing- infection	5	10

### Main theme 1: Perception of low risk to HIV infection

Most youth felt that being young, absence of physical signs of HIV, being sexually inactive and having parents not being HIV positive placed them at low risk of HIV infection. Perception of low risk to HIV infection was similar across the attributes of age and gender. It was mostly those with unknown HIV status who felt that they were at low risk to HIV.

“*…because I do not sleep with girls. I am still very young….*”

(*Male, 15 years, IDI*)

“*I thought that it was not my time cause like I was very, too young.*”

(*Female, 15years, IDI*)

“*… They ask what makes you test, what do you know at such a young age?*”

(*Female, 18years, IDI*)

For some youth, absence of physical signs of HIV convinced them that their risk to HIV infection was low.

“*Uhh {what makes me think that I didn’t get infected} is because I didn’t have the symptoms {of HIV}.*”

(*Male, 18years, IDI*)

“*I have never tested for HIV because I have never encountered danger like to go to test…Like changes to my body like maybe sores around my body…*”

(*Female, 18years, IDI*)

Others perceived they were at low risk to HIV infection as a result of being sexually inactive.

“*…The thing is I have never been intimate with a boy; yea I have never had sex.*”

(*Female, 15years, IDI*)

“*… if a person or youth does not… is not sexually active they don’t think it is necessary for them to go and test they just think cause I am not having sex they just sit and not go to test.*”

(*Female, 18years, FGD*)

Another reason for perception of low risk to HIV infection highlighted by the participants was the assumption that their own parents were HIV negative.

“*I don’t remember my mother going to the clinic to get treatment or my dad. I don’t remember so I doubt there is a way that I could be HIV positive.*”

(*Female, 15years, IDI*)

“*… Yes so; they would say that maybe because their mothers don’t have it that means they also don’t because others say, you get it from your mother.*”

(*Male, 15years, IDI*)

### Main theme 2: Behaviours related to susceptibility and HIV risk exposure

Although the perception of the youth’s own risk of HIV was low, youth still felt susceptible to being infected with HIV. There are different ways that youth exposed themselves to behaviours that made them vulnerable to HIV infection. These reported behaviours included high risk sexual behaviour and being victims of sexual abuse. Majority of the youth felt that unprotected sex, multiple partners, excessive drinking of alcohol and substance use were high risk behaviours that placed them at high risk of getting infected with HIV. There were gender differences reported for sexual violence where only females across ages spoke about their experiences of abuse.

Engaging in unprotected sex was one of the behaviours that placed youth at high risk of HIV infection.

“*Oh. Ah. I was having unsafe sex. So, the condom…like I was using the condom but it burst …*”

(*Male, 18years, IDI*)

“*… the first time that I tested was …2015 because I didn’t use a condom so I wanted to know my status.*”

(*Female, 18years, FGD*)

Youth felt being involved with multiple partners, excessive drinking of alcohol and possibility of being sexually abused placed them at high risk of getting infected with HIV.

“*…Ok like if they give us alcohol by the time I get to the corner I will be drunk and I meet people that want to go to {XXX} (Name of place), then I will go every other place and I will wake up undressed the next morning. Not knowing how many people I slept with, am I HIV positive, I am pregnant I don’t know the father of the baby I don’t even know how I got to where I am cause of alcohol.*”

(*Female, 15years, FGD*)

“*Because the people there would have drank alcohol and they would be drunk. And when they want to have sex…they would not be thinking straight because they are not in their right mindset.*”

(*Male, 15years, IDI*)

“*…when we are drunk we lose 60% of our right brain we just think random things…*”

(*Female, 18years, FGD*)

Other youth cited drug users as being at high risk of HIV infection due to excessive use of certain drugs and sharing of needles.

“*… most people use drugs like nyaope (an opioid) and now it’s taken in different ways, some inject it. They inject it through they don’t know where this injection has been or who has used, they use it without information because they just want to use it. Even those people will need help.*”

(*Male, 18years, FGD*)

A parent that frequently tested for HIV was suspected of having HIV, which some youth saw as a gauge for their own risk or HIV status.

“*Yoh! The way I saw my mom always testing. So, I also thought that I have HIV. Always, she was checking and telling us how it went, when coming back.*”

(*Female,15years, IDI*)

Other youth reported being victims of sexual abuse placed them at high risk of being infected with HIV.

“*… I was drunk, so I was with my friend we were at and we went to party. So there came these guys, they had a car so we went with them to the party then they took us to their place. …And then that day I don’t know what happened, I just found myself sleeping on a bed, but when I touched myself, I felt like I was not good and then, maybe someone raped me or something.*”

(*Female, 18years, IDI*)

“*… I was in grade 12 2015, it was Sunday and I got raped by a person, that person that I trusted, he was like a brother and an uncle to me, but that time he had changed and I did not know what was happening with him. The worst part is that my parents didn’t know where I had gone to so I was afraid to tell them what had happened…*”

(*Female, 18years, IDI*)

Others felt that suffering physical abuse such as stabbing or being involved in physical fight made youth susceptible to high risk of HIV infection.

“*…maybe when someone got injured, having been stabbed with a bottle and you have a wound as well. If the blood gets into your wound, you can be infected as well.*”

(*Male, 15years, IDI*)

### Main theme 3: Cues to action for HIV testing

Some of the cues to action that would encourage youth to test for HIV were support of parents, receiving incentives and improved confidentiality during the HIV testing process. All youth across gender and age attributes responded positively to the possibility of incentives.

“*…So at least at home if they tell you that you are positive, your mum will be there and accept your status…*”

(*Female, 15years, IDI*)

“*I think parents seeing those messages on child’s phone will also pressurise them to encourage children of theirs to go to test yea. So me as a mother seeing that there is this message popping up on my child’s phone meaning that this message is important so I must also put something adding to that message. So it will also encourage parents to encourage their children to go test.*”

(*Female, 18years, FGD*)

Most youth felt that receiving incentives would encourage them to access HIV testing services.

“*I think giving youth incentives …will encourage them cause uumh our youth are being encouraged by just small things so if we are given something’s like hats, caps, t-shirts or bottles that will, they will go all over telling other youth that we got this and that so when you go testing you get this and that. So lot of youth will go there in order to get what other people got after getting tested*”

(*Female, 18years, FGD*)

“*…So if they go to the university and say after you test we will give you study guides or we will give you textbooks obviously cause I don’t have textbooks but not because I want to know my status but because I will get textbooks so that I can get education.*”

(*Female, 15years, FGD*)

“*Maybe around R50 or R100, people can come. If maybe they say when you test you get food, they’ll come cause they like food too much…*”

(*Female, 15years, IDI*)

“*… a voucher will help cause the youth likes money, Ummh a voucher to buy food, or just money.*”

(*Male, 18years, FGD*)

Some participants expressed a need for improved confidentiality during the HIV testing procedure.

“*… some of youth are afraid of the clinic cause of that some nurses are, don’t know how can I put it, but they just tell everyone and shout. You see that one that came here and walked. He or she is pregnant, I mean HIV AIDS and stuff, so we need some confidentiality sometimes.*”

(*Female, 18years, FGD*).

“*… another thing is… find a place where you know for sure that, your confidentiality won’t be revealed.*”

(*Female, 18years, IDI*)

Some youth reflected on negative experiences at the clinic and seemed to prefer HIV self-testing and testing at home either in private or possibly supported by a healthcare worker.

“*… I think it would be better if I do testing on my own because I will be having my own little privacy and I will be the one who will know if I’m sick or not.*”

(*Male, 15years, FGD*)

“*… I think that the idea of using machine for testing it will be good and also incorporating it into our home so that we can have privacy while testing.*”

(*Male, 15years, FGD*)

“*…it will work because the point of door to door campaigns the nurses or whatsoever can come bringing information and if you are already infected giving you treatment at home you feel more comfortable. Someone maybe scared to be going to a clinic often to receive treatment, at the clinic you see there is no privacy yea I think door to door campaigns will play a role*”

(*Male, 18years, FGD*)

#### Sub-theme: Receiving information and options for HIV testing

The majority of youth believed HIV education and awareness through platforms such as social media could promote HIV testing among them.

“*Yes, you see people my age really like social media. So, if ever such programs have some social media or groups. [vibrating cell phone] If ever there are people who I want to encourage, I can add them then convince them to join the group. (Male, 18years, IDI) “Because they’re always on social media’s. Yah. That’s the benefit. They’re always on social media’s so they’re able to see: okay, there’s this certain thing happening.*”

(*Male, 18years, IDI*)

However, some felt that using social media was not a good option.

“*No, cause social media like, is just to have fun so {people won’t take it seriously. Nah, it wont be, it won’t, it won’t, it wouldn’t be cool because {things coming from my phone}, I don’t take them seriously. Yah I think, maybe {if it’s a call and not a message, that will be better…}*”

(*Female, 18years, IDI*)

## Discussion

The prominent findings of our study were that majority of the youth perceived that they were at low risk to HIV, despite the high background HIV prevalence of 6.5% among youth in the Gauteng province [3]. Factors that seemed to make youth think they were at low risk were being young, absence of physical signs of HIV, being sexually inactive and parents not being HIV positive. However, they identified behaviours such as high risk sexual behaviours (having unprotected sex, multiple sexual partners and excessive drinking of alcohol), being victims of sexual abuse, road accidents and violent behaviour that increased their vulnerability to HIV. Most youth felt that parental support, receiving incentives, improved confidentiality and receiving HIV education and awareness information via digital platforms could motivate them to test for HIV.

An interesting finding from our study was that most youth with unknown HIV status perceived that they were at low risk of HIV; yet reported exposure to behaviours that placed them at high risk for HIV. It also seemed that youth were unaware that their risk would be high even when in stable partnerships [3]. Surveys conducted in other populations found that majority of participants reported low risk perceptions to HIV infection despite having engaged in high risk sexual behaviours [3, 23–25]. In other African countries, such as Zimbabwe and Ghana, similar findings were described where youth perceived their risk to HIV infection as low despite being exposed to high risk sexual behaviours [7, 26]. Nel et al. focused primarily on men who have sex with men (MSM), where 57% perceived they were at low risk to HIV infection [24]. Contrary findings were reported in Zungu et al.’s survey conducted in SA in 2016; where majority of participants aged 15 years and older perceived they were at high risk to HIV infection [27]. The majority of research that has investigated risk perception to HIV infection used quantitative approaches [3, 7, 23–27]. Although previous research showed similarities in perceptions of low risk to HIV infection, our study was different in that we used a qualitative approach and focused on youth aged 15–24 years, an age group identified as high risk for HIV infection [12]. Our participants had a low perception of risk but were exposed to various high risk behaviours related to their health, therefore there is a need to reach those who have low perceptions of risk to test for HIV.

Our study found that incentives may be an approach to motivate youth to test. This is consistent with previous studies which reported that incentives improved HIV testing utilisation [28, 29]. Additional research conducted in SSA showed incentivising participants was an effective approach, from the use of monetary, non-monetary and lottery-based incentives [30–32]. Similar to our findings, a 2018 study from Zimbabwe emphasized that hard-to-reach populations such as youth could be motivated to test if economic incentives were provided [30]. The majority of these studies used quantitative approaches [28–31] except for Ndyabakira et.al whose qualitative study focused on HIV campaigns which were paired with incentives that appeared to motivate men ≥ 18 years old to test for HIV [32]. Even though there is research evidence that incentives are effective, there is limited information on what youth preferred as incentive amount and type.

Youth wanted HIV testing procedures where there would be improved confidentiality at HIV testing locations and handling of HIV test results. In addition, youth in our study highlighted that enhanced privacy and confidentiality could motivate them to test for HIV. Similar findings were reported by Ntsepe et al., where FGD participants emphasized they would test for HIV if there was assurance that HIV test results would be kept confidential [33]. Furthermore, school learners aged 14–27 years who participated in a survey felt that lack of confidentiality of HIV test results and private setting posed as a barrier which deterred HIV testing utilisation [34]. Confidentiality was viewed as a critical element of HIV testing procedure which could facilitate or deter HIV testing utilisation among youth [12, 35]. In contrast, a South African multi-site cross-sectional study conducted with adults ≥ 18 years old found that most participants (95%) demonstrated they were satisfied that their HIV test results were kept confidential, the majority had used private health facilities [36]. Although youth in our study preferred testing at home it is also possible that they may not realise that being tested at home may not take place with a health care provider.

Youth in our study highlighted that having parental support would motivate them to access HIV testing services. Our findings were similar to another qualitative South African study that was conducted in Vulindlela district, Kwazulu Natal province where majority of youth decided to test for HIV following support and encouragement by their parents [12]. Findings from a cross-sectional South African study with parents of adolescents showed that 93% supported promotion of HIV testing among youth [25], thus parental support remained an integral part of the HIV testing process for youth [37]. Soon et al. noted challenges with parents where youth identified barriers to get their parents involved such as lack of communication [38]. In our study, youth emphasised that involvement of their parents when making important decisions such as HIV testing would encourage them. However, there is limited evidence on how communication and type of parental support could be integrated into future HIV interventions.

## Limitations and strengths

This study was not without limitations. The study was conducted in one district, Ekurhuleni in Gauteng province; therefore generalisability of findings could be limited. Consideration should made to the possibility of self-report bias to sensitive and personal questions which might have resulted from social desirability during data collection. Other limitations included observer bias. We addressed this by having different interviewers conduct the sessions and assessed transcripts for saturation of themes. Although we had limited screening data on those who were recruited for the FGDs, the enrolled sample provided information that helped us to reach saturation of themes. The sample of the study also included male and female youth from a region burdened with HIV/AIDS highlighting the importance of effectively engaging youth in HIV testing.

## Recommendations

The study was aimed at identifying ways to encourage testing for HIV; therefore, youth interventions on HIV education needs to be expanded to make them more aware of their risks for HIV. Moreover, adapted HIV testing procedures are necessary to promote privacy during HIV testing and communication of HIV test results.

## Supporting information

S1 File Anonymised transcripts. (ZIP)

S2 File COREQ checklist. (DOCX)
