# Supplementary material for: Exploring perceptions of low risk behaviour and drivers to test for HIV among South African youth
Source: PLoS One. 2021 Jan 22;16(1):e0245542. doi: 10.1371/journal.pone.0245542 (PMC7822253; doi:10.1371/journal.pone.0245542)
Supplement: S1 File — (ZIP) [file pone.0245542.s001.zip › S1_File_Anonymised Transcripts/(29)FGD 004 Males 15 to 17 years Translation_QC_TM.docx]

Full Participant ID: Focus group discussion

Participant Type: FGD 004: 15-17 year olds Males

Location: Winnie Mandela Clinic

Date: 06 Feb 2019

Start time:

Primary interview language:

Name of Facilitator/Interviewer: Thapelo

Name of Note Taker: Ency

Name of Translators: Kgopotso and Kganedi

Length of recording: Audio 1 (15:15) Audio 2 (31:15)

Label Key

I = Interviewer

P = Participant

N = Notetaker

{ } = Indicates that details were changed or pseudonyms were used to anonymise data

Xxx = words were omitted to anonymise data

- = breaking into a sentence by the next speaker

… = pause or drawn out words

[ ] = indicates noise made, e.g. [laugh], [sigh], [pause]

[Inaudible segment] = Unclear section of the recording

? Mulenga Clinic? P3? = questionable text or doubt as to what was said or who said it

**AUDIO 01**

I: What do you think makes people not to go for HIV testing?

P18: We are just afraid assuming that if I go to test and find out that I am positive, my friends will distance themselves from me because of my status. So as you have already mentioned that we always chilling as groups and it happens that you have the virus, eventually you will lose friends fearing to get infected also.

I: But what are the things that you see they can do for your age group to can improve the… err…mmm… the experience of testing for HIV?

P16: I think the best solution is that, the health department should consider taking part in the HIV projects by going house to house testing the people. People are not really comfortable to do tests on the street because everyone is watching them as they go to the gazebos/tents in a way there is no privacy for them. By going house to house, talks are avoided in the society that means privacy is not invaded and no one in the community will find out if you have tested or not.

I: Yah remember we are talking about the age group of 15 - 24 ok… What are the things that you think can be improved to have a large number of people coming to test for HIV?

P18: Ooh I recommend the idea of the previous participant about consulting house to house. When I get a visit from the HIV counsellors at home that way I will have enough time to pay attention as they break down the process of testing, the important of testing and why should I know my status. By doing that it will be easier for people to test and be knowledgeable about HIV because people fear going to enquire on the streets.

I: Owk [clears throat] and then you my guy, what is your take on what is being said?

P14: Ah… I do not have answer for that.

I: You do not have answer but when you think of your age group what are the things that can be improved in terms of testing and to motivate them to be encouraged to test for HIV?

I: [silence] Don’t you have an answer that side?

P16: Err… err…

I: You can also add.

P16: Err… The issue is that our parents are not educated about that, they should educate our parents first.

I: Mmh… yeah!

P16: Yeah they supposed to educate our parents. Problem is our parents, they don’t understand.

I: Mmh yah!

P16: They don’t know…they don’t have the knowledge at all. So sometimes it happens that a child tell the parent that they have tested though they haven’t, just to deceive other children and even the parent agrees to that statement made by the child to can avoid the child to test or take part, which is wrong. We supposed to educate our parents…uhm… so that they can motivate their children.

I: Uhm…

P16: Yes.

I: What do you think of the suggestion made? Since you seem to agree with his hypothesis… What do you think?

P18: Oh so I think if they can manage to educate the elders first, it will be simple for them to discuss it with their children based on the knowledge they have acquired. When parents are knowledgeable they will persuade their children about the importance of testing based on facts and as to why they should know their status. What I am trying to say is that parents are more influential to their children and it would not be a problem when a HIV counsellor approach the child to test because they were informed first at home about HIV.

I: Owk…err… As they have mentioned something about us going house to house… what do you think about that?

P14: Yah…err… House to house is really fine as people will get more information on their private space. You will even manage to go to homes where they are afraid to go consult fearing what the neighbours will say.

I: uhm…neh… yah. Owk you are correct there… may I ask since you mentioned house to house ok… err… uhm… How do you think your parents would feel about you… err… receiving information about …err … uhm… err… uhm… How do you think your parents would feel [sigh] when you receive HIV information or even getting the information from social media on your phones…ehm…?

P18: Oh Firstly when our parents hear the words HIV and testing, they having assumptions that I personally don’t trust my wellbeing [clears throat]. Parents know that I was born negative and to them it would be like my lifestyle is out of order when I consider testing for HIV. It will be difficult for the parent to allow the child to participate when asking them questions about HIV and in a way they feel like is bad luck or cursing when asking me about HIV. The parents will then say “I gave birth to you and I know you are clean”… uhm…So parents should be more knowledgeable about HIV to make the process simpler.

I: uhm… You can add more my friend.

P16: …err…The problem is that we not open to our parents as teenagers. We are supposed to be open to our parents… uhm … there must be openness with our parents… uhm … even if we face that difficult situation … uhm … Even if it happens they come across HIV information in my phone they will not get angry at me because I have been open to them …mmm… and I do share whatever I feel…uhm…it will be perfect.

I: My brother what exactly can we possibly do for your age group to can be more open with their parents?

P14: Educate our parents so that they can be supportive to us and be able to provide us with information about HIV so that they can push us to go test… uhm … them having the knowledge, they would not deny us going for testing… uhm.

P18 … Err… I think if the parents really check our books, they will see that we learning

I: Yeah at school…

P3?: You said is the testing for 15 to 21?

I: Yeah we targeting that age group.

P18?: If our parents check our books, they will see for themselves that the subject L.O (life orientation) teaches us about HIV, is a compulsory subject for everyone. So if parents check our books, they see that it is not something we hear it from the streets, clinic or a person passing by it is something that we learn at school and also the education system of South Africa stress a lot about the issue and it allows children to learn about it.

I:Mmh.

P16: They are supposed to consider the idea of creating a show that will only focus on addressing issues of HIV and to strongly encourage the age group of 15 to 21 years, because that age group is influenced by the media… to be exact social media.

P18: Adding to what the other participant has said about social media, we use social media to create a group whereby we can interact with friends and families…So it will be easy for us to share our thoughts and spread information about HIV -I: hum…

I: What are the… err… err…type of social media page do you think children err…mm… at your age group [clear throat] uses the most and [clear throat] it more influensive to them?

P18: Yah… I think it will be Facebook and Whatsapp because it’s easy to communicate with our friends and create group discussion with them based on the HIV topic, whereby we can share opinions, ideas or suggestions about it.

I: Uhm…err… which social media do you think …err…can be more….-

P?: Suitable …yeah…

P16: So it can beFfacebook, Twitter and Whatsapp. However What’sapp is the good one because it is more personal and secured for us to speak about this kind of things, unlike Facebook, it is used by everyone, it’s not that secured for us to use because everyone will see what we are talking about it

I: uhm…uhm…So you feel like is not a good thing that this kind of issues must be spoken about in Facebook right, why wouldn’t it be right?

P16: There are disadvantages and advantages of it.

I: Yes …just … tell us the disadvantages and the advantages…

P16: The disadvantages is that –

I: Mmh…

P16: Eerr… Facebook is used globally-

I: yeah… this means that everyone using it can easily be in contact with you [bang]

I: Yeah … iyooh! ... [Silence]. Let me check…. I was also recording [inaudible segment]… [Silence].

P3?: [Sigh] We got a bit of nice stuff, we did… I think so now I’m starting to think it wasn’t bad as I think [inaudible segment].

P3?: You speaking a different language in my class there [inaudible segment] [shouting] [sigh] [screaming] you pushed me [inaudible segment] [sport commentator] unusual ….should be a what [inaudible segment]…. By Pakistan South Africa 30 for 2 miss out the first 6 overs wait just have a look at South Africa and where they scoring the wickets had failed …. Just got themselves into a bit of a tango. Jaja pretty started nicely and see that Chatty Sondan he likes scoring down down [inaudible segment] all rounds has been absolutely brilliant and that will show [knocking]… [Sigh] a good thing is that boards as far as [inaudible segement] is concerned they need on 53 so they should sort of be, it not just about rams, it does help to sort of see where they are [inaudible segment] they must as well forget where off, well Muhammad beaten their attack… let’s go straight [inaudible segment] it’s has been a struggle indeed.

**AUDIO 02**

[Background voices and movement] I: Owh yah…

P16: Yah the disadvantage of Facebook is that it is used globally. So the disadvantage part of it is that you might have a quarrel with somebody, they will turn to crush your idea when you trying to utilise social media to create awareness about the matters of health. Our enemies will join the group created because Facebook would not restrict them to participate, that way they will use it to criticise you in a bad way…err… Whatsapp is very suitable and remember I told you that it is personal

I: Yeah…

P16: So that means you are only allowed to participate only when added by the admin of the Whatsapp group… Your contacts are the people whom you trust and communicate with on the daily basis compared to facebook

I: Uhm…

P16: So now the advantage of Facebook is that [silent] the message you post will be viewed by many people globally –

I: Uhm…

P16: The other disadvantage of Facebook is that, many people can view your post but ignore it and is something that is beyond your control. You can only advice them but it is up to them if they want to participate or not. Whatsapp plays a good role according to me not Facebook, Facebook is only crucial for marketing purposes –

I: Ummh… mmh. Uhm… err… What do you think my friend about social media and how can it be used to err…encourage people to be interested in testing for HIV err… Mxm referring to your age group of course … Mmh… to be interested in testing for HIV?

P18: I think Facebook is good considering the fact that is being used by almost everyone in our century. Facebook accommodates a large number of people across the world and that way we will be able to motivate one another about that issue as we break it down analysing it.

I: What other forms of social media you think they will be suitable?

P18: It can be Whatsapp

I: Mmh… It’s going to be a small group to communicate with a limited number of people, not heard by many people across the world.

I: [clearing throat] [paging through] [clearing throat] Okay…err… My brother what do you think about, what they have said err… about the disadvantage and the advantages of using…err… social media?

P16: I fully understand [Silence] what participant 18 has said.

I: Yeah… about the disadvantages of Facebook, because [silence] most children at my age do not work, so they can’t afford to buy data. It will be simple if they use Whatsapp because it does not use a lot of data. Therefore they will not be wasting information on people who just ignore it like in facebook, but inform those who are interested in it and also will pass it on to the next person

I:uhm….

P16: So I personally think that Whatsapp it’s suitable because only the person you know will be more interested to hear the information about HIV testing.

I: Uhm… how do you think we can send the information through Whatsapp?

P18: On Whatsapp?

I: In which ways can we send that information?

P18: I think if [Silence] owh I must say participant 18 owk…as participant 18 I think if we can started a group whereby we send links and information about HIV on Whatsapp it will be simpler to attract the youth to consider testing for HIV and also sharing their point of view about the importance of testing for HIV

I: uhm… [Silence].

P16: Participant 16

I: Uhm…

P16: Err … can you please repeat the question?

I: Owh the question was that err… because you also said we could use err… Whatsapp but I was asking that, how do you think err… we can get the message across using Whatsapp?

P16: Err…

I: Yah…

P16: It can be through links –

I: Uhmm…

P16: Yah links –

I: Uhm…

P16: Are more simpler and suitable to use –

I: Uhm…

P16: It would be good –

I: Umh…

16: Because even if someone is not interested but every time when a person comes across the link of testing for HIV on his/her phone that thing will annoy them. Eventually that person will join the group chat –

I: Hum...Yah…

P16: Yes –

I: work [clearing throat].

P18: Adding to what participant 16 said

I: Uhm….

P18: I personally think that if we hear it [clicking fingers] [silence] in Whatsapp, it will cost us less money because of Whatsappmonthly data. This way more people will be able to see links like house testing for HIV and knowing your status is important, obviously people will be attracted by the links and will want to know more

I: Uhmm…yah, yeah. My friend what is the other thing you think we can do on Whatsapp to give out more information?

P14: As participant 14; I think what participant 18 said about having subheading is a good thing because it will be easy to know what is the link based on and you can also be part of the group chat.

I: Uhm…

P14: To get more knowledge about the link -

I: Uhm…

P14: Of HIV–

I: Uhm…Okay err…May I please ask? In which way do you think we can inform the youth more about testing for HIV?

P14: The ways that we can use? –

I: yah …yah…

P14: Yah we can use flyers –

I: okay…

P14: At the certain area where you will be doing the testing–

I: Like the flyers…

P14: Therefore you have to distribute them days before to alert people to expect you on a certain date for testing rather than approaching each person you see on the road and telling that person she/he must come and test, it will offend that person and it will waste time [silence]… [Background voice]… [Sigh]

I: Okay err… [Door closing] you can speak participate18.

P18: Owh I was saying err… mmh [clicking fingers] what I was saying.

I: You were talking about flyers –

P18: Owh it is better to hand out flyers instead of approaching and telling them about HIV testing and you might find that, that person you approaching has lot of things going on in their minds by that time you want to give out the information. Handing out flyers would not cause any harm because you are just quiet and giving it to the people passing by the streets. Another… it is better to to give out the flyers on time not on the exact same day of testing –

I: Uhm…

P18: They should be distributed a week before testing to alert the public about the event that is going to be taking place. It will give a chance for the person to go through the flyer on their leisure time and they will come willingly without being persuaded to test

I: Uhm…err…

P16: Participant 16 –

I: Uhm…

P16: As participant 18–

I: Uhmm…

P16: Told us that they supposed to give out flyers–

I: Uhmm…

P16: But they have already did that–

I: Uhmm

P16: You have already put the flyers out there–

I: Uhm…

P16: And people are ignoring

I: Yes…

P16: The other problem is that we doing L.O (life orientation) and they do teach us about HIV but it is not enough. They are supposed to add a subject or a leasson that will strictly emphasise on HIV…

I: Uhm…

P16: We have heard a lot from the topics of poverty –

I: Ummh or what else,ummh… yes

P14: Err…participate 14–

I: Uhm…

P14: Err… as participate 18–

I: Uhm…

P14: Spoke about the distribution of flyers, I think that they must also distribute the flyers at school so that the children can be informed–

I: Uhm…

P14: And can also inform the parents.Therefore it would be easy for the parent to allow –

I: Uhm…

P14: Their children to be part of HIV testing.

I: Mxm… what do err… you think err… might sometimes prevent you guys from coming to test at the clinic? What are the things or experiences that you have heard from people who have been at the clinic before to test, whether good or bad experiences?

P16: Okay participate 16–

I: Uhm

P16: As I have heard from my friends–

I: Uhm…

P16: Yah saying that they have a problem coming to the clinic because maybe their neighbours will see them there. For example, maybe you want to take condoms and you are afraid that they might see you and will report you to your parents. That is how we get affected which result to us not coming to the clinic to test. We think the best thing to do, is for you to do the house to house thing.

I: Yeah

P16: That might reduce,uhm the course of many people not coming to testing. Eish I want to say they suppose to …? [Sigh]

I: Okay is fine participant 16, what are the things you have experienced that can discourage you to come test at the clinic?

P18: Err…basically as someone said before that we always in groups

I: Uhm….

P18: So within these groups when someone starts a topic about HIV –

I: Uhm

P18: Automatically they think that you have HIV and they say they can’t hangout with someone who has that thing –

I: Uhm…

P18: It might also pass on to them –

I: Uhm…

P18: Some people do not know how does HIV affects us –

I: Uhm…

P18: Like mxm I think if in school do teach us more about the disease it can reduce that mentality. As participant 16 has said that in school it is were we can gain more knowledge about the disease –

I: Uhm…

P18: Be movitvated to testing each and every year. So schools must make the HIV topic to be an additional subject –

I: Uhmm...

P16: Participant 16, I think maybe they can make a machine that does HIV testing, something similar to pregnancy testing type –

I: Uhm…

P16: On its own. It would be more suitable for HIV testing because you will have your own privacy and only you will know that you are HIV or not, alone –

I: Uhmm…

P16: It would be perfect –

I: Uhm. Err… do you think it would be perfect?

P18: yes…

I: Yeah yah.

P18: Oh –

I: Uhm…

P18: Participate 18, to add to what participate 16 has said, the thing that made many people to be scared of testing, they think that there is no privacy –

I: Uhm

P18: Like the person who is going to test them will know their status –

I: Uhmm…

P18: Compare to the ones who are doing pregnancy test, they can do it on their own and only them knows their results. I think it would be better if I do testing on my own because I will be having my own little privacy and I will be the one who will know if I’m sick or not –

I: Uhmm…

I: ××× Participate 14?

P14: Err… as participate 14, I agree with what participate 16 and 18 has said that we ought to have machines that are doing testing on their own. I also think that having these machines it will help us a lot because many people are afraid as participate 18 has said that they are afraid of testing because they think that there’s no privacy in the clinics. So as participate 14, I think that the idea of using machine for testing it will be good and also incorporating it into our home so that we can have privacy while testing .

P16: Uhmm

I: Can I ask my friends, what do you think about us providing incentives for the young people at your age group?

P16: So please can you explain the incentives –

I: Incentives are like gifts ok…like gifts or something given after testing… What do you think err…mmm…? How do you think that can encourage? What do you think about it, if they were to give [paper squashed] gifts after testing people at you age group?

P18: Err… err participant 18 –

I: Uhm

P18: First if the person is HIV positive, you need to know what are the proper food suitable for them obviously is healthy food –

I: Uhm maybe fruits or solid food atleast they will know that they have this kind of illness and those are some of the food I ought to eat –

I: Uhm… so that they can know that certain food or fruits contain this and that… Even the person who is negative should also eat healthy food and be more careful with mxm using protection.

I: Okay you know im referring to, let’s say after testing should –

P18: Ooh after testing…

I: Give you something –

P18: Ooh…

I: After testing what do you think of the idea of getting something after testing?

P16: I think it wouldn’t… wouldn’t work because err… life… life is more wealthy than a gift or something, so they are thinking err… beyond because like they thinking err… that gift would’t help me because I will be HIV positive, like my life would be bad for for the rest of my life –

I: Uhm…

P16: So it wouldn’t help but it’s better as I already told you that err… testing machines or something like that would be good because like err… that person will know that I am HIV positive and will be responsible knowing that they are HIV positive. The issue is that as young teenagers, we are getting sick and we already familiar with HIV and its symptoms I don’t want to lie, we know the symptoms but I don’t want to test –

I: Uhmm…

P16: Is because the word positive affect us inside.

I: What do you think of the incentives idea if they were to give after testing, do you think that will encourage young people to come more for testing?

P18: Err… others it would not encourage them –

I: ummh…

P18: Most of the people will be encouraged becauase many people in the townships love freebies –

I: ummh…

P18: Ummh

I: What are the things at your age group you think many people would be very fond of receiving them?

P14: Mxm many people would be happy to get things like phone –

I: Ummh…

P14: Computers xxx –

I: Ummh… ummh…

P14: Couple of things that will make them happy –

I: Yes. What other things you think as incentives they can give guys your age and be appreciated?

P18: Owk xxx it depends… on the mindset of the person –

I: Ummh…

P18: I think youth and teenagers would appreciate especially if you are HIV positive… As a person you would see that okay these people really care about me and are even keen to help me by asking what exactly is it that I want, so basically I should live a healthy lifestyle. It depends on the mindset of the person but you should consider getting fruits and veges as incentives to have a healthy lifestyle –

I: Ummh…err… [Background movement]… [Clearing throat]… [Background voices whispering] -We are just about… [Background voices whispering] [coughing] Okay err… my brother, what do you think err… about about… what [silent] what do you think about err… the things that you think they would appreciate receiving?

P16: Err… things that will encourage them?

I: Yah to encourage them to come for testing.

P16: Okay ah I think like as youth –

I: Ummh… as teenagers we like silly stuffs - ummh…

P16: Like err… things that are not health related such as beers, things like that –

I: Ummh… yah…

P16: You cannot say teenagers do not want or appreciate fruits and vegetables –

I: Ummh…

P16: Things that contain vitamins –

I: Ummh...

P16: They like silly stuffs mostly but phones would be suitable for them –

I: Yah…yah…

P16: Okay.

I: And then err… can I ask err… What is the err…experience we could get from using cellphones, like what are the advantages and disadvantages of using cell phone err…technology to register young people like you to can be informed about HIV through technology err…do you think there are challenges in terms of registering young people like you and err… err…giving them information through cell phone technology?

P16: I think it would err… not work –

I: Ummh…

P16: It is better to communicate face to face –

I: Mmh…

P16: It is much simpler to express your emotions when speaking to someone through the phone like replying them in a bad manner which is not good, so I think perso… personal is good –

I: Ummh…

P16: You are physically talking to one another and I think that’s great –

I: Ummh…ummh… What do you think about receiving err… information or registering for, for err… HIV services using cellphone technology?

P18: Err… participant 18, I think it is a good idea because it will reach so many people –

I: Ummh…

P18: And with the online –

I: Ummh… a person can speak out freely without being seen physically talking, to can avoid people saying things like “why are you saying such things?”xxx

I: My brother?

P14: I am… as participant 14, I agree with with par… participant 16 it would not work because when communicating with the person online, you do not see them and you will never know what would happen outside –

I: Ummh

P14: After sharing that with that online person –

I: Ummh. So you think it is a disadvantage to you using cell err… cellphone technology?

P: Yeah... –

I: Ohwk.

P16: Can I give an example –

I: Ummh… for example, your mother tells you to look after the house while she is away and she calls to check if everything is still in order, and you tell her everything is fine yet you know very well that you are with friends… so err… like is easy for a person to tell you lies on the phone than face to face… that is why they say our body is part of language and they say 89% of our body is how we communicate you can tell from the looks in the eyes if they telling the truth or not –

I: Yes.

I: Can I ask err… what are the things that you guys, people your age ok… can speak honestly about their sexual behaviours errmmh…err…mmh maybe to their parents or anyone for that matter… any suggestions you might have about that?

P16: That would be suitable on social media –

I: Mmh…

P16: Err because such topics are very difficult to can communicate with parents –

I: Mmh… it would be like an insult to them according to the way they have raised you things like that -I: mmh… So it is very important and I will be happy to have such discussions on the phone on such.

I: Mmmh… yes

P18: Err… what was the question?

I: Question says, what are ways or the suggestion you might have about encouraging err… youth your age to speak honestly about their sexual behaviours?

P18: Owk participant 18, I think that it will be fine if we do it face to face with a person who does not know any thing about you, like where you come from, how’s your family background like and how is your lifestyle like

I: Uhm…

P18: I think that social workers are the best because they do not know anything about you at all.

I: Uhm…

P18: so it will be simple to open up to them because they are at work and they do not know you at all -I: owk.

I: My brother, what do you think err…We can do to encourage the youth to be more open about their sexual behaviour?

P14: They have to be open to their parents and trust them with everything. Parents must also be understanding

I: Uhm… uhm…

P14: So that their children can be open to them, it must not be that thing when you tell them about something then they start to join into conclusion.

I: Uhm… Err...mmh … yah can you please tell us any final thoughs about err… err... Youth and accessing err… err… HIV services and also the use of gifts? What do you think about that as we are about to close….Any closing ideas?

P18: I think this thing of HIV testing on youth needs to be emphasised more to can get youth tested. Back then by the time HIV was discovered, it killed so many people because they did not know it and there was no cure for it. The information needs to spread as much as possible to reach everyone and show that HIV testing it is important.

I: Ummh.

I: [clearing throat] Any final thoughts about err… youth to access HIV testing services ummh any final thoughts?

P16: Err… as participant 16 –

I: Uhm…

P16: Err… my final thought is that err… as I have already said –

I: Uhmm…

P16: As I have already told you that –

I: Ummh…

P16: The machines would be suitable –

I: Ummh…

P16: In a way it would be a secrete –

I: Ummh…

P16: They will be encouraged to test –

I: Ummh…

P16: They will be able to test for themselves on their own and know their HIV status and how I am infected –

I: Ummh…

P16: You see –

I: Ummh…

P16: Is going to be a problem if you test people in public places, as you have already mentioned that we always walk as a gang –

I: Ummh…

P16: Yeah so if I am with my gang I will fear to test but if I have my own machine alone it would be perfect –

I: Yeah.

N: Okay ummh…

I: What could be the benefits when using gifts after you have tested?

P18: Owk I think if you use gifts it will attract people to come and test, it will also make the same people to tell other people about the gifts they got from a certain place because they were testing for HIV. You also have to test so that you can tell other about the things you got after testing.

N: Owk and what are the barriers or constraints of using gifts after testing?

P18: Barriers like?-

I: The challenges...

N: What will be the challenges of us giving you gifts after testing and the benefits for you to tell the next person?

P18: Owk I think that the challenges will be that we are always in groups and if you see a person being given a gift for testing, they will discourage the fact that why must they get gifts after testing. They bribing you to come and test.

P16: As participate 16, I think that err… it would be suitable –

I: Uhm...

P16: Because most of us are from poor families –

I: Uhm…

P16: So it would be better if they can produce a meal or something to feed us every month, because most of the families are poor that’s why today we find err… kids who are prostitutes, it is not like they love to be prostitues.The problem is that they do not have food at home –

I:Uhmm…

P16: So they are trying another way to survive from the poverty –

I: Yeah…

P16: So it would be suitable if there are such benefits, even the mother can encourage her child to go and test because the child will be getting food –

I: Owk … [Clearing throat] [Paging through]. [Paging through] okay guys now we have come to the end of our session ok.

P14, P16 and P18: Yes… and err thank you for participating and then errmmmh… if you have any questions err… you can contact us err… there are numbers on the consent forms that we have given you ok. [Paging through] err… thank you very much for participating and the end time is umh…17:21, thank you so much guys ok -P14,P16 and P18: Owk… sharp [chair movement] wait a bit.

End Time: 31:15
