# Supplementary material for: Exploring perceptions of low risk behaviour and drivers to test for HIV among South African youth
Source: PLoS One. 2021 Jan 22;16(1):e0245542. doi: 10.1371/journal.pone.0245542 (PMC7822253; doi:10.1371/journal.pone.0245542)
Supplement: S1 File — (ZIP) [file pone.0245542.s001.zip › S1_File_Anonymised Transcripts/A01-029-TM-MALE 15-17-ENGLISH_QC2_TM.docx]

Full Participant ID: A01-029-TM

Participant Type: Male

Location: Winnie Mandela Clinic

Date: 22 September 2018

Start time: 08:58am

Primary interview language: Sepedi/English

Name of Facilitator/Interviewer: Bakang Mosime

Name of Note Taker: None

Name of Transcriber: Bakang Mosime

Length of recording: 29min: 40sec

Label Key

I = Interviewer

P = Participant

N = Notetaker

{ } = Indicates that details were changed or pseudonyms were used to anonymise data

xxx = words were omitted to anonymise data

- = breaking into a sentence by the next speaker

… = pause or drawn out words

[ ] = indicates noise made, e.g. [laugh], [sigh], [pause]

[inaudible segment] = Unclear section of the recording

?Mulenga Clinic?, ?P3? = questionable text or doubt as to what was said or who said it

I: Do you allow me to record our conversation?

P: Yes. [Papper Shuffling]

I: Can you explain to me uhm… your thoughts about HIV? What do you understand by the term HIV?

P: Err… [Papper Shuffling]

I: Anything you know about HIV… What do you understand by the term?

P: Ahh, maybe when someone is injured… or maybe when someone got injured, having been stabbed with a bottle and you have a wound as well. If the blood gets into to your wound, you can be infected as well.

I: [Noise from outside] Ok… Ok… what else do you know about HIV? Ok… what else do you know about HIV?

P: Through sexual intercourse

I: Mmh… What is it about sexual intercourse do you know? How is HIV related to sexual intercourse?

P: Is like when you having sex with someone who is HIV infected and you not.

I: What kind of sexual intercourse will that be?

P: [pause for 4 sec]

I: Do you think even when people are having sex using condom HIV one can get infected by HIV?

P: When you not using condom.

I: Do you mean, through unprotected sex?

P: Yah… [Paper shuffling]

I: Ok. So can you tell me, how one can get infected with HIV? Like you’ve said before, when is the person is most likely of getting HIV?

P: Just… just like when…Err… [Noise outside] Just like when someone is born with HIV.

I: Ok, what else? What are the ways that one can get infected by HIV?

P: Some of the ways that a person can get HIV infection is when… Err… [Paper shuffling] sometimes you have an open wound and you trying to help someone who is bleeding, when their blood get into your wound, you can get easily infected.

I: So, can you explain to me at what time or when have you felt you were most at risk of getting infected with HIV?

P: [Pause for 2 seconds] _

I: in your experience have you met an injured person trying to help them while having a wound? Or have you had an unprotected sex maybe?

P: Aah… never

I: So aren’t there any possibilities of you being at risk of getting HIV?

P: No

I: Ok, can you tell me the situation that you felt you may have been at risk of getting HIV?

P: ErR… I don’t understand.

I: Err… Have you ever been in a situation where you felt you might being infected with HIV?

P: No… No there’s none

I: Ooh there’s none? _

P: Yes

I: Ok, so can you tell me about the HIV services that are taking place in your area?

P: [Pause for a second]

I: What kind of testing services do you usually see happening around your community?

P: How so?

I: Where does the testing services for HIV usually take place?

P: Err… sometimes there are people with tents doing HIV services around_.Gazebo: Yes, some are doing HIV testing services in some schools.

I: So even at schools they do HIV testing services?

P: Yes.

I: Who are the people testing in the schools?

P: There will be people asking to get inside the school to do HIV testing services to adults._

I: Ok_

P: Yes, adults

I: They only test adults?

P: Yes

I: At what age do they start testing people?

P: Err… from 18 years and above.

I: Ok, [Paper shuffling] and then less than 18 they do not test?

P: Yes… As for us they do not test

I: Ooh…Ok. But if they were to test your age group (15-17), would have maybe got tested?

P: [People laughing]. Yes, some would have went and got tested. [Noise outside]

I: Mm…ok. So where exactly the HIV testing services are are normally taking place? Where else do you think the HIV testing services for youth are taking place in your area?

P: Mm… Maybe at the clinics

I: Ok_

P: Yes [Noise coming from outside]

I: Where else?

P: Hospital.

I: Ok [Mute for 2 seconds]… So can you share your experience trying to access these services?

P: Mm… No… I never tried.

I: You’ve never even went close to getting tested?

P: No no…

I: In your opinion what are the positive about current HIV testing services that are available to youth?

P: It is important because it helps many people to know their current status_

I: Ok_

P_ Yes… So that you can get help, maybe they transfer you to hospital for help and treatment

I: Ok

P: Yes [Paper shuffling]

I: So do you think these HIV testing services are Important to youth?

P: Ee… Yes they are important.

I: You just told me the positive aspects of HIV testing services that are available to youth. Now can you explain to me what the negative aspects of these services (HIV testing services that are available to youth?)

P: Oh, you mean those that are not important?

I: Yes, what is it you think is negative with HIV testing services that are available to youth?

P: [Silence for 2 seconds]_

I: Like you said it’s important for youth to go to HIV testing services, so that they know their status and treatment_

P: Yes…_

I: So now can you explain to me what are the negative aspects about the current HIV testing services that are available to youth?

P: People could die if these services are not available to them.

I: So are you saying all these HIV testing services that are taking place in your community are helping people?

P: Yes, they are very important.

I: If they are not important, what could you think of that is not important?

P: Err… [Silence for 4 seconds]_

I: Anything that you can think of? _

P: Ee… maybe they could get sick.

I: Ok, so you saying HIV testing services are helpful to communities?

P: Yes, they are helpful.

I: How do you think incentives can be used to encourage youth to tests for HIV and access treatment?

P: [Silence for 2 seconds]

I: Do you understand the question?

P: No No…

I: How do you think incentives can be used to encourage youth to tests for HIV and access treatment?

P: [Silence for 2 seconds]

I: Ok, so maybe before you could answer this question, let’s explore the word incentive. Can you explain to me, what you understand by the term incentive?

P: No

I: Ok, according to your own understanding, what do you think incentives are?

P: Err… maybe [silence for 3 seconds]… maybe when you give someone something.

I: How? Can you explain further?

P: Maybe through sexual intercourse.

I: Ok, let me explain to you._

P: Yes._

I: Incentive is something that I can offer you in a way of thanking you to take part on the service that I might be offering you. Do you understand better? _

P: Yes. _

I: So do you think if we were to offer these incentives to youth would they be able to access HIV testing services and treatment?

P: Yes, they can access those services.

I: So what kind of incentives would youth value most in order to access HIV services and treatment?

P: Maybe some clothes.

I: What type of clothes?

P: Maybe T-shirts, Trousers, Cabs.

I: What else, you can mention as many as you can think of, that youth could value and get the courage to come and test for HIV and access treatment.

P: Shoes, _

I: Ok, what else? _

P: Err… [Silence for 5 seconds] food.

I: What type of food?

P: Maybe bread.

I: And what else can you think of?

P: Maybe some burgers [paper shuffling]

I: Is that all?

P: Even pies. [Phone ringing]

I: What else?

P: That will be all.

I: Ok, so how often do you think we should give these incentives?

P: Whenever they come to get tested. Is then they should get them.

I: Ok, we should just give them only when they come to test?

P: Yes.

I: So what could be the challenges of giving out these incentives?[Message notification on the phone]

P: [Silence for 4seconds] _I: What challenges can we possibly face by giving out these incentives?

I: Any challenge you can think of?

P: People can come to test in big numbers.

I: That could be the benefits.

P: There can never be challenges

I: Ok, please describe to me your thoughts about being contacted on your telephone [Message notification on a cell phone] or via social media informing you about HIV testing services that are available to youth?

P: I don’t understand.

I: Ok, please describe to me your thoughts about being contacted on your telephone or via social media informing you about HIV testing services that are available to youth?

P: According to myself, I believe it could be the right thing to do[Birds Noise]

I: Can you explain to me further, what you mean?

P: Sometimes you might be having problems, maybe being sick but not knowing exactly what the problem is might be.

I: Ok, and how will you feel about being informed or registered for HIV testing services via your cell phone?

P: I think that could be a good thing, because most of the people might not know where the HIV testing services are taking place in their communities.

I: Ok, so can you please describe challenges that youth might face by receiving information via their cell phone?

P: Err [Silence for 2 seconds] some of them might ignore them.

I: Ok, Other challenges you can think of?

P: Some might see it as a good thing.

I: So how could cell phone be used to inform youth about HIV testing services?

P: By sending them messages.

I: Ok, any other ways?

P: Even if they could phone them. [Phone ringing]

I: So you don’t think that might be a challenge?

P: No.

I: So you told me about the challenges, what could be the benefits?

P: The good thing will be to alert them about places they could go to for HIV testing services.

I: In your opinion what type of social media could be used to contact youth for HIV testing services?

P: In… [Paper shuffling] _

I: Do you know what social media is? _

P: Yes, I know social media, mm… Facebook, Whatsapp and internet

I: Can you explain what should be on internet?

P: So they can have a page that we can download information about HIV testing services.

I: In all these social media platforms, do you think they should be accessed for free or they have to be paid for?

P: They should be for free.

I: What could be the challenges of using social media to inform youth about HIV testing services?

P: Err… Please explain further.

I: Err... What could be the challenges of using social media to inform youth about HIV testing services?

P: Some will feel like is a good idea, whilst some won’t feel good about it.

I: Ok, can you explain to me what could be the reasons behind all of those feelings?

P: Because some don’t understand how serious HIV testing is.

I: What could be the benefits of using social media to inform youth about HIV testing services?

P: It could help most people to know where are the HIV testing services taking place in there are.

I: So, can you explain to me how will your parents or legal guardian feel about you receiving information about HIV testing services on your phone?

P: They will be happy knowing that I’m responsible and that I’m doing a good thing.

I: Does this mean your parents are open to discuss everything with you, including sexual behaviours and opportunistic disease?

P: No, they don’t.

I: So do you think, it will be a good idea to if we teach parents about HIV and how to communicate with their children?

P: Yes.

I: How can we reach this parents? Or how can we make them access such information?

P: You can door to door campaign to teach our parents about HIV or by sending messages to ask them to come to the clinic to learn about HIV.

I: Do you maybe have any final thoughts of encouraging youth to access HIV testing services and treatment?

P: Is that they should know their status before anything else, so that they can start taking treatment early.

I: Are there any other ways that we can encourage youth to access HIV testing services and treatment?

P: By doing school campaigns.

I: How about we bring both parents and their children to get information about HIV? Do you think youth can feel free to talk about HIV in front of their parents?

P: No, they won’t feel free. You need to separate them.

I: So, what do you think of stationery being part of incentive?

P: That could be a good idea.

I: What should the stationery pack be consist of?

P: Books, pens, rulers and pencils.

I: What type of books? Reading books or writing exercise books?

P: Both reading and writing books.

I: And what else.

P: That will be all.

I: Any final thoughts about youth, HIV testing services or incentives?

P: No

I: Ok, thank you for agreeing to take part in our study, we have come to the end of our conversation, for any further information you can contact the numbers I showed you on the Consent form. Thank you once again.

Time Ended: 9:30am
