# Supplementary material for: Exploring perceptions of low risk behaviour and drivers to test for HIV among South African youth
Source: PLoS One. 2021 Jan 22;16(1):e0245542. doi: 10.1371/journal.pone.0245542 (PMC7822253; doi:10.1371/journal.pone.0245542)
Supplement: S1 File — (ZIP) [file pone.0245542.s001.zip › S1_File_Anonymised Transcripts/A01-030-TR_Translation_Reba.docx (updated version)_QC2_TM.docx]

Full Participant ID: A01-030-TR

Participant Type: Male

Location: Winnie Mandela Clinic

Date: 28 September 2018

Start time: 10:20

Primary interview language: English/Setswana

Name of Facilitator/Interviewer: Bakang Mosime

Name of Note Taker:

Name of Transcriber: Reba

Length of recording: 34:35

Label Key

I = Interviewer

P = Participant

N = Notetaker

{ } = Indicates that details were changed or pseudonyms were used to anonymise data

xxx = words were omitted to anonymise data

- = breaking into a sentence by the next speaker

… = pause or drawn out words

[ ] = indicates noise made, e.g. [laugh], [sigh], [pause]

[inaudible segment] = Unclear section of the recording

?Mulenga Clinic?, ?P3? = questionable text or doubt as to what was said or who said it

I: Do you allow me to record this converstion?

P: Yes.

I: [turns page] Okay. Can you describe to me your thoughts, what do you understand by HIV?

P: Mhmm…HIV, I understand, like…blood.

I: Mhm. [pause] What about blood? Can you explain further what you know about HIV?

P: Mhmm… I don’t know much about AIDS.

I: You don’t know much about HIV?

P: Yes.

I: And when you say blood, what do you know about blood?

P: Someone with HIV?

I: Mhm.

P: When you touch someone’s blood…

I: Mhm.

P: That has HIV/AIDS…

I: Mhm.

P: You will get AIDS…HIV/AIDS. That’s all I know.

I: Okay. That’s all you know?

P: Yes.

I: Uhh, can you tell me what places a person is mostly high…uh at risk of getting HIV? In which situations can a person be at risk of getting HIV?

P: At parties…

I: And then what…what? Hm? How would a person get HIV?

P: [pause] Others are born with it…

I: Okay. Another way that you think a person can get HIV is?

P: [pause]

I: Anything that you can think of…of how a person can get HIV?

P: Sleeping with someone who has HIV when you do not know, then you can get it.

I: Okay.

P: Without using protection

I: Protection…? So when we speak about protection, what are we talking about?

P: About condoms.

I: Okay. So which places…in which places do you think a person can mostly get HIV from?

P: Like how? Places?

I: Yah. You spoke about parties right?

P: Mhm.

I: Where else?

P: Mhmm… even at home, in places that are not okay.

I: Places that aren’t okay are places like what?

P: Places that have… [pause] pollution. Many pollutions cause dieseases

I: Okay. And what are other places you can think of?

P: [pause]

I: Are those the only places you know?

P: Yes.

I: ?[inaudible segment]? [3:41] So can you tell me about where HIV testing services happen where you live?

P: …

I: Where do they test for HIV?

P: Close, close to white one…house.

I: Okay.

P: Theres some other place there…where they have tents.

I: Okay. Who, who tests there?

P: The people that go, I don’t know them.

I: Oho…so all you see is tents?

P: Yes.

I: Okay.

P: They are written on the outside.

I: So what about the youth, where do they go for HIV testing services?

P: Some of them they go to clinics.

I: Okay. So you think youth prefers going to the clinic or at the tents for HIV testing services?

P: Most of the people go to the tents.

I: Oho.

P: But I prefer to come to the clinic.

I: Why do you prefer the clinic?

P: Cause… there, I don’t trust it there.

I: Okay.

P: You can see that after they test another person, they test you with the blood that they tested that other person with.

I: Oh so that’s the reason why, why don’t you trust them?

P: Yes.

I: So at the clinic, why do you trust them?

P: Because after testing someone, they put dustbins right next to them and throw away the things they used to test you with.

I: Okay. Have you ever…have you ever wanted to go and test?

P: No.

I: Haven’t you ever tried to go test one day?

P: No…

I: So you don’t have any experience of HIV testing?

P: Huh?

I: Don’t you have experience of testing?

P: Yes.

I: What do you think is important, when you are thinking from your head?

P: Yah.

I: What do you think is important about testing?

P: …Knowing..

I: Mhm.

P: Your status…

I: Mhm. So the testing services that you have been talking about from the clinic and the ones from the community with tents. What is positive about them?

P: Huh?

I: What do you think is important about them?P: With what? With testing?

I: Yes.

P: Mhmm…

I: Do you think that those people from the tents and the nurses from the clinic…- How do they help? How is what they do important?

P: [pause]

I: Anything that you can think of. How do they help people?

P: …By healing them

I: By healing them?

P: Mhm.

I: How do they heal them? Could you explain to me how they heal them?

P: They give them medication at the clinic…

I: Mhmm…okay. And then you told me what is important important about HIV testing services right?

P: Mhm.

I: So what do think [phone vibrates], is not important about HIV testing services?

P: …

I: Like you have already told me about how you are afraid of the tents because they could use something that they have used on someone else… What other things can you think of that can happen? That are not…that are negative about HIV testing services?

P: …[pause] Uh…

I: Is there nothing that you can think of? [Inaudible segment]? [7:40]

P: Okay.

I: Okay so…how do you think incentives can be used to encourage youth for HIV testing services to access…and access treatment? How do you think incentives could help youth to come and get tested…and to get treatment…for HIV?

P: … Even here you could do it, you just do a small event…

I: Okay. Let’s understand the word incentive. What do you think incentives mean?

P: Incentive?... Uh like, some you give me…

I: Okay.

P: Like sharing.

I: What are we sharing?

P: It could be a bottle…

I: Oh okay…

P: Mhm.

I: So do you think that if we give out those things that you are talking about, like bottles…people could be encouraged to test for HIV and to get their treatment?

P: No.

I: Why are you saying no? Can you explain to me why you are saying no?

P: Hmm…

I: Could you give me reason maybe, of why you are saying no?

P: Because I could be, I could have AIDS…I would drink and give it to you. You would also drink; you would have the possibility of getting it.

I: Okay… So what, what kind of incentives do you think we could give, that you think youth with be happy to receive if we said they should come and get tested for HIV and to get treatment?

P: …

I: Anything that you can think of, you have already spoken about bottles right?

P: Mhm.

I: What others do you think that youth would be happy to receive? [turns page]

P: [pause] Like the way you guys keep doing, you come to school...you teach us and explain to us that AIDS does this and this and this. Or you could open an event…right there at the park…

I: And then what is, you explained that an incentive is something that you are given so that you can come and test. So what kind of thing can we give you that could encourage you to come and test? Please give me examples.

P: What?

I: Examples of things that we could give you…to come and test for HIV, what are they?

P: … Clothes…

I: What kind of clothes?

P: …

I: With clothes there are shirts and pants, which ones are you talking about?

P: T-shirts… like…

I: Okay.

P: Caps…

I: Okay.

P: Yah.

I: What kind of T-shirts?

P: … Like half or?

I: How would you like the shirts to look like maybe? In color? What kind of color?

P: Maybe red, red and black.

I: Okay. On those shirts, should we write something or should they just be plain shirts?

P: They should write something?

I: Like what? The message that we could write should be what?

P: Hmm…

I: Any kind of messge that could make you feel alright?

P: …Teenager or?

I: Oh we should write teenager?

P: Mhm.

I: Okay. What others do you think you would like? Even if it is not you, what would others like? What would attract youth, what would attract youth to come and get tested?

P: Most of the kids at school need phones…

I: Phones?

P: Mhm.

I: Okay phones…and what?

P: … [sigh]

I: Okay let’s explain the phones, what kind of phones?

P: …Any kind of phone.

I: Any kind of phone?

P: Mhm. It could be a touch phone or it could a phone with buttons…

I: Okay. Anything else you can think of that could attract the youth are?

P: Hmm…watches, laptops…

I: Watches and laptops?

P: Yes and umbrellas.

I: Umbrella…and what also?

P: …

I: In some of the interviews that we have been doing, there are people that talk about food. Would you be attracted by food?

P: Yeah.

I: What kind of food?

P: Hmm… it could be macaraoni or bread…or juice.

I: Would you like food that is cooked or food that is not cooked?

P: That is cooked.

I: Okay anything else that you can think of?

P: What? Food?

I: Yes, food...

P: Hmm… Burgers or…[pause]

I: Okay. If we could give out stationery?

P: Yah even that would be okay.

I: So with stationery, what do you need, what does the youth need with stationery?

P: Glue…

I: Glue…

P: Pencil.

I: Pencils…okay.

P: Others don’t have bags. Yes, schoolbags.

I: Schoolbags?

P: Yes.

I: Okay. What are other things?

P: Hmm…sharperner, scissor…

I: …Okay.

P: Hmm…ruler.

I: So how often do you think that we should give out these things?

P: Huh?

I: When we give out these things, what should be happening?

P: If you want the youth to test…?

I: Mhm.

P: You could tell them, like you go to the schools…any school. You get there and tell them there is an event….and this and this person is coming…

I: Mhm.

P: Come and we will give you this and that and that. Yah, they can come and…

I: Oh so we should give them out only when you test?

P: …Yah.

I: Uhh... What could be a challenge for giving you guys these things? What problems could we have?

P: ... Like how?

I: When I give you a T-shirt or cap, right? To come and test…what is it that you think would be a problem while we are giving them out? What could happen?

P: … If there are other kids that couldn’t get?

I: Mhm.

P: They could fight Yah.

I: Fighting for what?

P: Fighting that others got stuff and they didn’t get anything…

I: So there should be enough?

P: Yes

I: Okay. And what would benefit you from getting these things after testing?

P: They would be happy…

I: Okay they would be happy; what other benefit is there?

P: Hmm… No…that’s all

I: That’s all?

P: Yah.

I: Do you think that by giving those incentives, people will be happy…to test? Will they be happy to test for HIV?

P: Some of them.

I: Mhm. Some of them? So what about the others? Will they not be happy…why do you think they won’t be happy?

P: … They will, they will be scared of testing…

I: What will they be scared of?

P: I don’t know… They are just scared.

I: Yes okay. What is scary about testing?

P: Like someone who comes back from testing, tells the other person that they prick you. Now they start becoming scared.

I: Oho… They are scared of being pricked?

P: Yah.

I: They are scared of the needle?

P: Mhm.

I: Okay. What else do you think people are scared of to get tested?

P: … [pause] No that’s all.

I: What are you scared of with testing?

P: I’m scared of that thing, like after they test another person…

I: Mhm.

P: That they would test me with the same…-

I: Same needle?

P: Mhm.

I: Okay. So what do you think we, as health workers, could do to make people less scared of getting tested? What can you think of that could help people to stop being scared of testing?

P: You could have a lot of needles…and then they could come…uhmm…

I: Mhm.

P: [pause]

I: We should have a lot of needles?

P: Mhm.

I: Okay. So can we discuss your thoughts, maybe describe to me your thoughts about being contacted via telephone or social media for HIV testing services?

P: Hmm…

I: Should I explain to you?

P: Mhm.

I: Please explain to me how you feel if we called you or sent you a message via social media telling you about HIV testing services?

P: How do I see it?

I: Yes.

P: It could be okay…

I: It could be okay? Why, can you explain to me why you see it as being okay?

P: Or if you told the person face to face…

I: Mhm.

P: They could get scared and not come. And they could tell others that they do this and this where…

I: Okay. What would make them scared when they are being told face to face? What do you think makes them scared when talking to you…. face to face? What would make you afraid?

P: Hmm…it’s because the people in the tents do not change their needles.

I: Mhm.

P: They don’t throw them away

I: Mhm. Okay.

P: It’s like they just wipe it…and use like that.

I: So if we contacted you via your phone or social media for HIV testing services would you feel okay?

P: Mhm.

I: Okay. Can you explain to me if you have ever heard about HIV testing services and how?

P: Hmm…

I: Around where you live. How did you know that there is are places to test around where you live?

P: On their tents…

I: Mhm.

P: It’s written that ‘get tested today’

I: Okay. Other ways that you have heard about it is being tested around?

P: From people talking…

I: Mhm…okay. And then how would you feel being informed and registered for HIV testing services using cellphone? How would you feel if we used cellphones to register you to come test, do you think it would be a better option for you or what?

P: Yah. Yeah it would be a better option.

I: …Being, being told via phone?

P: Mhm.

I: [tongue snaps] What other ways can you think of that we can use cellphones to tell people about HIV testing services?

P: … Like, uhh…Whatsapp, Facebook…

I: Okay and what?

P: And social media…

I: Mhm…social media. What other ways are there?

P: Uhh… Like giving someone letters…

I: Okay.

P: Forms…

I: Pamphlets right?

P: Mhm… [pause]

I: [birds chirping] Okay. And what challenges are there that you think youth may experience when we tell them via phone or through social media about HIV testing services?

P: Yoh, I don’t understand…

I: What challenges are there, would the youth be happy if we called them or if we told them via social media that there testing services, HIV testing services around their community?

P: Like would you post on Facebook?

I: Yes, do you think a lot of youth would like that approach?

P: Some of them, some of them…

I: Mhm. And other, some of them who might not like it, what do you think would be the challenge for them?

P: [sigh]… Hmm… [pause]

I: Okay let’s talk about benefits…of being contacted via telephone or social media. What would you guys benefits if we told you about HIV testing services around your community via telephone?

P: [pause]…hmmm. It would make us realize that HIV is a disease that…mhmm…like… [pause]

I: Say anything that you can think of…

P: …Like a bad disease.

I: Okay. So social media, do you think that social media could help to…to inform youth about HIV testing services?

P: Yes.

I: How are they going to reach it? How are they going to access it social media?

P: Even if it isn’t social media, if it is Facebook?

I: Yes

P: You just create a post, you do it there and you say ‘come to Winnie Mandela Park’ and then you’ll receive goods…

I: Mhm. Okay… So a lot of youth uses Facebook?

P: Uh…yes.

I: Okay. So when they use Facebook, do they use it for free?

P: Others, others use Free Mode and other buy data.

I: So just as we were talking about incentives, don’t you think or maybe…we could add data, do you think we should add data into incentive? Some of the things that we can give to the youth?

P: Yes.

I: Okay. So you, personally, how do you think your parents would feel if you got HIV testing services on your cellphone or social media?

P: They would feel okay…

I: [laughs] Why do you think they would feel…can you tell me why they feel okay?

P: Cause they ewant us to prevent dieseases…

I: Okay. So they would be happy that you are getting these messages?

P: Yes.

I: What do you think for other youth, what would their parents do, do they their parents would like it if they got messages like that?

P: Hmm… maybe.

I: Mhm. Why, can you explain why you are saying maybe?

P: Because there are parents who are strict, they won’t allow their kids to get messages…

I: Mhm. So strict parents, what would make strict parents get to a point were they understand about HIV and make them come together with the youth and ther children? What do you think can be done to teach these parents about HIV testing services?

P: Like you could make forms…

I: Mhm.

P: So that they can get home and give their parents and they can read the information about the disease.

I: Mhm.

P: About HIV/AIDS. So that they can understand.

I: Okay. So we should make pamphlets so that these parents can read?

P: Yes.

I: Mhm. Okay. What else can you think of that we can do to help parents understand about HIV testing services and to talk to their children about it?

P: …You could set up a meeting, if they come…you explain to them what HIV does…

I: When we have this meeting, should be parents only or should it be parents and their children?

P: If parents don’t understand…

I: Mhm.

P: It will just be them…

I: Okay.

P: If the children also don’t understand…you can combine them.

I: So, so … It could be a meeting of parents and children, where children can explain to them their challenges?

P: Yes.

I: Okay… So do you think that sometimes when parents do not want to understand about HIV testing services and the youth, is it because of beliefs?

P: Yes.

I: Mhm. What would be happening?

P: They would be going by the rules of their beliefs…

I: Mhm.

P: Like church for instance…

I: Okay. What does church do?

P: They don’t allow…

I: They don’t allow them to test for HIV?

P: Yes.

I: Okay.

P: Some of the church.

I: Some of the church…not all of them?

P: Yes.

I: Okay. So what do you think we can, as health workers we could go to the churches…and get there and explain to them so that they can understand about HIV testing?

P: Mhm. Yoh it’s tough…

I: It’s tough…

P: Yah.

I: It’s tough?

P: It’s tough…

I: Okay. So any suggestions maybe about how we could encourage the youth to test for HIV?

P: … Post. Post something on Facebook or social media, and show them that when you come to test, we gonna give you goods…like caps…

I: Okay.

P: Clothes, food…

I: I heard at, in the beginning of our conversation you spoke about maybe we could have parties…?

P: Yah.

I: So what kind of parties could we have?

P: You hire celebs, any celeb….as long as the person knows what they are doing. Artists from hip-hop, house, kwaito, jazz music…

I: So the youth? House, kwaito, Jazz? What does the youth like from all of them?

P: They like hip-hop and house

I: So we should call hip-hop artists?

P: Mhm. And house artists

I: …What other events can we do that you think the youth will come to?

P: Hmmm… Soccer…

I: Mhm.

P: Like sports.

I: Sports?

P: Yes. [sniffs]

I: Okay. What kind of sports?

P: Soccer…

I: Okay.

P: Hmm…netball.

I: Netball… And what also?

P: Cricket…

I: So…at a sporting event, how should it be? How would it be in order for the youth to enjoy themselves? Should it be sports from the start until the end or should there be…?

P: Yah. Like we could start by playing soccer first and after the soccer ends, netball starts and after netball, cricket plays.

I: Okay. While there are people who test in between or…? Should we teach them about HIV?

P: Yah.

I: Or should it just be games, there shouldn’t be HIV testing services?

P: They should be there…

I: Okay. [birds chirping] [sigh] Okay, any final thoughts about youth, HIV or incentive? What can you, do you have any last thoughts about everything we have spoken about, youth, incentives…what can you add?

P: Yah.

I: Mhm.

P: That the youth has to know that HIV is a dangerous disease…

I: Mhm.

P: They have to come and get tested…at the clinic.

I: Mhm.

P: Yah.

I: How would we let them know? We spoke about social media and phone, what other platform do you think it could help us spread the message about testing, HIV testing services?

P: You go to schools and you explain to them…

I: School campaign…thank you.

P: Mhm.

I: What other things can you think of?

P: Hmm… like, like drivng around in a car, while talking…you place mics and speakers in the car and speak.

I: Mhmm…oh okay.

P: Then people are walking while listening.

I: Okay. Anything else?

P: Yah…that’s all.

I: That’s all you can think about?

P: Yes.

I: Now we’ve come to the end of our discussion, thank you for your participation. If you have any questions…you can call the numbers on your informed consent. Between 8am and 5pm…Thank you once again.

End time: 10:50
