# Supplementary material for: Exploring perceptions of low risk behaviour and drivers to test for HIV among South African youth
Source: PLoS One. 2021 Jan 22;16(1):e0245542. doi: 10.1371/journal.pone.0245542 (PMC7822253; doi:10.1371/journal.pone.0245542)
Supplement: S1 File — (ZIP) [file pone.0245542.s001.zip › S1_File_Anonymised Transcripts/A01-043-CM_Translation_Reba_QC2_TM.docx]

Full Participant ID: A01-043-CM

Participant Type: Male

Location: Winnie Mandela Clinic

Date: 8 October 2018

Start time: 16:00

Primary interview language: English/Setswana

Name of Facilitator/Interviewer: Bakang Mosime

Name of Note Taker:

Name of Transcriber: Reba Ramoroka

Length of recording: 24:47

Label Key

I = Interviewer

P = Participant

N = Notetaker

{ } = Indicates that details were changed or pseudonyms were used to anonymise data

xxx = words were omitted to anonymise data

- = breaking into a sentence by the next speaker

… = pause or drawn out words

[ ] = indicates noise made, e.g. [laugh], [sigh], [pause]

[inaudible segment] = Unclear section of the recording

?Mulenga Clinic?, ?P3? = questionable text or doubt as to what was said or who said it

I: Do you allow me to record this conversation?

P: Yes.

I: [Turns page] Okay. Can you describe to me your thoughts about HIV…what do you understand by HIV?

P: HIV, its a disease.

I: Okay. What, what kind of a disease?

P: [People talking in background] A disease where the body can get infected…

I: Mhm. Okay… What else?

P: [Pause]

I: Okay. How can one become infected with HIV?

P: They can, they can get infected by having unprotected sex.

I: Okay. What else?

P: Uhh, HIV could get transmittied to you if you have a cut…you know?

I: Yes.

P: If you have a cut that you can’t see…

I: Okay.

P: Then you touch someone else’s blood…

I: Mhm.Okay.

P: Then you get infected with HIV.

I: Okay. So can you tell me what places a person is at…is mostly high…likely at risk of getting HIV? Which places do you think people could get HIV from?

P: In taverns.

I: In taverns… Any other places?

P: That’s all…that’s all I can think of.

I: Why in taverns? Could you explain to me why you’re saying in taverns?

P: Because the people there would have drank alcohol and they would be drunk. And when they want to have sex…they would not be thinking straight because they are not in their right mindset.

I: Mhmm…okay. That’s good. So can you tell me about any situation when you felt that you may have been at risk of getting HIV?

P: [Pause]

I: Could you tell me about a situation where you felt you were exposed…maybe to HIV?

P: [Pause] Explain again, I don’t understand.

I: Which situation have you ever been in where you felt like…eish this time, I could have HIV?

P: I haven’t had one.

I: You never had…that situation?

P: Yes.

I: Okay. So maybe, can you tell me about the HIV testing services that are taking place in your area?

P: The people that test people?

I: Yeah.

P: We sometimes find them on the roads. At the libraries…and the clinic.

I: Okay. So where are the testing services…HIV testing services for youth are usually delivered?

P: [Pause]

I: Where do the HIV testing services for youth take place?

P: At…at the libraries. On the streets, the clinic as well as the hospital.

I: Okay. In the streets, who get tested? Which people go and get tested?

P: Most of them are school children.

I: Who tests them?

P: People who come from different units…

I: Okay. So the school children that get tested, how old do you think they are?

P: 15, 16, 17.

I: Okay. Is it a lot of children that get tested?

P: No, it’s a small amount.

I: It’s a small amount… So in your opinion, what is positive about the current HIV testing services that are available to youth? What is important about HIV testing services that are available to the youth?

P: It is helpful to them…especially those that have unprotected sex.

I: Okay. [Turns page] Anything else that you can think of?

P: Thats all I can think of.

I: Okay. And then you told me about the positive aspects, can you tell me the negative aspects…of current HIV testing services that are available to youth? What would you say is negative …about HIV testing services that are available to youth?

P: Cause other people…could commit suicide after finding out that they have HIV.

I: Mhm. Okay. Anything else?

P: That’s all I can think of.

I: Okay. [Turns page] Why would they commit suicide?

P: Because they would be afraid of what other people will think of them. They are still young…but they already have HIV.

I: Mhm. Okay. So how do you think incentives could be used to encourage youth to test for HIV and access treatment?

P: …

I: How do you think incentives can be used to encourage the youth to come and get tested as well as get treatment?

P: Uh…they, they could come to schools and educate the youth.

I: Okay. [Writes down] and then, anything else that you can think of?

P: [Pause] That is all that I can think of.

I: Okay. What do you understand about…the term incentive? According to you what do you think incentive means?

P: It is something that they test you with, to check whether you have HIV. An incentive is something that you are given maybe after testing…like a water bottle, T-shirt…or a CD.

I: Okay. Can you describe the type of incentive that youth value which could encourage them to get tested for HIV or access treatment?

P: CD…

I: CD? What kind of CD? Music CD or…?

P: Yah I think music.

I: Music. What kind of, what type of music?

P: Hip-hop.

I: Hip-hop…and other incentives? You can say as many as you want to.

P: Water bottles for that plays soccer for example.

I: Water bottles… what else?

P: T-shirts.

I: T-shirts. Mhmm…

P: Caps…

I: Caps. So on the T-shirts and the caps, should something be written they should just be plain…like a message of some sort. To inspire and to draw the youth…in order to call them.

P: They should be written; they should be printed on.

I: What should be printed on them? What would you prefer to be printed?

P: They should be printed on something regarding HIV, and testing.

I: Okay. And what other incentives could, uh…the youth value?

P: [Pause] Thats all I can think of. Oh yah…and food.

I: Okay food. What kind of food?

P: Like sandwiches…

I: Okay sandwiches. Anything else?

P: [Pause] Ai, that’s all I can think of. And bags!

I: Bags…schoolbags or?

P: Yeah school bags.

I: [Writes down]

P: And money.

I: [Writes down] Okay out of…all the incentives you spoke about. What are your top 3? Those that you prefer if you got tested?

P: T-shirts…

I: T-shirts.

P: Water bottle.

I: Water bottle…

P: Schoolbag.

I: Schoolbag…[Pause] How often do you think these incentives should be, uh, given for HIV testing services? How often do you think they should be provided? When should we give them to people, during which occasions?

P: When people come in to get tested.

I: Okay. Anything else?

P: Yoh.

I: Anything else that you can think of?

P: Hai. There is nothing else that I can think of.

I: Okay. What could be the challenges of providing these incentives? What could cause challenges maybe? When we are giving out incentives.

P: I don’t understand.

I: Mhm, when we give the youth incentives, when we give out money, water bottles, t-shirts…what could be a challenge there?

P: [Pause] like people could fight.

I: Okay. What would they be fighting for?

P: They will be fighting to get the incentives.

I: Okay. And what could be the benefits?

P: Others could get tested for HIV.

I: Okay. Anything else that you can think of?

P: There is nothing.

I: Okay. Can you maybe describe to me your thoughts about being contacted via telephones, social me-…uhh or social media for HIV testing services? What would you think of us calling you or talking to you through social media about HIV testing services? Do you think it’s the right thing to do?

P: No.

I: Why are you saying no? Can you explain…why you’re saying no?

P: Because other people…will be asking themselves why these people are calling them.

I: Oh okay. And other reason?

P: Ha-he, that’s the only reason.

I: Okay. Can you explain, can you describe some examples of how you have been informed about HIV testing services? How have you been told of HIV testing services before?

P: I’ve never been informed.

I: You have never been told anything about HIV testing services?

P: [Pause]

I: Hmm? Never?

P: Yah.

I: Okay. Then how would you feel if you were to be informed or registered for HIV testing services using cellphone? How would you feel if we called you to talk about HIV or if we told you to register for HIV testing services?

P: It would feel bad because I would not want to get tested.

I: You would feel bad?

P: Mhm.

I: Because you wouldn’t want to test?

P: Mhm.

I: Why…could you maybe explain to me why you don’t want to get tested?

P: Because I would not be focused on other things. Instead I would be only focusing on the fact that I have HIV. It will make me think of things that are out of line.

I: Okay. Okay. And how do you think phones can be used to inform youth about HIV testing services?

P: SMSes, Whatsapp…

I: We should send SMSes and then…what were you saying?

P: Whatsapp.

I: Whatsapp…

P: Facebook.

I: Facebook…

P: Twitter.

I: Mhm…

P: Instagram.

I: Mhm…

P: Yah.

I: So out of these things, do you think…uh youth can access them? Will they be able to read their SMSes and access Facebook and Twitter?

P: Yah.

I: Mhm. How often? How often do you think the youth accesses social media? How long do they use it?

P: They are on social media most of the time…

I: Mhm. Okay. Can you describe the challenges that youth might experience if they cont-…uh, if they are contacted on their cellphones for HIV testing services? What challenges would the youth face if they were contacted regarding HIV testing services?

P: Others would ignore the calls.

I: Okay they could ignore them. What would others do?

P: Others would just hang up in the middle of the call.

I: Others?

P: Yah that’s all I can think of…

I: Uh so…because you have spoken about the challenges, what could be the benefits of being contacted ka cellphone? To tell you, to inform you about HIV testing services…what are the benefits?

P: For those that want to test, it would be helpful receiving those calls.

I: Okay. Anything else?

P: Hyai that’s all.

I: So in your opinion what types of other social media do you think can…can help to inform youth about testing services? You’ve already said Whatsapp, Facebook, Twitter…right?

P: Mhm.

I: What else can you think of?

P: Yoh…magazines.

I: Magazines.

P: Newspapers.

I: Newspapers. Okay. Anything else?

P: [Pause] TV.

I: Okay. Is there anything else that you can think of?

P: Radio.

I: So out of this, do you think uh…the youth would listen to the radio and read the newspaper?

P: Uh, uh, it is not a lot of youth that actually listen to the radio or read the newspaper.

I: Can you maybe explain why the youth does not read this kind of information?

P: Because they spend a lot of time on the internet. Now they are on Facebook and Whatsapp.

I: Oh so, are you saying if we were to publish information on social…on social media like Whatsapp and Facebook…that, that would be where the youth would access it?

P: Yes.

I: Would they read it?

P: Yes.

I: Okay. So if they…they access this Whatsapp, Facebook, Twitter. What are, what are they using to access the internet?

P: Data.

I: Okay. Where do they get it?

P: They buy it.

I: Okay. Can all the youth afford to buy data?

P: Yah.

I: [Writes down] Those that cannot buy data, don’t you think that data could be a part of incentives?

P: Yes, it could be a part of incentives.

I: How would it help them if it were a part of incentives?

P: Because they could log into the internet. And then they could have access to information and be told about HIV testing

I: Okay. That’s good. What are the challenges for using social media to contact youth for HIV testing services? [Turns pages]

P: Challenges?

I: Mhm. [Turns pages]

P: [Pause]

I: Anything that you can think of.

P: [Pause] I can’t think of any challenges that they could come across.

I: So you think it would be okay to contact them via social media?

P: Yes.

I: Okay. How do you think your parents, right?

P: Mhm.

I: Or legal guardians would feel about you receiving information on HIV testing or cellphone…on your cellphone or social media? Personally, how would your parents feel if you received calls on HIV or messages on social media?

P: They would not be happy.

I: They won’t be happy?

P: Mhm.

I: Can you explain why they wouldn’t be happy?

P: Because they would be focused on how young I am to be getting HIV testing done. So I wouldn’t tell them…maybe, I would commit suicide if I find out that I have HIV.

I: Mhm.

P: That’s why they won’t be happy if they find out that I am getting calls about testing.

I: Mhm. So at what age do you think a youth would be ready to go for HIV testing?

P: 19.

I: Why especially 19? Can you explain why especially 19?

P: Because at that point you are not like when you were growing up. You are all grown up and you can think the right way [(Logically)].

I: So can mhm…can you tell me other suggestions that you may have which can encourage youth to get, uh, HIV tested. Which other ways can we encourage the youth to get tested?

P: [Pause]

I: Anything else that you can think of…that could make youth come and get tested? What could we as health workers do?

P: You are asking what you as workers can do to encourage testing?

I: Yes. Anything…

P: Soccer.

I: Soccer?

P: Mhm.

I: Okay. What about soccer? What should we do?

P: Cause there are people who love soccer.

I: So if we had like a tournament, you think people will come? To-

P: - To test. Bash…

I: Okay. Bash, what kind of a bash?

P: The bashes they sometimes have at the clinic. The give people things.

I: Okay.

P: Yah.

I: So those are things the youth would be excited about?

P: Yah.

I: Is there anything you can think of?

P: No.

I: Okay. Uhh, do you have any final thoughts about youth, HIV testing services or incentives? Your final thoughts on youth, how we can improve HIV testing services and what kind of incentives you want to add.

P: The people that want to come for testing…

I: Mhm.

P: The incentives that they could give them are like data…

I: Okay. Data…and what else?

P: Others could be given balls…

I: What kind of balls?

P: Those that you play with…soccer ball-

I: -Soccer ball. Okay. Is that all?

P: Yah.

I: Okay…now, we have come to the end of our discussion. Thank you for your participation. If you have any questions. The form, the informed consent form I gave you has numbers that you can call just as I showed you. You can call between 8 o clock in the morning until 5 in the afternoon. Thank you.

End time: 16:25
