# Supplementary material for: Exploring perceptions of low risk behaviour and drivers to test for HIV among South African youth
Source: PLoS One. 2021 Jan 22;16(1):e0245542. doi: 10.1371/journal.pone.0245542 (PMC7822253; doi:10.1371/journal.pone.0245542)
Supplement: S1 File — (ZIP) [file pone.0245542.s001.zip › S1_File_Anonymised Transcripts/A02-039-NT Translation - Wellington_QC2_TM.docx]

Full Participant ID: A02-039-NT

Participant Type: In-Depth Interview

Location: Daveyton – Chief Albert Luthuli Clinic

Date: 08 October 2018

Start time:

Primary interview language: IsiZulu & English

Name of Facilitator/Interviewer: Wellington Maruma

Name of Note Taker:

Name of Transcriber: Wellington Maruma

Length of recording: 00:34:11

Label Key

I = Interviewer

P = Participant

N = Notetaker

{ } = Indicates that details were changed or pseudonyms were used to anonymise data

xxx = words were omitted to anonymise data

- = breaking into a sentence by the next speaker

… = pause or drawn out words

[ ] = indicates noise made, e.g. [laugh], [sigh], [pause]

[inaudible segment] = Unclear section of the recording

?Mulenga Clinic?, ?P3? = questionable text or doubt as to what was said or who said it

I: Thank you so much for being part of this interview. Do you allow me to record this interview?

P: Yes.

I: Ok thank you.

P: Yes.

I: So uhm… Can you tell me what you think HIV is?

P: HIV?

I: Yes. What is HIV?

P: Uh HIV eh maybe you see…It’s just that I don’t understand. You mean what is HIV?

I: Yes. Tell me what you think HIV is.

P: What is it?

I: Yes.

P: Maybe HIV is a disease.

I: What kind of disease is it?

P: It’s a disease. Maybe if you sleep with someone without using a condom. Yes, that is how a person can get infected with it or maybe when someone is helping someone who has HIV, let’s say maybe the person has an open wound and the blood of the other person with HIV gets in touch with that person`s blood, that’s how a person can also get it.

I: Okay and can you tell me about places where you think a person can get HIV? You’ve mentioned through unprotected sex and through bleeding. Is there any other way you know how a person can get infected with HIV?

P: HIV?

I: Yes. How can one get infected with HIV? Is there any… is this the only way you know how?

P: Eh. (Yes.)

I: Okay. So ummh can you tell me if there was ever a situation…Has there ever been a time where you felt you where exposed to HIV?

P: No.

I: Never?

P: There isn’t.

I: There isn’t?

P: Yes.

I: So you’ve always been…safe?

P: Yes.

I: Why are you saying so? What makes you think that you have always been safe?

P: No, because I do not sleep with girls. I am still very young… Yes.

I: Okay and what else?

[Silence]

I: You’ve never touched blood?

P: No. If ever you touch someone`s blood, you must wear gloves yes… to protect yourself.

[Noise in the background]

I: Yeah?

P: And when you help someone with HIV, you must first check yourself first you see? Things like that. You must have those plastic gloves, not those ones you wear when it is cold to warm yourself. And when you are done with them, you put them in a dustbin so that small children cannot play with them.

I: So where can people access HIV testing services – where do you know where a person can get tested for HIV? This clinic or where else do you know where HIV tests can be done?

P: Even in {XXX} (Name of clinic).

I: {XXX} (Name of clinic)?

P: Yes.

I: Where in {XXX} (Name of clinic)?

P: By the circle… at {XXX} (Name of clinic) mall.

I: Where else?

P: And at {XXX} (Name of clinic). They also do test at {XXX} (Name of clinic). Even at this clinic- at this very clinic.

I: And then you, where would you…you haven’t tested for HIV before right?

P: Eh. (Yes.)

I: So where would you want to get tested?

P: Right here at this clinic because it is closer. Other clinics are way too far.

I: Mhmm.

P: I would want to get tested right here at this clinic.

I: Why this specific clinic? Is it only because it’s closer or is there any other reason?

P: Because you see, it gets very full at other clinics. At this one, it does not get that full. Now if you are going to these other clinics, it means you will have to wake up very early.

I: Where? At…

P: {XXX} (Name of clinic) [main clinic].

I: It gets full there?

P: Yes. It gets very full at {XXX} (Name of clinic) [main clinic].

I: And is that also a reason you would not want to go and get tested maybe because it is always full?

P: No not that only. Also, if you do not have money, you are still able to come to the closest clinic to come and get checked out.

I: Okay.

P: Because a lot of people are unemployed here you see? You must use an avanza (taxi) if you have to go there.

I: Mhmm. Okay. And as for testing here at the clinic, is there any other reason you would not prefer getting tested at {XXX} (Name of clinic) [main clinic]?

P: No. I also want to get tested for other diseases, not only for HIV.

I: What else?

P: For TB as well. I also want to get tested for TB as well to see if I don’t have TB as well. I want to get for both of them (HIV & TB).

I: Okay yes. I am saying that isn’t you said that you would want to get tested here at this clinic…

P: Yes.

I: What is the reason you would want to get tested right here at this clinic compared to {XXX} (Name of clinic)mall or?

P: This clinic is fine because they attend to their patients very quickly and there, they take a lot of time to attend to their clinics. You can leave early in the morning and come back very late in the night.

I: {XXX} (Name of clinic) [main clinic]?

P: Yes.

I: (Okay. And is there any other good thing about getting tested here at this clinic or do you think you can also get tested somewhere else?

P: No. It is fine here at the clinic.

I: Why?

P: No because there, they don’t pay attention to you. They take time to pay attention to you. They usually start with the grannies or the elderly people. Now when you come and you stand in the queue, they take from that queue one by one…just like that. Now you find that the queue is so long that it starts from the gate, it gets very full there.

I: Mhmm.

P: Yes.

I: {XXX} (Name of clinic)?

P: Eh, {XXX} (Name of clinic)

I: And then here it does not get very full?

P: Here it does not get too full. The only section of the clinic that gets too full is where there is a lot of children and mothers. But if you are sick with anything else, that section does not get full.

I: Mhmm.

P: Yes.

I: And then besides it being too full at the clinic, is that the only bad thing about getting tested [for HIV] at the clinic or is there any other bad thing that you have heard or maybe have experienced?

P: No.

I: Is it the issue of being too full only?

P: The clinics being too full only. Even here, some people wake up very early. They wake up around 05:00 in the morning. Some stand in the queue up until they open the clinic at 06:00 waiting for them to open.

I: Mmmh.

P: Yes.

I: Okay. So uhm [Clears throat]. When you think of the word “incentive”, what are you thinking?

P: I think that… isn’t we sometimes come to the {XXX} (Name of organization)sessions, they tell us about HIV you see? Sometimes they tell us to come to the clinic so that they can give us something you see. They give us bottles and stationery for school… Yes.

I: And what else?... So for you to get tested for HIV, what else would you want to be given because you did mention that {XXX} (Name of organization)was giving you guys’ bottles and stationery…?

P: Yes.

I: What else? So for you to get tested for HIV, what else would you want to be given because you mentioned that {XXX} (Name of organization)gives you gives you guys bottles, stationery… what else? So for you personally, what is it that you want to be given to get tested?

P: A t-shirt and a cap.

I: Mhmm, why?

P: Because I want to show people that I am attending here… Yes, so that they may also want to come as well… Yes.

I: Mhmm. And what else?

[Long silence]

P: And as well… so that when we have arrived here, they can see who is attending. The ones that attend a lot of the sessions can show others that here are the shirts that we got… they can then wear their own clothes later.

I: Mhmm. And then what do you think of maybe uhmm…food?

P: Food?

I: Mhmm. Is that something that would encourage you to go and get tested? Like let’s say they were saying that they would give you food after getting tested. Is that something that would encourage you to go and get tested?

P: Yes.

I: Why?

P: Because you see… sometimes I come here very early without having eaten and if they can give us something to eat.

I: Mhmm, what kind of food?

P: Food – bread and soup.

I: Mhmm.

P: Yes. Even the ones that come very early in the morning, they are given bread and soup – the old grannies that arrive very early here at the clinic. Some of them arrive as early as 5 o`clock in the morning, and they are given soup and bread here at the clinic.

I: Okay and why do you think it is important that they give you food to encourage you to go and get tested for HIV? [Sound of paper being flipped].

P: You see there are other things that have chemicals that are strong so one needs power in their body, you see so that they are not weak in the body yes.

I: Ummh.

P: (And also again older people like the grandmothers they must take pills after eating yes.

I: Ummh, so you think maybe the reason some people do not get tested is like maybe in your age group, is it maybe because they are not given these things at the clinic?

P: Because they are still so young you see yes.

I: Ummh.

P: They can, like this clinic they do not want small children because there are other diseases here at the clinic, eish…I don’t know eish.

I: What kind of diseases?

P: Maybe you find that there are people that are really sick like they are coughing or they have TB so now they do not want small children here at the clinic.

I: Ummh.

P: Those people that are really must be put together on one side.)

I: Ummh.

P: But they do say here at the clinic that a person of a certain age must not enter you know things like that.

I: (Oh! So they say that kids of a certain age must not come to the clinic because they will get certain diseases?

P: They don’t say that.

I: They, ok.

P: Maybe you see there are rooms where they say a child of a certain age should not enter only maybe people with TB enter it, you see things like that.

I: Ummh , so you are saying maybe that is the reason some people don’t come to the clinic?

P: Ummh eish [long silence].

I: Why do you think that people your age do not come to the clinic to test?

P: [Long silence] why people in my age group do not come to test here at the clinic?

I: Yea, why don’t they come?

P: Because they don’t have an ID they are still young.

I: You do not need an ID so that you test.

P: [Long silence] eish I don’t know.

I: Ummh ok, why haven’t you come to test? Why haven’t you come to the clinic because you said compared to {XXX} (Name of place)you would rather test at the clinic?

P: Why haven’t I tested?

I: Ummh.

P: Because in some places you only start getting tested for things at 16 years old.

I: Ok.

P: Yes.

I: [ [Shuffling of papers] Ok so and then food, stationery, t-shirt, caps and food is the only things you would want so that you come or there is something else that you can maybe think of?

P: Ummh and also a phone.

I: Ummh, and what else?

P: [Long silence] I want a phone so that when my parent wants to call me maybe I am on the street they can call me.

I: Ok.

P: Yes.

I: So you think if people give you a cellphone they will be encouraging you to come and test like come to the clinic to test?

P: [Inaudible 00:11:46].

I: [Giggles], Ok what would make you come to test?

P: Ummh because sometimes I see that some people are really sick out here and it’s because they do not want to come to the clinic to get tested and know whether they are sick or not.

I: Ummh.

P: Yes. Other people are scared to come to the clinic to test.

I: Why are they scared?

P: I don’t know

I: Are you also scared of coming?

P: I am not scared.

I: Why do you think other people are scared?

P: Maybe you see maybe one has HIV this thing will be troubling them and will be always thinking of their status.

I: Ummh.

P: Yes.

I: What do you think we can do so that people are not scared to come to the clinic?

P: [Long silence], what we can do?

I: Ummh.

P: Ummh.

I: In your own opinion.

P: [Long silence], ummh…In my opinion I can tell them that there is nothing scary about coming to the clinic to test, yes.

I: Ummh, but why do you think that they are scared of coming to test at the clinic?

P: It is because other people over think things and end up having heart failure because they found that they are HIV positive, yes.

I: Ummh, ok. Ok let’s say you are saying that if we were to give you a cell phone you still wouldn’t come to test?

P: No I will come.

I: And then what about bottles, stationery the ones you have already mentioned.

P: Bottles?

I: Ummh.

P: Also it will help as I will not need to leave the line to go look for water as I might end up losing my spot. Ummh, eish I also want them because I can use then as containers to carry water.

I: Ummh.

P: Yes.

I: Ok, so you are saying if we don’t give you any of this you would not be encouraged to come and test?

P:Ummh I would come.

I: Even if we don’t give you these things?

P: Yes. Not that one must be given something in order to test because this is their life.

I: So why do you think other people want to be given something in order to come and test?

P: The problem is that people here love free things, so you see because of that they will come. If you tell them there are free things here at the clinic they will come.

I: Free things like food?

P: Yes like people love food.

I: [Giggles], you think that food will also be something that would maybe encourage someone your age to come to the clinic to come and test?

P: Yes

I: Ok, what kind of food?

P: Food like bread and soup and juice.

I: And juice?

P: Yea.

I: Ok and nothing else? So you have mentioned bottles, stationery. So what would consist of stationery? What kind of stationery?

P: Maybe school things like pens, glue and pencils.

I: Ummh.

P: Yea.

I: Only?

P: And crayons.

I: Ummh ok. Ok so you were saying that people will only come to the clinic if there is food and all those types of things right?

P: Yes

I: So you are saying that if we don’t give them food people will not come?

P: They won’t come and also some people only come to the clinic only when they are sick, if they are not sick they do not come to the clinic.

I: Ummh.

P: They only come to the clinic when they have a rash or they have a certain illness then they come to the clinic.

I: Ummh.

P: Yes.

I: So those are one of the challenges of people not coming to the clinic?

P: Yes.

I: Or giving people these things?

P: It is all the same here at the corner they give food and it gets so full on Tuesdays because they give food. Lots of people come I also go and {XXX} (Name of a person) also goes there

I: Ummh.

P: Yea.

I: Ok, and what do they do there? Do they test or?

P: They give food.

I: They only give food there; they do not test for HIV?

P: They don’t test people there.

I: Ok and how often does that happen?

P: Excuse me?

I: How often does it happen like how many times a week do they come?

P: Eish I don’t know yho!

I: Ummh.

P: Everyday on Tuesdays.

I: Ok and what do you think is the good thing with giving people these things? What is the good thing about them?

P: Mmmh?

I: What is the good thing about giving people these?

P: The thing is that if you give someone something they are more encouraged to come, if they don’t people will start saying that these ones are stingy. Just like here at the clinic they sometimes give people bread and when they stopped giving it people stopped coming to the clinic. Some people pretended to be sick just to get food here at the clinic.

I: Ummmh.

P: Yes.

I: So you are saying people only might only come to the clinic just to get food?

P: Yes and leave after that.

I: And they end up not testing?

P: Yes.

I: Ok. And what else do you think will be a problem with giving people these things?

P: These?

I: Ummh.

P: Other people do not come here at the clinic compared to {XXX} (Name of organization)because there they give bread.

I: Oh ok so just because they are not giving you guys any more, they do not like they are no longer coming?

P: Yes.

I: Oh ok that’s good. Ok ummh and so you said you do not have a phone?

P: Yes.

I: Do you have a phone or not?

P: I don’t have it.

I: Ok, but then let’s say you did have a phone, how do you think we could use the phone to connect with the youth?

P: Yes.

I: For information maybe about HIV.

P: [Long silence] If I had a phone?

I: Yes, how would we use it to give you information?

P: Using whatsapp.

I: Ummh.

P: Yes.

I: And what else?

P: Or facebook.

I: How do we use it? How do we use facebook, how do we use whatsapp?

P: You see you can give information on whatsapp.

I: What kind of information?

P: Explain to me; give me an example like an example of the type of information you would want to read on facebook.

I: Like maybe share with people how one can contract HIV, like having unprotected sex things like that. A person with HIV must always use a condom.

P: Ummh.

I: On facebook?

P: Yes.

I: And then whatsapp how would we use it?

P: How?

I: Ummh.

P: [Long silence]

I: Mmmh?

P: Whatsapp how we can use it? Uumh [long silence]

I: Mmmh?

P: Eish! [Long silence].

I: And facebook you are saying that people just sharing health information?

P: Yes.

I: About HIV?

P: Yes.

I: Ok.

P: Or on whatsapp you can take pictures of someone with HIV and show them how a person with HIV looks like and they must go test.

I: Ummh.

P: Yes.

I: And do you think that would encourage people to come and get tested?

P: Yes, when you show them a picture of someone with HIV they will become scared and come and test.

I: Ummh.

P: Yes.

I: Ok and then ummh if not facebook or whatsapp what else can we use?

P: Ummh, twitter.

I: How would we use it? How would we use twitter to get information out there?

P: How would we use it?

I: Ummh.

P: Ummh, you can download it and check how people with HIV like how they are and that will be how you get information.

I: Ummh.

P: Yes.

I: Ok and let’s say maybe you did have a phone. What do you think the challenges with that would be? So what is the bad thing like challenges that you could face maybe about a phone?

P: Ummh, you can tell people to come and test cause there is still time.

I: I am saying the challenges, like the challenges you could face with having a phone. Like what do you think they are? Isnt there is people who don’t have a phone like you.

P: Yes.

I: So that is a challenge, one of the challenges that’s a problem. So I am asking what other challenges do you think there are?

P: Another challenge will be I would tell parents to buy their kids phones because on facebook one can get important information about HIV.

I: Ummh, so parents must buy their kids phones?

P: Yes.

I: So how do you think your parents will feel when they hear that you are reading about HIV/AIDS?

P: They will be ok because it’s a good thing. Because someone with HIV can die at any time so they need to get treatment.

I: Ummmh.

P: Yes.

I: Why do you think other parents do not want their children reading about HIV on their phones?

P: It is because other kids like to watch things that are not appropriate for their age so parents end up not buying them phones. Or parents do not have the money to buy their kids phones.

I: Ummh.

P: Yes.

I: But why do you think that let’s say you had a phone.

P: Yes.

I: And you are reading information about HIV on your phone right. Why do you think some parents do not want that?

P: [Long Silence] other parents they do not want because they have not tested before at the clinic so they do not want to hear about HIV in their lives. Maybe they also might have it and don’t want to know about HIV.

I: Ummh.

P: Yes.

I: Why do you think they do not want their kids to know about HIV? Does it scare them or?

P: Yea people are scared of it.

I: What are they scared of?

P: Maybe they find that someone has HIV and they do not want to come close to them, things like that you see.

I: Ummh, but you are saying that your parent would be interested in that?

P: Yes.

I: Why?

P: Why? Because my older brother sometimes speaks of these HIV things you see. My mother doesn’t seem stressed about that.

I: Ummh, so you are saying that she will be fine with you reading these things.

P: No she will fine.

I: Ok and then how do you feel like let’s say ummh here at the clinic they call you to come and test, how would you feel?

P: At first I will be scared.

I: Ok. What will you are scared of?

P: Eeei eish, when people say come and test there is a prick that they use and they say it’s painful. So that is what will be scaring me.

I: Ummh.

P: Yes.

I: You are scared of that thing only?

P: Yes.

I: Ok and how do you think you can overcome that fear?

P: Ummh eish.

I: What should we do so that you are not scared?

P: I- I.

I: So that you also know your status.

P: I- I would ask my mother to accompany me to the clinic.

I: Ok, to escort you to come testing?

P: Yes.

I: Ok, and so what about data?

P: Data?

I: Yea.

P: [Long silence]

I: Is that something that you would want? I know that you do not have a phone but do you think it could be something that can be used to encourage people to come and test? Maybe we give them data or airtime?

P: Yea they can give it, you see you can go around telling people that here at the clinic they give airtime after you test. Everyone will want to come and test.

I: Ummh.

P: At the clinic?

I: Ok.

P: Yes.

I: Ok, like how much data do you think will be enough?

P: R30 is ok

I: It’s ok?

P: Yes.

I: And do you think if you had a phone would it work for you to come to the clinic?

P: Yes.

I: Ok and why do you think this does not work on other people?

P: How do you mean?

I: Like let’s say maybe someone else prefers something else data. Why do you think they would prefer something else not data?

P: T-shirts and stationery there are people who are of my age who would appreciate things like stationery.

I: Ummh.

P: Yes crayons, school things.

I: Ummh.

P: Yes.

I: Ok, so you are saying that school things are more important for the youth or?

P: Yes.

I: I see.

P: Because other parents do not have the money to buy these things.

I: Ok, and then what about let’s say for example you are saying school is very important right?

P: Yes.

I: So what about let’s say they will pay your fees in some sort of bursary or?

P: They will pay for us?

I: Ummh. So that you come and test, is that something that would work for you, do you think it would work?

P: Yea, it could also work.

I: Why?

P: [Long silence] because people spend a lot of money on fees, and others cannot afford it so bursaries will help.

I: Ummh.

P: [Silence]

I: Yea.

P: Other parents they only money they get is grant money for buying household things, things like that.

I: Yea. Ok so why do you think are the other reasons people get tested for HIV at the clinic, like come to the clinic to test for HIV.

P: Because people hear through whatsapp that someone died of HIV and at schools people my age are taught about it.

I: Ok, so you are saying that we can only use on the social media you mentioned I facebook, whatsapp and twitter only.

P: Yes.

I: Is there any other social media that you can think of that we can use?

P: No.

I: So how would you want to receive HIV testing information? Since you don’t have a phone is there another way you can get?

P: I would come here to the clinic there are pamphlets written about HIV. Like what is HIV, how does a person with HIV look and how do we treat people with HIV.

I: Ummh.

P: Things like that.

I: Yea.

P: There are posters.

I: Posters?

P: Like they say what HIV does to a person like show a picture of someone with HIV?

I: Ummh.

P: Yes.

I: Ok, so you are saying something like that would encourage people to come and test?

P: Yes.

I: Ok, do you have any suggestions on how we can get people like you to come to the clinic. Any thoughts on what we can do to encourage people your age to come and test?

P: Ummh no.

I: You mentioned food that maybe we give them food.

P: Yes.

I: And what else?

P: [Long silence] You see there are these loud speakers and we announce that we want to test kids this age. You tell them bring the kids to come and test.

I: Like speakers?

P: There is this thing like a loudspeaker.

I: Ummh.

P: Yes, they will use it to speak, like they will call a community meeting by the ground and tell them that there are testing services offered at the clinic come and test and this speaker will go all around the community...

I: So we can call a community meeting and explain to parents that there are testing services offered at the clinic

P: Yes, that way. That is another thing that can help.

I: And then what else?

P: [Long silence] or give pamphlets and people at the clinic distribute them by the gate and say in the pamphlet that people must come and test on such a date and everyone must know about it.

I: Ummh ok. Is there anything; are there any other suggestions you might have?

P: Nothing

I: Nothing?

P: Yes.

I: Ok, so what about a community meeting right?

P: Yes.

I: So what about they do an event in the community and then they ask you guys to come over and then there is someone there that is famous and the person speaks to you guys.

P: That’s also fine, because in other places they do that. The SGB will be the ones talking in the meeting.

I: Ummh, what kind of things do they talk about?

P: They say to us and warm us of thieves in the area who steal; they speak of things like that.

I: Ummh.

P: There are also police officers who are told to patrol the streets at night.

I: Ummh.

P: Yes. Yes.

I: So you are saying that this platform of community meetings where there are SGB members will work?

P: Yes.

I: Ok.

P: Yea they hold a meeting here at the school hall. There is a hall where they hold meetings and people are told the time they can come to the school for a meeting.

I: Ummh.

P: Parents come and they discuss then go their separate ways.

I: Is it just a meeting or there is some sort of event that happens?

P: No there’s is nothing else.

I: It’s only a meeting?

P: Yea it’s a meeting.

I: Oh ok. Ok and then also I mentioned someone famous coming to talk to you guys about testing or something like that right?

P: Yes.

I: You said that would work, so who do you think would work for that type of thing?

P: I don’t know.

I: Mhhm?

P: [Long silence]

I: So you are not thinking of anyone right now?

P: No.

I: Ok, so is there any final thought? We are almost at the end.

P: Yes.

I: Do you have any final thoughts?

P: No.

I: Anything you want to add?

P: [Long silence]

I: Mhhm?

P: No I have nothing to add.

I: So you mentioned that a lot of people come to this clinic compared to {XXX} (Name of clinic)right?

P: Yes.

I: Because uumh in {XXX} (Name of clinic) it gets really full and all those type of things.

P: Yes.

I: So how do you think we can do to ensure that clinics are not too full? And people are able to come to the clinic to test.

P: Ummh, you see you can build another clinic. There is only one clinic here and we get people coming from 12 there by the shacks to this clinic. And other people also leave {XXX} (Name of clinic) to come here because it gets so full.

I: Mmmh.

P: Yes.

I: Ok and then what else?

P: [Long silence]

I: What else do you want to add? Anything you want to add?

P: [Silence]

I: Before we finish

P: It’s not there.

I: Ok so you are saying we give you bottles, stationery, t-shirts and caps, food and cell phone.

P: Yes.

I: That’s something that would encourage people to come to the clinic?

P: Yes.

I: And then what if these things are not there?

P: [Silence]

I: Do you think people will still be coming or?

P: No, the problem is that people are not the same so some might still come if we do not give them these things while others might not.

I: Ummh, ok anything you want to add we are almost at the end of our interview anything else you want to add?

P: [Silence]

I: Mhhm?

P: No it’s not there.

I: Ok we have come to the end of our discussion. Thank you so much for participating, ummh the time is 15: 09. Thanks.

P: Yes

End Time: 15:09
