# Supplementary material for: Exploring perceptions of low risk behaviour and drivers to test for HIV among South African youth
Source: PLoS One. 2021 Jan 22;16(1):e0245542. doi: 10.1371/journal.pone.0245542 (PMC7822253; doi:10.1371/journal.pone.0245542)
Supplement: S1 File — (ZIP) [file pone.0245542.s001.zip › S1_File_Anonymised Transcripts/A02-040-GB_08102018.TRANSLATION_QC2_TM.docx]

Full Participant ID: A02-040-GB

Participant Type: 16 Male

Location: Chief Albert Luthuli clinic Daveyton

Date: 08 October 2018

Start time: 15:21

Primary interview language: IsiZulu/English

Name of Facilitator/Interviewer: Wellington Maruma

Name of Note Taker: N/A

Name of Transcriber: Siphelele Khoza

Length of recording: 28:32

I: Thank you so much for being part of this interview, do you allow me to record this uhm interview?

P: Yes

I: Speak louder.

P: Yes!

I: Okay. Thank you, so [tongue clicking] can you tell me what you think like give me your, your uhm idea or description of what you think HIV is. What is HIV?

P: Like HIV, like it’s a virus like in the body that slowly eats you inside.

I: Uhum

P: Yes, that’s what I think HIV is.

I: And then, how do you get it? What?

P: Eh! HIV like is transmitted when like, like DNAs clash together yes.

I: Uhum.

P: Yes!

I: And?

P: And? Rain? It enters into your body and fights your, your immune system [shouting outside].

I: Yes!

P: Yes.

I: So like you said DNAs clashes, how does it enter in…in your body?

P: Like, through blood, yes like that.

I: Uhum, and how else?

P: Yes, and also if you have sex so, yes.

I: Uhum, yes!

P: Yes, that’s what I think is HIV.

I: uhm okay, and then so uhm you said? Before? Like uh blood, like in what way? Ah how? [a bit of interruption between I and P] Is that only with sex or maybe there is other way you can get infected?

P: Like, ma…like maybe when I’m cut maybe, maybe you help me you see. Like that, maybe our bloods mix so. Yes!

I: Uhm okay. And then is there ever? Was there ever a situation where you felt that you yourself you are at risk of getting HIV?

P: Ay No!

I: Never!

P: Eh!

I: Okay. Never? Are you saying never?

P: Never.

I: And why do you think you are not at risk? So do you think you are at risk of HIV?

P: No!

I: Why not?

P: No because I don’t like mixing myself with the crowds.

I: Uhum.

P: I like to be individual and, yes that’s why I think I’m not at risk.

I: Yes, because you wh… you like to be alone.

P: Yes.

I: Ohkay! And so testing for HIV right! So where do you know where you can go test? Is there any place where you can test for HIV?

P: Ah, its only here at clinic.

I: Only?

P: Yes

I: Ah no! Anywhere else?

P: No.

I: It’’s okay. So if you were ever to get tested for HIV you would want to get tested here at the clinic? Maybe in the future or when you want to get to know your status.

P: Yes.

I: Or is there, is there any other place where you can test?

P: No just here at the clinic.

I: Why here at the clinic?

P: It is because no one will judge me here because they understand these things of checking.

I: Uhum.

P: Yes, they know better. They will give me advise also, so I think the clinic is best.

I: Mmh. So it’s because they won’t judge you?

P: Yes.

I: Okay. And then do you think other clinics are judgmental?

P: No! I didn’t say it like that.

I: Oh, okay.

P: I said like a clinic, any clinic. Yes, I would be better if you go to the clinic because if you go to the clinic they won’t tell people that you have? AIDS? Or something.

I: Okay. And then why do you think people like your own age don’t come to test at the clinic? If.

P: Yes, people are like shy, so. Other say they know but they don’t.

I: Why are they shy? Ashamed?

P: Others already know they have HIV, but others are not shy. There are people already who take ARVs, yes.

I: And then why like…is it because people think that other people know that they have HIV that’s why they are ashamed?

P: Yes, like a person does not want to be known and what people will say about them, yes.

I: And then how? Like how do you think these guys you can overcome that?

P: Like, maybe like some sort like have tents in like open spaces you see. And then… like you’ll know they’ll come to test and Yes. Everything done will remain in the test, a person will leave alright.

I: Uhm

P: You see.

I: So uhm you think that like if we can have tents and call people they will come?

P: Eh, some like a few can come through.

I: Uhm, why…why do think other people will not come, they won’t come?

P: Because others already they will…they will like, it’s just that a person will say others will see me on the testing sports and say I have AIDS, but no…not like that. There are those who say I don’t care what others say about me.

I: Uhm.

P: I’ll go there when I want to. so.

I: Uhm okay. And then, so you think at your clinic they are not judgemental and or any other things right?

P: No.

I: So that’s one of the positive things for coming to this clinic right, from here? Is there any other negative things that…maybe things that you’ve heard about the clinic this one about HIV testing?

P: No…no.

I: Nothing. Any other clinic?

P: Aah ah.

I: Okay, any other clinic?

P: No!

I: So, you’re thinking clinics are right for HIV testing?

P: Yes, I think they are better.

I: Compared to testing in tents. So compared to tents and clinics where would you want to get tested?

P: Eh…the clinic.

I: At the clinic. Why not in the tent?

P: Eh, when I come to the clinic you see there are many reasons that can make me to come to the clinic. So they won’t say you’ve come to test or so. They see I don’t like people talking about me, I don’t want to a topic...

I: Oh so you saying at the clinic like there are a lot of reasons that you can come to the clinic. So people won’t know why did you come, you came to test...

P: Yes.

I: But in the tent they will know that you-

P: Yes, they will see since it will be written outside. They’ll see that likewise you came to test when you come out finding them outside asking you what is your status, you see pushing you to the corner.

I: So why do you think people are scared of testing? Like maybe your age group. Why do you think that they are scared?

P: Others maybe they know that they have it. Others they know they don’t have it.

I: Okay. So for those who don’t know why they don’t come?

P: Those that know they don’t have it?

I: Uhum.

P: Like-

I: Like how do they know if they did not test?

P: What?

I: How do they know?

P: Like one would say they know their ways they have not slept with anyone. Yes so; they would say that maybe because their mothers don’t have it that means they also don’t because others say, you get it from your mother.

I: Hmm.

P: Yes.

I: So what about…cause like you need to say oh- others get HIV through blood right- So what about maybe when they’re helping another person not sleeping with them, but they were helping another person with blood and then he got infected or something. Don’t you think they’ll want to know?

P: Yes! That one can go to the clinic to check or to the tent.

I: Okay, but why do you think this one doesn’t go, cause I mean, why do you think they don’t go?

P: Because others were not aware that their blood, like so, others like just say it was a small thing only I wiped it, but no.

I: Mmh okay. So…is there any-so the only negative thing about…I mean the only positive thing that you’ve mentioned about testing at the clinic is that there they don’t judge right?

P: Yes.

I: So what are the positive things do you think?

P: Like they give you advice so yes they show you how to do things. They help you so, yes.

I: Uhum, and what else?

P: Like here already…already its safe like already. It’s like a place that was made for us to feel safe that when we have complains they can show us what we can do.

I: Uhm. Okay, and then any negative? So you said there is nothing negative about the clinic?

P: Eh, yes-

I: Or there is?

P: Eh, no like for HIV I would say there is nothing.

I: Uhm, and for other services?

P: Like I would hear others say here at the clinic they tend to be slow so.

I: uhm.

P: Yes.

I: Being slow right-

P: Yes.

I: So the lines are always full.

P: Yes.

I: Uhm okay.

P: And then like old people would stay for long at the clinic.

I: Uhm.

P: Yes they say so.

I: Uhm okay. And then, how do you think- so do you have any suggestions that you have maybe that we can do away with this negativity; testing, and slow clinics? Are there any suggestions that you have? Is there is anything we can do so that the clinic can be more efficient maybe?

P: Yes maybe.

I: What can we do? Any suggestion?

P: Yes maybe like encourage the like the staff you see, yes.

I: Umhuh.

P: Like show them that people stand in line for too long, yes. Another negativity they say like people like here don’t get pills [10:52].

I: What?

P: Like…like have you seen when a child is sick and they say they will go get pills, so yes like maybe say they will give them panado.

I: Ooh!

P: Yes only, they say they don’t have other pills.

I: Ooh, okay, so like the medication sometimes is not available?

P: Yes.

I: Ohkay. So when you think of ahh- so you, for you you’ve never tested right so let’s say uhm…what would encourage you to get tested?

P: Me?

I: Yah, cause…like something we can give you so that you can test.

P: Yes I can go to test I just haven’t thought about it.

I: Mmh.

P: Yes.

I: But what could make you to test or someone maybe your age? What is it that can encourage you to come to the clinic?

P: Yes, maybe like see like, like in our friends group someone like become sick. Us also we would want to see why this person is sick, what does he have didn’t he infect us? Maybe ay he must first go check AIDS maybe you see so.

I: Okay, and then like do you know that other HIV testing services give away maybe caps right for testing, shirt or anything else? Is there-shirts and? T-shirts? Is that something that could encourage you too to come and test or…will it work?

P: Ah, ey for me no, shirts no.

I: what would you want?

P: What?

I: What would you want? Cause you say no shirts right?

P: Yes, maybe bangles and a cap can be alright.

I: Only or is there any other thing?

P: No, those are fine.

I: Bangle!

P: Yes, bangle and hat-cap yes.

I: Is that just for boys only or girls have to get something different?

P: Girls? Yes, girls like bangles so, maybe a t-shirt so.

I: T-shirt?

P: Yes.

I: Okay, what kind?

P: A t-shirt, any type like a t-shirt for girls.

I: Mmh, what do you think of ahh maybe give out food after- after you have finished testing? Is that something that could encourage you personally?

P: Yes, many people will come because there is food.

I: What kind of food?

P: What?

I: What kind of food?

P: Especially if it has meat they will come for sure. Maybe bread, meat, and chakalaka they’ll come.

I: Uhm, so food, t-shirts, cap and bangle.

P: Yes.

I: anything else?

P: A bottle maybe.

I: Uhum.

P: Yes.

I: Anything else?

P: No.

I: Is that all?

P: Yes.

I: Okay. So out of these things you’ve mentioned right, a bangle, a cap, a t-shirt, food and bottles. Which are your top 3 things that you- you think are important for you?

P: Like?

I: Like things, let’s say I’m saying I’m only gonna give you 3 things to have after you have finished testing, I’ll give you only 3 things. What are those 3 things here? Let’s start with number 1, number 2, number 3.

P: Yah, maybe a cap, food and a bangle maybe.

I: So these three things are important for?

P: Yes, they are the ones I would choose.

I: Mmh, okay and then why these three things and not a t-shirt and a bottle?

P: No! there is a bottle you see at home so, there is food at home but like its different. A cap, I’m not a person who likes buying caps so if I can get it here that would right.

I: Mmh, a normal cap or a cap with something?

P: Yes, like, maybe it must have maybe like those things like their own brands so.

I: Mmh.

P: Yah, be written the company name.

I: Mmh, okay. So uhm you have a phone right?

P: Yes.

I: So how do you think we can use the phone? That maybe we, maybe we- like give out information about HIV testing services. How do you think we can use the phone to get that information to- to you or to someone of your age group?

P: Like messages maybe?

I: How can we use sms- you saying messages?

P: Yes.

I: How?

P: Eh maybe…maybe like, maybe you can use maybe WhatsApp groups so.

I: Uhum

P: Yes.

I: ?No?

P: Yes and Facebook maybe.

I: And then, oh like Facebook how? Maybe just take me through that.

P: Like maybe you post things about AIDS you see people like can see them and even make comments, likes the post you see. Maybe others can ask more details so yes.

I: Mmh and then what about the WhatsApp group?

P: WhatsApp group maybe like, just like those who use it put them in a WhatsApp group. Like maybe use it for asking questions you see and we all teach each other what is HIV.

I: Mmh.

P: Yes.

I: Okay. And then what about let’s say when we say the clinic right must call you for example to say you must come to the clinic to test for HIV, would you be happy about that or you won’t?

P: Ah…I can come to test.

I: Yes.

P: That would alright.

I: Okay. And then is there any other thing that you may probably want to add, eh on these things that you’ve added maybe bangle, cap, t-shirts, food, bottles?

P: Ah…

I: What about data?

P: Data?

I: Uhm.

P: Eish! Data is something most people want.

I: Yah-, will it work?

P: Yes it will work because other’s data will be finished like maybe in the middle of the month so if there are things like that maybe a person can save money ? And left with?

I: Uhm what about for someone those at school, stationery?

P: Stationery!

I: Uhum! Do you think that it will work?

P: No, it’s just that in schools they give out stationery.

I: Mmh-

P: [inaudible 18:21]

I: [inaudible 18:25] so it won’t work?

P: Like maybe stationery like calculator so…yes things like that.

I: Mmh, okay. And then let’s say...you said that on Facebook you can post information about HIV about everything else right. How do you think your mother would feel about you receiving HIV information on Facebook?

P: Eh ah, like my mother like she won’t say like maybe I have HIV or something you see because she will ask like what is this for. Like I will tell her I’m learning like, she will say that as long as this thing propels you to move forward with life and not dragging to back it’s a right thing.

I: Mmh, okay. And then why do you think other parents don’t want for their children to get information through like from of social media about HIV?

P: Like others think like, that social media has bad reviews. Like it teaches them bad things so because most of them are always on their phones you see. So they think this child is not concentrating at school so then decide not to allow them to be on the internet, even take their phone you see, like that. Others maybe say they want their child to be focused you see.

I: Mmh.

P: Yes.

I: So like what do you think we can do so that parents can be more supportive like maybe and for their children to be able to learn about HIV wherever?

P: Like maybe like show them that this thing teaches about HIV that children should just not use condoms so, yes.

I: Mmh, okay. So using WhatsApp groups and other social media like Facebook has benefits. Do you know of any benefits that you can maybe share with me?

P: Like?

I: You were saying [stretching noise]

P: Like? What was the question again?

I: Ah, why do you think other parents don’t like their to receive information on Facebook?

P: Like maybe others see that their children will see things done by celebrities and other may copy them so you see. Like others they just think about things like that.

I: Mmh.

P: You’ll find that other like maybe smoke marijuana in their music videos and them the child will also want to do this, try marijuana see how it’s like and yet it’s not right sometimes.

I: Mmh, so you saying that maybe sometimes people are influenced by celebrities in a negative way?

P: Yes because this person who is a celebrity is old and knows what they are doing and how life is like you see. He/she does their own thing because they know why they are doing it. Us we copy because we don’t know what is going on that this thing is good you see. Us, we don’t know how stop using these things.

I: But maybe do you think celebrities could also be used maybe to…give people or the youth information about HIV?

P: Yes.

I: How so?

P: Like maybe like celebrities maybe can come to the location like maybe so that they can tell people like what’s life like with HIV. Like explain so.

I: Mmh.

P: Yah, people can come when there are celebrities because they’ll want to see them, touch the like that you see.

I: Mmh.

P: Yes.

I: Uhm these benefits a- so let’s go back to the benefits of social media. So things like WhatsApp and Facebook that you mentioned right, so you’ll be able to reach obvious a lot of people at these things right. What are the benefits do you think they are with using things like Facebook like to reach the youth?

P: Yah, like many people like, they like to on Facebook, WhatsApp basically social media.

I: Mmh.

P: So if you can…like go there and maybe, maybe have one celebrity that backs up your thing they’ll come in numbers like you see so how is does thing happen.

I: Mmh.

P: Yes.

I: And then what kind of celebrity do you think this will be? Maybe give me an example.

P: Yes! Actresses like you see. Familiar people on TV, yes.

I: Mmh, okay. And then so you were saying that other parents they, they don’t want their children to- to learn HIV or anything on social media or cell phones because maybe they are scared or something like that-

P: Yes, they are scared that they will get bad influence.

I: Mmh, and then how do you think-how do you think we can overcome that?

P: Like maybe like show them that this is about learning so like you teach children what is HIV.

I: So like educator parents?

P: Eh! Like show parents that you’re educating their children about HIV.

I: How can we do that?

P: Like maybe like, eish make them support their children like show them or advertise on social media...

I: Mmh okay okay, so you’ve given us like some suggestions right like for examples of having celebrities, actresses, and then also posting on Facebook and posting on Facebook or WhatsApp and give out bangles, bottles, data, stationery and all these other things. Do you have any other suggestions that can encourage someone your age to come to the clinic?

P: Clinic?

I: Uhm, that to come and test or in any other HIV testing…spots. Suggestions?

P: Yah! Like others like maybe people would say they will be bored maybe say like which entertainment will be available maybe like or are they going to eat something like that, so.

I: Like games?

P: Yes!

I: Oh okay. So take me through that what types of games?

P: Like-

I: What type of games that could be happening there?

P: Like maybe, like maybe find a playground and then say it will be girls versus boys so, things like that. The game will be some sort of a challenge.

I: And then what does the winner do?

P: Maybe the winner gets something maybe like, like-

I: Things mentioned?

P: Yes maybe bottles, food and so on you see...

I: Uhum, and then like so this ground thing, ground right and then there’s a game that is being played between boys and girls, and then how do we get them to get tested then? How do we make them want to test then? So is it the game only whereby they win and then go home?

P: Like maybe they can start at the clinic right.

I: Okay!

P: Maybe, start with testing before being part of the game like that and so on.

I: So the, only the ones that have tested can be part of the game?

P: Yes, maybe like that, do it like that you see.

I: And then what if people don’t want to go test [door sound], mmh?

P: What?

I: What if people don’t want to get tested? Mmh?

P: Like maybe, maybe there is food maybe; maybe you get data, maybe stationery. Others will come maybe say we are just doing it to get all these things only, you see they will test. They’ll see like that you won’t die from testing. Let me just test get these things play the game and go home you see. It will be easy like if there are things they will get.

I: So you’re saying if we give them these things people can come?

P: Like maybe say they’ll just test only?

I: Yes.

P: Like others will say these people are wasting our time and also say they didn’t bring food so we can eat. At least people here at the location will say it like that.

I: Mmh, okay-okay. So uhm you’ve actually give- given use some, some suggestions that were really good. You also said one of the suggestions was to encourage the staff that they must do right-

P: Yes.

I: Mmh, so is there anything you would want to add onto that? Before we finish…we almost done, at the end. We almost at the end of our, of our…interview. Are there any final thoughts you have?

P: A-ah!

I: Anything you want to add before we finish?

P: No! it’s finished.

I: Ohkay! Thank you so much, uhm the time is 15…uhm, 15…what!? The time is 15:50. Thank you so much for being part of our interview. Thanks hey.

P: Okay.
