# Supplementary material for: Exploring perceptions of low risk behaviour and drivers to test for HIV among South African youth
Source: PLoS One. 2021 Jan 22;16(1):e0245542. doi: 10.1371/journal.pone.0245542 (PMC7822253; doi:10.1371/journal.pone.0245542)
Supplement: S1 File — (ZIP) [file pone.0245542.s001.zip › S1_File_Anonymised Transcripts/A02-041-NL TRANSLATION by Nokukhanya_QC2_TM.docx]

Full Participant ID: A02-041-NL

Participant Type: Female

Location: Daveyton Main Clinic

Date: 08 October 2018

Start time: 15:15

Primary interview language:

Name of Facilitator/Interviewer: Bakang Mosime

Name of Note Taker:

Name of Transcriber: Nokukhanya Ndinisa

Length of recording: 31:44

Label Key

I = Interviewer

P = Participant

N = Notetaker

{ } = Indicates that details were changed or pseudonyms were used to anonymise data

xxx = words were omitted to anonymise data

- = breaking into a sentence by the next speaker

… = pause or drawn out words

[ ] = indicates noise made, e.g. [laugh], [sigh], [pause]

[inaudible segment] = Unclear section of the recording

?Mulenga Clinic?, ?P3? = questionable text or doubt as to what was said or who said it

I: Thank you for agreeing to participate on our study. So, do you allow me to record our conversation?

P: Yes.

I: Thank you. Can you tell me your thoughts about HIV? What do you understand by HIV?

P: Uhm, I understand a lot about HIV, like people with HIV must be taken care of. They must eat healthy food, take their medicine and go for their check-ups… Uhm, yeah!

I: Okay. So, how can you become infected with HIV?

P: When you stay with someone who has HIV, if they get cuts and their blood mixes with yours. Some get it when they are born or when you have sex with someone who has HIV without using a condom.

I: Okay.

P: Yeah.

I: That’s good. Can you tell me places where a person is at risk of getting HIV?

P: Uhm… I don’t understand the question.

I: Uhm, what are the places where a person can get- is more likely to get HIV at?

P: Oh! Parties, street bash, uhm… when someone comes back, it is dangerous there by the bridges at night. And event-

I: What kind of event?

P: Maybe yoh… Uhm… I don’t know.

I: So, can you tell me about any situation where you felt like you were at risk of getting HIV?

P: Yoh! The way I saw my mom always testing.

I: Hmm.

P: Yeah. So, I also thought that I have HIV. Always, she was checking and telling us how it went, when coming back.

I: Okay. So, you felt like maybe you also-

P: Have it?

I: You might have?

P: Yeah.

I: Okay. So, about the HIV testing services that are taking place in your area. Can you tell me about them?

P: Like how?

I: Uhm… Can you describe the testing H-- the HIV testing services that are taking place in your community?

P: Testing places? {XXX} (Name of store), {XXX} (Name of clinic), and at- Yeah, I have only seen these so far.

I: Okay. So, are these services youth-friendly?

P: [Silence]

I: Are they youth-friendly? Do they allow young people to come and get tested?

P: Yeah. Like starting what age?

I: Maybe 15 to 24.

P: Yes. they are friendly, yes.

I: Okay. So, where do they happen?

P: What happens where?

I: These HIV testing services. Where in your community does they take place?

P: {XXX} (Name of store)

I: Okay.

P: And at {XXX} (Name of place).

I: Okay.

P: And at clinic, outside.

I: Oh, outside the clinic? And who does them? Who is testing?

P: Who tests people?

I: Hmm.

P: I don’t know. I don’t know them, like I have never seen that I know testing people.

I: Hmm… Okay. So, is it nurses, maybe?

P: Oh! Yeah, nurses.

I: Okay. So, in your own opinion, what are the positive aspects of the current HIV testing services that are taking place in your community? What is positive with- those testing places?

P: Yoh, I don’t know.

I: What he- e-e helps the community?

P: To?

I: With testing services they have around the community.

P: What helps the community?

I: Hmm.

P: So that they-

I: What e- We are talking about HIV testing services, right?

P: Yeah.

I: What you see outside the clinic, {XXX} (Name of store)

P: Yeah.

I: How do you think helps the community there?

P: It helps by telling them to go and take ARV’s so that their HIV goes well, how to take treatment, how to behave and what to eat and use.

I: Okay. So, what do you think are the main negative aspects of HIV? You already told me what is positive, right?

P: Yeah.

I: So, what do you think doesn’t work?

P: Food.

I: What does food do?

P: They tell others what to eat, right?

I: Hmm.

P: And most of the people, what helps them are ARV’s and pills healthy food ah, most of them don’t even finish it but ARV’s help them

I: Okay. So, during these HIV testing services you saw outside the clinic, so people go though?

P: Yes, they do.

I: They go and test?

P: Yeah, they go. Especially the youth.

I: So, how do you think incentives can be used to encourage youth to get tested for HIV and access treatment?

P: [Silence]

I: How can incentives help-

P: To make the youth come and test?

I: Hmm.

P: Uhm, by telling them about how they can get HIV or how to get treatment if you have it and what will happen to you. When others have HIV, their body changes. So, when they want to test, they must tell them when to come and test for HIV and how to treat it so that you can be fine. Bodies also get affected-

I: Okay. Let’s try to explain what you understand by the term incentive. What are incentives?

P: I have never heard that name.

I: Okay.

P: Yeah.

I: Incent- we can say that incentives are things you can you-

P: Give?

I: Yes. So that you can test.

P: Okay.

I: So, do you think if we- health workers are giving those incentives can encourage youth to come and get tested or access treatment?

P: Yeah, it can help.

I: How?

P: Cause I’m sure when they give you incentive, right?

I: Hmm.

P: It tells you same time if you’re positive or negative. Yeah… yeah then they will come and test and then get treatment.

I: Okay. And what type of incentive do you think youth would value that can help them to encourage them to come and get tested or access treatment. What type of incentive?

P: T-shirts.

I: T-shirts.

P: Uhm, bottles.

I: What kind of bottles?

P: The bottles.

I: Water bottles?

P: Yeah. And caps.

I: Others?

P: Uhm… Yeah, I’m only thinking about these… Phone.

I: Cell phone?

P: Yeah… And what? Money.

I: Name anything that you can think of. Anything that youth would be happy to get.

P: [Laugh] And watches.

I: Watch?

P: Yeah.

I: Okay.

P: Bracelets, things like that.

I: So, out of all of this, what are your top three items that you can value? That you think youth can value.

P: Cell phone.

I: Cell phone.

P: Money.

I: Money.

P: T-shirts.

I: Why all these?

P: Cause some of us don’t have phones. Other people need money because of their situation at their homes. So, they can come and test so that they get and others, t-shirts. Yeah t-shirts.

I: Okay. So, cell phones? What would you do with them?

P: Cell phones?

I: Hmm.

P: To connect with many people, our family, friends… uhm, and other people.

I: Okay.

P: Yeah.

I: So, as for youth… Cell phones, what do you think youth uses cell phones for, most of the time?

P: Social media… WhatsApp… Social media.

I: Okay.

P: And WhatsApp… Uhm, Facebook, Instagram, YouTube, yeah.

I: So, do you think if we were to give cell phones to young people-

P: Yeah.

I: Uhm, do you think then maybe we spread messages about HIV testing services youth will be interested?

P: Yeah, they will be interested.

I: Okay… And how are they going to access this information? How are they going access this information on YouTube, Instagram?

P: By maybe if we say… Uhm… post.

I: Post on Facebook?

P: Yeah. Data-

I: Okay.

P: Dates.

I: So, data must come with the cell phone or?

P: Like-

I: Must it be a part of incentives?

P: Yes. Data must come with the cell phone.

I: So, do you think data can be used as an incentive?

P: Yes.

I: And how often do you think we should give out these incentives? When should we give incentive?

P: When should you give them?

I: Hmm.

P: During school days, cause everyone is at school, right?

I: Hmm.

P: They attend school. And during holidays, some come and visit.

I: During school holidays?

P: Yes, and during school days.

I: And what could be the challenges of providing these incentives?

P: Uhm…

I: What could be a problem when giving out these?

P: The phones?

I: Yes.

P: What could be a problem?

I: Hmm.

P: Like how?

I: We spoke about giving out-

P: Phones for people to come and check-

I: Money, t-shirt. So, what could cause the problem?

P: Most of them, won’t use the cell phones to search about HIV. They will use it for their own things, organize parties, music and taking pictures.

I: Okay. And what could be the benefits of these incentives for HIV testing services? What can they benefit?

P: Uhm… yoh! I also don’t know.

I: By giving you money, t-shirt, water bottle, caps. What could be the benefits of giving you all these?

P: To help us. This helps us to come and test, right?

I: Yes.

P: Yeah, we will benefit a lot. We will benefit a lot of things. Uhm, we will- What can I say? I don’t understand.

I: Can you describe your thoughts about being contacted via telephone or social media for HIV testing services?

P: I would-

I: How would you feel maybe if we call you or send messages via social media, telling you about HIV testing services?

P: I would feel… Uhm, happy. Happy, cause the more people tell us about HIV information, the more I will get help about how to treat HIV.

I: Okay. So, you think being contacted via telephone or social media can be a good thing?

P: Yes.

I: Okay. Maybe describe to me some examples of how you have been informed about HIV testing services.

P: I should describe in two sentences?

I: Yes. How have you been told about HIV testing services?

P: Uhm, they can tell us about HIV and that it is not the end of your life if you have it. Uhm, because if you have HIV you are like everyone else, it’s just that there’s a disease.

I: Who told you that?

P: It’s ma’am.

I: So, at school?

P: Yes.

I: Oh. So, anywhere else?

P: Yes, and my mom.

I: You talk to your mom?

P: Yes, she talks.

I: Okay. [Background noise] How would you feel if we were to inform you or register you for HIV testing services using cell phone?

P: How would I feel?

I: Hmm.

P: I wouldn’t have a problem.

I: Why? Can you explain why maybe you wouldn’t have a problem?

P: Because I want to be sure that I don’t get it and I want to know how to protect myself so that I don’t get it.

I: Hmm.

P: Yeah and to always know when to attend events about HIV, and how to be protective because they will tell me what to do if I don’t want to have it.

I: Earlier on, you told me how cell phones can be used to inform youth about HIV services. You told me about social media. Can you tell me about any other way we can use cell phones to inform youth about HIV?

P: Hmm… Send SMS.

I: Okay, SMS.

P: And calling

I: Telephone?

P: Yes.

I: Any other way?

P: No, I don’t know.

I: So, do you think there could be some challenges giving information about HIV services via telephone or social media?

P: No, never. There won’t be challenges.

I: Can you explain why?

P: Cause some people have HIV and they’re scared to talk about it but the more you send messages, the more they will talk, because they’re scared since people like laughing at those with HIV.

I: So, the benefits? How do you think this could benefit- using cell phones could benefit youth?

P: Uhm… It will… [Sigh] Let me see.

I: How will it encourage? How will this thing of using cell phone to encourage them to get tested?

P: Okay, you send message to a person’s phone, right?

I: Hmm.

P: They will see that message than to tell people in front of their friends.

I: Hmm.

P: Uhm, I think it’s a good idea to send messages so that they can test.

I: You told me about the types of social media that could be used to contact youth for HIV testing services. You told me about YouTube, Facebook, Instagram. What other platforms can we use besides these ones?

P: Uhm…

I: Any social media platform that you can think of?

P: WhatsApp.

I: Okay, WhatsApp.

P: And what… What? And Twitter.

I: Do you think youth use this social media?

P: Yes. Yes, they use it.

I: Can you explain to me. How do they use it? What do they do? What do they want?

P: Some want pictures. Some want music and artists

I: So, if we use these social media platforms to talk about HIV, inform people about HIV people- youth about HIV, do you think they will read the post?

P: Yes, they will read it, because it is important to know your status and where you stand.

I: Okay.

P: Yeah.

I: So, to make it look interesting, what kind of message do you think we should write?

P: Uhm… To make the message interesting?

I: Yes.

P: Ask the youth to test for their own good, cause some of them are being raped and they fear talking. By bad luck, some get HIV, without knowing. So, ask them to come and test so that they know where they stand.

I: What could be the challenges of using social media to contact youth for HIV testing services? What challenges can be there when using social media to tell youth about HIV testing services?

P: Uhm…

I: What… Problems that youth can face when they are informed about HIV testing services via cell phone… or social media?

P: Some would commit suicide.

I: Okay.

P: Yes.

I: So, what would make them commit suicide?

P: Shock. They don’t want people to know about status and that they have HIV.

I: And what could be the benefits? You told me about challenges, right?

P: Yes.

I: What could be the benefits of using social media to contact young people to access HIV testing services? How would it help?

P: Help to?

I: Write on social media about how youth can access HIV services?

P: To consult with you.

I: Okay.

P: Yes… Then they should talk to someone about HIV.

I: Anything else?

P: Huh-uh.

I: Okay. About yourself, how do you think your parent or legal guardian would feel about receiving information on HIV testing on your cell phone or social media? How would your parents feel if maybe they find out that you get information about HIV on your phone? How would your mother feel?

P: She will be happy because she always tells us about growing up as a girl and getting married, maybe to someone with HIV, then you must use protection. She would be happy that we know all information about HIV so that we know how to prevent getting it.

I: So, she won’t have a problem about you receiving information about HIV on your phone?

P: She won’t.

I: Okay. Can you tell me other suggestions that you have which will encourage youth to test?

P: Uhm…

I: Any suggestions?

P: If they write posters that maybe Saturdays or Friday afterschool, there is a place where you can meet and talk about this or they can… they can contact them. Especially because today’s youth has Facebook, they can post on Facebook about this and this and that.

I: Okay. And about the gathering, you said they can meet. What kind of gathering do you think youth are interested? What do they like?

P: Young people, most of them like acting.

I: Acting?

P: Yes.

I: Okay.

P: Others like choirs.

I: This gathering would be uhm… drama, music-

P: Yes.

I: What kind of music?

P: Uhm…

I: They like- What kind of music does the youth?

P: House.

I: House music?

P: Yes. And others gospel.

I: Okay. Any other suggestion you can think of?

P: Uhm… And the suggestion I have is that I think churches and schools should encourage youth to test and at school assemblies.

I: Any other suggestion?

P: No. [Sigh]

I: Are there any final thoughts you have about youth, HIV testing or incentive? Any final thoughts? Something that you missed about HIV, HIV-testing, about youth or about incentive? Do you want to add to your list maybe?

P: Huh-uh.

I: What can you think of maybe? When you go to test, do you think youth can need refreshments like food or-

P: Yes.

I: Okay. While waiting ne?

P: Yes.

I: So, what kind of food do you think youth would love, would like?

P: Fruits.

I: Fruits? Okay.

P: And vegetable.

I: How should the vegetables be? cooked or raw?

P: Cooked.

I: What about stationery? Let’s talk about stationery. So, you think you have enough stationery?

P: Huh-uh.

I: Do you think it should be part of incentives?

P: Yes.

I: And what kind of stationery?

P: Books.

I: What form of books?

P: Textbooks.

I: Okay.

P: Ballpens, rulers. Then, uhm... scissors.

I: Is that all?

P: Maybe and uniform.

I: What sort of uniform?

P: Shoes.

I: School shoes.

P: And jerseys.

I: Okay.

P: And trousers.

I: So, out of these incentives, what do girls, young girls need most?

P: Pads.

I: Sanitary pads.

P: Hmm.

I: Okay. Any other thing?

P: Things we use for bathing.

I: Toiletry?

P: Hmm.

I: And what should be in the toiletry pack?

P: Soap, Dawn and roll-on.

I: Do you have anything to add? Out of everything you have been talking about?

P: Huh-uh.

I: Now we’ve come to the end of our discussion. Thank you for taking part in our study. If you have any questions, maybe later on or maybe you need more information or clarity about something, use the number on your consent form to call between 8 and 5 o’clock.

P: Okay.

I: Thank you once again for taking part on the study.

End time: 15:47
