# Supplementary material for: Exploring perceptions of low risk behaviour and drivers to test for HIV among South African youth
Source: PLoS One. 2021 Jan 22;16(1):e0245542. doi: 10.1371/journal.pone.0245542 (PMC7822253; doi:10.1371/journal.pone.0245542)
Supplement: S1 File — (ZIP) [file pone.0245542.s001.zip › S1_File_Anonymised Transcripts/A02-042-NT_08102018 TRANSLATION_QC2_TM.docx]

Full Participant ID: A02-042-NT

Participant Type: In depth interviews, female, 15

Location: Chief Albert Luthuli Clinic

Date: 08 October 2018

Start time: N/A

Primary interview language: English/IsiZulu

Name of Facilitator/Interviewer: Wellington Maruma

Name of Note Taker: N/A

Name of Transcriber: Siphelele Khoza

Length of recording: 25:26

I: Thank you so much for being part of this interview. Do you allow me to record this interview?

P: Yes.

I: Thank you sister [Laughs]. So uh can you tell me what you think of HIV, like what is HIV; in your own thoughts?

P: HIV?

I: Uhm…according to you what is HIV?

P: A disease.

I: Uhm.

P: Transmitted such as when having sex with another person without using a condom. You can get HIV that way and when you mother is pregnant with you, you can get HIV that way. Also when sharing a toothbrush, when I have bleeding gums and HIV positive I can infect you that way.

I: Uhm. So those are the only things tha like those are the only ways you know that you can infect a person via them?

P: Yes.

I: Ok. And then you yourself, have you ever felt or been in a situation where you felt like you were at risk of getting HIV? …Was there a time when you felt as if or thought I am infected or might be infected?

P: No.

I: Never!

P: Yes.

I: So you think your you’re clean?

P: Yes.

I: Ok. And then why do you think that is?

P: [Inaudible segment 00:02:02-00:02:04]

I: Mhmm?

P: [Inaudible segment 00:02:04-00:02:05]

I: Why do you think like that?

P: Because-.

I: Why do you think you will never get HIV?

P: Because the people I play with are the people I know. They have not told me they have HIV, I also don’t know if they have HIV. So therefore, I cannot just go to them and say I am not going to play with you because you have HIV.

I: Mhmm-.

P: Yes.

I: So you think because of that you are safe?

P: Yes.

I: Ooh. Ok. And then so do you know any testing services for HIV? Where HIV tests are done, which places do you know?

P: Uhm, they usually put out tents for testing asking people to come and test, usually they are stationed at the mall nearby Shoprite.

I: Uhm. Do you also know about the clinic-?

P: Yes, also here at the clinic.

I: And then, so for you because you’ve never tested before; where would you like to get tested?

P: I come here at the clinic to test-.

I: Why at the clinic? Why not go to the tents?

P: I don’t hear you.

I: Ah. Why go to the clinic not to the tent? Why do you choose the clinic?

P: Because I think it is better than the tents because there are doctors who can check me. At the tents there are no doctors.

I: Uhm. So you are saying you’’ll never test get tested on the tents?

P: I would test there but I would rather test at the clinic-.

I: Which one do you prefer? Do you prefer the clinic?

P: Yes.

I: Because it’s better?

P: Yes-.

I: Why is it better? [Laughs], (5 seconds pause). Why do you think it is better than tents?

P: Because here at the clinic it’s a closed space where only two people can be in the room; but when using the tents anyone can see that you went in to test, things like that.

I: Mhmm. So at the clinic there is privacy-?

P: Yes-.

I: Like, people don’t see you…Mhmm. So when you think about it if people see you will that be a problem?

P: It’s just that everyone would say they saw me going to test, maybe I have HIV.

I: Mhmm. So people talk?

P: Yes.

I: What do they say? [Laughs].

P: [Laughs].

I: What do they say exactly at the clinic, I mean in the community?

P: They talk about you say you have HIV, they’ve never even went to check with you and they don’t even know you have HIV. They go around talking saying this child; don’t play with her and don’t touch her, things like that.

I: Mhmm. They talk like that? Mhmm Okay. So because of that you would rather get tested here at the clinic because people talk and all that? Mhmm? I’m saying you would rather test here at the clinic then?

P: Yes. Yes.

I: Mhmm Okay. So any other reason-any other reason as to why you want to test at the clinic not at the tents? No reason?

P: There is none.

I: So what is the good thing about testing here at the clinic?

P: It’s like a closed place-.

I: Closed! Ok, its privacy.

P: Yes.

I: Ok and then what else?

P: [4 Seconds pause]. Ay! I don’t have another.

I: Uhm. Do you think there is any bad thing about testing here at the clinic?

P: Ah nothing because you come here to test not knowing whether you have HIV or not.

I: Mmm.

P: Yes.

I: So you said in the community people talk?

P: Uhum.

I: At the clinic, don’t they talk when you’re done testing?

P: We don’t know what do they say when we’re gone.

I: Mhmm. So you don’t think it can be a problem right? Mhmm?

P: I don’t think so.

I: Why do you think so?

P: Because when I come to the clinic I’m sort of taking a break from my community with the thought that maybe nurses will not go around talking about me because they don’t know me.

I: Mmm.

P: Yes.

I: Ok. So do you think the clinic is accessible to the youth? Is it somewhere where you can go or?

P: Yes.

I: Uhm. [4 Seconds pause]. Mhmm? Relax [Laughs]. Mmm okay so when I speak of incentives what do you think, what comes to mind? When I speak of incentives, what comes to mind?

P: Like you mean things like {XXX} (Name of organization)? Things like {XXX} (Name of organization)?

I: What does {XXX} (Name of organization)do?

P: They say to people come and test for HIV we will give you bottles or stationery-.

I: Bottles and stationery-.

P: Yes-.

I: {XXX} (Name of organization)gives people stationeries?

P: Yes.

I: Ok.

P: And and then children will come running to test because they heard they will get bottles and stationery.

I: Uhm. And then for yourself, what would you like to get for you to come and test? Just bottles and stationery, or is there anything else?

P: Uhm, I would want to be given a phone.

I: A phone?

P: Yes.

I: Why a phone?

P: A phone is better than bottles and stationery because bottles get lost and the stationery gets finished off, but then a phone is always there.

I: Mhmm, what else?

P:? There is nothing else?

I: So th- those three things are the only things that will encourage you to go and test?

P: Yes.

I: Only?

P: Yes.

I: Nothing else? What about food?

P: Yes…food, no!

I: It’s not important for you right? Ok and what about t-shirts?

P: Yes.

I: So you sta you saying stationeries, bottles and cell phones are the only things you need, or is there anything else?

P: No.

I: Ok. What else?

P: Ehm…laptops maybe and school uniform.

I: School uniform?

P: Yes. Mhmm-.

I: Only?

P: Maybe also school bags.

I: Uhum.

P: Yes, that’s all.

I: Ok. So you think that school bags, uniforms, laptops an-are something that would make people to want to come to the clinic to test?

P: Yes.

I: Ok. Why do you think like that?

P: Because people here like things. If you were to say come and test and we won’t give you anything, not even one person would come. But, if you were to say come and test there are laptops, they will come running.

I: Mhmm. W- why are they like that? [Laughs].

P: They like things. [Laughs].

I: So you saying that they will not want food?

P: They won’t.

I: Serious?

P: Serious!

I: Ok. And what about maybe cause other people don’t have phones right? So what about data? What about airtime?

P: No! They’ll have to hustle for themselves. They can’t expect to be given laptops and airtime on top of that, they can’t!

I: Mhmm. But do you think airtime will work?

P: They won’t work...

I: They won’t work right? Ok so you’ve mentioned stationery, bottles, cell phones, laptops, uniforms and school bags right?

P: Yes-

I: So out of these things which are your top three?

P: Yes.

I: So let’s say I say I’ll give you only three things out of these things-.

P: Yes-.

I: Which ones would you choose?

P: I would choose a laptop-.

I: Uhum, as number one?

P: Yes.

I: Ok.

P: Followed by school uniform,

I: Uhum.

P: Then school bags.

I: Uhm.

P: Yes.

I: So these things are important for you; laptop, uniform and school bag.

P: Yes.

I: Uhm OkOk. And then so, ah let’s say they say they’ll give you these things right!

P: Yes.

I: So would you, how often would you want them? Would you want them every day, every month, every two days, every three days, every… how many times would you want them?

P: Once.

I: Once?

P: Yes.

I: When you come to test?

P: Yes.

I: And then what if come again to test?

P: Yes, I will come without expecting to get other things because I would have been given them before. I would come this other time even without the availability of these things.

I: Uhm.

P: Yes.

I: So why do you think maybe other people would want these things every time they come to test?

P: Because they’re not thinking of other people who haven’t received these things, they just want to get them again even though they have had a chance before.

I: Uhm.

P: There are others who also need these things, but those who have received them want to receive them again.

I: Uhm OkO. So so uhm you think only some people come for these things because like, just because they are there not actually for testing-?

P: Not for testing.

I: Ok. And then what are, what are other challenges do you think are there for giving people these things? Do you think there is any bad thing for giving people these things or is there any good thing?

P: No, there is nothing wrong with giving people these things.

I: Ok, and then any good thing?

P: It’s just that they attracted us with these things and influenced us to come to test. What they wanted us to do was to come and test not to come for laptops-.

I: Uhm.

P: We came to test not for laptops!

I: Uhm-.

P: But what we were thinking in our minds is that we came for laptops not for testing.

I: Uhm.

P: Yes.

I: Ok. So you think that maybe the problem for giving out these things would be that people won’t come to the clinic?

P: Yes.

I: [Inaudible segment 00:12:29-00:12:30].

P: Yes.

I: So what can we do for people your age to come to the clinic to test? Do you have any suggestions maybe?

P: No.

I: Mhmm?

P: No, I don’t have.

I: So what would you like for yourself that would make you to come to the clinic to test?

P: [3 Seconds pause]. Eish, I don’t know?

I: Only these things…so if these things are not available you won’t come?

P: I would come.

I: So what else would bring you to to test?

P: When…if they can say we are giving out vouchers for grocery, well on that note I can come. That’s because even at home, they need grocery so I can definitely come to test.

I: Uhm. So grocery voucher?

P: Yes.

I: This voucher you’ll use for buying grocery?

P: Yes.

I: Oh Ok alright like at {XXX} (Name of store)…{XXX} (Name of store)-.

P: {XXX} (Name of store)

I: For how much?

P: R3000.

I: Uhum Ok. So the voucher will also be given out once?

P: Yes.

I: After you’ve finished testing?

P: Yes.

I: Oh OkOk interesting. And then what else do you think can be done so that people can come to test?

P: [6 Seconds pause]. It’s that (tongue click) people from {XXX} (Name of phone service provider) say they’ll give you sim cards which have R30 airtime in them. Yes, then people will come running because they heard they’ll be getting sim cards with R30 airtime. When they insert them then find that there is no airtime there it was just a way of calling out people to come and get sim cards.

I: Mhmm. [Laughs]. So {XXX} (Name of phone service provider)’s giveaways for people-.

P: Sim cards-.

I: With no airtime-.

P: Yes, they say it has R30 airtime-.

I: Uhum.

P: When they insert them, they have no airtime.

I: So [Laughs]. So what if they find out when they’ve tested? Won’t they tell other people not to come to test?

P: [Inaudible segment 00:15:01-00:15:02].

I: So don’t you think that would be a problem?

P: It will be a problem, but it would have been already done and there is nothing they could do to change it.

I: Uhm. Ok, Ok. So you said you don’t have a phone right? So let’s say, let’s say you had a phone; ah, how can we use it to give you information about HIV? How do you think we can use the phone for you to get information about HIV?

P: I can research; research about HIV, or go to the internet, ask people that know about HIV to give me information-.

I: Uhm, through the internet? And then, how else? How can you use it?

P: For googling.

I: Uhm, only?

P: Yes.

I: Ok. What about maybe Facebook? How can we use it, do you know? (5 Seconds pause). Mhmm?

P: Facebook?

I: Do you know social media?

P: Yes, I know it.

I: What do you know?

P: I hear they say there are free things.

I: Uhum. Facebook and WhatsApp, do you know those?

P: I know them because my sister uses them.

I: How can we use it? (5 Seconds pause). Mhmm?

P: By asking people who know HIV better in terms of what is it and how do you get it? Then they’ll give me better information than what I what I already know.

I: Uhm.

P: Yes.

I: So you said your sister uses WhatsApp?

P: Yes.

I: So let’s say you receive i-information about HIV on WhatsApp. What do you think your mother would say or your father, your parents?

P: About HIV?

I: No! For receiving information about HIV. On what do you think about it? Do you think they would be happy about that, or?

P: They won’t be happy about it.

I: Why? (8 Seconds pause). Mhmm? (6 Seconds pause). (Loud breathing). Why do you think your mother won’t be happy that you are getting information about HIV and AIDS? Why not on your phone?

P: Because we won’t know if she has HIV and that would make her sad; that we are researching about HIV whereas she has it, things like that.

I: Uhm. But but, why why would like (loud Breathing); why do you think that she will be sad…be sad? Don’t you think she will be happy that her children know about this HIV?

P: Yeah she’ll be happy that we won’t go sleeping around with ever other person without a condom not knowing whether they have HIV or not.

I: Uhm. But you said your mother will not be happy. Mhmm? (5 Seconds pause). So which one is it; will she be happy or not happy?

P: She will be happy.

I: Why?

P: Because we will know better about HIV, how you get it, ways you cannot get it; things like that.

I: Uhum. (Inaudible 00:19:27-00:19:80) protect you.

P: Yes.

I: Ok. So for someone who doesn’t have a phone, how can we then send i- information about HIV on the phone if they don’t have one? That is a challenge right? So what do you think we can do about that? How can we get information out there? (3 Seconds pause). If they don’t have phones what can we do so that they can get i-information?

P: We might have to buy the person a phone.

I: Uhm.

P: So that the person can also be able to get information in a better way on how to prevent HIV.

I: Uhm. But what if the person does not have a phone, like you right now you don’t have a phone? how would you like to receive information about HIV, how?

P: I can tell my sister to research more for me about how you get HIV, what’s HIV like; things like that.

I: Uhm. And then you, do you have any other suggestions that we can use?

P: No.

I: So, you think that only sim card for R30 will work?

P: Yes.

I: Mhmm?

P: Yes.

I: If it doesn’t work what can we do? (10 Seconds pause). Mhmm?

P: If it does not work?

I: Uhm! (4 Seconds pause) What else can we do?

P: We can do a Saturday service whereby children my age would come and be offered food such as bread and juice. Sometimes having food is right; yes.

I: Uhm-.

P: They will also come in numbers because they heard there’s going to be food.

I: Uhm

P: Say they came to check.

I: Ok. Ok, and then uhm do you have any final thoughts we almost at the end of interview? Do you have any final thoughts that you want to add to the interview? Maybe something you forgot to say!

P: [Inaudible segment 00:22:07-00:22:08]

I: Mhmm?

P: Nothing.

I: Nothing?

P: Yes!

I: Ok and then do you know of Instagram?

P: No.

I: You don’t know it? Ok and then so let’s say at the clinic they say they will call you, maybe if you have a phone right. They’ll call you and ask to come and test at the clinic. Would that make you happy?

P: Yes, that would make me happy.

I: Why?

P: That would make me happy because I wouldn’t have known my HIV status and I would like to know whether I have HIV or not. I would like to know because; you can get HIV if you have a cut without even realising it then help someone, then end up having HIV. So that would make me happy to come and test at the clinic.

I: Uhm. And then what about maybe getting a sms that you should come to test? What would you prefer; a sms or a phone call?

P: It’s better to receive a phone call that will tell me on which date to come or day to come to test.

I: Uhm Ok. And then uhm…Ok. So and then you said these things; stationeries, bottles, cell phones, laptops, and school bags you only want to get them once, after-after you’re done testing?

P: Yes!

I: Ok. Why not twice, three times, every time you come to test?

P: Because we have to give others a chance. They also wanted to receive these things and they didn’t get a chance to because they were finished, so others should be given a chance.

I: Uhm Ok. And then what do you think is there uh like a challenge about that? That people who come to the clinic just to test, I mean to just receive those things? Mhmm? (3 Seconds pause). Challenges for example; you know that people may come to the clinic just to get food, you said bread and juice-.

P: Yes-.

I: Every Saturday right?

P: Yes.

I: And do you think they’ll come for because of these things or?

P: Yes, they’ll come only for these things because they would have heard that next week there won’t be any incentives to be given away; that testing would be done solely without providing incentives, therefore, they won’t show up at the clinic.

I: Uhm…Ok thank you so much we we are at the end of our interview. Do you have any final thoughts you want to add?

P: No.

I: Thank you so much for being part of the interview. [Clears throat]. Uhm the time is 16…16:32. Thank you.

End time: 16:32.
