# Supplementary material for: Exploring perceptions of low risk behaviour and drivers to test for HIV among South African youth
Source: PLoS One. 2021 Jan 22;16(1):e0245542. doi: 10.1371/journal.pone.0245542 (PMC7822253; doi:10.1371/journal.pone.0245542)
Supplement: S1 File — (ZIP) [file pone.0245542.s001.zip › S1_File_Anonymised Transcripts/FGD01_Females (18-24 year olds) Translation_QC_TM.docx]

Full Participant ID: FDG01_Females (18-24 year olds)

Participant Type: Focus Group Discussion

Location: Winnie Mandela

Date: 14/08/18

Start time:

Primary interview language:

Name of Facilitator/Interviewer: Wellington Maruma

Name of Note Taker:

Name of Transcriber: Ornate Masuku

Length of recording: 1:13:28

Label Key

I = Interviewer

P = Participant

N = Notetaker

{ } = Indicates that details were changed or pseudonyms were used to anonymise data

xxx = words were omitted to anonymise data

- = breaking into a sentence by the next speaker

… = pause or drawn out words

[ ] = indicates noise made, e.g. [laugh], [sigh], [pause]

[inaudible segment] = Unclear section of the recording

?Mulenga Clinic?, ?P3? = questionable text or doubt as to what was said or who said it

I: Focus group discussion, uumh the date is the 14^th^ of August 2018 uumh with females aged 18- 24 at {XXX} (name of an area FGD took place)and the facilitator is {XXX} (interviewer name), the scribe is {XXX} (scribe name), and we have five participants with unique identifier numbers starting from 1-5. Thank you guys for agreeing to be part of this focus group discussion, do you allow me to record this focus group discussion?

P2: Number 2 says yes

P3: 3 yes

P4: 4 yes

P5: 5 yes

P1: Number 1 yes

I: Thank you guys. So ummh I just would like to find out from you why you got tested for HIV? We can start with number 2 why did you get tested for HIV?

P2: I was at the career expo last of last month, so there was a tent where they were testing people so I decided to go there

I: Ok

P2: Yea

I: Ok and number 3?

P3: Ok, last year I was pregnant, I got tested there

I: And if you were not pregnant would you have decided to get tested?

P3: I wasn’t because I was kind of afraid

I: Ok. Number 4

P4: Uumh every year I go testing, so whenever the year starts then I get tested. So this year I went on the 29^th^ of June, so practically every year I go, I just want to know my status

P5: Number 5, I tested uumh the first time that I tested was …2015 because I didn’t use a condom so I wanted to know my status

P1: Number 1: first time I got tested was last year. I had an incidence where I slept with like two people and I didn’t use protection and I decided to test

I: Ok and number 4 you mentioned that you test every year why is that?

P4: Uumh I also have a boyfriend and so we don’t use protection so I just to be safe I get tested for my safety

I: Uumh ok. And then do you guys have any knowledge of uumh, do you know what an adolescent friendly clinic is or have you ever heard of that term?

P2: Number 2 says no, I don’t know

P3: No

P4: No

P5: No

P1: Number 1 no

I: Ok, so uumh youth friendly an adolescent friendly clinic is a clinic that provides friendly services to adolescents or young adults right this could be HIV testing this could be like anything that the adolescent might need so it just makes sure that every service that the adolescent might need they are available at the clinic whether family planning or HIV testing services. Do you think the clinics you guys got tested at were youth friendly?

P2: Number 2 says yes because eemh that they welcome you with a smile. I don’t believe that you can be afraid when you see them cause they are talkative and they allow you to ask questions before you even decided to go in and test, they don’t force you to go to test they just you can just go there and ask questions and leave or decide to go in after asking questions so it was so easy for me there

I: Uumh ok

P4: uumh number 4 I personally never went tested in a clinic I go to like those people who are located on the streets making tents so go there cause sometimes going to the clinic you can get a long queue, so there is little number of people going there cause they afraid of seeing by other people around. So I use those tents to go testing, clinic I have never went to the clinic to testing

I: Ok, and what do you think would make you want to go get tested at a clinic now that you say you have never been tested at a clinic?

P4: what would make me go to the clinic? Oh! Number 4 …its if they if they make sure that they work effectively cause sometimes there are long queue so people stand at the queue and end up wanting to leave so they don’t actually get tested due to that long queue so if they were get effective then I will think of going to test in the clinic

I: Uumh

P4: Yes

I: Number 3 do you have anything to say about what number 4 said?

P3: 3 no

I: Number 5 do you want to comment on what number 4 said regarding long queue at the clinic is that something you have experienced as well?

P5: Yes. Number 5 yes cause sometimes you find that I have decided to go and test and I find a long queue so I decide to go back because these people are not working they are slow so I end up leaving the clinic without getting a service that I wanted

I: Uumh and then you think what can be done regarding the long queues so that they are shorter so it can help more youth come to the clinic

P5: I think they must hire more people uumh so that they even reduce the high unemployment rate by hiring people

I: Uumh

P: So there can be 5 people in a 5 room consultation it makes more sense that there are 5 rooms were people get tested

I: Ok

P5: Yes

I: So those are like the negative experiences you guys have had at the clinic sometimes. And then the positives? Tell me maybe a positive experience of yours when you went to test. Was there any?

P3: 3 no I didn’t get positive going clinic

I: ok what was negative about your experience then? Do you want to tell me more about it?

P3: No it’s the lines,

I: Uumh

P3: You find at times there’s a lot of people that are waiting to be tested the whole day so ending up not tested cause of those lines and stuff

I: Ok

P3: Uumh

I: Ok number 5 wanted to add something

P5: Yes, one positive number 5 one positive things I got before they test you … they do counselling before, so they give counselling and they tell you if you find you are positive start your ARVs immediately so you are able to be protected don’t delay so yea that was my experience that I encountered when I wanted to test for the first time They did counsel me yes that’s why I agreed that I get tested and I left there happy

I: Uumh

P: Yes

I: Ok, so uumh number 2 do you want to add something to what number 5 has said? You look like you have something to say

P2: Yes number 2 says the positive thing again is that the they work with a lot of people even if you are from around they will never know that this person came at this day and like, some people even though they know that our results are confidential but as co-workers you can discuss that one came to test and his or results are like this. They work with a lot of people they won’t have time to recognise a person or to discuss about his or her results

I: Uumh

P2: So it’s a positive thing

I: Ok like, uumh having your confidentiality secured means you are more able to come to the clinic or something like that

P2: Yes cause if ever I come here and start hearing that people know my results even if I am negative or positive people discussing about my results I won’t be able to come again I will just maybe decide go somewhere else or never go testing again

I: Uumh

P2: Yea

I: Ok do you also agree number 3?

P3: Yes

I: You look so quiet, you want to say something?

P3: Yes I agree with her number 2 is right some of youth are afraid of the clinic cause of that some nurses are don’t know how can I put it but they just tell everyone and shout you see that one that came here and walked he or she is pregnant I mean HIV AIDS and stuff so we need some confidentiality sometimes

I: Uumh

P3: Uuumh

I: Ok I understand that, so uumh how do you think, so like I mentioned that a lot of youth are not getting tested right for HIV what do you think uumh you guys or the government or community what do you think can be done to get them to come and get tested?

P2: Number 2 ok I think like when there is a career expo like that. So there you know most of the youth will be gathering there so you just go there and be friendly like the people who are testing must be friendly and welcoming like they just don’t have to be strictly or they must just be able to talk with the youth. So I think the more they are opening up is the more youth can go to them and ask questions and need to go and test. So at the career expo and the schools sometimes you can go to schools then some of the youth will be testing there

I: Uumh

P2: Yes

I: Ok what do you think about it number 4?

P4: Uumh number 4 for encouraging youth to come testing government should, there are a lot of people who are professional and have this disease so I prefer that they must contact them maybe two or three like three people who are professionals and are well known to go to schools and the ones who are local people who were then go to schools and share their part of their story cause most youth when seeing someone who is educated and professional like they get encouraged so if they get that words from a professional person they will be more encouraged to get tested because they saw someone who like owns billions of companies like for example {XXX} (name of a well known South African businessman)everyone wants to be like {XXX} (name of a well known South African businessman)if a person like him goes to a school that would encourage a lot of youth to go testing because they know like that guy is well known with rich companies.

I: Uumh

P4: So I think they must get those kinds of people to go to schools and encourage learners to go testing

I: Uumh …number 5 number 5

P5: [clears throat] the question again

I: No anything do you want to add to what she said? Do you agree/ disagree with what she said?

P5: Number 5 I agree with her

I: Tell me why. What is it about what she said that you agree with, what do you agree with?

P5: Oh! The part that professional nurses must go to schools and talk to the students about how important it is to know your status

I: Ouch

P5: Because knowing your status so that if you are positive you immediately start treatment as early as possible cause all treatment has side effects

I: Uumh

P5: Yes

I: do you think that is something the youth have in mind like to know their status, like how important it is to know their status

P5: Number 5 no some they don’t some they do

I: And why do you think they don’t know the importance of knowing their status

P2: Number 2 some youth thinks that, that still have that mentality if you have sex or unprotected sex you will only be contacting HIV/AIDS so if ever eemh so if a person or youth does not his not sexually active they don’t think it is necessary for them to go and test they just think cause I am not having sex they just sit and not go to test

I: Uumh

P2: Yea

I: Ok, I would like you guys to just maybe give me a brief definition of what you think an incentive is. What comes to mind when you think of an incentive?

P1: Number 1 uumh incentives are something you get in return uumh for doing something

I: Uumh

P: Like someone can leave here and go testing isn’t they will be given t-shirts and caps, so incentives are those things

I: Ok and then number 3 you are too quiet. Do you want to add something number 2?

P2: Number 2 say no

I: Number 4.

P4: I think I am covered

I: What do you think an incentive is number 5 according to your own yea …or How do you think like number 1 mentioned that it is something given in order for you to do something right

P5: Uumh

I: How do you think incentives play a role in getting the youth to get tested wherever they choose to get tested? How do you think they play a role of incentives?

P2: Number 2 I think it will encourage them to go and test cause there will see something that they get from get testing so I think they will be more encouraged to go and test, Even them they can have that thing that they are getting in return

I: Uumh ok

P2: Yes

I: And number 4

P4: …Number 4 says I think giving youth incentives yes I agree with number 2 it will encourage them cause uumh our youth are being encouraged by just small things so if we are given something’s like hats, caps, t-shirts or bottles that will, they will go all over telling other youth that we got this and that so when you go testing you get this and that. So lot of youth will go there in order to get what other people got after getting tested.

I: Ouch

P4: Yes

I: Ok and number 1’s definition of what an incentive is she mentioned certain things like shirt, caps and number 4 mentioned bottles. What other incentives do you guys think could get youth to in addition to the shirt caps and bottles what else do you think can be…

P5: Number 5 food [laughs]

I: Why?

P5: [laughs] Number 5

I: Uumh

P5: According to my side like us youth we like food. Like if there is a tent belonging to {XXX} (Name of a non-profit organization)where people test and after you test you will be given a plate of food. They would understand that you are hungry and also someone can see you with a plate of food and you can tell them that after testing they gave me a plate of food, they would also go too cause they would be wanting that food too

P3: Number 3 I think pens will be right cause some youth are still in school so maybe they will some don’t have pens so I think pens will be right

I: And what else do you guys think you have mentioned shirt, caps, bottles, food I have heard pens or stationary right

P3: Yes

I: What else?

P?: Bags

I: Like school bags?

P: Uumh

P5: Number 5 uumh on the female side maybe sanitary towels yes

I: Uumh ok why do you think those would encourage someone to come and get tested? So if you don’t give them these things they won’t otherwise get tested?

P5: No from my side no cause

I: Uumh

P5: Because on the side of other people it is difficult to buy these things they don’t have the money and end up using toilet paper

I: Ok so in the... Ok so let me pause a question to you guy’s ok, say we don’t provide any of these things and we still expect the youth to come and get tested. How do you think that will affect the number of people that come to get tested? Do you think we would still have the number?

P1: Number 1 I don’t think the number will be the same it will go down because according to me the way that I see it a lot of people test because they didn’t get those incentives

I: Uumh

P1: Others they don’t test because they want to know their status uumh ummh

I: Uumh

P1: And then there are others who don’t want to know, they don’t want to know what they have so they don’t go there

I: Uumh

P1: So number will decrease compared to the numbers that we have now

I: Yea

P1: Uumh

I: Ok so one of the challenges is that people only come because we give them these things if we don’t give them they won’t come. What other challenges do you guys think there might be in providing these incentives?

P2: Number 2 I think people need to have that the reason why the number is it will increase/ decrease or stay the same people need to have a reason to go to test. Maybe I am pregnant I should go to test maybe someone he or she had an accident they will test him or her so they people will need to have a reason before they go test.

I: Uumh

P2: So the incentives will help then just to go to test because they will just go to test because of those incentives, like they will wait for a good reason to go test yea I think so

P3: Number 2 uumh number 3 [chuckles] I think the rate will decrease cause some people are afraid to get tested because they think someone just get some wrong results or something some are afraid because the of not handling the issue of the results. Another thing I think they have fear of losing their loved ones coming of not coming test.

I: Uumh ok makes sense. So you guys have mentioned that if maybe there was career exposition or something like that would encourage the youth to come and get tested right. Or if maybe like number 4 mentioned like if we have professional people and they go to schools and they give information about HIV testing they are more likely to get the youth to come and get tested. Can you guys think of other measures that can get the youth to come get tested for HIV over and above the ones you guys have mentioned? Anything that comes to mind?

P: Pardon?

I: Anything that comes to mind

P: The question again

I: Oh ok you mentioned that if there was a career exposition like at the career exposition that is where we are more likely to find the youth, and number 4 also mentioned that if there was like professional people to go to schools or like even people from the local community if they speak to you guys about uumh if they speak to the youth about HIV testing and these type of things you are more likely to get them to the clinic right. So I am asking over and above everything you have mentioned is there any other measure you guys think there can be put in place to get the youth to come get tested or to want to know their status?

P1: Number 1 I don’t think it’s there [laughs]

I: Uumh

P1: Like no I don’t think there is a thing that can be done to attract the youth so that they come test especially these days in the now world we live in. Like I don’t think so like it would take someone who is very creative to come up with a very good idea a brilliant idea that will attract the youth to come and test

I: That’s why we are here to get those ideas from the youth to see what appeals to you guys

P1: I don’t think it’s there

I: So you are saying we will never have the youth coming to get tested?

P1: Maybe it will be there isn’t

I: Uumh

P1: But personally I don’t think so like I don’t know

I: Ok is it like maybe now it won’t be possible but sometime in the future? And what would have changed in the future?

P1: Uumh, yho! Yho! It is hard to think that far, but maybe in the future yea

I: Number 4

P4: Uumh number 4

I: Uumh

P4: I also agree with number 1 cause the youth of nowadays this thing of testing aah! It’s like a wind that’s passing, they don’t even think of going testing so maybe in the future will get encouraged because these people dying according to this disease is not decreasing but increasing so we are losing our loved ones due to this disease, and not minding this disease so I think this issue must it’s also in the televisions cause this issue is affecting our people a lot so when youth are watching this TV they will see that lot of people are dying cause of this disease so I think they will see in the future that it is very much important to always know your status cause we are losing our loved ones due to them not minding their status so it’s an important thing to always know your status cause when not knowing your status it will be late by that time that you get that you have this disease and it will be that point even professionals won’t be able to cure you, it would have already affected that person a lot

I: Uumh

P4: Even radios and TV’s must make more effort on telling the youth how they should know their status because it’s an issue a huge issue

I: Number 1 you wanted to add something?

P1: Yea number 1 like being honest we don’t really care that someone woke up and decided that I am going to the clinic or let me go to the mall and test. Number 2 mentioned that one has to have a reason, today’s youth what we really care about is fun, do I look good am I impressing people that I know?. How your health is we don’t take it seriously until you see your health deteriorating at a speed that’s terrible

I: Uumh

P4: When they take the person to the clinic maybe the person will give in and say let us test and see what the problem is

I: Uumh ok. So do you think uumh maybe using those parties of young people could that be also, like maybe having HIV people plant themselves there do you think that creative idea could work as well?

P1: Number 1 [laughs] you know when we are drunk we lose 60% of our right brain we just think random things so I think maybe it will work

I: Uumh

P1: Yea I think it can work on these huge events and then a few people can test there. There are others that can go and see what’s happening what’s there.

1: Uumh

P1: Yea

I: Ok number 3

P3: ….

I: So number 4 you mentioned that, number 4 ok you mentioned that if there was some information on TV, radio that is being relayed to the youth that could encourage them to go get tested or something. What kind of message would you want those types of TV ads or radio ads to say that can encourage someone to come and get tested? Ok

P4: Number 4, when they maybe TVs they advertise that HIV is a huge disease that is affecting our youth so in order to save our youth we should everyone should know his or her status

I: Uumh

P4: Yes so it must always pop up after every advertisement in order to remind them like everyone knows your status.

I: Uumh

P4: Yes

I: Ok so there are HIV advertisements on TV right now, so you are saying they must be done after every advertisement then there will be like in your face all the time right. So number 2 you had your hand up you wanted to say something.

P2: Ok, number 2 even there is an episode or something like that like shows that showcase the person who is healthy and who is positive. It will encourage us to know our status because no one would want to be like the one already affected. So we will wanting to know that are we safe should we go take ARV’s maybe show another person who is taking ARV’s to show how ARV’s helped maybe I think it will encourage the youth to go and test

P4: Number 4 adding to what number 2 saying, yes our youth these days love soapies especially those ones with gangsters so if those soapies also encourage this thing of testing they must also have these part of people who are also affected act soapies. Our youth knew they don’t watch news nowadays they love these soapies of gangsterism so maybe if they put people with this disease one who is taking the ARV’s and one who doesn’t so that we can see that testing is very much important cause someone can end up being like that one on the TV yes they must also encourage those things on soapies as well.

I: Uumh

P4: Yes

P3: Number 4, number 3 I think number 4 is right yes. It has to come up with some story on TV or something

I: Uumh

P3: So that youth will watch it and learn something on it

I: Uumh, you mentioned maybe relaying maybe information by radio and TV. How else do you think we can get information across to the youth?

P5: Number 5 road shows

I: Uumh

P1: Number 1 social media

I: Uumh, what about social media?

P1: Like facebook, like most of us are on face book. Like face book it can have uuumh some sort of page that pops up there. Or your intagrams and twitter

I: Number 5 do you want to add to what number 1 has said? Number 4

P4: Number 4 yes she is right also social media is trending these nowadays so they also put those adverts there. So every youth whenever he or she gets online there must be an advertisement that pops up every time he or she logs in

I: Uumh

P4: Like yes that reminds them know your status

I: Uumh ok. So obviously social media you use it on your phone right tablets and what nots. What do you think the challenges will be for those that don’t have access to these things? Like We want people to know these type of HIV testing information broadcasting them on Facebook like she said number 1 integral and Twitter but there is that youth sitting there without access to a phone or social media how else do you think we can reach that person?

P2: I think oh! Number 2, I think these things works hand to hand if ever there are radio, TV and social media. So if ever I don’t have a phone I will have a radio if I don’t have a radio I will have a TV. So somehow I will get the message

I: Uumh

P5: Number 5 I think flyers and brochure will do because the flyers and brochures they give you, you can read them

I: Uumh

P5: If a you are interested in it that’s when that’s when I will do that thing. In those flyers and brochures will show the side effects of HIV and the symptom of it because the youth you find that they have the symptom but they don’t know it’s a symptom for what disease

I: Uumh

P5: Yes

I: Ok number 2 you wanted to say something

P2: Yes I just want to add something or disagree you see youth we don’t like reading, we don’t like books, we don’t like reading so if ever you come give me flyer I might get it from you just to throw it away without reading it. We are ignorant in reading so some might get the message some might at the flyers but most of the youth don’t like reading. We lack information just because we are not able to read.

I: Uumh

P2: Yes

I: Ok so you said that they don’t like reading?

P2: Uumh

I: So do you think if a message was to pop up on Facebook would they read that?

P2: Yes number 2 yeas cause we are always on Facebook if ever you log in on your Facebook the message will not wait for you to link in just pop up, you would want to know what is this on my phone then read and somehow you will get the message

P1: Number 1 uumh I want kind of disagree with what number 2 is saying that we don’t like reading

I: uumh

P1: just that we choose what we read, so number 5 mentioned that we mention symptoms there I mean if you get a brochure or a flyer or whatever on the road and I take it and I read it maybe I see a symptom maybe I have this and I am not sure what it is for that will push me to go and test and know what’s going on with me

I: Ok say we have a message written on Facebook, Instagram, Twitter the social media you guys have mentioned what would interest you? What would you want to see on Facebookthat will make you go and get tested? Would you want a post that says come get tested? How would you want give me an example? You log on to Facebook now and that message pops up what would that message be like that would make you want to get tested after seeing that message?

P2: Number 2 maybe a picture of an infected person. I think it will, I will not like to be that person so I will know what can I do to stop that from happening to me.

P1: Number 1 to add on to what number 2 said to me it also comes back to the symptoms I log into Facebookthere is a pop up shows up and then maybe it will have a link that will lead you there

I: Uumh

P1: Eye

I: Ok so you mentioned all these social media can be used on a phone what not. How else can we use a phone to spread HIV information to the youth?

P2: Number 2 I think sms, yea like the one who are advertising like sms can just get into your phone without you contacting them I think sms when it pops in your phone you would just need to know who is sending you an sms and you will get the message from there

I: Ok and then

P1: Number 1 sorry that she is saying you see when you receive a call me back message that irritating one at the bottom like the message its possible they add them there, instead of telling us about {XXX} (Name of a weekly South African TV Soapie) let it be about youth knowing their status so they can lead better lives better life

I: Uumh

P1: Yea

I: Ok so through smses and sms will be coming from clinic from department of uumh

P2: Number 2 I think come from department of health

I: Uumh ok and how often would you guys want get these types of messages?

P5: Number 5 more than 5 days like 5 a day yea that will be alright more than 5 even

I: Ok, do you agree with that number 4 or you want to add something you look like you have something to say?

P4: I want to add on number 5 more than 5 time cause when I get an sums 5 times it will irritate me and I just want to prevent it from happening ever again in my phone cause like these messages sms always coming in my phone sometimes it irritates more often

I: Uumh and I think it was number 2 that mentioned that on Facebook maybe there is a link or something is it number 1? Yes. Do you think you have to would want to have to use your own data to…

P1: No number 1 no

I: So you would want it to be free?

P1: I want it to be free it is sort of something educational so it should be free

I: Ok and what do you think the challenges of that will be?

P1: Uuumh yea that link? Who will fund that data? All that data we will use who will fund it?

I: Yea do you think data will also be an incentive?

P1: Uumh it should be

I: It should be what else?’

P1: It should be an incentive

I: What else?

P5: About data adding on data they must send messages and write to get 5mb of data you have to open this link and you will this data. Although some people will just open the link without reading

I: But then don’t you think that would be a problem then people would click on that link get their data and we would still not see them at the clinic. How do you think we can structure that so they do come and they get their data?

P5: Ok that one of getting data when you finish testing them gives you a voucher like data voucher. After testing they give you that voucher

I: So how much data? Give me an estimate

P5: Let’s say 30mb, I don’t have money to buy data I will be forced to go test to get data at the clinic

I: Uumh ok

P3: I agree with number 5 I think those data will make youth come and test and a lot of them like to chat and stuff. So I think data will make them come and test so they can get data watch those messages on face book and stuff and obviously there will be that one message from the department of health yes

I: Uumh ok and how often do you think this data should be maybe given once, every test or every time you come and get tested or

P2: Every after 6 month’s maybe. Number 2 I think data should be given every after 6 months even at the clinic they tell you if go testing they tell you to come after 6 months, every after 6 months you come then they should give you the data

I: Uumh ok interesting so you guys have mentioned a lot of incentives data, shirts, caps, give me an idea of like maybe like how would a cap look like something that would interest the youth to come and get tested. What would it look like would it just be a white shirt would it have something written? Like what give me an idea of what that thing would be that cap, give me an idea for what would look like paint a picture for me

P1: Number 1 a plain cap [laughs] it must be a plain cap, youth if I go and test at {XXX} (Name of a non-profit organization) and they give me a {XXX} (Name of a non-profit organization)cap I won’t wear it cause people are going to know I am coming from testing. Like I don’t want people to have that mentality personally I think our mentality is damaged like it’s not good like we are scared of what people will say, how will society look at me?

I: Ok

P1: Yea

I: Number 4 you seem to disagree with that

P4: Number 4 no I agree with cause yes the youth mentality is harming cause they will think that everyone will see that this person went testing cause of what is written on that cap so yes it must be plain so that if you want to write something on that cap let it be you writing something on that cap not us writing it cause it will show everyone that you went testing. The youth of now we are too much secretive like we don’t want everyone seeing that we went testing

I: Uumh

P4: Yea plain caps will be fine

I: Ok, number 2 you want to add something?

P2: I think that number 2 I think they must mix the one with the plain and one with a message cause I might come across of you but I don’t want to ask you or I don’t want talk to you but for the information that is there I get something that ok people are testing there so they got that cap from there so if ever it is plain it does not send any message to anyone it’s just for you. The meaning of the incentive is for the people to go and get like to give information to the others so they can go and get tested. So if ever they write something it might be ‘Be Aware of this disease’ it might be ‘Play safe’ just that for the message that I can go and get tested

I: Uumh

P2: Yes

I: Ok so say that I was going to, so we have 5 options or maybe the options of a shirt, cap, bottles, pen, food, bag and data voucher this is 7 things right. And then I ask each one of you to choose one and you have to explain to me why you would choose that 1 thing over the other 6. Or maybe just score them for me isn’t there are 7 right. So maybe you can say food is at the top of the list for you it is something that will encourage you to go get tested where else data doesn’t mean much to me or something like that. So tell me which one of these will be more important to you or would appeal to you to come and get tested out of the things that we have mentioned

P1: Number 1 data will be top of the list, as number 5 mentioned like its hard sometimes to get data if they give you that opportunity to get a data voucher why wouldn’t I go? I would go

I: Uumh. Do you agree with her number 3?

P3: Number 3 yes I agree with her data will make them to come and test

I: Ok, and what would be the second thing on your list

P3: Those pens

I: Pens?

P3: Uumh, what else

P5: Number 5 food [laughs]

I: Food, so that will be the first thing for you?

P5: Yes

I: Ok

P5: Number 5

I: Uumh

P5: Cause in some families they last ate the previous night and most mornings there is no food they only eat at night so if there is an opportunity that {XXX} (Name of a non-profit organization)they will test you and then give you food

I: Uumh, ok number 4

P4: Number 4 I will take the data voucher

I: Uumh why

P4: Cause to some families it’s too hard for them to just give you money to go and buy data

I: Ouch

P4: There is only money to buy something more important

I: Uumh

P4: That will benefit all of us in the family. So yes data will be number 1 then number 2 will be food

I: Ok

P4: Yes cause as families we are not the same, some it will be plenty of food than in the other family and others yes you eat once or twice a day. So when knowing that if I go I will just get free food so some will also encourage other youth to test there in order to get that food to eat, knowing that when I get home I am going to wait for the food that I am going to eat late

I: Uumh

P4: Yes it can also help

I: The consensus that I am getting from this is that data and food would be most important, it would be something that would bring the youth to come get tested

P1: I feel like caps, especially t-shirts, caps I see them but t-shirts are outdated they have been used for a long time

I: Uumh

P1: Like they have been there for years so we don’t want t-shirts no more

I: Uumh

P1: [laughs]

I: So say that I was to take data and food how do you think that would affect people and we still have other things on the list, what do you think will be the next thing that they choose that would be more important for them?

P5: Number 5 partying

I: How?

P5: Like after testing like at 5 there is an after party and after you test there is a thing that they will give to gain you entrance to the party, you know the youth likes things

I: Uumh ok. Can we go back to the issue of using cell phones or social media? So say that your parents know that you are on Facebook receiving these type of HIV testing services how do you think they will feel? How will they react to it knowing that you get these types of HIV testing information is it something they would be happy about, would it be something they will angry about tell me?

P2: Number 2 I think they won’t mind cause whether we like it or not HIV is there and will always be there so when a child gets information or doesn’t get information he/ she might be infected so it is safer or maybe they must have their own adverts so that they should encourage their children to go testing. So that even though you know that they get, we this information they must see it as a helpful thing not something we should not know about

I: Uumh

P2: Yes

P4: Number 4 I think parents seeing those messages on child’s phone will also pressurise them to encourage children of theirs to go to test yea. So me as a mother seeing that there is this message popping up on my child’s phone meaning that this message is important so I must also put something adding to that message. So it will also encourage parents to encourage their children to go test

I: Uumh do you agree number 3?

P3: Yes I agree with them

I: You seem so quiet do you want to add something to what she said? Agree or disagree? What is it that you agree with?

P3: When parents see those messages popping on their phone yes I agree her some parents will react badly some other parents just don’t tell their kids to go test. And as I know that even me I have parents there is no one there that has told me to go and test and I know it’s not only me, I know a lot of people don’t tell their children to go and tell their children to go and test

I: Uumh

P3: Yea

I: Ok number 5 you want to say something about, how would your parents feel if you were to receive those HIV testing series from your phone social media platforms we talked about?

P1: Number 1

I: Number 1 yes

P1: As people our parents are not the same like parents of child number 1 they won’t understand why their child is receiving those types of messages from Facebook or whatever social media you use. And other parents would be happy and actually encourage them to go and get tested

I: Uumh

P1: Uumh

I: Ok thank you guy so can you, you have given us a lot of suggestions that may help us encourage the youth to speak more honestly about HIV testing services right. Is there any other suggestion you might have not have thought of a few minutes earlier? Mmh?

P1: Number 1 uumh you see these that test on the street in tents

I: Uumh

P1: I think they must like come up with something more creative to youth besides using caps, t-shirts, bottles and school bags. Ok a school bag can attract people, I mean I want a bag but not a plain bag something a bit fancy that will be attractive so it can be noticeable and people can ask where you got the bag, hence also come test. Like sometimes they must add something maybe they can, now it’s almost summer we heading for summer maybe they must have sandals for people and give them after testing you get sandals. If you like fun they can maybe give you speaker’s things like that more creative unlike caps

I: Uumh you have added actually two more incentives to the list sandals, speakers. Ok any other thing?

P1: I will think about it [laughs]

I: And you mentioned that parents aren’t the same right

P1: Yea

I: How do you think we can encourage them to speak to their kids that they become more honest about their sexual behaviours?

P1: How you can encourage parents

I: Uumh

P1: Yho in some cases it doesn’t work

I: Mmmh, how do you think we can do to encourage youth then to be more open and speak more honestly about their sexual behaviour?

P1: Who do we open up to? Even if I get tested I am keeping that to myself I am not telling the next one that I tested, sometimes there is a chance that the results are positive who do I talk to you understand? Especially if your background is rough like you have strict parents and you can’t talk to them about anything they have that mentality that we don’t talk about such things. Even with friends it’s difficult to tell them friend this is my status and understands. You will also have that mentality that everyone probably knows my status

I: Uumh

P1: After a positive result people change. Someone who was often bubbly and talkative they all of a sudden become quiet

I: Uumh

P1: Unless one gets counselling, the person that tested you can as you how are you doing mentally

I: Yummy ok

P1: Uumh

I: Number 4 how would you encourage the youth to speak more about their sexual behaviours?

P4: Number 4

I: Uumh

P4: …Uumh what number 1 said yes most of us our background is not right yes, maybe if we have some counsellors talking to us about how to prevent or how to be when you have this disease or how to encourage our kids to go testing. Yea so maybe the counsellors

I: Uumh

P4: Yea maybe they will increase at the clinics and on the tents maybe they can also go to schools these counsellors if you get that you are positive

I: Ok

P3: Number 3 I think counselling will be a good option or maybe if indoors like the go to people’s houses to counsel them

I: What else?

P2: Number 2 if after testing if they ask you who you want to share with, I might want to my mother but I am afraid to approach her, so if ever there is someone present while I tell her my results it might be easy so they might ask gore who you would want to share your results with

I: Uumh

P2: So if ever I am saying my mother not necessarily my house cause it might be expensive maybe we are travelling. So if ever I am there at the consultation room my mother will be on loud speaker the consultant tell my mother about my result it might be easy for me to go home and open up again

I: Uumh

P: Yes

I: Do you guys have any final thoughts about the youth and HIV testing?

P1: Number 1 yes, I think the youth of today we need to be educated more and told that HIV it doesn’t kill according to me it does not kill but what kills is the mentality one wakes up in the morning and remembers that they are sick and during day I have that mentality that I am sick don’t do that people need to know that HIV doesn’t kill, if you test and you find your results positive attend counselling

I: Uumh

P1: If you attend counselling you will ease that mentality cause youth once they do a test and the results come positive they think them dying. No you are not dying no you just have a disease that you can live with and keep controlled and levelled going forward we need to be educated more about that

I: Ok

P1: Uumh

I: Ok any final thoughts number 5 about youth maybe?

P4: Number 4 about those professionals going to schools

I: Uumh

P4: They must always go to schools because our youth is in schools so if they go to schools twice in two months to remind the guys it is important for you to go testing

I: Uumh

P4: Yea so they must go to schools, they must go to schools cause if they say there is a youth meeting some won’t come but all kids will be at the schools

I: Ok number 3 give me your final thoughts on incentives that we have mentioned, HIV testing services, youth. Number 2

P2: Ok number 2 I think when going to schools maybe they will have minimal time like I might be at school and not get the message the message the people were here for. So they must be at school and also have tents outside the school. So if ever I need more information they can get it outside that would be more informative

I: Ok we are almost at the end of our discussion is there anything you guys want to say before we close off? Anything? Number 4 you look like you have something on your mind

P4: Number 4 yes this thing of contacting professional people to go to schools is very much serious if they take someone professional well known to go to schools. They mustn’t take someone from the community cause youth is disrespectful why should I listen to this person yes

I: Who would that person be?

P5: Maybe {XXX} (Name of a leader of a political organization) [laughs]

I: Why him? Do you think he is influential when it comes to the youth?

P5: Yes, if you go to one of his rallies at a stadium in Seshego Limpopo you find that most of the people there will be youths

I: Number 1 you seem to agree with that you are shaking nodding your head

P1: I agree that guy is very influential when it comes to the youth. If he is to arrange the youth and say let us all go to test this is what you get will believe me we are all going

I: Uumh

P1: Yes

I: What would {XXX} (Name of a leader of a political organization) be providing there give me an idea, like an incentive that he give so that the youth can come?

P5: Food parcels, clothes, sanitary pads

I: What else? Number 1 you look like you have something to say

P1: I am just trying to imagine say {XXX} (Name of a leader of a political organization) saying to all the youth that came to get tested majority of us want to go back to school but due to financial reasons and other factors, and he says out of all of you I will pick 10 and give you bursaries and go to school, that’s where we are going to get tested so that we are able to have a better life too

I: Ok thank you this was very informative and I would just like to thank you guys for your participation all your inputs have been noted and I just want to talk you again for participating in the, this focus group discussion we are actually at the end of our focus group discussion. The time is 13: 56 thank you guys

End time: 13: 56
