# Supplementary material for: Exploring perceptions of low risk behaviour and drivers to test for HIV among South African youth
Source: PLoS One. 2021 Jan 22;16(1):e0245542. doi: 10.1371/journal.pone.0245542 (PMC7822253; doi:10.1371/journal.pone.0245542)
Supplement: S1 File — (ZIP) [file pone.0245542.s001.zip › S1_File_Anonymised Transcripts/FGD02_Males (18-24 year olds) Translation_QC_TM.docx]

Full Participant ID: FGD02_Males (18-24 year olds)

Participant Type: Focus group discussion

Location: Dayeton

Date: 7 September 2018

Start time: 10: 45

Primary interview language:

Name of Facilitator/Interviewer: Wellington Maruma

Name of Note Taker:

Name of Transcriber: Ornate Masuku

Length of recording: 1:22:28

Label Key

I = Interviewer

P = Participant

N = Notetaker

{ } = Indicates that details were changed or pseudonyms were used to anonymise data

xxx = words were omitted to anonymise data

- = breaking into a sentence by the next speaker

… = pause or drawn out words

[ ] = indicates noise made, e.g. [laugh], [sigh], [pause]

[inaudible segment] = Unclear section of the recording

?Mulenga Clinic?, ?P3? = questionable text or doubt as to what was said or who said it

I: Focus group discussion uumh the date is the 7^th^ of September 2018, at {XXX} (name of interview location) with five male participants aged 18-24 with HIV testing experience. Their unique identifier numbers are 6, 7, 8, 9 and 10 and the time is 10: 45.This session is being facilitated by {XXX} (interviewer name)and the scribe is {XXX} scribe name). Thank you guys for allowing me to for participating in this focus group discussion, do you allow me to record this session?

P: Yea

P10: Number 10 yes I agree

P7: Number 7 yes I agree

P6: Number 6 I agree

P8: Number 8 I agree

P9: Number 9

I: Thank you guys. Ok the reason why we are here is to, we are just going to have a discussion right ok! Ok so you just tell me what you guys think about what HIV is

P9: Number 9, I think HIV is a disease that can be curable but can be prevented

I: Ummh

P9: Yea that’s all I can say

P7: Number 7, I think HIV its, HIV differs from AIDS, HIV is still a virus which is the human immune virus, I agree with number 9 it can be curable and prevented

P6: Number 6, uumh I think HIV is a disease that lives among humanbeings and it can also like number 7 and 9 said it can be cured but now it seems no cure can be found because eemh the services that in our country there is a lack of service I can say. Because if there If our country so eemh delivering service we could have had that cure for HIV

I: Ummh. Number 10 you want to say something?

P10: Number 10, I think eeemh the cure for HIV they have it but they don’t want to take it out now cause they think the state of the country will decrease, the more too much of trading of ARV’s and there will be less of people who be living with HIV I think

I: Ok, number 8

P8: Number 8, eemh I agree with number 10 …because I think eish

I: Ok it’s fine

P8: Ok I agree with number 10 ok, but I think there is a cure for HIV

I: Ummh

P8: But only rich people have access to it (the cure of HIV)

I: Ok, number 6 isn’t you mentioned that the lack of services is that what you experienced when you went for HIV testing?

P6: Yea I experienced it in terms of service like, as number 6 eemh I am saying I experienced it cause sometimes when you are going to have a test for HIV test, what should they do is that they should provide some sort of eemh maybe counselling before and after you are tested actually they should counsel you but now sometimes you can find that they take you to a room and test you for HIV without telling you what the processes will be using or anything that they will be using materials and stuff like that

I: Ummh

P7: Number 7, personally I agree with number 6 I think the services they are giving us its poor, as he said name I think before you go and test, there must be some sort of counselling or someone who will talk because in most cases here in the townships we find that maybe there's a tent, maybe they have pitched a tent and they are convincing us to come and test. Maybe convincing you to just come and test maybe they are looking for statistics and stuff. It shouldn’t be like that as number 6 has said maybe something like you can first start by sitting down and think thoroughly because knowing your status it’s important.

P10: Number 10, I agree with number 7 because most of the time it’s hard to know that you are HIV positive. Maybe like the way they have called you to say come and test at those tents you see maybe you are rushing somewhere to do something so when you find out that you are HIV positive like some people will kill themselves like that kind of stuff yea

I: Ummh

P9: Number 9, I agree with number 10 people should be prepared when they are taking a test, don’t rush things they must first think about then do the test and be convinced that they want to do the test

I: Ummh

P9: Not just when you are going somewhere just like number 10 said

I: Ummh ok, so you guys have mentioned the lack of service as one of the HIV testing services right

P?: Yes

I: Can you guys take me through the other negative experiences you guys have had with being tested for HIV. …Any other negative?

P9: Well number 9 the negative that I had, my experience when I first got tested, I don’t think that they were private with my information because I was tested at the reception and all the people were busy I didn’t have that privacy of

I: Ummh

P9: And even when they told me there were people behind me

I: Ummh number 7 wants to add something to that

P7: Number 7 name I agree with number 9 maybe it mustn’t maybe the room for testing must not be in public and we are able to see who enters the room first. It can be you are going to test and people can bad mouth you and say why would you go test for HIV blah blah cause us as black people we are scared in most cases of HIV. So I think the negative part, sometimes is that this testing happens in public it must be something confidential and private for a person

P8: Number 8, I agree with number 7 and 10 I think when like how can I put this like peoples’ information must be private from other people cause most of the times here at the clinic, I don’t think that as number 10 said we don’t get eemh what’s that again

I: Privacy

P8: Privacy, yea

I: Number 6 you want to tell us something?

P6: Number 6, I would like to add to what my fellow number 7 just said. In most cases you find that those people you are dealing with those that are going to test you. Sometimes they are not professional in that eemh if you are going to maybe test someone there is a procedure you have to follow before going to a room to test someone. Maybe gloves to protect themselves or maybe use, in some cases some nurses or doctors whoever is going to test you using the same needles, you find things like that

I: Uummh

P6: And also some other people will be like just injecting you maybe in a wrong way and you feel some sort of pain if they inject you. So it seems like they don’t care about anything or about you

I: Uummh

P6: Yes

I: Number 7 you are nodding your head, do you want to add something

P7: Number 7 I just agree with number 6

I: Ok, number 10; you have your hand raised up

P10: No its fine

I: Ok, we have spoken about the negatives right. Have you, are there any positives that you guys have experienced, like when you guys went to get tested what was good thing about it?

P9: The positive is that, Oh! Number 9 the positive is that a person gets what they want like come and get tested, get the service and his result

I: Ummh

P9: And the information given by the tester is always correct because they hand in the information first, and then they show the test results

I: Ummh

P?: Yea the positive thing eemh I have experienced is that after that they give you some emh emh protection so that you play it safe, that’s what I like about it

I: Ummh, number 7

P7: Number 7 I agree with number 6, the positive thing is sometimes that when you finish testing you gain information yea you see, the important part is that you gain some information; information is what kills us in most cases, like the lack of information

I: Ummh

P9: I would like to add to number 7, Oh! Number 9 I would like to add to number 7 the other positive they do is that they hand in pamphlets, so that people can even show their friends as evidence that there is something done by the HIV virus

I: Uumh, number 10

P10: Number 10, I agree with number 6 as he was saying after you get tested they give you some protection so you can protect yourself when you go under sexual intercourses, so you can protect yourself do that, yea

I: Ok, so we have heard the negatives and the positives you guys have experienced at these clinics right. Can you maybe give me suggestions; I need a suggestion from each one of you of how we can improve HIV testing services especially amongst people your age groups you know. What do you think can be done in the clinics in order to improve the negatives you guys have had?

P8: Eemh number 8, I think they must get, what’s that called. A professional person that knows their work in order to how can I put this …in order to decrease the number of complaints about the clinic and uumh

I: Number 10 you have your hand raised up

P10: Number 10, they should do private room when they test you, cause some other times other times other people they do hear these results that you are HIV positive, they tease you when you are going in the streets those kind of stuffs yea

I: Number 7

P7: Number 7, I heard number 8 saying that they have to get professionals ok, I agree with you have to be professional but besides being professional maybe the aim is that they must hire someone dedicated not someone who’s aim is to go to work but like the aim is to serve people, like they must hire someone that understands people as number 10 has said also someone’s privacy its very important they have to make private rooms where, besides we must be tested by people we don’t know because we can’t be tested by someone we know you see. We must be tested maybe by someone you don’t know, yea that could work

I: Ummh number 8 you want to add onto what number 7 said?

P8: Yea, eemh I think they must get people that are passionate not someone who works just because they want a pay check at the end of the month and they must employ someone who loves the job and is patient with people

I: Ummh

P8: Yea

I: Ok, so uumh so you as, I want to hear from each one of you as a 18-24 age group, how would you want to receive information about HIV testing and treatment? Do you want to get it via pamphlets as number 9 said you know they issue pamphlets is there any other way you would like to receive information about HIV testing services? Number 7 you have your hand raised up.

P7: Eish as number 7 we all know that people our age group when it comes to pamphlets eish most of us are scared to take these pamphlets maybe about HIV or circumcision whatsoever or things like that we feel like its private or we are scared of other people. So I think eemh maybe the best part sometimes we can use phones maybe social media and stuff to receive information about HIV and whatsoever.

I: Ummh

P7: Than to be given pamphlets because some of us don’t know how to read you see, they don’t want to read you see

I: Ummh

P7: People are often holding their phones but pamphlets you can offer to give them pamphlets and they can pass without looking at you or what you are doing

I: Ummh

P7: And what I have noticed is that HIV, HIV affects mostly unemployed people maybe it can happen that the government maybe hires people to run programmes like this one as a session and we talk as the youth about HIV. Talk about it and stuff like that maybe that would work, maybe there is something number 9 knows that I don’t know I can get information from him

I: Number 9 do you think using like number 7 suggestion to use social media, do you think that’s eemh something that would work?

P9: Yea I think that is something that would work. Oh! Number 9, I think it would work because every youth these days has a phone and can easily access information via face book, whatsapp, internet and other social media

I: Ummh

P9: Yea it would work really

I: Number 6

P6: Number 6, I agree with the guys because nowadays our lives are run by technology, but now when you look at the technology that we have, most cell phones tablets e.t.c. they need data. So you have to buy data where do you get that money like now you are not working so at some point it’s a disadvantage you see. But now pamphlets play a big role because now let’s say you don’t have network you can get that information from the pamphlet

I: Ummh. Number 7

P7: Number 7, as number 6 has said his point is valid. I still think this issue of pamphlets is simple or whatever but I still think I social media the government should maybe pay for something maybe create an app for free on social media that when a person wants information about HIV they can get it for free without using data or what. Our government must implement this type of thing

I: Ummh

P6: Number 6, I agree with number 7 but now for people who do not have cell phones, how will they get this information? It’s a question we have to ask ourselves at some point

I: Uummh [laughs] that was my next question. Number 9 you want to answer that?

P9: I think I can answer that because those who don’t use cell phones they read news papers and magazines

I: Ummh, number 10 do you agree?

P10: Yes, I agree with number 9, maybe you can get information from a friend maybe when I am playing social media I can share that they are talking about HIV and those kinds of stuff

I: Ummh

P10: Ummh you can get information

I: Number 6, you look like you have something on your mind

P6: I’m fine now number 6

I: Ok, number 6 you actually touched on something like not having a phone or access to data, do you think that’s something that would incentivize you to come and get tested to receive information on your phone, maybe if data was provided for you to access information?...Like if we were to give you data or some people give you data to access HIV testing information on your phone. Do you think that is something that would encourage you personally to come for HIV testing if you do not know your HIV status?

P6: Number 6, I don’t think so because if I receive that data

I: Ummh

P6: I will use it for something else because I will say no man! Maybe for instance talk to us and say come and test tomorrow we will give you some maybe 10 megabytes daily, I will be like ok I will come but I have to receive that megabytes first. After I have just received it I will say this guy is trouble I will not go there, again tomorrow it will be the same thing but data and stuff like that will not work for me.

I: Ummh, do you have an idea of what will work for you personally, like what would make you come and get tested for HIV?

P6: Number 6, if I may ask may I speak for males or females

I: Both anything is fine

P6: Ok let me go first then for the females when you look at rural areas

I: Ummh

P6: Rural areas there is a lack of employment and stuff, access to services and stuff like that so they don’t have the necessary things that are fundamental to their personal wellbeing so maybe like in some sort of rural areas maybe for females maybe you can give them sanitary pads or stuff like that maybe they will be encouraged to come and get tested because they will get those things because they don’t have them by that time

I: Ummh

P6: So as for males it will be like eemh eish most like maybe if you can get some motivational speakers they could play that trick

I: Ummh and number 7 you were nodding your head when number 6 mentioned sanitary pads

P7: Emmh number 7, I agree with number 6 as he said if we look at rural areas there are unemployed people besides the ladies if maybe those who go test people at the rural areas go with things people need in the rural areas for woman pads, and also condoms they must go with condoms cause HIV it spreads rapidly in the rural areas and stuff. And they also need information, even information can be an incentive to them they can come plus in rural areas we know there is kings and chiefs so you can talk to one of them to gather people there is I respect there and maybe you can do your testing and stuff and stuff. And everyone be satisfied knowing their status even at schools of these rural areas go there and do testing and stuff to those who are comfortable.

I: Ummh, ok so you mentioned motivational speaker’s number 6 rights, so give me an idea like in your mind that would this person be? Like whom would you want to spoken to?

P7: Number 7, maybe a motivational speaker can be a pastor or maybe a doctor as someone who knows, like they can motivate in relation to HIV because he has information about HIV so yea. A pastor he’s everywhere

P6: Yea no, number 6 I would just like to add on number 7 has just said like a motivational speaker can be someone, maybe a well known person who has lived with HIV since birth or what. Because if that person talks to us we will be encouraged to go test cause no man, this thing does not kill only take your medication and live a healthy life. So what is the use of not knowing your status where else you have those people that want to help you to know your status

I: Ummh, ok that’s good. So with, you guys have mentioned a few is it number 7 that mentioned the word incentives

P7: Yea

I: I just want to get an idea of what you think incentives is number 7

P7: Number 7 I think an incentive is something that can change your behaviour or something to trigger your attention or something that can attract you to do something

I: Ummh

P7: Yea

I: Ok and you guys have mentioned a few of those, number 6 said data but you guys said that wouldn’t work, number7 mentioned condoms, sanitary pads, the use of motivational speakers. Is there anything else you guys have in mind, something that you think would work for your age group something that would encourage people to come and get tested?

P6: Number 6

I: Ummh

P6: I think again like eemh like entertainment or mobile entertainment like you see those trucks where they use music and art and stuff like that they can also, if you find that maybe at the school or somewhere rural. Because rural is not much developed so entertainment for those people maybe they can see that we are being acknowledged or being taken care of so they also have to play the part

I: Ummh

P6: Because people are bringing things to us so maybe also we have to go meet them half way like stuff like that maybe there is a play in that mobile entertainment about HIV and stuff like that how could we prevent yea can also

I: Ok, yea number 9 you have your hand raised

P9: Number 9 I think that if we get someone aged 18-25 who has got HIV and can explain to people our age group, I think people who are aged 18-24, and 25 higher will be able to relate to that person because they are the same age as them

I: Ummh, number 7

P7: Number 7, I heard number 6 talking about mobile entertainment you see. Ok on that point I can disagree with him a bit, still you can have mobile entertainment and people can just pass so I think it will depend on your ability to entertain us but what will you give us because it doesn’t help for us to just watch but what else do we get after watching. Maybe they can be singing and we just pass unless maybe they sing and after that maybe they give us some caps, t-shirts at least. T-shirts can be written something like “Fight HIV” or those types of stuff yea maybe that one could work

I: Ummh, and you think these caps and t-shirts would encourage you to come and get tested, if like at these entertainment mobile trucks

P7: Yea, yes I like the point of a play because it’s very valid. And I play will be something touching, if you watch a play based on HIV yea like do dramas based on HIV, but they must not be discouraging but encourage us

I: Ummh

P7: Because in most things we do we exclude people living with HIV and stuff but there is a need that we do things that include them so that they are not discriminated against they must not be criticized

I: Ummh, number 8 you’ve been quiet

P8: Number 8, I agree with number 7 I think it’s better that we include HIV positive people so that they can give us a better experience and tell us how should you live when one has HIV. What are the things one must and mustn’t do things like that?

I: Ummh, number 7

P7: Yea number 7, I agree no number 8, the main problem about going to get tested is not that you are scared, of the fact that you might be positive. But I’m scared of what happens after I test and I am HIV positive like what will I do next. But looking at how maybe people with HIV are treated can be that something that encourages one to go and tested maybe I can also learn how not to discriminate people living with HIV

I: Ummh

P7: Because the fear it’s not I have HIV but its being scared that after I know my status what will I do you see

I: Ummh, yea you will be stigmatized.

P7: Yea things like that

I: Number 6 you have your hand raised up, do you agree with him or you want to add on to his point

P6: Emmh number 6 I would like to add on to both number 7 and 8 to their points because after we have talked about the good things that we have received after testing you see. The good thing about testing

I: Ummh

P6: As number 7 said more people didn’t have information or knowledge about HIV, and also about their health because when you look at their health after you have received your HIV status maybe you will get a counsellor maybe they will just tell you how to live your life in a better way a healthy lifestyle because sometimes most people can, if you are an HIV activist maybe you can have something like maybe you can start your own vegetable garden and can live a healthy lifestyle because you don’t have that money to buy those vegetables so they can help you somewhere somehow to grow your stuff

I: Ummh, number 7

P7: Number 7 I agree with number 6 because what kills many people is not HIV per se but defaulting treatment you see and in most cases people default cause they don’t have food, so maybe as he said seeds are not expensive maybe if people with HIV the government can provide in clinics when they come get pills they get seeds and she will be encouraged because some people can get seeds and sell them you see. They should be encouraged or after give them seeds and stuff. HIV in most cases is not among the rich in most cases it’s among us the unemployed people and poor people. So maybe the government trains them for a place these people gather them together and they plant the seeds, like if a lot of us have HIV and we see each other and communicate and encourage that they buy them a place where they can meet and plant those seeds that they give them, this will help that they are not idle. Being idle makes, they think a lot about HIV and stuff but if they are kept busy it will help, and seeds are not expensive

I: Ummh, I actually like your idea of the seeds maybe seeds can also be an incentive for people to thingy so that they don’t default on their treatment right

P7: Yea

I: One of the reasons for defaulting treatment is the lack of food, so giving them food would encourage them to come get tested or maybe adhere to treatment yea ok. Anyone who has a different view to that? Or do number 9? Number 8 you want to say something?

P8: No

I: Mhhm?

P8: No

I: Ok what is it that you agree with that number 7 has just said?

P8: Yea I think the suggestion of food is right cause eish!

I: Uuumh

P8: Cause [clears throat] I don’t know what I can say but I think that suggestion is right

I: Ummh, number 10

P10: I think they must buy the youth a place where people with HIV can meet so that they encourage each other that they must not give up but continue planting like encourage each other that even with HIV one can live

P6: So number 6, I can speak in Setswana isn’t? So I am not sure if I am right or wrong so like number 7 has said that food can help a lot but sometimes the government must bring hampers or food parcels for people with no acess to get food like once a month.

P7: Number 7 ok like as number 6 said the government can bring out these hampers but as people we are different someone can take that hamper and sell it, so the main thing is not to feed them as I said they must however plant because someone can take that hamper then they see it and still default treatment.

P9: Number 9

I: Ummh number 9

P9: I think hamper will motivate people knowing that every time they come get medication

I: Ummh

P9: Yea it will motivate them

I: Number 6

P6: Number 6, so can I ask about the treatment of HIV is it one pill or many

I: Its one

P6: Ok

I: Ummh

P8: Number 8, just one or?

I: So it depends which regimen you are on but I think it’s one pill but I am not sure about that. So uummh can we go back to the, do you think cause you guys mentioned the idea of providing seeds right, number 6 mentioned food hamper. Do you think a feeding scheme would help as well to encourage people to not default their treatment like if they have acess to food?

P7: Number 7 actually I think feeding is the best than hampers because hampers like I said before they can sell it. Like someone can sell the hamper then still default on treatment, but a feeding scheme they can monitor them that for sure this person comes and they definitely eat. And the government must maybe hire people maybe to do door to door campaigns and encourage people with HIV to those feeding schemes or soup kitchen

I: Ummh, thank you guys for your suggestions so we have actually covered incentives like food hampers, feeding schemes, t-shirts, caps, condoms, entertainment, sanitary pads, ummh data right. So what I wanna find out from you is do you think there is any challenges we or the government might face in providing these incentives? I mean you have motioned one of them which is if we do give people hampers they might sell them, that’s one of the challenges we might face right with providing these types of incentives. So can you guys think of other challenges?

P9: Number 9 challenges some with HIV can come for their benefits just like someone can come and get that food hamper knowing they don’t have HIV that can be a biggest challenge

I: Number 7 you are nodding your head

P7: Number 7 I agree with number 9, people, the society is somehow especially when it comes to food and stuff so even people who don’t have HIV. Maybe then can come and demand food in a manner that is not right they can demand by force and say what is special about those that are receiving food that’s a challenge the government could face

I: Ok, number 10

P10: Number 10, the challenge most of the time the community can go there and criticize people with HIV that they get this food and they don’t you see things like that

I: Uuumh, [coughs] number 8 you want to add on?

P8: Ummh, yea I agree with them

I: What about what they said that you agree with?

P8: Yea number 10 I agree with him some people can come and criticize those living with HIV. That why is it that they receive special treatment yet we stay at the same place?

I: Ummh, maybe let’s take this incentive by incentive that you guys mentioned right. Let’s think of condoms, so I give you condoms and then you like as an incentive for you to come and get tested right. What are the challenges that we could face with those?

P?: I don’t think there will challenges

I: You don’t think there will be any challenges? Ok and the what about the issue of sanitary pads

P: I also don’t think there will be challenges because you decide for yourself do you want the pads or not. I don’t think there will be challenges on that.

I: Ummh

P: Because everyone can test, they know that by testing they will test and receive pads afterwards. If they don’t want the pads that’s that and no one forces you

I: Ummh, number 8 you wanted to add

P8: No, he has said what I wanted to say

I: Ok, so ummmh what about t-shirts, caps don’t you guys think like who’s going to be providing them isn’t that one of the challenges? How many, supply demands those types of things, just think maybe in that area

P7: Number 7, maybe there can be sponsor that will provide those t-shirt and caps but yes maybe the number being large can be a problem. People can even start fighting for these t-shirts and caps so that can be a challenge, some people might even end up hurt over these things. People are like that maybe test thirty people and they all get the t-shirts and the 31st person comes and tests and they are told the t-shirts and caps are finished, the person can feel some type of way that they didn’t get them. So it means the sponsor or whoever they must be strong financially so that they are able to provide as many t-shirts and caps

I: Ummh, ok but you know the t-shirt and cap thing has been done by a lot by HIV partner’s right. And it has some effect obviously but do you think that is something we should continue with or you want to add on another incentive that you are probably just thinking about now. Number 6

P6: Number 6, you can, have you ever seen eehm I have tested before like the last time I tested ok number 6 they gave us these USB sticks that looked like belts they were smart and decorated so those can do and a lot of times we have them and we are encouraged because you don’t have one so you go and test to get a USB

I: Ummh, and ok what else? Do you think that would work number 9?

P9: Yea I think it would work

I: Why do you think it would work?

P9: People love things and things bring people together in bulk

I: Ummh

P9: Yes

P10: Yea I think this thing of number 10, I think this thing of USB can be for other people for example like you can store information from the computer it can help

I: Ummh. Number 6 you had your hand raised, is that your hand raised up or?

P6: Number 6

I: Yes

P6: I was just I’m not sure if I am correct or wrong you see like most people they should be eemh reminded maybe for the motivational talk we spoke of people like the like of {XXX} (name of a late activist) maybe he should be an example that people should come and test and stuff like that

I: Ummh, number 7

P7: I agree with number 10, I agree with number like the whole of them who are speaking now. Mina I want to add that like stationery too maybe giving school going kids’ stationery maybe they can test and be given stationery like pens maybe and stuff like that could work maybe people in our age group they can go and test

I: For stationery?

P7: Yes

I: Stationery you mean like books?

P7: Yea like anything

I: Ok, uumh so we have spoken about the challenges and number 6 added to the incentives USB and stuff ok. Ok so I want to hear about your suggestions of how we can use social media to attract cause, is it number 6 that said that number 9 said that most youth have phones right

P9: Ummh

I: And they are on Facebook, Instagram, oh and number 7 also mentioned the use of phones and social media, I want us to touch on that a bit. So how do we use Facebook to get information out there about HIV testing and also encourage youth to come and get tested for HIV? Let’s start with Facebook

P9: Number 9 I think we can provide group chats that people can join and discuss the issue

I: Ummh

P6: Number 6, I agree with what number 9 said we can create a page about HIV yea

I: Ummh, and then what would this page be focusing on? Like

P6: Number 6, maybe like maybe challenges certain people are facing, challanges or information maybe information can be added

I: Ummh, number 10 you have your hand raised

P10: Number 10 I agree with number 6 I think the page can give information how to protect yourself to HIV/AIDS, how can you…

I: I’m listening I am listening

P10: How can you…

I: Ok, number 7

P7: Number 7, I agree with number and number 10 maybe there should be a Facebook page that will focus on the challenges of HIV or how can we prevent HIV you see so yea maybe it can encourage a lot of people

I: Ummh, and then instagram how do we use it?

P: I don’t even know it

I: Was it number 9 you said Facebook and Instagram. Take me through how you use Instagram

P9: I didn’t say Instagram I mentioned Whatsapp

I: Ok. How would you use Whatsapp?

P9: Whatsapp you can take peoples’ numbers by bulk ok, so that you oh! Aged 18-24 so that you can discuss the positives and the negatives of HIV

P: Yea it’s just like that

I: Number 8 you said

P8: And challenges

I: Challenges

P8: Sure

I: Ok

P6: Number 6, as number 9 has said like we go back on the issue of Facebook. On that page that is created on face book there can be peoples numbers for whatsapp, so these numbers can be visible so that whoever wants can create a group on Whatsapp. Sometimes people would want to speak in person so maybe we can make an arrangement and see face to face and talk cause talking helps a lot cause if you hold everything inside you at the end of the day it only kills you so Whatsapp can also help

P9: Number 9, I Whatsapp can help there are some people who cannot read or write in Whatsapp you can be able to record for those people so that they can understand

I: Ummh, as in like voice notes?

P9: Yea voice notes

I: Ok and then who will be sending out these voice notes? Is it like you or?

P9: Obvious the group admin must state the rules that for the sake of some people who lack they must record

P6: Number 6, I would like to say that media this whole thing also needs the media. Because for instance I know this old man who cannot see but can hear. So since he can hear we can use radios and they discuss issues like these, this can help him get information and can then spread the information to his grandchildren and children and stuff like that

I: Ummh, number 7

P7: Number 7, I agree with number 6 that you can put information on the media on radio even people who cannot read they can see but not read so if these issues are discussed via the radio they can hear and have information and a page on Facebook I think especially maybe the Whatsapp group must be specific and not just anyone should be part of the group. The group admin must not just be a person, but a person from a higher institution must be the admin so that it is not accessible to everyone the group must be locked

I: Ummh, and who would you want to be the admin?

P7: Eish I don’t know but maybe it can be a pastor or a person from a particular NGO organization or someone who will understand because there will be challenges discussed in the group so the person must be able to ensure there are no judgements and stuff in the group

I: Uuumh

P7: And it should not be accessible to just anyone though It’s on Facebook because someone might not have HIV and wants to be part of the group only to swear and disrespect people

I: Ummh

P7: That’s a challenge when it comes to groups and stuff, and this issue of groups obviously maybe if I create a group. Say I know this person in the group personally, what if I don’t know his status once I see him on the group now I know that they are living with HIV now there will be no privacy to their status

I: Ummh, number 8 you have been quiet

P8: …Ummh I like the idea number 7 said because some people just like to criticize people about their status you see so yea eish i don’t know what to say now I am out of words…

P6: Number 6, I would like to talk about NGO's because NGO's are often the ones that work extremely hard to help people living with HIV but they don’t have all the resources they need the government somewhere somehow can help maybe fund those NGO's so they can give people necessary services

I: Ummh, ok number 7

P7: Yea number 7, I agree about NGO’s they can go door to door spreading information even at schools the NGO's can go there also because eish our age group is killed by peer pressure, a person will listen more to what their friend says than what their parents say but if NGO’s play their role and things like {XXX} (name of a non-governmental organization)they can like these types of programmes the government does not really prioritize. Government doesn’t care about such programmes but the government must lend a hand promote in most cases. If they need funds they must fund them so that they are able to fight this disease called HIV .You can open maybe a soccer team for maybe people living with HIV. As I said at the beginning government must gather them some seeds and some others can maybe play soccer or whatever sport so that they are busy. They are living with HIV but they are kept busy with other things so they are not idle and think about things

I: Ummh

P7: Yea

I: You said something interesting that people in our age group they speak with their friends more than their parents about HIV and whats not right. So how do you think your parents each one of you have to answer how do you think your parents will feel if you were to receive HIV testing information on your cell phone? How do you think that they will take it? Like say that your parents don’t speak to you about it number 7 said that a lot of people speak to their friends more than their parents about HIV

P7: Number 7, eish it will depend on the parents as they differ, it doesn’t mean if I get information about HIV, I am HIV positive , I think they could implement a particular like a subject in school specifically to deal with chronic illness although it won’t just be HIV only But deal with chronic illness that subject it can work

I: Ummh so you said parents differ so

P7: Yea one parent can take it the wrong way and ask why you want to look at such information about HIV you see, but eish life one can make mistakes and HIV can infect just about anyone. It’s not like when you have HIV it means you are a fool or something, it’s something that can happen to anyone and you can get it in different ways so you don’t know where will you find yourself tomorrow. It’s a must we should have that information, government must deal with our parents. Maybe an NGO comes and explain it’s a must as youth we must know about I HIV you see.

I: Ummh, number 6 you have your hand raised

P6: Number 6 yea as number 7 said sometimes you see our parents get upset and the way it is other parents do not have knowledge of HIV. So they end up lets say maybe for instance you come to test and your results will be negative or positive so for you to talk about your results with your parents and you find that the parent has no knowledge of HIV or how HIV can be prevented or treated and at the end of the day t. When your parent makes noise then you feel like a bad person and then feel depressed and you tell yourself whats the use for living when my mom doesn’t love me. So this thing of lack of information as number 6 said cause there is clinical depression and the youth end up commiting suicide and do drugs. Just to feel better they forget about their problems. Mara immediately these things leave your system and you think but if your mother is educated she can support you

I: Ummh, number 8 please add on to what he said

P8: I didn’t hear him the phone was disturbing me

I: Number 10

P10: Number 10, I think what number 6 said that parents most of the time they need to teach their own kids and not to leave them out to the wolves but support them whatever their status is. They must like give them information you see so that they don’t turn to drugs and alcohol

I: Ummh, my question remember my question

P8: Ummh number 8 I think parents I agree with number 10 I think parents they should speak their kids about everything in order to keep them safe and know what they must and must not do. No one what tomorrow holds so the youth must be told everything. Personally I am scared to talk to my mother about certain things

I: What is the stuff you are scared to talk to your mother about?

P8: Yho! Like there are things I am only comfortable with talking to a man

I: Ummh, number 6

P6: Yea like number 6 like number 8 just said that he is scared to talk to his mother we could have programmes like brotherhood like man to man you see, maybe meet twice a week and then we talk about the issues we have. Cause it is a small group we know that whatever we discuss is kept private, unlike at home you can talk to your mother and the next thing you hear your issues being discussed by people who stay next door. So this thing of brotherhood man to man services will help

I: Ummh, number 7 do you want to add something?

P7: Number 7 like as number 6 said this thing starts from our parents we grew up and we are scared to talk about HIV because of our parents we don’t talk it’s something we are scared to talk about although it is something already there . It’s a must that we implement these men’s forum where we as men we can talk about challenges we face as men.

I: Ummh, ok so you guys have mentioned quite a lot of things that could encourage youth to get tested for HIV you suggested men’s forum which number 8 has mentioned and number 8 also said something about that he prefers men to talk to about maybe their sexual behaviours or any other stuff. So can you guys maybe give me another suggestion how we could youth to talk more openly about their sexual behaviours like an example number 8 gave was speaking to a male, a male person who would understand what you are going through. Can you guys give me other suggestions? Like how would you encourage youth to speak openly about their behaviours? So you have mentioned the brotherhood you could discuss in those kinds of forums

P6: Number 6, I don’t think, I don’t know if I am right or wrong but I think this takes us back to the NGO, in NGO's there are social worker practioners maybe one can feel free to talk to someone they don’t know and this profession prides its self in confidentiality

I: Ummh

P10: I think like number 10, I think that like as friends if we are close that much too much we can talk these kind of things how can HIV, to protect ourselves like

I: Ummh continue I am listening

P10: That how we can protect ourselves against HIV, if you are not able to talk to your parent you see, as young mrn we understand each other but talking with someone you trust that will never discuss your issues with other people.

I: Ummmh

P7: Maybe, number 7 maybe there be churches maybe where they can apply a man to man approach so that as a society you talk about effects of HIV

I: Ummh, ok so I will just go through all the suggestions that you guys have given us right to improve HIV testing services and then if you have any other thing that you, that I haven’t listed on the list you are welcome to add. Ok number 6 you wanna say something?

P6: Yea, to add onto what number 7 said, door to door programmes because now you find that at home people don’t have acess to clinics maybe it’s a distance, from here to the mall, so they don’t walk and go far. So the door to door programmes help cause now at least they bring the programmes to people, like they are here where residents are and that makes other people feel better and comfortable.

I: And do you think that would help if people come to your house to test you for HIV?

P6: It will help cause now I know that maybe I close the door even if I don’t close I am not scared that anyone might be looking at me. So it is better if the service is brought home.

I: Ummh, and you think that would also work for like treatment as well like they bring treatment at home?

P6: It will work

P7: Number 7, it will work because the point of door to door campaigns the nurses or whatsoever can come bringing information and if you are already infected giving you treatment at home you feel more comfortable. Someone maybe scared to be going to a clinic often to receive treatment c, at the clinic you see there is no privacy yea I think door to door campaigns will play a role like sometimes people it’s not like we don’t wanna do things like it’s not like I don’t wanna go and test but I am lazy and since the door to door people are already here let me just do it. Sometimes the clinic is far and maybe I am always busy if they get there and I am busy I can always spare 2 minutes of my time and they test and stuff

I: Ummh

P6: Number 6 we spoke about this thing number 9 just said that’s sometimes at the clinic there is no privacy like you can be tested in public therefore there must be rooms that are specially made for people who come to test. And there must also not be long queue cause they make people not come to test as they are impatient if they find a long lines they just go back. But if there are no long lines one knows that I go and test and I go straight back home. So If these door to door campaigns are there is no need for us to go to the clinics where there are long lines

I: Now these long que at the clinic is that a reason why people don’t come in and get tested?

P7: Number 7 besides the long queue for testing at the clinic it’s just full of people and people get discouraged, because its full and so I think door to door can work. Yea even at the church like the professionals at church so maybe allow a platform and people get tested at the church, these must be professional people because it’s a must that everything that is done is confidential

I: Uumh, we are almost at the end of our session I just wanna find out if you have any final thoughts on the youth, maybe accessing HIV treatment something you maybe forget to mention and maybe the use of these incentives like t-shirts, caps, food hampers, seeds, stationary, USB, uumh entertainment, condoms, data and all these other things, and sanitary pads. Thoughts on that?

P8: Number 8, ummh I think uumh a voucher will help cause the youth likes money

I: Ummh

P8: I think it will play a role

I: What kind of voucher would this be?

P8: Ummh a voucher to buy food, or just money

I: And how much would that be? Like just give me an amount. Like let’s say I am standing outside of the clinic and I have a voucher, if you come and get tested for HIV you would get this voucher, how much would that voucher be?

P8: From R50 to R100 ummh

I: Yea, ok ummh thank you guys any final thoughts

P6: Number 6, sometimes we must raise those sharp to the target group, target group cause that’s where the problem is they are too active sometimes they are sexually active and condoms are not available at schools, you find them getting busy at the school toilets There must be condoms at schools and parks

I: Ummh

P6: Yea

I: So you think providing condoms at schools is one of the suggestions

P6: Even if teachers don’t approve but these things must be easily available

I: Number 8 your hand was up

P8: Sorry, sorry, mistake

I: Number 9 you wanna add something you have been very quiet

P9: No

I: 10

P10: Yea I agree with number 6 as he was saying condoms must be made available in schools this will help avoid teenage pregnancy and HIV and other diseases

I: Ummh, number 7

P7: Circumcision we must not forget it too and circumcision and condom use reduces the risk of spreading or acquiring HIV especially from schools as he has said [giggles] they must encourage from schools the importance circumcision and condom use

I: Ummh, back to what number 6 has said about targeting schools and schools not having condoms and stuff. Do you think these students know how to use condoms or it is just about the lack of knowledge about the importance of HIV status or do you think there is something else missing there?

P6: Yea but before providing protection at schools they must be given information about how they are used for instance there can be an assembly only for giving information . We can even use taxi ranks, like most people use taxis so you can leave the pamphlets in the taxis and seeing them every day one can become curious and take the pamphlets and you look at it and you think I don’t know my status I should go and test.

I: Ummh, any final thoughts before we wrap up? Anything else you wanna add, number 6

P6: Number 6, At the clinic when people come and test for TB or flue they must talk to them about HIV and things like that, they must speak to them. Someone other time you find that you are escorting your mother or grandmother to the clinic and you know nothing about HIV but when you get to the clinic you are given some information

I: Ummh

P7: Yea even number 7 maybe like parks, soccer fields you can go there give them information

I: Ummh, lets wrap up

P6: Number 6 I am not sure if I am right or wrong but the point said by number 7 that government must sponsor sporting events netball, football, and tennis. Most people will be interested and be given information after that session and also get something and also be given information when they participate in these events at the end of the day they would have gotten information.

I: Ummh

P9: Number 9, I think there is an issue that we haven’t touched yet you see, we live in where most people use drugs like nyoape (a hingly addictive, illegal, destructive and street drug) and now it’s taken in different ways, some inject it. They inject it through they don’t know where this injection has been or who has used, they use it without information because they just want to use it. Even those people will need help.

I: Ummh, and how do you think we can help those high risk individuals

P9: Those I think they need to be talked to face to face because they don’t read newspapers or have phones

I: Do you agree with him, number 7

P7: Yes I agree with him because the nyaope (a hingly addictive, illegal, destructive and street drug) guys they need to maybe they can listen to someone or be at a particular place so I think it will be best as we spoke of NGO and professionals can do a campaign. Maybe when they pass by they can call them and tell relevant information like the dangers of these injections

I: Ummh, number 6

P6: Number 6, yea also prostitution plays a huge role because most of them don’t use protection and they are at work but it kills us as the society because a man can sleep with a prostitute and bring the disease at home and infect their partner

P7: Number 7, l agree with number 6 and prostitution like being a prostitute differs being a prostitute doesn’t mean you have to go to Springs hotel or, this thing of blessers maybe someone is dating an older I they don’t know they are HIV positive. Alcohol plays a role in the spread of HIV especially in taverns, so it I don’t know how we can deal with the alcohol issue. Even girls when they are drunk they cannot hold themselves and they become vulnerable to rape and worse they will be wearing short things. They way that, that express themselves through their dress codes contributes towards the HIV

I: Number 8

P8: He has said what I wanted to say

I: But what else do you wanna add on what he said you were listening right

P8: ….

I: Number 6 then we, is going to wrap up soon

P6: Number 6, you see our hospitals they don’t function well, like if you need blood you might find that they don’t have it. They also don’t do regular checks on the blood so somewhere somehow one might get blood of a positive person and end up being sick

P7: Number 7, I don’t agree they will not give you blood that positive

P6: Number 6, how come just recently they found a scissors in someone’s stomach like I think doctors just made a mistake

P7: That one it happens we have heard of such stories doctors were just not careful when it comes to blood they check the blood

I: Thank you guys [laughs] thank you guys for the discussion we have just had it was really helpful and enlightening and I learnt a lot from you guys. Especially the incentives that we were kind of looking at and also the use of social media, cell phones and how to interact with the youth with regard to HIV testing services, so the different suggestions that can be used to encourage the youth to be more honest about their sexual behaviour your men to men programmes or forums you have also mentioned places that we can target the youth like the taxi ranks, you have mentioned the sports grounds and you have also mentioned people that are using injectables and how we can reach those people an ummh yea. Thank you so much for being part of this focus group discussion and we are at the end of our discussion right now thanks you the time is 12:08 thank you guys so much

End Time:12: 08
