# Supplementary material for: Exploring perceptions of low risk behaviour and drivers to test for HIV among South African youth
Source: PLoS One. 2021 Jan 22;16(1):e0245542. doi: 10.1371/journal.pone.0245542 (PMC7822253; doi:10.1371/journal.pone.0245542)
Supplement: S1 File — (ZIP) [file pone.0245542.s001.zip › S1_File_Anonymised Transcripts/FGD03_Females (15-17year olds) Translation_QC_TM.docx]

Full Participant ID: FGD03_Females (15-17 years)

Participant Type: Focus Group Discussion

Location: Chief Albert Luthuli Clinic-Daveyton

Date: 08 October 2018

Start time:

Primary interview language: English and IsiZulu

Name of Facilitator/Interviewer: Wellington Maruma

Name of Note Taker: Bakang Mosime

Name of Transcriber: Ornate Masuku

Length of recording: 1:30:53

Label Key

I = Interviewer

P = Participant

N = Notetaker

{ } = Indicates that details were changed or pseudonyms were used to anonymise data

xxx = words were omitted to anonymise data

- = breaking into a sentence by the next speaker

… = pause or drawn out words

[ ] = indicates noise made, e.g. [laugh], [sigh], [pause]

[inaudible segment] = Unclear section of the recording

?Mulenga Clinic?, ?P3? = questionable text or doubt as to what was said or who said it

I: Focus group discussion with females aged 15-17 years old. The location {XXX} (name of interview location) and the date is the 8^th^ of October 2018. The primary language will be English and IsiZulu. The facilitator is {XXX} (interviewer name), the scribe is {XXX} scribe name). Ummh thank you guys for being part of this interview, this focus discussion group. Do you allow me to record this focus group discussion?

P14: Yes

P11: Yes

P15: Yes

P13: Yes

P12: Yes

I: Thank you guys, [giggles] relax. Ok so what I want to find out from you is what do you think HIV is?

P14: HIV is a virus because it can be cured but not exactly cured but it can be controlled

I: Number 11 you want to say something?

P11: Uumh I think that HIV it’s a virus which causes AIDS and other diseases. Ummh HIV cannot be cured but it can be suppressed actually, ummh yes that all I wanted to say

P15: Number 15

I: Ummh

P15: Me actually I think HIV is a venom that has killed so many people in the country, it can’t be cured but it can be controlled, eeemh eish yea

I: Ok, number 13

P13: As I can say HIV is caused by like if you have a partner and you don’t use protection and one of you or your partner has HIV, you can transmit it to another person. And if ever you use the treatment you can be healed

I: Ummh, number 11, I mean number 12

P12: I personally think that HIV is caused by like when someone is bleeding you see, and you don’t wear gloves, sometimes maybe you might have a small cut and you don’t notice it and then you can get HIV that way

I: Mmhhm ok. So I take it you guys have all tested for HIV or have experienced HIV testing. So what I am going to find out is what your experiences were when you went testing for HIV testing. What was it like? Is there anything you want to change maybe anything that you liked particularly?

P14: When we went testing it was like something, we were playing we weren’t really serious that we were going to test. We just thought that Oh! These people are testing let us also go then when we got there it was really scary because when our first friend came out she said “Yho! This thing is painful” and they also told us about the two lines, if there are two lines it means you are positive and one line means you are negative. We all said lets go back home what if you are positive and what if you are not so but then one of our friends told us that you just go there do the thing and if you trust yourself or if you really want to know your status then go and test no matter what is it. And then they will give you advise if you are HIV positive then go and get some treatment but then if you are not play it safe. Yea that’s what I want

I: Ummh, ok number 13 you want to say something? Number 11

P11: For me it was very scary, very hard, very depressing because I was sexually active and I was ummh I got actually in contact with unprotected sex so I was very nervous, I was like what if I am HIV positive and I am going to die. Ok back then I was, I actually didn’t have much information about it. I thought that when you have HIV you just die since I have this disease, I will live on medication. It was something like that but they educated me and told me that no ARV’s are there which can help suppress the viral load and be normal and all that.

I: Ummh, so you mentioned that you were scared. What were you scared of? You said it was depressing; you want to tell me more about that?

P11: Ummh yes yes I can tell you about that. Ok ummh I was scared because I thought that I was going to die actually, I am going to get sick cause like we often see stories of people with, they are depicted very thin and boney. I had those actual pictures in my head that yho! I am going to be like that

I: Ummh

P11: Yea

I: Ok, number 15 you want to tell us about your experience?

P15: Yea yea I was very scared, because I had already started having sex and I didn’t use protection and I also had a child, I was scared that if ever I am to be HIV positive and I die what will happen to my child and things like that.

I: Ummh, so you have a child?

P15: Yes

I: So you were scared that you might infect your baby?

P15: Yes

I: Ok and how did you deal with that? How did you overcome the fear?

P15: Aaah I just entered the place and the lady that was testing me was so friendly

I: Ummh

P15: Yea she was making jokes it was fun

I: Ummh, number 13

P13: Oh! Yea I can say that it is a scary thing cause of like you stay with a lot of people like in the area so when they tell you there is thing called HIV when you see someone who has a cut maybe by a glass you are scared to touch them because you think that you might also contract that thing HIV cause HIV is something that exists. So yea I also decided to come to the clinic and test, because there are a lot of people that I loved to help if they have cuts, like I would help and clean them up, so yea after that I came to the clinic to get tested. And I found out I didn’t have it.

I: Ummh, ok number 12

P12: When I first went to test I was scared, it’s a scary thing cause of like my mother had HIV, so that’s what motivated me to go and test so that I know whether I have it or not. Because when my mother died she had HIV, so that’s what motivated me to go since I didn’t know whether she had transmitted it to me or not.

I: Ummh ok. So ummh number 15, is it number 15? Yes. You mentioned that when you went for testing the lady from the clinic was friendly and made it nice. What other thing was positive about testing?

P15: Finding out that I am HIV positive.

I: Ummmh, what else?

P15: That I can adjust and move on with life

I: Ummh Ok. And do you guys have any negative experiences with maybe when you went for HIV testing?

P14: Ummh, like when people see you at the clinic the only thing they think is that you are HIV positive, you have AIDS or you are pregnant. So always when you go to the clinic you will be like “who is seeing me before I enter at the clinic? So that was the thing that most of us don’t want to be tested at the clinic. Cause the time when we tested they had come in those tents and were doing blood drives and all that stuff. So we were like cause we are not at the usual places lets go and get tested but it’s not like we really wanted to get tested. We were just being naughty we are young so we were curious as to what happens there and what they will tell us. So that’s the thing we tested but it’s not like we had intended to go and test we just wanted to go and see what the Sisters will say or do when we get there.

I: Mum

P14: Even when I entered the room the room I was like I am not here to test; I am just here to see. Then they were like if you are just here to watch sit and let’s play a game in this game if you lose we prick you with this thing but it won’t hurt. I was like ok, then we played a game played a game then I lost then they prickled me and took blood and tested it said ok its fine we played we played then I lost again then they looked at that thing that they put my blood on and told me my status, I asked if they had tested me they said yes. I was like I didn’t say that I want to test, they were like ok give us the phone numbers of your mother so we can call her and ask for permission to test you but they had already tested me by then. I was like its ok as long as I know my status its fine.

I: Ummh

P14: Ummh but then the game was good and I left. When I got outside I told my friends that we were playing a game there and there was food and everything you know everything is top you know. Then cause they are forward and they like things they went in too. But they were testing in there but they just use the game as a way not hurt you and not think a lot about what you are here to do and they use it as a way to calm you down and not think what if my result is positive.

I: Ummh, this game was it played by nurses or?

P14: Yes, like it’s played by nurses when they start they say if you lose we prick you with this thing if we lose you also get a chance to prick us. They made it by all means that they win and you lose

I: Where is this game played?

P14: No it’s like they will take cups then takes a small ball or something then spins them around, then you predict where the ball is. Then they say since you lost we will prick you

I: Then how old were you then?

P14: I was 15

I: 15?

P14: Yes

I: So there was no parental consent, like your parents had not agreed that you get tested?

P14: Yea my parents had not agreed but then when you enter they ask are you sure that you want to test? I was like yes I agree you can test me but as I said before I had not gone there to test honestly but I wanted to see what they will do to me.

I: Ummh ok you mentioned something that you don’t want; you would want to test at the clinic why not?

P14: Cause yho! Since you also see that this neighbourhood is a township and obviously as you turn at every corner there are people obviously the minute you enter the clinic people will start discussing you. They ask each other what is she doing at the clinic is she HIV positive or is she pregnant, these same people see you after a few months and you have either slimmed down or gained weight they conclude and say to each other “friend remember I said this person is sick” so you that’s the things

I: Ummh, do you also think the same way?

P11: Ummh number 11, yes I agree with her, the thing that makes me not want to test at the clinic is the treatment I received at the clinic before.

I: Tell me about how you were treated

P11: What I am about to share is confidential but then its fine yea, uumh I once had thrush around my vagina. So when I got here the Sister was like to me you are already sexually active? She’s like you are sexually active at this tender age? She was like yho! Go and test go and test. I thought that at the clinic you are tested by nurses but it’s just other people Just like here, they move you from here and say come into this room and you find someone friendly...So yea testing at the clinic its eish most nurses are rude, they are very rude.

I: Ummh

P11: Yes

P13: Ummh I agree with number 14 testing at the clinic at time are not right. Like at the clinic that’s here at the township when you enter everyone sees you and they instantly have negative thought. They conclude that you came because you are pregnant or here to fetch treatment or something like that, so usually I prefer going to a clinic that is far. Ok besides that sometimes nurses make you feel somehow like in such a way that you feel like that clinic is not ok. Like let’s say you came cause you were sick, when you are sick you are requested to go and test even though you did not come with the intention that you are here to test or they say start you on family planning like there is no discussion about it they kind of force you to do things that you do not want to do.

I: Mum

P11: Yea

I: And then how do you think we can deal with these things? How do you think we can improve these things? Number 15

P15: I think that often times they just hire anyone like number 15 eish! They just hire anyone. I think that a nursing job is not for everyone they must hire someone with the heart for it cause as I said as a teenage mother they treated me in a manner that was not nice at all when I would come to clinic they would be rude to me you see things like that rude. They would say things like “Like what do you want from boys when you are so young?” Things like that are depressing and you end up deciding not to go to the clinic anymore. Since I have a child I always have to go to the clinic for family planning so that I can maintain my life for the sake of my child

P13: As number 15 has said something’s are not right cause when she came to the clinic and she was discriminated in such a way that made her feel uncomfortable and not feel good. She could have ended up thinking of aborting the baby because of the way they were talking to her and making her uncomfortable when she would get to the clinic.

I: Ummh

P14: And some nurses are from the neighbourhood obviously they know you. So the minute you enter the clinic then you are pregnant, let’s say you are the first people to know you are pregnant and you haven’t told your mom or your friends the minute you enter the clinic and you get there and you say that you are pregnant and I need your advice . So they will be like actually why are you pregnant at this time? You are young those types of things, the thing of them treating you rude it’s not nice and most of the time at the clinic you must arrive there early at like 8 o’clock sometimes it gets to 4 o’clock without being helped and they tell you to come back tomorrow. You arrive at a specific time knowing that you need help like now or else what that is making me sick will kill me or maybe the pain of it won’t go away. The next thing they are telling you they are going on their tea break. They leave us there; some patients are extremely sick but they are not given attention.

I: Ummh, number 11 wants to add

P11: Sometimes the other thing that I see ok

I: Ummh

P11: That makes people scared neh, ummh sometimes when you get to the clinic and you find that you are HIV positive ok.

I: Yea

P11: When you come and get your treatment here, I have heard a nurse before saying those that are here to get their treatment must come this side. So some people would rather die inside that if anything is eating them it does so... I have heard a nurse say those that are here for treatment must come this side and those that are pregnant this side.

I: Ummh

P11: Yes, it’s this thing that at, happens at the clinic I think there should be cameras, I don’t know maybe the people from Ekurhuleni or something and they must look at how people are treated. Cause even though you put a complaint they remove them by themselves and throw them away. Cause there was once a nurse that I dealt with when I went for family planning she said that if you have a problem go and write it down and put it in the suggestion box, although she will make sure that she removes it and throws it away

I: Really?

P11: Yes

P14: When they leave here they often meet friends by the corner and tell them such a child is pregnant or this kid that is like this and this came to get ARV’s today. So the friend will also tell another person and it ends up everywhere in the community obviously and that’s not right

I: Ummh, number 12, so you mentioned that you heard that your mother passed away from HIV, did you get any sort of counselling or something after that?

P12: I got some sort of counselling ok, but some of the social workers that were assigned to me didn’t have time for me.I only got proper counselling this year and all along they were not telling me the truth they used to say that my mother died of diabetes and stuff. I only found that out early this year that my mother actually died of HIV. The social worker said she will come often to check up on me, and I should ask questions if there is anything that I do not understand and discuss how I feel about the whole situation. But I honestly I did not gain anything from counselling, they didn’t tell me things like don’t be depressed or give me any sort of

I: Ummh, ok and do you think ummh is there someone that you spoke to about this whole thing and how you really feel about it.

P12: I spoke to my sister

I: Ok

P12: Yea probably she is the one that I tell everything

I: Ok and you are getting help

P12: Yea they are helping me

I: Ok that’s good to hear. Ok guys back to the back to, so number 15 mentioned that some of the nurses are rude and how do you think we can manage that cause you said we can hire… what did you say? Can you repeat that again?

P15: I said that they must hire people that know how to deal with people, those that know how to speak with people in a polite manner and they have the heart to work with people not just work because they need a salary at the end of the month

I: Ummh

P15: Yea

I: Ok, number 15 you want to add onto that

P13: Yea I think they must find other people for nursing, because nursing is not a profession were you can be rude to the patients, you cannot treat them in a way that you are rude to them. Like you must be the type of person that has sympathy, a good heart and be able to withstand all the situations that you come across at the clinic or hospital. Even if someone comes in and they are stabbed they must help me isn’t this is what they studied for and maybe if I am pregnant and I come to the clinic wanting to talk to the nurse and after speaking to her she goes behind my back and tells people that this child has AIDS and shares with other people what I was there for. If I am HIV positive the whole community will know about it.

I: Ummh

P13: And which is not right.

I: Ummh, number 14

P14: Ummh I agree with number 13. Cause another thing that makes me not wants to come to the clinic if I am sick or something is wrong I would rather go to the Main clinic in {XXX} (name of a clinic). The reason why I don’t come to this clinic is always the nurses here, when you get here like once I came when I was writing a maths exam at 12 that day. I got here at 8 am told them that I had an exam by 12 they said ok we will help you before then and you go to school. After 11: 30 I went to them and said that I told you that I have an exam at 12 yet I still haven’t been helped or got attention from anyone, why haven’t I been attended to they were like we are on tea break we will see you when we come back, their tea break lasts an hour so often come back at 12 or 12:30 even then only one person returns. That one person that returns when you go there and say nurse I need help like right now, she will ask if you can’t see that she is busy wait for the others before you come in. So this whole thing feels like they don’t want to give us attention, they make us feel like we are not important. And the other thing is that some people will study nursing after that they will come work here they will start feeling like they want their jobs and that will hurt. If you do nursing then after that you come here and demand their jobs they will go around saying this child wants my job and this child I used to help when she was pregnant or came for treatment back then. All these things make one not want to come to the clinic

I: Ummh, so not being given attention?

P14: Yea not being given attention made me to miss my maths test. I left here and went to Main clinic in {XXX} (name of a clinic) and I told the nurse what had happed at the other clinic and that I was supposed to be actually at school writing a test. Then they made it by all means that they write a letter and phone the principal to say such a child was at the clinic that is why she could not write her test, please give her a chance that she writes it.

I: Ummh, but did you get the service you wanted at the other clinic?

P14: Yes at the Main clinic

I: Ok, number 11 you wanted to add on?

P11: Ummh

I: Ummh

P11: The way I see it

I: Ummh

P11: Nursing is a calling, just like teaching ok. Other people knew, I think that they studied nursing because back then it was only one year or two and the person would quickly get into the job market. Nursing is the type of profession that requires that even if you have your own problems you set them aside and focus on the patients. But often time’s nurses will take out their stresses and frustrations on patients. Everyone has their own stresses but they treat you anyhow and they don’t consider you.

I: Ummh

P11: Yea so like that is the kind of treatment we get at the clinic

I: Ummh, number 13

P13: Ummh, I agree with number 11 cause here at the clinic there are nurses that are known to treat you in the way she has mentioned. There is Sister that is well known at this clinic I won’t mention her name but she is very cheeky everyone knows that when you come to this clinic you will find her here is very unfriendly and she takes out her stresses on patients as number 11 has said. Another thing that makes people not come to the clinic is segregation like in front of everyone they say those that are here to collect their treatment come this side those that are pregnant this side. I think that they must give people appointment ticket instead, so when they come to the clinic they just say I am here for an appointment rather than being called like those that are here for pills come this side, it makes people uneasy and everyone concludes their status.

I: Ummh, number 12 you want to add onto what she just said?

P12: Yea I agree with number 13, another thing is that at the clinic they are very judgemental, when you leave they are already discussing that such a child is already using prevention pills, she is sleeping around they say such things...No yho! They say why such a child must prevent at such a young age. What I ask myself though is that what is their business in all of this cause their job is to offer a service what are they concerned with what we are doing.

I: Ummh

P12: That’s what I ask myself sometimes when I come to the clinic

I: So you guys mentioned a lot of negative things experiences that you guys have had with clinics right. Are there any positives that you might want to share number 11?

P11: I’m very asthmatic. A clinic that I can talk about that gave me great treatment is the one at {XXX} (name of a clinic). I went jogging with my friends one morning and I had an asthma attack, when I got there they immediately took me to the emergency room and I got oxygen and treatment. My sisters like my friends were treated very well they were not chased away but were given respect. And when we got there, there were people in the waiting room but when we got out they were gone which means like their service is quick.

I: Ummh

P11: Yea

I: Ok, number 13

P13: Ok the clinic that I can talk about that gave me good treatment its {XXX} (name of a clinic) clinic you are sure that whatever service you require you will get at the clinic. They are not like other clinic that tells you that they are out of stock on some medication and tell you to do a home remedy in the mean time. They make sure everyone is helped by the time they leave.

I: Ummh

P13: Yea

P14: The clinic I can talk about that gave me a good experience is the main clinic in {XXX} (name of a clinic), cause immediately when you enter obviously school going kids when we come we come to the clinic we are wearing school uniform cause maybe you come in the morning and from the clinic you are going to the school. School going kids when we come to the clinic we are wearing school uniform and you come in the morning they say all those that wearing school uniforms it doesn’t matter what you are here for come this side you will be helped first and since you need to go to school and then they call the elderly then everyone else. If you come in and are very sick they attend to you first. Obviously they take you to the emergency room and if you are bleeding and all that stuff they will also take you and attend to you and your problem.

I: Ummh

P14: And then even when they are giving you pills they ask about your overall health let’s say you came here because your eyes are sore. When I get there they say since your eyes are sore what else are you feeling on your body? Maybe you have a headache from time to time, or your say my feet get more often and sometimes stomach cramps and all of that. Then after you have mentioned these things they check what the real issue is with the body parts that you mentioned to be sore at times. Ok if they realise it is all not a real issue they let you go but if it is serious and they cannot offer you help you and call the ambulance to take you to the hospital they stabilize you.

I: Ummh, number 12 you had your hand raised up

P12: {XXX} (name of a clinic) clinic is the one that I found to be ok, they do not discriminate whether you are black or what, or you don’t know how to speak proper English like the people from the clinic in {XXX} (name of a clinic). There was once some Shona and stuff that I heard saying this one “she cannot speak properly, why is she slow?” things like that so {XXX} (name of a clinic) is the ok clinic that I went to.

I: Ummhh, so do you guys think that the things that you have mentioned the negative experiences that you guys have had, do you think those are the reasons why some like youth in your age group, do you think that is some of the reasons why some don’t come test for HIV? 13

P13: Ummh I think yea cause of in most clinics they are like negative about everything, so you will keep saying and asking yourself what if I have that thing? You become scared and some even lose the strength to come and test.

I: Ummh

P14: Ummh, and the other thing is when you get to the clinic, the minute you enter the clinic obviously if the nurse, if someone knows you even if you are not necessarily going to their room to let’s say I mean you are not even testing for HIV in their room. But he or she will make it by all means that they go to the receptionist and ask what is this child doing at the clinic? Then they will say you are here to test and before you leave the treating room, they would have told everyone and assumed that you have HIV.

I: Ummh

P14: Or which means there is something wrong with you. Or the others will just assume the worst and conclude that you did something yet you didn’t and immediately when you come from the testing room they will say “I have always said that this child has HIV, just that I did not want to say this child has HIV but now I know for sure she has HIV. Even though you come to collect the pill maybe for your mom because is not feeling well so she asks you to go on her behalf. After you get the pills and you are seen caring them it does not matter what the pills are for as long as it is pills they assume it’s for treating HIV since you once came to test.

I: Ummh, number 11

P11: Ummh the way that I see it I agree these are the all these are reasons why people do not want to test. Someone will think but the nurses there are rude, and when you get there they look at you funny and just seem unwelcoming. Like they don’t make you feel comfortable so how will we talk in that type of environment?

I: Ok finish number 11

P11: That time when I had thrush I had that experience that the person looked at me funny.

I: Ummh

P11: She was like oooh you are now sexually active? Like something like that and I discovered that I had gotten it at the toilet not from sexual activities.

I: Ummh, number 13 you wanted to add something?

P13: Another thing that scares us when welcome to test is immediately when you enter. Ok fine the nurse wills what you are here for.

I: Ummh

P13: Then you tell I am here to test, they will say “no aah you! You are very young for you to be coming to test, why is it because you are sleeping around?” Even though you are not sexually active, you just want to know whats happening with your life, you want to know your status but you will hear them say “why are you here? You are sleeping around” that’s why like most people don’t come to the clinic to test, they get scared and go back home.

I: Ummh

P13: And they don’t want to continue the process no more.

I: Ok, so what do you think can be done? Number 12 you wanted to add something? So what do you think can be done to encourage people in your age group so that they go test for HIV even if we have the challenges that you have mentioned. Number 15 mentioned that if we hire people with a heart like nurses and number 15 mentioned that nursing is a calling you know, you just don’t become a nurse. Sorry number 11 mentioned that nursing is a calling like people that know their job. What else do you think can be done? Number 11

P11: Ummh incentives what are those things, incentives like things that are similar to a bribe so that you can come and test, like you come and test and we give you something. Us as young people we like things so give us something we will come.

I: Ok, like what thing? Like what?

P11: Something like earphones, watches, lipsticks like things like that.

I: Ummh, do you think those would encourage people to come and test?

P11: Yes

I: Why?

P11: Because we like things as young people and free things as South Africans we all know that we love them, yes so if you tell us come we will give you this we will come.

I: Number 15 do you agree?

P15: Yea I agree especially food yho! If they say if you test we will give you food and drinks we will definitely come.

I: What kind of food?

P15: Like nice food there must be meat and salads, we would come and we call each other to come.

I: So number 11 mentioned a very good concept, you mentioned incentives right what you think those are because you mentioned like you gave a brief description.

P11: Yes

I: So I want to hear from all of you guys on what incentives are. Number 14

P14: I think incentives are- I don’t know the specific explanation but then its I give you give me like we both benefit from each other; we both benefit from what we are going to do. If I give you a packet of snacks which means you will maybe escort me to the shops and buy more snacks know yea.

I: Ummh, number 12 what do you think incentives are?

P12: [inaudible]

P13: I think it is something that will attract one to come and get tested, like its things that you fetch in most instances in return for testing as number 13.

I: Ummh.

P13: And then maybe I get I t-shirt or a cap, obviously even number 11 will also want to test cause they have seen me as number 13 by coming to the clinic I got something so she too will come and test too in hopes of getting those things. Even if she had not planned to come and test but they saw number 13 got something they say let me go too and know my status and also get a t-shirts and a cap.

I: Number 14

P14: Emmh, this is what they do at {XXX} (name of a non-profit organization)immediately after you donate blood they give you a juice, packet of snacks, packet of sweets, a dictionary, a diary and all that stuff a pen, stationery. Just to say thank you for coming to donate, obviously when you leave {XXX} (name of a non-profit organization)you are fully packed you are fully loaded and when going back home you tell people I donate blood and I got these in return, and they will also say we are going too cause we want those things.

I: So do you think these things dictionaries and stationery would also help as well since they are have been used in blood drive. Do you think they can also be used as incentives?

P14: Yes they can also be used.

I: Is that something that would encourage you personally to come and test?

P14: Yes cause eemh I am a learner and as I am a learner so I need a dictionary on most of the things and it will also help to give me more knowledge and all that stuff.

I: Number 11 you shaking your head tell me

P11: Ummh I do not agree; we all know South Africans we are scared of school. Something which relates to books and pen to write. Your mother can hide money in the dictionaries you won’t find it because you won’t go there, what would you be doing there even? So incentives should be something like fashion, food, money those are the type of things that will attract young people.

I: So, ok number 15 you want to add something?

P15: I don’t agree with number 14 cause when they are giving dictionaries and stuff they inspire you cause sometimes you find that at your house they cannot afford to buy you a dictionary. So getting a dictionary will help when you need it you will be able to use it. It inspires us that we learn obviously we want to succeed in life.

I: Ummh

P15: Because people come from different backgrounds, so even if they cannot afford one at your house, you can go and test and get it after you test.

I: Number 13

P13: Ummh, I agree with number 14 some of us actually need these dictionaries and you find that your parents cannot afford it and at school they need you to have a dictionary. So it wil be a good incentive to get us dictionaries or dairies. In my diary I can be able to write my secrets, for instance if I am to find that I am HIV positive and I do not want to disclose my status and I can write in my diary that I am positive isn’t I can lock it and no one can see what I write. Or I know that I can put it at a safe place where no one can find it, a diary can help if I am not willing to share or disclose my status to anyone.

I: Ummh and what’s your thought on that number 12? What kind of incentive would encourage you personally to come and get tested? Do you think diaries would help? What is it that you agree with?

P12: I agree with number 11, when you look at the township there are a lot of people that are not going to school, so what good will getting a dictionary as an incentive do since we are not in school.

I: So what kind of incentive would you want?

P12: Mmmh?

I: What would you like?

P12: Food [giggles]

I: What kind of food?

P12: Whatever type of food that would be given is ok; if someone gives you food you accept and won’t be choosy about the type of food that you will be given.

I: Number 11

P11: Ummh, I think that if they want to encourage the youth more, they must look at what the youth likes and what they want.

I: Ummh

P11: Like, let them give out those things they will see.

I: What do the youths like?

P11: All the wrong things

I: Like?

P11: All the wrong things, dagga is now legal now they can give out dagga, alcohol, yes all those things. Free tattoo maybe, free beers, if you just give those you will see young people.

I: Ummh

P11: Ok I like I am young person and I know topics that make us come together, these are often topics that are not right, often things that that bring us together are bad things those are the ones that we like. So if you come and give us books what’s the point when the ones that I have at home I don’t even read? Of course a dictionary can be helpful and all but these days there are cell phones that we can use to search information instead of using an actual dictionary

I: Ummh

P11: Yea

I: So at the beginning of the interview you mentioned that you guys were scared of testing for HIV right.

P11: Yes

I: So do you think if we still give you these things you will still be scared? Like what do you think will happen?

P11: I will be

I: Number 11

P11: Oh I won’t be scared as much of course I will still think what if I am HIV positive but I will also be thing of the incentives that I will be getting after the thing that I can’t afford myself.

I: Number 12

P12: No! like I don’t agree with number 11, cause what’s the point of getting these things and then you find that you are HIV positive, those things would not matter much at that time all you are thinking of is that I am positive.

I: Ummh, number 13

P13: I don’t agree with number 11 [giggles] on that part that the youth need to be attracted to test using alcohol and drugs things like that. It’s not right because these people want to test us and make sure that we are alright and we know our statuses. I don’t think alcohol and drugs are the solution...

I: Number 15

P15: Can I please add to what number 13 has said because honestly speaking if they give us alcohol we might just end up having unprotected sex then contract HIV. So it will just make things worse and the virus spreads all over.

P14: I agree with number 11 that they must give the youth things that they like, if they say come and test and we will give you bibles no! We would look at each other and say bibles? What would I even do with a bible. So obviously if they say to you please come and test and we will give you cigarette, wine obviously people will come yho! with the end goal of getting those things I would even call my friends and say“eh! My friend there is free alcohol here” obviously you will see everyone from the township will be here at the clinic to test. But then I don’t say that they must give alcohol or dagga so that we can go and test. They can like give us they can say please come and test and we will give you these cards. Like these are scratch cards and you will get airtime of R12 or airtime of R10 so obviously that will be free airtime, as a person you think of free airtime. You just think I will get myself data and chat all night and day.

I: Number 13

P13: Yea I wanted to say that I agree with number 14, there is no need to provide alcohol, cigarettes and stuff, like there are many things that they can use to attract us as young people, like alcohol its not a solution.

I: Give me an example

P13: As number 14 has said, like airtime for free, like we love the internet like data for free we would appreciate it. Like now {XXX} (name of a cellphone network service provider)you buy airtime for R17 and you get to access Facebook for free. Like we like those type of things and alcohol is not a solution. Like I said before things like t-shirts we like those and maybe print something nice. Caps, airtime for free, food plus we really love food, juice, like is nice right, like lipsticks for girl’s yea.

I: Ummh

P13: Yea, not alcohol no

I: Number 12 you want to add something

P12: Yea something like vouchers so, like you get a voucher from {XXX} (name of a fast food restaurant). Who would just give you a voucher from {XXX} (name of a fast food restaurant)? [Giggles in the background]

I: A voucher ok.

P12: Yea

I: You think that will work number 11?

P11: Ummh, number 11 I think I agree with you don’t have to give them all the wrong things to attract the youth, but I was making an example that they are attracted by wrong things.

I: Ok, can I ask so far what methods are they using to attract the youth, so far what methods are they using?

P13: Ummh as far as I know the methods they use now to attract

I: Ummh

P13: They give out bottles for free, t-shirts, and caps, and key holders so far.

I: And do you think they work those things?

P13: Yea, I can give an example like about boys in the area and circumcision in June they were attracted to come using bottles,shirts and key holders. Those are the things that they would come with from the circumcision place, so others would be attracted that way when they see their friends bringing bottles and stuff, t-shirt or a cap they say let me go there too.

I: Ok

P11: I don’t agree that it’s really working ok; if it was working you guys would not be here. A way would have been found by now to control HIV and a lot of people would have tested for HIV, and those people by {XXX} (name of a retail chain supermarket) wouldn’t be there. So I think it works but it is a slow process. So they actually need something that will attract people even more. It’s not working because there are many people that I know that are not circumcised

I: Ummh, number 14.

P14: Ummh I agree with number 11 they must find something to attract the youth like something that will bring people to test fast

I: Things like what so?

P14: Like not really sure but something that will make people like the youth to come and test.

I: Ummh

P14: I also agree with number 13 that they don’t have to give out the wrong things so that the youth comes to test. Ok like if they give us alcohol by the time I get to the corner I will be drunk and I meet people that want to go to “Mabaso”, then I will go every other place and I will wake up undressed the next morning. Not knowing how many people I slept with, I am also I am HIV positive, I am pregnant I don’t know the father of the baby I don’t even know how I got to where I am cause of alcohol.

I: Ummh, number 13 you have your hand raised up

P13: I agree with number 14 like other things are not right cause ok, if they attract you using alcohol and cigarette things like that. We would want those things daily and end up doing things that are not right. Like stopping school, getting pregnant as number 14 said you get pregnant for someone you don’t even know and that person won’t want to be responsible for the child and one is forced to end up having an abortion.

I: Ummh so number 11

P11: Ok they are all my point all these numbers ok

I: Ok

P11: They are all my point this whole thing of alcohol but they didn’t hear me properly, I said the youth are attracted by wrong things I didn’t say we must give them the wrong things

I: Ok, so what do you guys think of maybe like so you mentioned like stationery right like what kind of things stationery will that be?

P?: Pencils and pens

I: Ummh

P?: Pencil case like at times there are kids from shacks and when you look at them they don’t have the basic things that they need.

I: Ummh

P11: I think that these people that test should go to schools

I: Ummh

P11: They must give the school children forms to give to their parents or call a parents meeting after that parents that give their consent that their children be tested they will be given school uniform.

I: Uniform ok

P13: Ummh I agree with number 11 cause there are other school kids that do not have uniforms, so yea I think it is a good idea to give uniforms to the kids that test, they give them uniform, school shoes and jerseys for winter sometimes you find in winter some kids have no jerseys during winter or the jerseys are torn. So it is ok that they give them these.

I: Ummh, number 15 do you agree?

P15: Yes one will be encouraged to go to school daily when they have uniform. Sometimes school kids are afraid to go to school because they have no jerseys and stuff.

I: 14

P14: I think that they should go to the universities obviously when you are a 1^st^ year student, let’s say the situation at home they can afford all the textbooks, you can afford the study guides they can afford all the things you need laptops, printing machines, faxes and all that stuff. So if they go to the university and say after you test we will give you study guides or we will give you textbooks obviously cause I don’t have textbooks but not because I want to know my status but because I will get textbooks so that I can get education.

I: Ummh

P11: I think there should be bursaries for people that test

I: Bursary?

P11: Bursary Yes they should go to or they should be learnerships for people that come to test, the more you are on the learnership you must test every three months they must be told that every three months you test know your status.

I: And you think that every three months should that be how often they should give these things?

P11: I, ok, I don’t understand what you are saying

I: So isn’t you said every three months they must give these bursaries

P11: Yes

I: These things that you have mentioned. You think every three months they must get it

P11: No

I: What do you mean?

P11: Its part of the bursary let’s say the bursary is for four years, and you are part of that bursary you will become a member of the organization that gave the bursary and you motivate other people that they come and test and you test every three months you go and test yourself

I: Ummh, ok and how often do you think these things should be given?

P11: Given?

I: Every three months?

P11: Yea every three months

I: Why do you think so?

P11: Yea cause more people need to be recruited

I: So you are saying for example let’s say we were to give you food right or airtime, would you want that every three months?

P11: No I would

I: How often?

P11: Every month I would need that

I: So you would test every month?

P11: Yes [laughs] yes

I: Ok

P11: Like if you want to build a healthy nation we must take risks I mean steps even if it means taking one person and they go test for HIV and they know their status and if they are positive they are put on treatment immediately.

I: And practically you don’t think that would be expensive?

P11: Oh! It will be expensive

I: Ok and what other challenges do you think will be with giving out incentives.

P11: As giving out incentives other people, number 11, other people would want to come twice so they get the benefits twice on the same day ummh ummh yea corruption might be rife cause of these incentives.

I: Ummh

P11: And other people who give out those things can become corrupt and not give people these things but sell them.

I: Ummh, do you think, what other challenges do you guys think of with providing incentives for HIV testing? Number 13

P13: Ummh, number 13

I: Ummh

P13: I think as number 11 has said it can bring corruption because like I’m sorry to say this but as black people we love things that are free, where ever there are free things we are there. I can go now and they test me and I find that I am positive, I can come back cause I don’t care about anything else I just want these incentives. Even if I am negative I will go back people always want things for themselves they don’t give others a chance.

I: Ummh, 14 do you agree as well?

P14: Yes I agree with number 13 and number 11. Because other people they are like I went for the first time and got a plate of food and at home they are five so she changes clothes and goes back for more foods. Like as people we are never satisfied or appreciate that maybe someone maybe gave you a bucket of water, we now question why someone else was given 10 litres

I: Ummh

P14: Yea

I: Number 11 you have your hand raised

P11: Ummh ok there are people who came to our school once to talk to us about HIV. There was like a guy like a male person. He told us that the reason why they were there was that the government is using too much money on supplying ARV and stuff so their intention was to reduce the prevalence of HIV. So what I am thinking is that in as much as we want these vouchers on these incentives we must not forget the government suffers too. Transporting medicine here it’s expensive. Testing should be about us wanting a healthy nation, it should not be about briberies but because us as the youth we want the wrong things more than the right things.

I: Umhh

P11: So people have to find methods even though they hurt them somewhere somehow that we are attracted to testing.

I: Ummh

P11: Yes

I: Ok, going back to number 14 you have your

P14: Ummh, I agree with number 11 that eemh government wants us to be a healthy nation but then us as a nation we don’t see that we just think that HIV kills that’s all because we are uneducated about HIV, people don’t know anything about HIV. The only thing they know is that HIV is a virus and it kills, and there is this conception that only people that sleep around get HIV. Obviously if you are 12 years old and you are HIV positive obviously the woman in the community will say she was sleeping around that is why she is HIV positive. Firstly, they do not know your background and how one contracted HIV and there are. They don’t know that maybe the person was involved in an accident then there was an accident and there was a mixture of blood with another person that had HIV and that time you had a cut. You can also have HIV that way but they think the only thing they think is that sleeping around is the only way one can get HIV, this thing that they are uneducated about HIV is what is killing the nation, there are so many misconceptions, like it’s not easy for someone to say I am going to test cause people do not know the reasons why you want to test. It’s also not easy to wake up and decide to go and test you will keep asking yourself what will people say about me.

I: Ummh

P14: What will they think about me, how will I live when I come back from clinic from testing, like the minute you enter the clinic you already know that everyone knows that I went to the clinic to test and that’s not right..

I: Ummh and what do you think we can do to educate people about HIV like older people?

P14: Ummh like there should be classes not proper classes but go to a class and be taught. No like let’s say right now people come to clinic the classes can be done here.

I: Ummh

P14: At the clinic while they wait to see the doctor and all that stuff, let’s say one of the nurses can come and say I am here to teach you about HIV and the ways you can contract it, ways to prevent HIV, and the ways like we can use to play it safe and after that the parents will teach their kids at home about HIV the same way they were.

I: Ummhh

P11: Another way in which they can teach people are that there are people that are trained and they will go house to house. House to house I think as this can help save money and attract people at the same time.

I: And what way is that do you have any idea?

P11: Ummh, use cheaper things or we work with the community and counsellor, let there be a day that all the youth meets in one place and certain topics are discussed. And this law that a child must have parental consent before they are tested it is not right. Because other parents they are very ignorant so there must be a law that rather says all school going kids must test for HIV.

I: Ummh

P11: Yes, I think it will help reduce somewhere somehow.

I: Ok, back to incentives that you guys mentioned right.

P: Yes

I: So you mentioned earphones, watches, lipsticks, food, t-shirt, caps, cell phones. Number 12 mentioned voucher, number 11 mentioned cell phone, and 11 also mentioned bursaries and learnership. So what I want to hear from all of you guys is you have to give me your top 3 of all the things that you have mentioned. Something that would encourage you personally out of all the things that we have mentioned here. And you have to give me the reason why that thing is number 1 or number 3. Number 14 you want to go 1^st^?

P14: Number 14

I: Ok

P14: Airtime

I: Ok

P14: Emmh everyone needs airtime, young adult, adolescent everyone needs airtime

I: Ok

P14: And for you to get that airtime you have to go through testing right. Us as the youth we just want data. Every day we want data; you will go testing so that you get data.

I: Ok, and the thing you will choose second?

P14: Ummh my number 2 its vouchers that number 12 mentioned

I: Ummh

P14: Obviously when they say come and get tested we will give you voucher for a spa treatment, yho! This is a township if you come here and talk about spa people will get excited yho! yho! Even a three year old, you will see them at the line too because you said spa treatment. Like that thing will get even the one that is sleeping will wake up after hearing spa treatment.

I: Ummh

P14: And the other thing is food. Yho! Food if you can tell my friends about food, you and them can be best friends for ever cause of food yho! yho! yho! that’s another thing.’

I: Ok, let’s hear from you number 11

P11: Ummh number 11, is it necessary for me to mention the ones the ones that I have mentioned before?

I: You can, so your top 3

P11: My top 3

I: Ummh

P11: Ummh bursaries we all know that some of us we come from backgrounds that are not right so bursaries will help us a lot when it comes to, let’s say I am in university and I stay on campus and the bursary will cover my studies and all that. And another is food I love food very much so if someone speaks about food you are talking my language neh and everyone needs food and everyone loves food especially if its junk food. Ummh the third one could be vouchers clothes vouchers yes, I am a teenager I love fashion whatever comes in fashion I also want it.

I: [giggles] Ok number 15

P15: Well personally a bursary cause I have a baby so I want us to have a better life and make my parents proud that even if I have a baby life goes on. Food is number 2 I love food a lot. The third thing what can it be, I don’t really like things so maybe they work with a youth organization that focuses on art I love art and we can entertain the youth and this will also give us a platform to talk because it is not easy to talk to adults about such issues.

P13: Ummh number 1 its school uniform cause like there are children who go to school and don’t have school shoes and jerseys and these things are essential

I: Ummh

P13: Number 2 its food I love food

I: What type of food?

P13: Anything like any type of food, I love vegetables especially. Ok and number 3 eish my number three I can say bursary for those in university cause maybe the parents can only pay for one year and after that it is hard to continue paying.

I: Ummh

P13: So they need help and NFSAS will also continue helping.

I: Number 12

P12: Oh airtime eish data yho! And then u number 2 food, and then number 3 is school uniform.

I: Why? Why in that particular order?

P12: Other kids the background that they come from they need these things like uniform cause maybe their jersey is torn and its winter

I: Ummh

P12: Yea

I: So you think ummh, number 14 you want to add something?

P14: Guys did you notice that everyone mentioned food? This means with food as an incentive people would be encouraged to come and test that means it should be on the top of the list because everyone mentioned food. So they really must use food as an incentive and it must be made evident even in the plate, like a plate of food that has pap and wors and gravy, the wors must not be small. But in appropriate proportion with the pap. The wors must be a lot because after you have eaten it must be evident, and even when you take a takeaway plate it must be seen that you had good food.

I: Ok so number 11 mentioned that we can maybe give people cell phones right, she mentioned that we could maybe give people cell phones. So what I want to find out if you all have cell phones right?

P?: Yes

I: So how do you think we can use cell phones to maybe get HIV testing information out there to people like you?

P14: Social media everyone uses social media and then if we are scrolling on Facebook they must not say it straight like come test for HIV but they should put a joke obviously when someone sees a joke they would want to read everything since it is a kind of joke. In the middle of the joke say something like “I once fell, when I got up I went and tested and they gave me wors after that” So everyone will be like eh joe! Where did you get this wors and you say {XXX} (name of a clinic), everyone will say let’s go and test. So obviously from there that post will be shared

I: Number 12

P12: Another thing that we can use with social media ok, is like the clinic {XXX} (name of a clinic)host a party nothing big so that the youth can be can be encouraged to come and test.

I: Like a party, like an event?

P12: Like an event a small one so that we can all just make ourselves happy.

I: Like what will happen there? Like what would happen there?

P12: Like 1^st^ we test ok. And after testing we celebrate, we won’t do anything big and there is no need for alcohol just provides food.

I: Ummh

P13: Ummh number 13, I don’t agree with number 12

I: Why?

P13: Because like some of us like let me make an example using myself like if I come to the clinic to test and find out that I am positive obviously I will not enjoy the party I will not be interested in it since I just found out that I am positive. Let’s say maybe if they give us incentives for coming to test that is better and I will not have a problem with that cause even if I am positive I can take that thing and keep it in my house till I figure out what to do.

I: Ok, number 11

P11: Ummh can I say something different from what number 13 has said.

I: Ummh

P11: First of all that part I agree with number 12 ok cause that party we would be doing it for someone who has never tested before. Ok we are not refusing any of what she says, when someone finds out that they are positive in this party we will provide counselling for those kind of people, they will not go home like that , there will be people that they can talk to help them calm down and accept the situation. This thing can help cause during that event they will be teaching people about HIV like guys go and test and all that. Actually it will be an encouragement on one hand and on the other we are actually having fun.

I: Ok back to what number 14 said about social media, Facebook. What other social media platforms do you think we can use?

P14: Ummh, its Twitter, ummh even newspapers. There are other people that they still read newspapers and they still enjoy reading newspapers. And magazines, the fashion magazine while you look at fashionable things you come across an advert that says go and test for HIV yea. Even Whatsapp, you can send a link then that link is shared among people. Even on the internet they can link this word HIV to other words so that when you search HI the V comes out, and then you will see HIV and then you will be curious and read up on HIV and there they will encourage you to go and test for HIV.

I: Ummh

P11: Another thing on social media, Oh! Number 11, talking about newspapers, there are these papers people put around poles, they can use that way too cause when you are looking you do not look down but around so everywhere there must be things about HIV. So even in buildings there must be things about HIV/AIDS even in taxi ranks.

I: Back to Whatsapp, Twitter and Facebook, like what do you think are the challenges with these things, like maybe using these platforms to get information out there?

P14: As people we are all not the same out there are other people just add negative thoughts to the link or to the advert of HIV testing. Then you can comment and say guys lets go and get tested someone can reply and say “since you go for HIV testing it means you have this HIV”. That will obviously start commotion like how can you talk to me that way when I am trying to encourage, they you are bringing other things it will be wrong.

I: Ummh, number 15 you want to add something.

P15: Yes, the question was what challenges we would face. The thing is that we like jokes so maybe they can show {XXX} (name of a former president of South Africa) carrying a sheep’s head. We can look at HIV in a way that will attract people because it is such a serious subject so maybe if {XXX} (name of a former president of South Africa) or the {XXX} (name of friends of former president of South Africa) are shown.

I: Ummh do you think maybe if someone like {XXX} (name of a former president of South Africa) like you just mentioned, let’s say {XXX} (name of a former president of South Africa)comes to these parties that number 12 mentioned do you think that would work?

P15: Yes, very much

I: Why do you think so number 12?

P12: Aaah isn’t here in the township people are used to hearing and seeing {XXX} (name of a former president of South Africa)so they will come like yea.

P11: Ummh I say let there be more shows on TV about HIV let there be more jokes, but jokes that will not make a person living with HIV feel uncomfortable.

P13: Ummh, I agree with number 11. They must put things that are entertaining; reading sometimes is tedious so we need something to entertain. When you just write about someone can say who said I want to test for HIV and it won’t be such a serious thing even though the person does not know their status.

I: Ummh, number 14

P14: Ummh, like this thing they should do it like at blood drive. At {XXX} (name of a non-profit organization)there’s a photo of a small child, like she is inside a hand beneath that child is the slogan “your blood can save this child”. Obviously when you look at this child you think of other kids like here and you decide to donate your blood so that children like her can be saved. And sometimes you get a chance to meet the child that you donated blood to and show you look this child is alive and well thanks to you.

I: Ummh, ok let’s say you are receiving all this information on Whatsapp or Facebook right. Like these jokes like you mentioned jokes on Facebook or any other HIV related information on your phone. How do you think your parents will feel when you receive those types of messages?

P14: No like emmh

I: Your parents

P14: Like your parents if they are educated about HIV they will also feel the need that, like you can say mama I saw this message on Facebook that is like this. And then if she is knowledgeable or experienced about HIV then she will say my child go and test and she will explain to you that there is no need for you to feel uncomfortable or to feel like there is no need to go and test cause it’s the right thing to do.

I: Ummh

P15: I see it as the right thing because at home we sit down and talk about HIV! That you can take your treatment, exercise and eat right you would still lead a good life. Like we have a family member that is living with HIV and one can’t even say that she has it. Cause we talk about it and are open and we discuss that if you are ever in this situation no need to kill yourself but she is proof that you can continue living even with HIV.

I: Why do you think that some people are not open with their parents?

P15: It’s because community is the one that makes people not want to disclose their status because they will assume the person got it from sleeping around so like even as the youth of today we know that there are many ways of contracting HIV besides sex, so the community must educate people that HIV can be contracted in these ways, if you are a child and your mother has it can be transmitted from mother to child it is not a matter of sleeping so people must be given more knowledge cause people only know sex.

I: Ummh

P11: Ummh as far as I know HIV came in the 1980’s the way it was introduced it was a deadly disease people were told that once you have it you die, you cannot even touch or sit next to someone with HIV you will die. So I think that the background of it, the way it was introduced that thing is still in our minds that once you have HIV you die, you can’t date a person with HIV cause you will die but we have things like condom which you can use during intercourse and all that. So I think it is all how HIV was introduced

I: Ummh, so how do you think, speaking of the introduction HIV right, there are some people who are still uneasy to talk about it. How do you think, do you have any suggestions how we can encourage people like you the youth to speak more openly about maybe their sexual behaviours?

P14: Like TV you know, you see right now on {XXX} (name of a local TV soapie) the thing that is done by the character called {XXX} (name of a character in a TV soapie) that people must open about their sexuality, so they can also use that thing to encourage people who are HIV positive and take a stand and say when I am HIV positive but that doesn’t mean I will die today or tomorrow or day after tomorrow but then that, means that when you speak about HIV you are educating others that I am HIV positive but I am still living a normal life and a healthy life, you know.

I: Ummh, do you want to add onto what number 14 has said?

P11: Ummh ok, number 14 is right ok that they can use TV ok. Like you see they can do a story on TV and we are shown people who are living with HIV like how they lead their lifestyles like they lead normal lives they must speak of these things, let’s say one speaks and says I got HIV at a certain place but today I am still alive and I am taking my treatment and it helps a lot. Often times we like things that we watch than something we read.

I: Number 12 How do you think we can encourage the youth to speak more openly about their sexual behaviours.

P12…I agree with number 11; the problem is that some people have no TV’s so where will they watch these things?

I: Ummh

P12: Things like that so, maybe we can use {XXX} (name of a local newspaper publication)and they do a story and maybe that can help people be open-minded.

I: Ok, number 15

P15: I think they can a session like this one we are doing today, because such discussion helps as we discussed things in depth and it helped that we are age mates. Cause yho! Your mother you can say mom “Thabo” kissed me, what? Yho! Parents even if your parent is educated or not there are things that you cannot discuss with them [giggles in the background] yho!

I: Number 13

P13: Oh I agree with number 15, like as the youth we like art we follow art, drama and music. Art can help us they can tell today we are doing poetry about HIV and AIDS, and obviously there are people from around who would want to watch us from the community they will say we want to watch “Thubelihle” and tonight. Then they come and watch us doing our thing about HIV and AIDS, maybe then in this drama there can be a woman who goes to fetch her treatment, she eats healthy and all that.

P11: I agree with number 11 her view. But I would like that they add a subject at school, cause in these subjects we learn at school they just give us a brief summary about these diseases, but there should be a practical subject. Not practical but practical in the sense of doing drama about HIV that how can we defeat HIV. There will be experiments showing the whole thing about HIV how to prevent it and everything, or about TB it’s like this and that, where we can speak more about it.

I: Ummh, ok guys thank you so much for all the suggestions that you have so we have like drama, art, ummh subject at school. Is there anything you guys want to add, number 14?

P14: As a youth we like celebrities, everyone has a favourite celebrity and everyone has a role model and it’s a celebrity. So I suggest they bring a celebrity just one and write a song about HIV, and let’s say during some awards the celebrity can do a poem about HIV as number 13 said they do a poem about HIV so that like at the {XXX} (type of music awards awarded to South African musicians) awards everyone wants to know who will win .So if they take celebrities to talk HIV, it will be easier and people will get more educated that way.

I: Ummh ok thank you guys we are almost at the end of our discussion. Do you have any final thoughts about the youth, how we can access HIV testing services more freely or maybe the use of these incentives maybe? Any thoughts on that before we conclude. Maybe there is something you want to add that you forgot on that list of incentives that you guys added. …let me ask you this number 11 mentioned we could give maybe give lipstick obviously that will work for girls, do you think there is specific incentives that would work for boys and other incentives that would work for guys. Number 12

P12: Boys love soccer and maybe bucket hats…

P14: Ummh boys around here most of them are “phantsula” (referes to the type of dressing some boys like in the township), most of them they dance and all that stuff. So if they give them a CD or the speaker box or the USB ok its fine they put their music there. And most of the people love music obviously some of us need music every day.

I: Ok and number 11 mentioned that maybe for girls we can use a voucher for fashion right it’s for girls so any other thing that you think could be girl specific

P11: Girl specific, makeup. Number 11 I think makeup, free hairstyle or give them hair piece. Maybe there is a truck of hairpiece and you choose

P12: Oh! Perfume

I: Anything else you guys want to add? We are almost at the end of our discussion

P14: For boys like tickets for soccer matches so they can go to the stadium and watch soccer or give them let’s say there is a music show somewhere you can go and watch Euphonic playing yea…

P11: Mum ummh, so let’s say they go to teams let’s say boys play soccer and girls play netball they approach them and talk to them talk to their parent’s maybe but them uniform and “Cox” (referring to soccer boots). Or say guys please test and we fix your grounds.

I: Ummh ok, anything else you guys want to add? We are almost at the end of our discussion. Is there anything else you want to add that maybe you forgot to, maybe you just want to share?

P11: Aai some of us will never stop talking so if I speak all my ideas we will not leave so we can close up.

I: Number 15

P15: I am ok I have nothing else

I: 13?

P13: No

I: You have said everything, you wanted to say? Number 12? Anything you want to add number 14?

P14: No I am great cause if I can continue talking eish

P13: Can I add something, as for older people we can organise transport maybe they choose a day and take them somewhere to watch a movie that will about HIV so that they understand what is happening.

I: Thanks so much guys for being part of this discussion, your input is really valued and yea thank you so much. We have come to the end of our discussion, thank you for your active participation mum the time is… 10 past 2. Thank you so much

End time: 14:10
