# Supplementary material for: Exploring perceptions of low risk behaviour and drivers to test for HIV among South African youth
Source: PLoS One. 2021 Jan 22;16(1):e0245542. doi: 10.1371/journal.pone.0245542 (PMC7822253; doi:10.1371/journal.pone.0245542)
Supplement: S1 File — (ZIP) [file pone.0245542.s001.zip › S1_File_Anonymised Transcripts/YA01-004-MK Translation_QC2_TM.docx]

Full Participant ID: YA01-004-MK

Participant Type: In Depth Interview

Location: Winnie Mandela

Date: 06/08/2018

Start time:

Primary interview language: English (Tswana)

Name of Facilitator/Interviewer: Wellington Maruma

Name of Note Taker:

Name of Transcriber: Ornate Masuku

Length of recording: 30:56

Label Key

I = Interviewer

P = Participant

N = Notetaker

{ } = Indicates that details were changed or pseudonyms were used to anonymise data

xxx = words were omitted to anonymise data

- = breaking into a sentence by the next speaker

… = pause or drawn out words

[ ] = indicates noise made, e.g. [laugh], [sigh], [pause]

[inaudible segment] = Unclear section of the recording

?Mulenga Clinic?, ?P3? = questionable text or doubt as to what was said or who said it

I: Qualitative Interview Date is the 6^th^ of August. Uumh interviewer is {XXX} (interviewer name), PID is YA01-004-MK. Thank you for allowing us to do this interview. Do you allow me to record this interview?

P: Yes, yes I…

I: Ok, Thank you. Uumh can you please tell me what your thoughts are on HIV?

P: My thoughts on HIV?

I: Uuumh, what is HIV, what does…what do you think HIV is?

P:…It’s a virus

I: Uumh

P: Yes human deficiency virus something yes

I: Uuumh

P: and then it lives in our blood

I: Uumh

P: …Yea that’s all I know

I: Ok and do you know how people can become infected? How can you become infected?

P: Yes, through sexual relations

I: Uumh

P: If you are not protecting yourself you can be infected and then…through what?? Through I don’t know…I don’t know…

I: Through what?

P: Sexual Relations

I: Ok, and what else? Are those the only way you know? Ok tell me more

P:..I don’t know how to say this

I: Ok

P: Though I am going to try

I: Ok no problem

P: Ummh, maybe if you touch someone’s blood who is infected, then you are going to be infected

I: Uummh

P: and then what again…yea maybe the two

I: Ok, and can you tell me about like places you think a person can be at risk of getting or being infected with HIV.

P: Places?

I: Places or situations

P: Situations?

I: Uuumh

P: …Situations, I think will be when someone, when someone is in prostitution, you see those kind of things and like right now in ,like this life style we are living us youth, like the things that we do

I: Uuumh

P: Yes, and maybe when we are grooving yea, when we are drunk we do somethings,

I: Uuumh

P: Yea that’s when we get infected

I: How else can you think you can get HIV?

P: Maybe you can be raped

I: Uuumh, ok do you wanna talk more about that?

P: [gigglees]

I: And Can you tell me about a situation where you persornally felt you were at risk of HIV?

P: Yea

I: Ok

P: It was… 20, 20 what?? 2014 I think and I was drunk, so I was with my friend we were at {XXX} (Name of place)and we went to party. So there came these guys, they had a car so we went with them to the party then they took us to their place. …And then that day I don’t know what happened, I just found myself sleeping on a bed, but when I touched myself, I felt like I was not good and then, maybe someone raped me or something

I: Uumh

P: Yea and then the person didn’t use protection

I: Uuumh

P: Yes

I: Ok and did you ever get help for that? Did you like maybe attend any testing services?

P: No I was scared

I: Ok tell me more about that

P: I was scared, I was just scared and I was stressed and from then I started losing weight, because of stress I think because I was scared.

I: Uumh

P: Yea and then I didn’t go for help

I: Uuumh

P: I was scared you know how that feels

I: Ok

P: Uuumh

I: Ok and can you tell me about the different maybe HIV testing services that might have been available to you at that time …That you think you might have taken advantage of maybe.

P: I don’t understand the question

I: Ok Do you, uumh Is there any HIV testing services around you said {XXX} (Name of place)

P: Yea there was but I didn’t go for it, because I was scared

I: Ok, and how many of those do you know, like where do you think people can get tested for HIV?

P: In the clinic

I: Ok

P: And hospitals

I: Ok, where else?

P: I don’t know where else

I: Ok, and out of the ones that you mentioned, do you think, uumh, do you think uumh [cough]. Have you accessed any of these before?

P: Uuumh uummh

I: Ok, can you tell me why not

P: Oooh! [laughs], its scary …imagine [laughs] no its scary, like going to the clinic, and sitting there for a test yho! [laughs]

I: Wouldn’t you rather know?

P: Uuumh?

I: Wouldn’t you rather know your status?

P: Eish Its better to know your status but then going to the clinic

I: Uumh

P: Getting tested, waiting for the results yho!

I: Uumh

P: Yho! [laughs] mmmh that’s heavy

I: Yea. What do you think would make you more comfortable to acess these services?

P: I don’t know …maybe if I can get tested at home

I: Uumh

P: Or what

I: Ok

P: Maybe at home neh …or maybe at school, the time I was in school, yea maybe if they came there and tested us, it would have been better.

I: Uumh

P: Yea

I: Ok and why do you think its better to get tested at home or school compared to the clinics?

P: It’s more comfortable

I: Ok tell me more

P: At home its comfortable like …yea I think home is comfortable and school I don’t know but I think school is better like your friends will be there, teachers will be there

I: Uumh

P: And then you will get support, if maybe something you know

I: Uumh

P: The result comes positive

I: Ok, so you think [clears throat] by getting tested at school or home you will be getting support? Something that you would not otherwise would have gotten at the clinic?

P: Uumh, at clinics

I: Why do you think the clinics are not supportive?

P: They are but then eish

I: Ok

P: Your family won’t be there, your friends won’t be there. At school your friends will be there, your family will be there, your teachers will be there

I: Uumh

P: So they will be able to support, give you that support, that comfort you know, yea

I: Ok, and what do you think the benefits are for testing at home or at school compared to testing at the clinic? You mentioned that ummh you would be more comfortable

P: Uumh

I: What do you think are the other benefits are? …Or what is the good thing about testing at home or at school?

P: …I don’t know you won’t have to waste money, like you know because testing, maybe the clinic will be far then you are going to have to use your own money to go to the clinic and sometimes you don’t have money, you see, yea

I: Ok and was is your, what is it that, what comes to mind when you think of incentives? When you think of that word incentives what comes to mind?

P: Can, can, can you tell me again what is the meaning of that word?

I: Incentives?

P: Yes

I: So incentives is more like something that is given to you,

P: Ok

I: On order for, to change a behaviour

P: To change a behaviour?

I: To change behaviour or to make you do something … maybe good or bad but its, yea, so in the absence of that thing certain behaviours won’t be seen

P: Oh ok

I: What comes to mind when you think of incentives?

P: For us as youth, I think is what, for me personally

I: Uumh, for you persornally

P: Maybe if I can get headsets

I: What for? Why would you wanna get the headsets?

P: [laughs] …Why would I wanna get headsets

I: Uumh

P: Because I love them everyone does, everyone wants them

I: Uumh

P: And then they get lost [laugh]

I: [laugh] Yea but im saying my question is why would you wanna get headsets? Like why what for, for you to do what?

P: To listen to music cause I love music [laugh]

I: Ok, and with current HIV testing services, like what kind of role do you think incentives would play in making the youth, uumh to, to like access HIV testing services?

P: Come again? I don’t understand

I: You don’t understand?

P: Uummh

I: Ok, so with regards to incentives like you mentioned, remember I explained what incentives are

P: Uumh

I: So what do you think, uumh what do you think the incentives, what is the role incentives can play in making you want to go and get tested for HIV?

P: Oh! Obviously I, I will they, eish can I say it in SeTswana

I: Yea its fine, its fine

P: Obviously by that time I would want to get them

I: Uumh

P: So I will be forced to, not really forced to but I would want to go so that I get what I want

I: Ok and then

P: Uumh cause that’s the thing that usually encourages people

I: Ok and then why do you think this will encourage people?

P: Not people but the youth

I: Why them?

P: They want them, obviously they want them

I: Yea, ok and uumh do you think these incentives would make them want to get tested for TB for HIV sorry?

P: 100% sure, im sure of it.

I: Ok and is that something that would encourage you to go and get tested?

P: Of course [laughs]

I: [laughs] Ok, and what kind of incentives are these? Like you mentioned headsets

P: And then maybe what? I don’t know anything

I: Give me an example

P: A shirt maybe a cap

I: Uumh

P: Or maybe I don’t know what else, maybe lotions, something I don’t know

I: Uumh

P: Yea I think that

I: So you mentioned headsets, shirts, caps and lotions, like give me an idea of what this shirt would look like

P: The shirt?

I: Uumh

P: Maybe a golf shirt

I: Uummh

P: It doesn’t have to be expensive

I: Uumh

P: But the cap though has to be expensive [laughs] but not too expensive, at least it must be nice

I: Ok and then why do you think the cap has to be expensive than the shirt?

P: Cap is gonna ecourage boys, to come and get tested that I am sure of

I: Uumh ok and what do you think will encourage girls?

P: Yho girls! [Laughs]

I: Yea

P: Uummh, what mara …ok girls, I don’t know maybe lotions but im not sure maybe there have to be something else for girls

I: An example anything that comes to mind?

P: Because I am a girl let me think

I: Yes, yes

P: [laughs] Maybe cutex yea, cutex yes

I: Ok cutex, is that nail thing right?

P: Yea

I: Ok, what else?

P: …And then what and then what? Maybe cosmetic something you know,

I: Uumh

P: I don’t know I’m

I: Uumh

P: Just something yea

I: Ok and why do you certain incentives would encourage boys better than girls?

P: What a cap?

I: Uumh…why do you think cosmetics won’t work for boys and why do you thinks caps wont work for girls? Or something like that

P: Oh! Because boys love caps and then girls love the cosmetics, cutex, makeup, at least soap for the face

I: Uumh, ok and out of the things you mentioned which one would be more important for you? You mentioned headsets, shirt, cap, lotions, cutex, cosmetics what would be more important for you? Something that would encourage you persornally?

P: Me?

I: Yea that one thing

P: [laughs] Headsets?

I: Headsets? Ok.

P: Yes

I: Ok , why do you think for you its that important compared to the others?

P: I want them, I, I always want then i even though I have them I still want other ones

I: Ok if I was to give you a bag of cosmetics instead of headsets, would that change your behaviour in terms of coming to test for HIV?

P: No cosmetic will be ok because I will appreciate, since I didn’t buy with my own money

I: Uumh

P: I didn’t use my money

I: Uumh

P: Yes

I: Ok, and then how often do you think these things should be, should be uuumh should be given, should be provided?

P: Once

I: Why?

P: Why always?

I: Why not always?

P: This is money we are talking about [laughs]

I: Ok

P: We cannot get everything for free, like always uumh uumh

I: Mmmh, but why do you think we need free things to encourage people to go and get tested, why isn’t it up to them?

P: Because we are black people you know how we are [laughs], black people are like this [laughs]

I: [laughs] ok

P: I’m sorry for bringing that part

I: No it’s fine, it’s fine, it’s fine. And so say that there was a company right that would offer these things to the youth around {XXX} (name of an area) or wherever

P: Uumh

I: What do you think the challenges could be in getting these things?

P: Getting who the youth?

I: Uumh

P: The challenges?

I: Yea

P: Yho! I don’t understand your question

I: What do you think the challenges of providing these incentives will be, for HIV testing services? Do you think it would be a good thing, like do you think by just giving them to you people will automatically come or what challenges can you think of, is there anything you can think of?

P: Uumh uumh

I: Ok and what are the good things about, what do you think are the benefits of providing these things?

P: I think ahem, most of the youth, will will be able to know their HIV status from then, cause after, before getting those they will have to get tested

I: Uumh

P: And then I think these things will encourage them to come and get tested

I: Ok and why do you think in the absence of these things people do not wanna come?

P: I think yho! Like I said getting tested like so its scary, so these things will obviously encourage people to come, they know that im not just going to waste my money, im gonna get something yea

I: ok, so you did mention at the start of our interview that you do have a cellphone

P: Yea

I: So how would you feel about getting HIV testing service information about I mean yea HIV testing informationon your cellphone?

P: Yho! That would be great [giggles] yho! much better, uumh uumh

I: Ok and what would be one those types of messages?

P: Mmmh?

I: What would be on your messages?

P: What would be on those messages?

I: Yea on those messages, on those information that you are receiving from your cellphone. What would you like to be on the message?

P: [inaudible, 18:45]

I: You like what?

P: straight answer or

I: Ok

P: I don’t know if maybe I don’t understand the question?

I: Do you want me to rephrase it?

P: Uumh

I: So I am saying, so you have a cellphone right and you are saying that it would be a good thing for you to get, like information regarding HIV testing services right

P: Uumh

I: So I want you to give me an idea of what messages, you would want to get

P: …Oh! The kind of message I would want to get?

I: Say like if you were to get a message now about HIV testing services, what would be in that message give me an idea. Like just paint a picture for me …So your phone rang now and you receive a message yea about HIV testing services?

P: Yea no I understand you [giggles]

I: Ok ok I just have to make sure

P: …Yea on the message, I think, I think maybe if the message can be or the messages encourage that I must get tested

I: Uumh

P: Mmmh

I: Ok, and have you ever recieved, like maybe a message like that on your phone?

P: Yea but not on my phone

I: Ok

P: But they send my grandmother those types of messages

I: Ok

P: Mmh

I: Ok give me an example, like what would be on that message? You spoke of encouragement

P: Oh! In a message they say she must eat healthy, she must go for testing and she must do what now, that’s all I remember

I: Ok, and do you think that kind of strategy will work for HIV testing as well?

P: Yea I think so, but in a different way not not to tell us that we must eat healthy

I: Please explain to me that difference, explain to me that diffrence

P: In a way like, they must tell us about th …I don’t know how to explain this

I: Uumh no, its fine relax

P: Tell us what? Tell us yho! Maybe tell us what will happen if we do not get tested and the disadvantages of testing or not testing for HIV, yea

I: Ok

P: Ummh

I: And then if we could say there was a platform,or maybe you know those * what what that you must dail on your phone, ok so lets say there was those types of things that you must register for HIV testing services. How would you feel about those? …so maybe you have to dail that * what what and then you will

P: For free or?

I: Uumh

P: For free? For free yea

I: But how would you feel about it like

P: How would I feel about it?

I: Uumh, would that be a good thing would that be a bad thing why not why

P: It would be a good thing, uumh

I: Uumh why?

P…Why?

I: Uumh

P:…I think it will be good because its on the phone, and then you said registration is on the phone, yes

I: Uumh

P: And then everything these days we doonline so fone for me it will be ok

I: Ok

P: Uumh

I: When you say we do things online, whatelse do you think, what you guys do online

P: Mmmh?

I: You said that you do a lot of things online

P: uuUh

I: Give me an example of things you do online

P: A lot of things

I: Like?

P: Everything, we live online [laughs]

I: Ok so online what do you do, like obviously there is google whatelse?

P: facebook

I: whatelse

P: Fhatsapp

I: Ok

P: I use the two, facebook and whatsapp. Then there is also twitter, instagram

I: Ok and how would you feel if you were to receive HIV testing services through those

P: That would be great [laughs]

I: Why?

P: Why?

I: Ummh

P: Why do you always ask why? [laughs]

I: No I need to know why would you choose facebook over whatsapp and why would you choose sms over any other stuff

P: Ok I would choose whatsapp

I: Why?

P: Because whatsapp is more private, facebook is public, message uumh is also ok ,still fine but whatsapp will be the best for me

I: Ok, and on whatsapp like those messages that you would receive where would you want them to come from? Your friends or from yourself or to something, like where would you want to get these messages from? Who will send them to you on whatsapp?

P: On whatsapp always get messages with links to things,like games they send the games so that we play games, you know those things

I: Uumh

P: So we can also receive the messages in that way

I: Ok

P: Yea,

I: Ok ok

P: We send to each other as different family and what

I: Ok, and then the challenges of using social media like for HIV testing for, for distributing information ka HIV testing services? What do you think those are?

P: These days

I: Uumh

P: I don’t think there will be much of a challange, cause data is not that expensive, I was going to mention data but it is not that expensive

I: Uumh

P: Mmh

I: …Ok and so say that I send you a message about HIV testing services what will your mother say? What do you think she will say?

P: I think she will be happy

I: Why?

P: She is always encouraging that I must get tested,that I must look after myself I must do this, I must do that, so if she sees that type of message she will ask me about it but will not have a problem, I am sure she will be happy

I: Uumh, and then why do you think other parents are not happy when they hear their children receive such messages?

P: Mmmh they are overprotective, they are protecting their children because some parents dont believe that their children should get this type of information. This is because they still hold on to beliefs of old age

I: Uumh

P: And these parents themselves do not have the right information

I: Uumh

P: Yea

I: From everything we have discussed ,what do you think should be done in terms of the youth, over and above everything that you said, what will make the youth go to test for HIV and if they are HIV positive stick to treatment?

P: The youth is diffificult

I: Give me another suggestion over and above the ones that you gave me any other suggestions?

P: Any other?

I: Uumh, why do you say that the youth are difficult?

P: Yho! they are hard headed [laughs] serious we are difficult. Some of us we have pride, a side effect of ARV’s is obviously you gaining weight and then the body changes, but still they still want to look good so they stop taking treatment

I: Uumh, so what is it, like maybe give me an idea that comes to mind so that youths sticks to their treatment, …cause you mentioned that if maybe we give them headsets what what so that they come to the clinic. But in order for them to adhere to treatment what do you suggest?

P: They must change treatment, that bit must not be taken everyday [laughs], everyday no, drinking pills daily every day no, persornally I do not like pills

I: Ok,so they must change treatment and whatelse?

P: Maybe if you inject them like you do on women that come for the prevention of pregnancy

I: Ok so you think by changing treatment and then inject them that would make them, encourage them to adhere to treatment of HIV?

P: By changing yea, changing treatment I think

I: Ok and then do you have any final thoughts about incentives,HIV testing or youth, any other thing?

P: Uumh uumh

I: Anything that comes to mind? mmh

P: Uumh uumh nothing

I: Nothing, ok so just wanna wrap up everything [shuffling of papers]. So you said the testing services for the youth is at the the clinic or hospitals. Where else?

P: Uumh, I only know clinic and hospital

I: Only?

P: Uumh mmh

I: Have you not heard of testing services in tents?

P: Uumh uumh

I: Ok ok

P: Persornally I would not go to that one

I: Why not?

P: [laughs] Why not? its public, isn’t the ones that they pitch up on the streets?

I: Uumh

P: Its too public maybe the community halls

I: Ok

P: Yea maybe it will be better

I: So you think if maybe HIV testing services were conducted in community halls, people will go there more than if its conducted at some clinic or?

P: There must be everywhere both clinics and community halls

I: Ok so we are almost at the end of our discussion, ummh so you don’t have any other final thoughts about the incentives that you? Maybe you wanna add more

P: Uumh uumh

I: Ok …thankyou so much for being, for your participation, and would uumh yea. Thank you so much

P: Thank you

I: The time is 15:08. Thank you

P: Thank you

End time: 15:08
