# Supplementary material for: Exploring perceptions of low risk behaviour and drivers to test for HIV among South African youth
Source: PLoS One. 2021 Jan 22;16(1):e0245542. doi: 10.1371/journal.pone.0245542 (PMC7822253; doi:10.1371/journal.pone.0245542)
Supplement: S1 File — (ZIP) [file pone.0245542.s001.zip › S1_File_Anonymised Transcripts/YA01-005-MT Translation_QC2_TM.docx]

Full Participant ID: YA01-005-MT

Participant Type: In-depth interview

Location: Winnie Mandela Clinic

Date:13 August 2018

Start time:

Primary interview language: Sepedi (English)

Name of Facilitator/Interviewer: Wellington Maruma

Name of Note Taker:

Name of Transcriber: Ornate Masuku

Length of recording: 41:53

Label Key

I = Interviewer

P = Participant

N = Notetaker

{ } = Indicates that details were changed or pseudonyms were used to anonymise data

xxx = words were omitted to anonymise data

- = breaking into a sentence by the next speaker

… = pause or drawn out words

[ ] = indicates noise made, e.g. [laugh], [sigh], [pause]

[Inaudible segment] = Unclear section of the recording

?Mulenga Clinic?, ?P3? = questionable text or doubt as to what was said or who said it

I: In-depth interview at {XXX} (Name of place where interview took place), participant ID is YA01-005-MT. The date is the 13^th^ of August 2018. The participant is female aged 21. Uuumh and the interviewer is {XXX} (interviewer name). Thank you so much for agreeing to be part of this interview, uumh do you allows me to record this this interview?

P: Yes

I: Thank you can you tell me what your thoughts are about HIV

P: …Ok, first of all uumh HIV like ages ago HIV was something we never thought would have a cure, like there was no way we could prevent it, it killed and killed and still kills lots of people but then these days there are doctors and medication to help prevent getting HIV. People can live more than 50 years even though they have HIV and you cannot see whether that person is HIV positive or not.

I: uuumh ok any other thing about HIV that you want to tell me about? How do you think people get infected by HIV?

P: Ok, the way I think about how people get infected by HIV, eemh sexual intercourse, someone can be HIV positive and you sleep with them without using a condom then you can be infected with HIV and sharing syringes you can one syringe with an HIV positive person and then you can get infected. Another thing through blood like a person can have a small scratch and then blood come while you are trying to help him or her then, without putting gloves on your hands you can also be infected.

I: uumh, ok personally have you been in a situation where you may be felt you were at risk of getting HIV? Have you been in a situation where you thought you are at risk of getting infected?

P: Yes, I might say.

I: ok, do you want to tell me more about that?

P: [giggles] ok it was, it was, I was in grade 12 2015, it was Sunday and I got raped by a person, that person that I trusted, he was like a brother and an uncle to me, but that time he had changed and I did not know what was happening with him, the worst part is that my parents didn’t know where I had gone to so I was afraid to tell them what had happened. Yea

I: Mum and how did you deal with that rape ordeal?

P: No, I just stayed like that with my heartbroken cause eemh I was raped on a Sunday and that Monday I was supposed to be writing Physics paper 2 and I did not pass so yea. So I actually repeated Matric because I was unable to do pass.

I: uumh, Ok sorry about that

P: Ok

I: ok and then can you tell me about HIV testing services that you know, like can you tell me more about any HIV testing services that you know of. Like testing services of HIV

I: Like you know at the clinic, those people that work in the tents, can you tell me anything about those HIV testing services that you know of?

P: Like I don’t understand

I: HIV testing services, like at the clinic you can test for HIV, even outside those people in tents test for HIV, there’s people walking around maybe coming to your house for HIV testing, right

P: Uuumh

I: can you like tell me more what do you understand about them?

P: Personally I don’t, for me those people that go around visiting people’s houses for HIV testing and those ones that outside. Like I don’t trust them that’s why I haven’t tested for HIV cause I was afraid

I: Why don’t you trust them?

P: Aah! People could be fraudulent and just take your blood

I: Uumh, so you think that they might use your blood for something else maybe?

P: Yes

I: Ok, and then why do you think so?

P: [giggles] Ok I don’t know why I think this way, but someone cannot just visit and test people’s blood and all that. Why don’t they give us a platform and make clinic appointments for us and say you come at this time to test

I: Uuumh, so getting tested at the clinic is better than being tested on the street?

P: Yes

I: Why is it better to test at the clinic? From your own opinion

P: personally I think it’s better to go to the clinic because you can trust people at the clinic, you can tell them stories, like even if you are positive there is confidentiality. But those that visit people’s houses to test you cannot trust them, you can enter a shop and find your picture with the caption this one is positive [giggles]

P: Yes

I: But was there ever a point where you wanted to get tested but you never did or something

P: Uumh

I: Ok, tell me more about that, like what happened that day?

P: Ok, like I have always wanted to go get tested cause my friends were always asking me why I don’t get tested and I always said I will go. They would go and get tested and I never went because I always thought how the person testing me would look at me if they find out that I am positive they will say such a young child with this thing

I: ouch ok, and then I think you mentioned that the clinic is better cause you feel like you can trust them unlike people that test outside in tents, anything else that is positive about testing at the clinic

P: …I don’t know [giggles]

I: …Anything anything positive or what are the benefits of testing at the clinic, you mentioned that you feel like you can trust them. What are the other benefits of testing at the clinic?

P: [sigh]

I: Ok, what is the bad thing about testing at the clinic?

P: Bad thing about testing at the clinic?

I: Uuumh

P: People’s eyes

I: People’s eyes?

P: Eye

I: Ok

P: Yea, going to the clinic chances are you will bump into someone that you know. If the person talks too much they can go around saying I saw such a person at the clinic so,

I: Uumh

P: I don’t know what they were doing there but they were behaving as if they were there to test

I: Uuumh

P: Cause if the person has no child it is obvious there are there to test

I: Uuumh, and then do you think the youth are given friendly HIV testing services or is it different. Or the youth and elders are tested differently? Like is it different for you when you come to the clinic, like how is it different for you as a youth compared to elders. Do you think you are both treated the same or not?

P: Not at all they don’t treat us the same

I: Why? How do they treat the youth?

P: [laughs] Ok you mean when you come to the clinic isn’t?

I: Uumh

P: Then you…

I: Like they, you know there are different age groups like there are elders, youths, kids and everything you think they treat you all the same

P: No

I: Ok tell me how they treat these ones this way and they treat these ones like this

P: They don’t treat the youth very well because they are children and the worst part is that they do not know how the person got infected. What if they got the infection through their parents, if that is the case they are not at fault, at the clinic they under estimate people you find them saying you like sleeping around that is why you are here, but with older people they don’t judge them cause they are much older.

I: Uuumh

P: Uumh

I: And then what do you think we can do so that the youth is treated better at the clinic?

P: They must know your status first

I: Uumh

P: After that they can ask what happened then you can explain that I was raped or you got via sexual intercourse is also fine, if you got it through your parents you are not at fault. Many kids know that they got eemh HIV through their parents.

I: Uumh

P: And they are not at fault we cannot blame them. You cannot say that since this child has HIV I cannot look after her nor do certain things for her.

I: Uuhm

P: Uumh

I: Ok uumh, isn’t I explained to you what incentives are?

P: Uumh

I: According to you what do you think they are? Explain to me in your own words what incentives could be

P: Uumh it is something that could encourage the youth, the youth to come test for the thing. But you know us youth we do something when we know we will get something in return. I cannot just leave my things and come here for you to only say bye bye when I leave, no I want something.

I: Uumh

P: I want something in return

I: And then what is that something that you actually want? Give me an idea that can make you come to test isn’t you haven’t tested before like what incentive can we give you or one that can work for the youth so that they come test.

P: It can be money, it can be t-shirts, it can be bottles, and it can be food yea.

I: When talking about money how much do you think it should be?

P: … [Laughs] eh! Maybe R150

I: Ok why do you think R150 is better than R20?

P: No ways [laughs] not with someone’s blood

I: Uumh

P: Not with R20 for someone’s blood, if I used three taxis to come here and you only give me R20 it’s obvious that I will use R10 from one location to the other, then another R10 and another R10, so what will not make me leave my house cause I would have used R30 to get here and for you to only give me R20.

I: You mentioned t-shirts, bottles, food. On the t-shirt give me an idea on what kind of t-shirt it should be what should and should not be written on the t-shirt. Give me an idea of what kind of t-shirt will appear to you?

P: Uumh

I: What kind of t-shirt will appeal to you? What colour?

P: Red and white or red and black

I: Ok, why those colours?

P: …The t-shirt what can I say, what you wear obviously attracts people. As you know the colour of blood is red so if it will be designed in a way that is attractive and customised. People will be curious and start asking where you got that t-shirt.

I: Uumh

P: Yea

I: Ok and then will something be written on the t-shirt or it will be a plain red and white t-shirt?

P: … [Sighs] I don’t know

I: Huh? Visualize it, like let’s say I am about to give you that t-shirt

P: Uumh

I: What kind is it? I can already see it because you said it’s red and black, what would be in front? What would be at the back? Like give me an idea what that t-shirt would look like.

P: …Aai

I: Mmh? Like this one I am wearing is red.

P: Uumh

I: But you can see something is written on it.

P: Eeh

I: So the one that we give you what will be written on it? Any message that you can think of maybe?

P: Maybe…[sighs]

I: Ok, the bottles that you mentioned how would they look like?

P:…Just normal bottles

I: Ok

P: Yea

I: But then that and that and that and that are 4 different bottles

P: Oh no! They must be nice [laughs]

I: Ok

P: Uou cannot bring still water bottles and expect to give them to people.

I: Ok

P: They can say we can get these bottles on the side of the road.

I: That’s what I am trying to find out from you, when you say bottles I can bring bottle and those will not encourage the youth to come test. Like what would that bottle look like for you? Does it have a certain design? Does it have a certain message on it? What size? Give me an idea what it would look like?

P: It can look like yours but in a red and black colour. Same size same shape so

I: Uumh ok. And then food? Like

P: Maybe… maybe what can I say though…can be sandwich

I: Uumh

P: Uuuumh… sandwich?

I: So you mentioned money, bottles and food. What else can be done to encourage the youth that they go test. Isn’t if people are given money they will come

P: Uumh

I: If we give them t-shirts, bottles and food they can come.

P: …

I: Mmh?

P: Maybe a Tablet.

I: Tablet ok, what else?

P: …

I: Mmh? Ok so out of the 5 things you mentioned

P: Uumh

I: What, what do you think if I was to tell you out of the 5 things you mentioned I can only give you one and not all of them what would you chose first.

P: Its money

I: Ok, and the secondly what would you chose?

P: The bottle

I: And the third thing you would choose?

P: Tablet [laughs]

I: The fourth thing you would choose?

P: T-shirt

I: Then lastly?

P: Food

I: Ok, why did you choose money first?

P: [laughs] Money

I: Uumh

P: Ok money is the first cause I am thinking ahead like you would need transport to go back when you are coming from, and what you will eat.

I: Uumh

P: So if I am to choose a t-shirt, this t-shirt will not take me back to where I am coming from

I: Uumh

P: Yea

I: Ok, and then do you think these things that you mentioned

P: Uumh

I: Like should it be different for the youth and older people or these things can only work for the youth only. Or there will be things that will work for the adults that is different from the youth.

P: As for t-shirts, bottles food and tablet will work for the youth only

I: Uumh, why do you think that is?

P: [laughs] Like us youth we like things

I: Uumh

P: Yea there isn’t an adult that will leave their house and come here for t-shirt only, no

I: Uumh

P: They won’t come they will just stay there. But you were to get to their homes and tell them you are here to test them for whatever they will just give you their hands and let you test them and won’t ask much questions

I: Ok

P: Yea

I: So you think these things will be more effective for the youth?

P: Uumh

I: Ok. So let’s say we give you these what do you think the challenges will be. Do you think giving people these things to try encourage they will come test or there might be other challenges?

P: Yea if we give people these things they will definitely come test.

I: Uumh

P: Yea

I: And then the challenges of giving people things so that they come test, what do you think the challenge will be?

P: …Like

I: Like if I don’t give you these things you won’t come test isn’t

P: Uumh

I: And that’s already a challenge because it means you are only coming for the incentives and if they are not offered the incentives you won’t come test

P: Uumh

I: What do you think are the challenges that can come up?

P: Ok as I said that I was afraid to come test, but then if they offered something like a cell phone when you come test it would encourage me

I: Uumh

P: Like if I use a small phone and here they give proper phones like Samsung

I: Uuumh

P: Obvious I will come for testing

I: Uumh

P: But then it will be challenging if one comes to test and they find out that they are positive. One will forget about the Samsung and focus on their…

I: You then mentioned something interesting that maybe if you give people phones. Is that something that you think can be added to the list of things that you ordered?

P: Yes

I: Ok why?

P: [laughs] As you know that phones are expensive

I: Uumh

P: They are really expensive

I: Uumh

P: Very very expensive, there are a few jobs and are scarce you can have a degree and still stay at home. So if you aren’t working where would you have gotten this phone?

I: uumh, ok so let’s talk about phones since we are on that topic. How do you think a phone can be used to disseminate information about HIV testing?

P: …Uumh maybe you send, eemh there is this thing I don’t know it’s from Vodacom or what they send messages of questions and they say you must answer and that is free of charge. So I think that would be something that works for the youth.

I: Uumh, you said Vodacom do you know the name of that thing you are talking about?

P: No I don’t know the name but I have it on my phone. Maybe they ask you questions like what you won regarding soccer things but it is a free service they send you messages and the reply is free, as soon as you answer they send another message until its complete and they can say you are done with today’s game wait for tomorrow.

I: Mmmhm. So after you answer those questions you win something? Is it like a competition?

P: Yea like a small competition

I: Ok interesting. And then what type of questions would you want to get with regards to HIV? Let’s say we adopt this same thing of Vodacom what type of questions would you want to get of?

P: HIV?

I: Uumh

P: Ok maybe you can ask when last I tested. Then maybe ask about my sexual intercourse …eeehm eeemh how does HIV affect people and how to treat people living with HIV?

I: Uumh, ok

P: Yea

I: Ok interesting. And then how do you think your parents will react when they see you receive these types of messages? How do you think they will react seeing these HIV messages?

P: They will be happy

I: Ok why?

P: Ok these days isn’t our parents are afraid to talk to us. Like at times we make mistakes but we don’t realise that it is a mistake because our parents don’t talk to us and we think as long as my mother has not said anything this is the right thing to do. They are afraid to talk to us, for instance with girls when we get to the menstruating phase my mother is supposed to sit down with me when I am young and explain what menstruation is that it is a natural thing for woman and help me understand period pains so that you don’t go to the hospital for mere period pains

I: Uumh

P: If you are menstruating it means that the chances of falling pregnant are high. Our parents are afraid to talk to us so if they start seeing that we receive messages like these of HIV and all I think they will be happy.

I: Uumh

P: Yea

I: Ok like persornsally would you want to be part of a similar program like the one of Vodacom, like something similar to{XXX} (Name of cellphone service provider). Would you want to get those messages about HIV?

P: Yes I do

I: You would want to

P: Yes

I: Ok and eemh so you talked about this sms. Like it comes as an sms right on your phone?

P: Yea like sms comes with questions

I: It comes with questions

P: Yea after the questions that like after the question you find multiple answers like four different answers and three of them are incorrect and the one is correct. So you chose one answer if it’s correct they say congratulations and if it is incorrect they reply oops you will try again tomorrow.

I: Ok

P: Yea

I: aAnd then what kind of things do you win on this thing?

P: Tablet

I: Oh!

P: They once offered it to me but I failed to reach the stage they wanted so that I could win the tablet

I: Ok

P: Mmh

I: Ok, so that is something that could encourage you to go

P: Yea

I: Ok so above eemh, other than the sms and everything else how else do you think we can use phones to get youth into this whole thing to get tested

P: Social media

I: Social media ok

P: It can be twitter, facebook, instagram and youtube

I: Uumh

P: Yea

I: Ok and then how would we use twitter. Just how you would want to get information about HIV testing via twitter?

P: …

I: Like how would you get information via twitter? How would you get information using twitter?

P: …Maybe you can ,maybe post something on twitter and you will see that I am interested if I start following everything that you post and I make comments and request more information.

I: Uumh and then how about face book?

P: You can open a page

I: Open a page and this page would be related to HIV right?

P: Yes

I: And the type of post you would want to receive or what kind of posts would those be?

P: Type of post? Ok …ummh it can be syringes, you can post condoms, eeemh you can put people who are infected with HIV.

I: Uumh

P: Yea

I: Ok. And then how do you think that will help like posting people that are infected by HIV on face book for example? Do you think these would encourage someone to get tested?

P: Yea cause like you know like HIV I don’t know how to put it is a bit unpredictable and does not affect us all the same.

I: Uumh

P: Some start with vomiting, yea then another their body changes and they start losing hair and all that then others start having sores, let’s say maybe you posted someone with HIV and they have sore or something and are looking rowdy. If I have never tested I would have that thing that what if I am positive and will develop that thing rather I go get tested.

I: Ouch

P: Yea

I: Ok and then what about YouTube?

P: …YouTube maybe in a form of a video

I: Uumh

P: Yea

I: This video, give me an idea of what would be in that video.

P: …Ok maybe first of all like you can start with the meaning of HIV, how it affects people, how can you prevent it and how to treat it.

I: Uumh, ok and then what about instagram? Because you mentioned twitter, facebook, YouTube and instagram right?

P: Uumh, also instagram you can also open a page yea.

I: Ok and which one of the social media platforms will be more effective in reaching the youth?

P: Facebook

I: Facebook ok, why do you think so?

P: [laughs] Like young people love facebook

I: Uumh

P: Like a lot than the other social media platforms we all love facebook, like the only thing about facebook is accepting friend requests from people you don’t know and you start talking privately you don’t know who this person is and you exchange pictures without knowing who this person really is and you become interested in the guy/girl but you do not know what their intentions were.

I: Ok. So you think ummh facebook will be more effective?

P: Yes

I: Compared to the other social media platforms

P: Uumh

I: And then the challenges of using social media what do you think the challenges are in terms of relating this information to the youth

P: The challenges

I: Uumh

P: How?

I: Like other people don’t have phones

P: Uumh

I: So that’s a challenge already …So it means a person with no phone will not see the messages on facebook, twitter, YouTube and instagram. What other challenges do you think there might be?

P: …Data

I: Data ok

P: Ummh cause we don’t always have data and all social media platforms require data, that’s why I said facebook is better cause there is free mode. Yea so maybe let’s say you have 10mb you can view that video and like it, if it finishes you can use free mode and comment

I: Ok so data is something that can be added to the list of incentives as well?

P: [laughs] Yes

I: Ok, like how much data would you need to get tested much?

P: 1 GB

I: 1 GB ok [laughs] you think that it will be enough?

P: Uumh

I: Why do you think that why not 500mb or 20mb or 3 GB. Why do you think 1 GB is going to be enough?

P: Aaah! I think 1 GB will be enough because of the smart phones we use

I: Ummh

P: Like 500mb finishes in 2 days for the smart phones we use

I: Uumh

P: So then 1 GB I think will do.

I: Ok, and so you mentioned we can use something similar to the Vodacom like sums right,

P: Uuumh

I: Social media any other ways we can use phone to encourage the youth

P: Emails

I: Emails, ok

P: Yes

I: And then emails of it, who would have sent them? Where would they be coming from? Who would be sending these emails you are talking about?

P: Who will send?

I: Uumh, or like who will you receive this email from or from where?

P: …Like I don’t understand

I: So you receive an email right now who would it be from?

P: Oh! Coming from whom?

I: Uumh

P: Maybe you guys from {XXX} (Name of non-profit organization)what, it can be from {XXX} (Study team member)somewhere there [laughs]

I: [laughs] Ok

P: Uumh

I: What would be in this email give me like a brief thing, message like something that you would want to be in that email, so that when you read it you are encouraged to go test.

P: Ok maybe ask when you last tested obviously these are people you know and trust they can proceed to ask how it went when you last tested and what your status was and are you happy about your status or

I: Mmhm ok I like that. Do you have any final thoughts about HIV, youth and, the incentives anything you forgot to mention, final thoughts

P: What I want to say is like I want encourage you that we go and get tested because you cannot trust someone completely even if you use condoms that should not prevent you from going to test.

I: Uumh

P: Like you can sleep with someone and use a condom but then you get infected and you don’t know how. So the only thing that I can say is we must go and get tested even though there is money, t-shirts, tablet and cell phone you must get tested for the sake of your own life.

I: Ok yea that’s good

P: Yea

I: So you are saying that the youth should just be encouraged to get tested even without those things that you mentioned.

P: Yea

I: Ok

P: Yea cause like life cannot be compared to receiving a tablet, because my life is worth more than a tablet. So I cannot say that I will not get tested cause I will not receive a tablet I will not put my life at risk like that. The youth these days people test so that they can receive tablets and t-shirts.

I: Yea

P: We don’t care about our lives

I: Uumh ok and then uumh let’s go back to the incentives.

P: Uumh

I: So in terms of money you said R150 or you said it will be enough.

P: Yes

I: And t-shirts and tablets what not, how often do you think you should get these things for coming to test. Should they be provided every time you come test or once every amount of month?

P: Maybe once

I: Once ok and then do you think if I only give you once you will come back again after three or four months?

P: Yes, I will come back because the last time I got a t-shirt that means I will also get one today. And then when you when you finish testing you will be told today we are testing only no incentives.

I: And then do you think you will come a third time?

P: Yes I will come.

I: Even if we didn’t give you the second time around?

P: Uumh [laugh]

I: Ok and we are almost at the end of our discussion and I would like to thank you for your participation.

P: Ok

I: Is there anything else that you can think of, anything on your mind?

P: …No [laughs] I just want to encourage the youth to go and test for the sake of their lives

I: Uumh

P: Not for t-shirts but for the sake of their lives, they must test for the sake of their lives, if they are positive they can get treatment quickly, people now live a long time with HIV.

I: Uumh ok, so social media you mentioned facebook, twitter, YouTube and instagram what else

P:…

I: Or out of all of those which one do you use the most

P: Facebook yea and whatsapp.

I: Oh ok

P: Whatsapp depends whether you have someone’s number or not, you cannot receive messages from people that don’t have your number

I: Ouch

P: So and whatsapp will be more useful like if we create a group for those things.

I: Uumh

P: Yea all the information will be found on the group.

I: Ok on the whatsapp group who would be the admin? Would be among your friends? Or would it be some from the clinic and they add people to the group as they come to the clinic. Or would it be among your friends on the whatsapp group?

P: No someone from the clinic, cause if like it’s among friends and I am the group admin if we get bored we can talk about other things. But if the group admin is from the clinic or {XXX} (Name of non-profit organization) it will be fine. Whoever will create this group will announce that the purpose of the group is to talk about things related to HIV not anything else so that we don’t waste our data

I: Ok thank you this is the end of our interview and I will just like to thank you again for being part of this interview and thank you.

P: [laughs]

I: The time is 11:28. Thank you so much

P: Ok

End time: 11:28
