# Supplementary material for: Exploring perceptions of low risk behaviour and drivers to test for HIV among South African youth
Source: PLoS One. 2021 Jan 22;16(1):e0245542. doi: 10.1371/journal.pone.0245542 (PMC7822253; doi:10.1371/journal.pone.0245542)
Supplement: S1 File — (ZIP) [file pone.0245542.s001.zip › S1_File_Anonymised Transcripts/YA01-011-BK_Transcription_QC2_TM.docx]

Full Participant ID: YA-01-011-BK

Participant Type: Female , 18 years old

Location: Winnie Mandela Clinic

Date: 23 August 2018

Start time: 14:53

Primary interview language: English

Name of Facilitator/Interviewer:

Name of Note Taker:

Name of Transcriber:

Length of recording: 33:21

Label Key

I = Interviewer

P = Participant

N = Notetaker

{ } = Indicates that details were changed or pseudonyms were used to anonymise data

xxx = words were omitted to anonymise data

- = breaking into a sentence by the next speaker

… = pause or drawn out words

[ ] = indicates noise made, e.g. [laugh], [sigh], [pause]

[inaudible segment] = Unclear section of the recording

?Mulenga Clinic?, ?P3? = questionable text or doubt as to what was said or who said it

I: In-depth interview, uhm the date is the twenty-third of August. Participant ID is YA-01-011-BK and the participant is eighteen-years old, female at the {XXX} (Name of place where interview took place). Thank you so much for being a part of this interview. Uhm [tongue sound], would you allow me to record this interview?

P: Yes.

I: Okay, so we can get started. Uhm, can you tell me what you think…like what are your thoughts on HIV?

P: Uhm…HIV? -

I: Yeah, what is HIV? What are your thoughts on HIV?

P: Uhh, HIV is a virus… Uhh that damages…your…uhh internal system. Yah.

I: Mhmm… What else do you know about HIV?

P: That, uhh…HIV, its not curable.

I: Mhmm…

P: HIV/AIDS, uhh…there are treatments for it.

I: Mhmm…

P: You cannot get affected if you don’t touch, uh, another person’s blood. Uhh, you don’t get, uhh…uhh intimidate with the person who is HIV positive. And uhh, it can also be transferred by breastfeeding - mother to child.

I: Mhmm… Uhm, can you tell me like, about places that a person can feel at risk of HIV?

P: Uhh, when you having unprotected sex?

I: Mhmm

P: Hmm, touching, uhh, another person’s wound…who is HIV positive while you also wounded… And then, also mother to child breast feeding process.

I: Okay. And, can, was there ever a situation where you felt you might have been at risk of contracting HIV?

P: No.

I: Ever? -

P: - Never

I: Really?

P: Yes [laugh]

I: [laugh] Okay [inhale], and can you tell me about HIV testing services that take place in the community?

P: I often see, uhh, people…uhh asking, uhh, people who are around to come and test. So that they can know their status.

I: Mhmm

P: Yah.

I: Is that the only places you know where people can get tested?

P: You can also get tested at the clinics…

I: Mhmm…

P: … Yah [laugh], that’s all.

I: Okay, but you’ve never has an experience of HIV testing?

P: No…

I: Why not?

P: I never thought of it…

I: You don’t wanna know your status?

P: [sigh]

I: You don’t think its important to know your status?

P: Yah, I think its important to know your status…

I: Mhmm…

P: …But I never thought of it [laugh].

I: But is that something you’d, you’re thinking about now, maybe?

P: Yah, its something that I’m considering.

I: Okay and what would make you go and tested?

P: …

I: Now that you’ve never thought about it, what would make you go get tested?

P: After you have, uhh, explained what the study is about…

I: Mhmm…

P: This, pulled my interest into it, so... its something that would make me go and get tested.

I: Yeah, but had it, had it not been for the study, would you not want to get tested? Is it something you never thought about?

P: No, I never thought about it.

I: Okay. Uhm can you tell me about, also you said you never acc-, access these HIV testing services?

P: Mhm.

I: How do you think these HIV testing services can be made…accessible for youth? Maybe age between fifteen and twenty-four?

P: They can bring, uhh, this programmes at schools.

I: Okay.

P: Because that’s where we find, uhh, the age group. Then at higher institutions, like varsities…uhh-

I: -Mhmm

P: We have a certain day, every month, where youth get tested… Yah

I: Mhmm…

P: And it can also be added in…the syllabus, studying syllabus.

I: Mhmm…

P: Mhmm, where they teach, all the grades that testing is very important.

I: Okay. And what do you think are the positive things about the current HIV testing services? Like the ones that you mentioned, you know in the streets, you did mention that there was people that –

P: Mhmm

I: What do you think is positive about that? Like had reaching people in those initiatives?

P: Can you please repeat?

I: Okay, what do you think is a good thing about reaching people, like in the streets or in the clinic or…, for HIV testing services?

P: So that people can know their statuses.

I: Mhmm…

P: Mhm. And, uhh, that people after knowing their statuses, if you have HIV, you can start taking the treatment and if not, and, you were in, uhh for example. You were having unprotected sex and you don’t have it, you can now protect yourself.

I: Mhmm… Okay and what do you think are the bad things about, maybe, any challenges you think there might be in these HIV testing services that you mentioned?

P: Uhh people will see you when you go there [laugh].

I: Okay and, so you’re worried about confidentiality?

P: Yes.

I: What else…do you think would be a challenge?

P: Uhh…another challenge?

I: Mhmm…

P: Can be that, uh, maybe sometimes you’re not ust the only person in the room. Theres another one. So obviously…they are going to hear your status. And its no longer a secret.

I: Mhmm…okay. So you’re worried more, more about…uhm, confidentiality?

P: Yes.

I: What else do you think might be a chanllenge with the HIV testing services? Particularly to the youth.

P: A challenge?

I: Mhmm…

P: Maybe, uh, as young people we often scared to go and know our statuses.

I: Mhmm…

P: So I think that would, can be a very big challenge.

I: And do you think that is? Why do you, why do you think the youth, uhh…aren’t interested in knowing their status or are, you said scared, are they scared? Why do you think their scared to know their statuses?

P: Because most of the time, uh, the, they are putting their lives at risks.

I: Mhmm…

P: Mhm.

I: Mhmm…

P: Having unprotected sex…then, uh…when they are using drugs, that bluetooth thing. When they exchanging, the exchange of blood, yah.

I: Mhmm…okay. So can you describe to me what you think, so what do you think comes to mind when I mention, when I say incentives? What do you think incentives are?

P: Incentives?

I: Mhmm…

P: Incentives is, uhh…when you do something, you expect something in return which is more than what you did.

I: Okay. Can you explain that further? Give me eamples maybe?

P: For example, when you working…

I: Mhmm…

P: You expect a good salary or a promotion.

I: Okay.

P: Mhm.

I: Okay. So, lets think about this in the context of, uhm, HIV testing services amongst youth, right?

P: Mhm.

I: So, uhh, what type of incentives do you think the youth could value? That would make them, to come and get tested for HIV or to access HIV treatment?

P: Uh, after testing, I think there must be programmes that encourage youth to encourage others to come and test. Not just once but maybe after a period of a month or so…

I: Mhmm…

P: Uhh and maybe freebies…tees, caps, yah.

I: Mhmm… What else?

P: Another thing?

I: Mhmm…

P: Maybe uh this tested, uhh, we can find people who are young to conduct them.

I: Mhmm… Okay, carry on…

P: [laughs] Uh, another thing is… uh, find a place where you know for sure that, uh, your confidentiality wont be re-, realed, revealed.

I: Mhmm…

P: Mhm.

I: Okay so you’ve mentioned that…things like freebeies, as you mentioned, as you said, you know like t-shirts, caps…uh, are something that could, uh, incentifies youth to come and get tested. Why do you think those things are very important, why do you think incentives are important for encouraging you, the youth to come and get tested?

P: Everyone who want to do something and get another thing in return so, I think, when you giving them. You attract them by giving them free caps and free t-shirts.

I: Mhmm…

P: Yeah that’s where the number of people who are coming, who will come test will be…-

I: Okay-

P: -High

I: So uh would that be something that would encourage you to come and get tested since you’re not tested before. Like if I were to give a T-shirt right now, would that encourage you or is there something else that would encourage you to come and get tested?

P: [laugh] Mhmm… yah, another thing, not with freebies then [laugh], maybe a programme where after testing, yah, there’ll be a programme where both people who are HIV and those who are not will attend. We don’t like, uh, solidify, uh people who are HIV attend such programmes and people who are not; no like we just attend like the same programme.

I: Mhmm…

P: Yes.

I: Okay. So, uhm, You’ve mentioned programmes, t-shirts, caps. So with, like can you describe to be what like, these t-shirts would look like?

P: These T-shirts?

I: Yeah, how would...yeah

P: Uhh, we can have…pink and blue t-shirts for boys and girls.

I: Mhmm…

P: Written ‘know your status’, uh, ‘get tested’…yah.

I: Mhmm…

P: Mhm.

I: And the caps?

P: And the caps… Uh… the caps?

I: Mhmm…

P: ‘Prevention is better than cure’, especially when there is no cure. ‘Get tested, know your status’.

I: I like that [laugh]

P: [laugh]

I: Okay so, do you think t-shirts and caps will be enough to draw in young people, like the youth to come and get tested? I mean those have been done before…

P: No…

I: Okay what else do you think…-

P: Maybe- [exhale] ahhhh [laugh]

I: Yeah [laugh] okay

P: [laugh] Maybe a stationery pack…

I: Mhmm…

P: Because we are trying to attract youth.

I: Okay…

P: Yah.

I: What else?

P: And, another thing… We can also have bottles and lunch boxes. Mhm.

I: Mhmm… Anything else [laugh]

P: [laugh] No, that’s all [laugh]

I: Okay so you’ve mentioned t-shirts, caps, stationery packs, bottles, lunch boxes, right?

P: Mhm.

I: Uh which of these things do you think will be more important to you? Cause you did say if I were to give you a t-shirt right now, it would not be something that would encourage you to comeand get tested.

P: Mhm.

I: What of these things would make you, would encourage you to come and get tested? What would you value more?

P: The stationery pack.

I: Why?

P: Because…most of the time, uh, during exams, you’d find people, asking another person for a pen to write.

I: Mhmm…

P: So I think lack of stationery in schools will make people to go out there and get tested. So that they can get the free one instead of buying.

I: Mhm... Okay and the next thing on that list, would be what?

P: The next thing?

I: Uhh

P: On that list? Uh, can I also add?

I: Okay. No problem. [laugh]

P: [laugh] Uh, okay. They can also give people contraceptives.

I: Mhmm…

P: Then they can also give girls…uh, sanitary towels.

I: Mhmm…

P: Yah.

I: Okay.

P: Then I think those sanitary towels will be the next thing, I go there.

I: Okay, so that will be the second thing from the stationery pack?

P: Stationery pack, yes.

I: Okay and why is that?

P: Uh, why is that?

I: Mhmm…

P: Sanitary towels…most of the time are on sale so when, someone offers you to give you for free, that’s obviously, that’s where you’d go. Because of, uh, if you don’t have them, that means, uh, some of the kids don’t come to school because of they don’t have sanitary towels.

I: Mhmm…

P: So I think, if they were to be given for free, they’d come and get tested.

I: You think so?

P: Yes.

I: Okay… So another thing from that list would be? So we are now ranking them in order right?

P: Okay.

I: So uh, the first one would be stationery pack, second one would be sanitary towels for the girls.

P: Mhmm…

I: The third one would be…?

P: Uh, programme.

I: The programmes?

P: Yes.

I: Okay. Can, can you just take me through these programmes maybe? What type of programme do you think would encourage…

P: We can have a programme, uh, which is conducted by youth.

I: Mhmm…

P: Where, uh, we discuss issues, uh…of HIV/AIDS and other uh, sexual transmitted infections.

I: Mhmm…

P: Uh then, we discuss a way forward, we look at a practical solutions; not the problems.

I: Mhmm…

P: Mhm. If someone, uh, has HIV…uh, is positive, HIV positive.

I: Mhmm…

P: We looking at the way forward. Yes, its not curable but there’s a treatment and how are you going to take the treatment and look for other ways; you can exercise and yah…

I: Mhmm…okay I like that actually. So, uhm…the, the freebies that you mentioned like the t-shirt, caps, bottles, stationery packs, lunch boxes and all these things that you mentioned…

P: Mhm.

I: How often do you think these should, should be provided? Uh, would that be a once-off…when you come, like when you come and get tested? Would that be once off or…-

P: -The t-shirts and caps…

I: Mhmm…

P: I think they should be once off.

I: Okay. And then the rest?

P: Uh, even the lunch boxes, they can be a once off thing. But the sanitary towels…every month.

I: Okay.

P: If its possible.

I: Okay.

P: Mhm.

I: So does, are you saying that like with the sanitary towels ok, would that, would we then, like would we then require girls to come and get tested every month, for them to come and get it or…?

P: Yes, they can come get tested every month so that they can know their statuses and in return they will get those sanitary towels.

I: Mhmm…

P: Yes.

I: And what do you think the problem with, with uhm…providing free uhm, in- in- incentives once off would be? So if we were only to give you, uhh like you said t-shirt and caps so we can give it to you once off, right?

P: Yes.

I: What do you think the challenges of that would be?

P: Uh, the, the following, uh for example the following session of the testing; people won’t be, uh, a lot. Like they were before. Because of there are no longer free things.

I: Mhmm… And how do you think we can like go around that challenge? Just to make sure that we stil have the…same number of people that are coming with or without the incentives maybe?

P: Uhh, we can have the same number of people? That’s why I say, uh, there should be an invention of programmes. Mhm.

I: Mhmm…

P: Where people, uh, that’s why I was talking about addressing issues and coming up with practical solutions. Obviously everyone want to know; if I’m infected, whats the solution? Or whats the thing that I have to do…?

I: Mhmm…

P: To…get better.

I: Yeah.

P: So if we spoke about excercises the…previous week…

I: Mhmm…

P: Definitely the other session would be talking about taking your treatment regularly, going to the clinic…yes.

I: Mhmm… Okay. Uhm, and what do you think are the benefits of providing these service-, these incentives? So you did mention one of them being that will have a lot of people coming in, because of we’re providing these things…?

P: Yes.

I: What is the other benefit do you think there might be for providing these things?

P: People will know their statuses…

I: Mhmm…

P: And another one…uhh [pause] you becoming more knowing your statuses. Uh another thing…?

I: Mhmm…

P: Mhmmm [pause], people uh will bring others…to come, and participate in such…studies.

I: Mhmm…

P: Mhm.

I: Okay. Uhm you did say that, so, okay so let’s say these programmes that you’re mentioning right?

P: Mhmm…

I: Uhm the different session that you, uh the different sessions that would be targeted or that would be taught about in the programmes…

P: Mhm.

I: You said maybe for example it could be how to get tested, mhmm where to get tested, uhm…how to take your treatment you said right?

P: Yes.

I: How else do you think we can get that information into the youth?

P: Uhh…we can also create an app. Because most of the time, youth spend most of their time on social media where we have a Facebook page where we discuss such issues. We can also have an app alone…mhm that discuss such issues.

I: Okay. And what would be in this type of messages you would, okay you mentioned social media like Facebook page…?

P: Mhm.

I: What type of messages would be there? Like give me an example of maybe a message that you’d wanna see on Facebook, a message that would encourage, like, like when you see it, like okay maybe its time for me to go and tested.

P: [pause] Uhh, ‘know your status, you only live once’.

I: Mhmm… Okay, and you uhm, is that, would that only be on Facebook or any other social media you can think of?

P: Uh…Facebook, and also Twitter. I think it’s the top social medias that youth interact on.

I: Mhmm…

P: Yes.

I: Okay. Theres no other social medias that you can think of maybe? [pause] Which one are you active on?

P: Mostly?

I: Mhmm…

P: Whatsapp [laugh]

I: Whatsapp?

P: Yes.

I: And you don’t think we can get information through there as well?

P: [sigh] Yes, yah we can get information through Whatsapp.

I: Mhmm…

P: Whereby there will be a groupchat.

I: Mhmm… And that group chat will be facilitated by… You?

P: Yah I can [laugh]. Uhh will be facilitated by…yah, I can also facilitate it. [laugh]

I: [laugh]

P: And uh other people from the study… Mhm.

I: Mhmm… Okay and what do you think the problems are like, with using social media to many, to give people this type of HIV testing information? Especially for the youth. The challenges with that.

P: Someone can hack your phone! And, uh, go through your confidential information so its an, a disadvantage.

I: Mhmm… Okay, what else?

P: Your phone can be stolen and people can go through it.

I: Mhmm…

P: Mhm.

I: Okay, and… What about, uhm [pause], so what about youth that do not have access to cellphones or do not have access to social media maybe? How else do you think we can get like these, info-, like uhm HIV testing services, like information about HIV testing services to them? Yeah…

P: Uhh, maybe, uh…in a month we can have a meeting where youth come and interact with the issues.

I: Mhmm…

P: And, uhh…maybe sometime they can have one session with, [dogs barking] uhh, with people who test them or proffessionals.

I: Mhmm…

P: Mhm.

I: Okay, can you go back to the app that you mentioned?

P: Mhm.

I: Right. So…uhh, can you just tell me more about it, what would the app be like? What type of information would be there? How would you interact with app? Would it be the same thing as something that’s already currently happening maybe that you can think of?

P: The app can be the same thing as Facebook, but now we on a different matter where we’re posting about our health.

I: Mhmm…

P: Yah. We get messages, uh, on how to…on how to…uh, challenge, to get through the challenges of risky behaviour.

I: Mhmm…

P: Yes.

I: Okay. Have you used thay USSD thing? On cellphones? You know like star-one-four- what what and you get; maybe like star-one fourty and you get your airtime balance or star what what what…?

P: No

I: Okay. Have you received any health information maybe through SMS maybe?

P: No.

I: Okay. How else do you think we can use a cellphone to, to, to [pause] to uhm, to get HIV testing services? To get testing, HIV testing information?

P: [pause] Uhhh, how else…?

I: Yes, you mentioned we could use an app, social media; how else do you think –

P: -Ohh, uh, maybe, uh…uh phone numbers. And contact details can be collected from each school or other institutions.

I: Mhmm…

P: Whereby, uh, each and every individual will be called and be informed…about the testing.

I: Okay.

P: Yes. And SMSes.

I: Okay… Okay. That’s great. And then…so uhm, and how do you think your parents would feel if you were to get like HIV testing information on, on your cellphone? Maybe information regarding HIV, how do you think they would feel?

P: Uhh, my parents…?

I: -Whoever you stay with

P: [laugh] Uh, I think they will feel…they will feel good. Because I’m finally taking responsibility, I’m not waiting for them to tell me, uh go and get tested; know your status or wait till you are old. Uh, its a sign of maturance.

I: Mhmm… Okay. [clears throat] And why do you think some parents would not be receptive of this idea of having their kids receiving information about HIV? Or go and get tested, do this?

P: Uh, some parents, uh, uh…for example.

I: Mhmm…

P: If…a parent knows that, uh, the child is already HIV positive and they don’t do anything about that. The parents, uh, wil feel uh…how can I put this? [pause] [laugh]

I: Take your time…

P: Uh, will feel underestimated; will feel little and will feel uh, very frightened that the child will finally know their status.

I: Mhmm…

P: Mhm.

I: Okay. Can you also give me any other suggestions that you might have…on ways we can encourage {the youth to come and get tested for HIV} [translated from Setswana]. You’ve mentioned a lot actually like programmes, uhm an app, Facebook, maybe even having people go to the varsities or schools; or maybe having HIV testing services introduced into sylla-, I mean information being introduced in syllabuses. Right?

I: [laugh] Yah.

I: …For schools and also providing incentives such as t-shirts, caps, stationery packs, bottle, lunch boxes and all those-, do you have any other suggestions?

P: [pause] Mhmm… We…can have posters and flyers, pamphlets [pause]. Informing youth about testing, uh, so that they can come and know their statuses.

I: Mhmm…

P: Mhm.

I: But that is already being done, don’t you think? Its already being overdone…is there any other way maybe? Like any creative way maybe that you can think of…? Cause there are pamphlets all over the clinic here that you’ve seen I’m sure about HIV testing. But we’re still not seeing a lot of youth that are taking action, to come, to come and get tested. You have seen those pamhlets right?

P: Yes. Uh…how about an event?

I: Okay.

P: Like those top events on TV (television), ‘Come Duze 2018’ and so ever. We can have programmes like that.

I: Mhmm…

P: Whereby we bring celebrities to come and perform and…uh, people will come in massive numbers, will come and get tested.

I: Mhmm…

P: Mhm.

I: But don’t you think they will be coming there for the party, {just}? Not for the actual testing? Cause you know the focus here is to get them to get tested and access HIV testing; access treatment services.

P: [pause] They will not be coming only for the parties but, uh, at the end of the day they’ll be knowing their statuses.

I: Mhmm…

P: And, uh, the music can be related to knowing your status; it can also be poetry…yes.

I: Mhmm… Okay. Any other final thoughts that you have about youth? How we can get them to get tested?

P: [pause] Uhh…us! Youth…

I: Mhmm…

P: We can also encourage others by telling them how good is it to get tested. Yah.

I: Mhmm…

P: I think what you hear from your peers, yah, will-, you will get it much better than from other people who are older than you.

I: Mhmm…

P: So if your age group have experienced something, you will also want to go there. And…get tested.

I: Yeah… do you have nay friends that have tested for HIV before?

P: No.

I: No-No? Okay, so you think if they have tested for, if they had tested for HIV and they told you about that, would you…have tested gone, get tested as well?

P: Yes.

I: Really? [laughs]

P: Yes [laughs]

I: Okay. Okay so uhm, any other final thoughts about incentives, the ones that you mentioned? Do you wanna add on maybe? Something that came to mind maybe now?

P: Mhmm No [laughs]

I: Nothing?

P: Yes…

I: Okay. Okay so you said that the most important, so the top three incentives that were important to you...

P: Mhm,

I: You said were providing stationery pack, uhm…providing sanitary towels to the girls or having these programmes right?

P: Yes.

I: So see that I removed all these three from this list, okay?

P: Mhmm…

I: Uhm, would you still wanna get tested if I were to give you lets say, a lunch box?

P: No.

I: Why not? Is that not important for you?

P: Its not important for me…

I: Mhmm… Why not?

P: I can also go to the shop and buy it for myself.

I: Mhmm… But you can also get this stationery pack and buy it for yourself as well.

P: But the stationery pack is more expensive than the lunchbox so…

I: Mhmm… Okay. Okay so and the about the…the contraceptives, can you just explain to me how contraceptives can be incentives?

P: After testing? Uh, they can give both girls and boys condoms because now we have female condoms and male condoms.

I: Mhmm… Okay. But do you think that is something that would, that, that would make someone want to come and get tested cause they can even get them here at the clinic for free without even testing?

P: No [laugh]… Uhh I think, uh, when they are given, uh, contraceptives…uh, uh the, where they get tested they’ll be given contraceptives but mostly they’ll be encouraged to abstain.

I: Mhmm… Okay. Okay any final thoughts before we come to the end of our discussion? Is there something that you wanna add or you wanna elaborate on?

P: Knowing your status is the best thing you can do alive because yours, you’re taking care of yourself. If you know whtas wrong, uh, you’ll start dealing with it. Mhm.

I: Mhm. Okay and I hope you’ll take your own advice and get tested [laughs]

P: [laugh]

I: Okay so we’re almost, uh we’ve come to the end of our discussion. With participant, with the participant. Uhm, thank you so much for being part of this interview, uhh thank you so much.

P: Okay. [laugh]

I: Uhm the time is fifteen, twenty-seven.

End time: 15:27
