# Supplementary material for: Exploring perceptions of low risk behaviour and drivers to test for HIV among South African youth
Source: PLoS One. 2021 Jan 22;16(1):e0245542. doi: 10.1371/journal.pone.0245542 (PMC7822253; doi:10.1371/journal.pone.0245542)
Supplement: S1 File — (ZIP) [file pone.0245542.s001.zip › S1_File_Anonymised Transcripts/YA01-012-TM Translation_QC2_TM.docx]

Full Participant ID**:** YA01-012-TM

Participant Type: Female

Location: Winnie Mandela Clinic

Date: 23 August 2018

Start time: 14:57

Primary interview language: English

Name of Facilitator/Interviewer: Bakang Mosimi

Name of Note Taker:

Name of Transcriber: Nokukhanya Ndinisa

Length of recording: 24:48

Label Key

I = Interviewer

P = Participant

N = Notetaker

{ } = Indicates that details were changed or pseudonyms were used to anonymise data

xxx = words were omitted to anonymise data

- = breaking into a sentence by the next speaker

… = pause or drawn out words

[ ] = indicates noise made, e.g. [laugh], [sigh], [pause]

[inaudible segment] = Unclear section of the recording

?Mulenga Clinic?, ?P3? = questionable text or doubt as to what was said or who said it

I: Uhm… you’re welcome. [noise in the background]

P: Thank you.

I: Do you allow me to record our conversation?

P: Yes, I do.

I: Okay… please describe to me, what do you understand about HIV?

P: Uhm, what I understand about HIV is that, eh… HIV is a virus that lives inside your blood. If I should say, uh… it can get treated, but not cured and uh…okay, if you don’t get treatment, it goes to like AIDS, where you get sick and stuff. [pause] And people don’t die because of HIV but because of uh, okay, HIV kills your soldiers, I don’t know, in your body and stuff. So, if you not physically active, eh… [pause] you can get other diseases easily and get more sick.

I: Okay. [sigh] How can one get infected with HIV?

P: Unprotected sex with someone who is HIV positive, using the same needle with someone who is HIV positive, Uhm, [pause] okay. When you have a cut and then you touch some’s blood who’s infected, uhm… by breast-feeding… okay when you born... you can be born with HIV and AIDS, HIV actually, not AIDS.

I: Okay [pause, background noise]. Tell me, what places a person at risk of getting HIV? In which places can people be at a higher risk of getting HIV?

P: Eh… I don’t know if I should say like poor communities as where people don’t have knowledge about HIV/AIDS, like they lack the knowledge. [Pause, cell phone beep] That’s where people can get HIV/AIDS.

I: Okay, poor communities-

P: Eh... Places with many taverns; places where parents are drunkards who don’t take care of their children’s health.

I: Okay. Why do you say taverns? What happens in taverns that exposes people to HIV?

P: Okay, people drink alcohol and when you’re drunk, they start fighting and end up cutting each other with bottles. It might happen that the person you’re fighting with actually has HIV, and when you touch their blood, you might also get infected.

I: Okay, thank you. Uhm, so can you tell me about any situation when you felt that you may have been at risk of HIV. Any situation whereby maybe-

P: Okay, it was when we were young and playing with my friends in the street. One of my friends mistakenly got stabbed by a bottle and we were supposed to help her, but we didn’t have gloves to wear when taking the bottle out.

I: Okay, can you tell me about the HIV testing services that take place in your community? [pause] uhm… eh… Which HIV services are offered in the area where you stay? Do you maybe have to go to the clinic or?

P: Clinic and then… some people stand in the street corners, I don’t know what they call them–

I: Mobile clinics?

P: Yah, you can test there.

I: Okay. Where are the HIV testing services for youth usually delivered? Where does the youth usually test? And which services do they use?

P: Mostly it’s clinics and the mobile clinics, that you’ve said.

I: Okay, can you tell me about your experiences accessing these services?

P: Can you elaborate?

I: Uhm…please explain to me about your experiences, if you have maybe wanted to access these HIV testing services. Explain what happened?

P: Okay, I once did but then I was scared that what if I have it. I don’t wanna know if I have it or if I don’t, like yes. That’s how I felt.

I: What were you exactly scared of?

P: That if I found that I have HIV, what would I do?

I: In your opinion, what is positive about the current HIV-testing services that are available to use?

P: The positive thing is that they help us to know our status, which is a good thing, we should know our status and they are sweet, so it’s easy for us to go and get tested where we know that we are free.

I: So, do you think that there is a need for these HIV youth-friendly testing services?

P: Yes.

I: Okay, in your opinion, what are the negative aspects about the current testing services that are available to use? Since you told me about the positive ones, what are the negative ones?

P: I don’t think there are. This thing is helpful.

I: Okay… How do you think incentives could be used to encourage youth to test for HIV and access treatment?

P: As a youth, eh... Obviously, if they say we must do something and then they offer us something else, so we would want to go there and do it so that I can get whatever that they are giving us.

I: Offer something like what exactly?

P: Okay. Maybe you know that when you get there, after testing, you gonna get food, Uhm... okay [pause]. Like if you get there to test and you find that you are HIV positive. Okay, it might happen that you will be depressed and stuff, then they can offer therapy so that you feel better.

I: So, what is your understanding [cough] by the term when we say something is an incentive? When we are talking about? Incentives, what is your understanding there?

P: Incentives are somethings that you get so that you can be more active. For example, workers get bonuses as incentives. So obviously, if you get bonus, you become more active when you do your job.

I: Okay. So, can you please describe the types of incentives that you value, which could encourage them maybe to test or access treatment?

P: Eh –

I: The types of incentives? Which incentives can we give the youth so that they can test or access treatment? You know sometimes, you would be scared to take treatment for certain reasons.

P: Okay…Give them t-shirts, caps… uh... watches, okay if you walk, transport back home. As girls, since we are youth, we love beauty. Sometimes you can give them like maybe nail polish and make-up so that they can do their faces and stuff.

I: Okay, is that all? Are those the things that you want in order to be encouraged to do all these?

P: Yes-

I: If so, how often do you think these incentives for HIV-testing services should be provided?

P: After three months, when you test after every three months.

I: Okay, after every three months?

P: Yes.

I: Okay... [pause] What could be the challenges of providing these incentives for HIV-testing services?

P: Uhm… People not coming to test, some will come because, okay… just to get whatever that you guys are giving.

I: Okay, and what would be the benefits of providing these incentives for HIV testing?

P: The benefits? More people in South Africa are going to know their status, which is a good thing.

I: Okay. Is that the only good thing about -

P: [Laugh]

I: … Providing incentives just to get HIV-tested?

P: We will be more active to get tested every time. Since if we come and get tested, we will know more about HIV and we’ll be able to protect ourselves from getting it.

I: Okay. So, on your previous question, you said you girls prefer lipstick and make-up, what about boys? What do you think would boys like?

P: Boys? Soccer… Like soccer balls, rugby, free tickets to watch matches at the stadium.

I: Okay… [pause] okay. Please tell me your thoughts about being contacted via telephone or social media for HIV-testing services.

P: If –

I: Please explain what your thoughts would be if I call you to inform you about the HIV testing services around you or if I inform you via social media

P: I think social media because that’s where we get most of the information and through social media, you reach more people.

I: So, do you think it will be more convenient for you go get information about HIV testing services via phone-call or social media?

P: Eish... Sometimes phone call for people because we don’t always have data and not many people are active on social media.

I: So, you prefer telephone calls?

P: Yes.

I: So, can you describe some examples of how you been informed about HIV testing services?

P: How I’ve been informed?

I: Mmmh…

P: At school, my L.O teacher. At home, and also at clinics, when we go, they encourage us.

I: So, at the clinic, what would you be doing at a clinic when you receive this information?

P: When I’m sick, maybe with flu or headache.

I: So, they give you health talks?

P: Yes.

I: Okay, how would you feel [cough] about being informed and registering for HIV testing using your cell phone?

P: It’s a good thing, yeah. I’d feel good. [laugh]

I: So, you’d feel good about registering and being informed about HIV testing services via your phone?

P: Yes.

I: Okay. How could cell phones be used to inform youth about HIV testing services?

P: We can call them using cell phones. Also, as you said, social media. Our youth is more active on social media, so it will be easy to reach them.

I: So, don’t you think that they would ignore those calls, maybe?

P: No, [laugh] they won’t.

I: Okay. Please describe any challenges that youth might [cough] experience if they’re being contacted on their cell phones for HIV testing services?

P: You won’t find them because some are busy studying –

I: Okay.

P: Then, okay, most time, phones are on silent and they don’t hear it when it rings.

I: So how do you think, we can address these challenges?

P: We can have certain times where we know that we should call these people from this time ‘til this time, that’s when we’ll be able to get them.

I: And what time do you think will be convenient for these young people to be reached?

P: I think from half past three, to six o’clock.

I: Please describe the benefit of contacting youth on their cell phones for HIV testing services. What are the benefits because we just talked about challenges? Benefits?

P: It will be easier for them to know more about HIV and like, people would be encouraged easily to test for HIV.

I: Okay. In your opinion, what types of other social media should be used to contact youth for HIV testing?

P: Facebook, WhatsApp, Twitter and Instagram…

I: So, if we’re going to use those kind of social media, what would be the challenges?

P: Not everyone has data and not every phone has internet.

I: Okay, I understand. So why preferably Facebook, WhatsApp and –

P: Eh… It’s easy to get information through Facebook and… and [pause] and then WhatsApp is something where you can get people every day. So, I think data should be like one of the incentives.

I: So, data would be more import- [pause] more relevant?

P: Yes.

I: So that you reach those social media networks to see the information about HIV?

P: Yes.

I: Okay. [clearing throat] So what are the challenges for using social media to contact youth for HIV testing services? Which challenges do you think will be there?

P: For youth? [pause] data.

I: Okay. So, data would be more efficient for youth to –

P: Yes.

I: So that they can reach these sites, right? Is that what you’re saying?

P: Yes, that’s what I’m saying.

I: I don’t want to put words on your mouth.

P: [laugh] Oh no! That’s what I’m saying.

I: Okay. What would be the benefits of using social media to contact youth for HIV testing services?

P: If we get data as incentives, so like, it’s gonna be easy for us, youth to go to internet, research… eh [pause] go to like Facebook, search your pages and we will learn more.

I: Okay, that’s a good thing. How do you think your parents or legal guardians will feel about you receiving information on HIV testing services on your cell phone or social media? What do you think your parents will say? Maybe seeing you on social media looking at HIV and what-not and getting a call?

P: Okay... I think it’s a two-sided thing. The good thing is that they might be happy that their child is concerned, like me as their child, they’d be happy that I’m concerned about my health and I wanna know more about what’s happening around me; and the other side is that they might get concern about the reasons why I’m searching HIV/AIDS if I’m doing nothing that will make me get HIV/AIDS… Yes!

I: Okay. Can you tell me about other suggestions that would make the youth get tested for HIV?

P: Uhm… we mentioned incentives, right?

I: Yes, what other ways can we use? [background noise]

P: Eh… Go to schools [pause] get time with different schools, tell people about HIV and also test them. Uhm… sports campaigns and entertainment.

I: So, what kind of entertainment maybe will encourage youth come and listen to what, maybe we came we came as {Aurum} saying to you maybe what kind of entertainment should we maybe do?

P: Dancing should be involved, singing –

I: Dancing competitions, is that what you’re saying?

P: Yes, something like that and singing, modelling, eh… [pause] like the talent show and stuff like that.

I: So, you spoke about sports campaign, what kind of sports? Which sports codes do you think will bring girls and boys together to? –

P: Soccer, both females and males can play soccer and then netball for women and also rugby for boys. Uhm… I think atlantics, right? Like the competition for running, where there will be a winner.

I: So, I heard you emphasized entertainment and talent show which falls under. So, [cough] if entertainment-, do you think entertainment will push youth to come and get HIV testing and information, maybe?

P: Yes, I mean as youth we like a place where, like the energy and atmosphere is good and like up there. So, everyone would wanna come.

I: So, what kind of entertainment? Like talent show, maybe?

P: Yes, anyone with any talent can come and join the talent show. Like, every child will have an opportunity to show their talents. Bring celebrities to become judges. Obviously, if there’s maybe like Cassper and most of us have never seen him, obviously, we will wanna come there just to see him and also others can expose talents and many opportunities can be available.

I: So, that means it won’t only be HIV testing services, then it will be about showing your talents and getting exposed to those celebrities?

P: Yes, and also opportunities. Like, okay, we have singers, celebrities and let’s say they were going to do a music video and you come to the talent show and show us your talent that you can dance, then there’s an opportunity that the person can come to you and offer you an opportunity to become part of the video.

I: So, it important for HIV testing to go hand-in-hand with entertainment?

P: Yes.

I: Okay. Uhm… Any other final thoughts maybe that you have about youth, HIV testing or incentives?

P: Uhm… You must do your job more, what you’re doing is very good so it should not be like Gauteng only, we should go around all provinces, this should be there, like everyone, especially in the rural areas where they lack knowledge about HIV/AIDS, you can teach them about HIV/AIDS, especially the youth there and offer them the same incentives that you’ll be offering us here.

I: Okay, is that all?

P: Yes.

I: Okay, now we’ve come to the end our discussion. Thank you for taking part in our discussion.

End time: 15:20
