# Supplementary material for: Exploring perceptions of low risk behaviour and drivers to test for HIV among South African youth
Source: PLoS One. 2021 Jan 22;16(1):e0245542. doi: 10.1371/journal.pone.0245542 (PMC7822253; doi:10.1371/journal.pone.0245542)
Supplement: S1 File — (ZIP) [file pone.0245542.s001.zip › S1_File_Anonymised Transcripts/YA01-013-LM_Translation_QC2_TM.docx]

Full Participant ID: YA01-013-LM

Participant Type: Female

Location: Winnie Mandela Clinic

Date: 23 August 2018

Start time: 16:52

Primary interview language: Sepedi (Transalated to English)

Name of Facilitator/Interviewer: Bakang Mosime

Name of Note Taker:

Name of Transcriber: Reba

Length of recording: 26:31

Label Key

I = Interviewer

P = Participant

N = Notetaker

{ } = Indicates that details were changed or pseudonyms were used to anonymise data

xxx = words were omitted to anonymise data

- = breaking into a sentence by the next speaker

… = pause or drawn out words

[ ] = indicates noise made, e.g. [laugh], [sigh], [pause]

[inaudible segment] = Unclear section of the recording

?Mulenga Clinic?, ?P3? = questionable text or doubt as to what was said or who said it

I: Do you allow me to record our conversation?

P: Yes, I do.

I: [sniff] Okay… Please describe to me your thoughts about HIV, what do you understand when we talking about HIV?

P: Uh, when we talking about HIV, we talking about, uh, the virus that you get from…from… Okay you get HIV when you are…when you being sexually active. Without protecting yourself…

I: Okay… And what else?

P: Uhm, [tongue snaps], use any language? Okay. {You can also get HIV when you touch another person’s blood when you are hurt for example and that person is infected with HIV; and then…they are hurt right?}

I: Mhmm…

P: {Yah, then your blood goes into your blood, you know?} That’s how you get infected.

I: Okay. How can you become infected with HIV?

P: [pause] Like I said, {you get HIV when there is someone who is hurt and then you touch their blood or} you’re being sexually active… {being sexually active and not using protection}.

I: Protection, what do you mean when you say protection?

P: {I mean condomising}

I: Okay… Tell me about what places a person at risk, is at risk of getting HIV? {Which places is a person} at a high risk of contracting HIV from? [door closing sound]

P: Uhm… {I would say…like are you saying places where a person can get HIV from?}

I: {Yes} That is that are more likely…maybe {when you’re there you could get HIV?}

P: {When you are at a club…}

I: Okay…

P: Cause {the people that are drunk} they end up having… {sleeping together} cause they are mentally, uhm, {their minds are not working properly}.

I: Ohoo…okay. So {is it only in clubs where you think} people is at high risk of getting HIV?

P: Yah, I think.

I: Okay… Okay, can you tell me about any situation when you felt like, that you may have been at risk of HIV?

P: Well {me, because I haven’t tested, like even now I’m afraid of being tested… I haven’t seen my status so I don’t know my status}.

I: So {maybe you…you haven’t felt that you are} at risk of getting HIV at some, at any point?

P: No, {during times when I was still a child, I used to, I like sharing sweets with my friend}, that’s when I got afraid thinking maybe {I could have HIV} and all that.

I: Okay…okay. [turns page] Can you tell me about the HIV testing services that take place in your community?

P: {There’s a, uh…there’s some people…I don’t know like, they set up tents and they test people for HIV.}

I: Mhmm… Okay. Is that the only place you know that are offering HIV testing services?

P: No, {even at the clinic}.

I: Okay. [cough] Where are the HIV testing services for youth usually take place?

P: {At the clinic and at school sometimes}

I: Okay. So do you have people coming to the school doing HIV testing services?

P: Yah {but you go if you want to}

I: Okay, but is it voluntary?

P: Yah.

I: Okay… Can you tell me about your experience accessing these services? {Which experience did you have, maybe you once tried to, to go and get tested?}

P: No, I haven’t tried.

I: You haven’t?

P: Yes.

I: So you don’t have any experience of HIV testing service?

P: No.

I: Okay. In your opinion, what is the positive about the current HIV testing services that are available to youth?

P: The pos-, the positive?

I: Yah. What, what do you think is positive about youth getting, uh…HIV testing…services?

P: {So that they can know the risks of HIV and they should also know their statuses…}

I: Mhmm… [page turns] Is that all?

P: Yah.

I: [page turns] [pause] Okay, and then what are the negative aspects of the HIV testing services that are available to youth, {isn’t it} you told me about, uh [turns page] {the} positive aspects?

P: Mhm.

I: What are the negative aspects? What, {what is it that you think is, is negative about} HIV testing services?

P: Well-

I: -For youth…

P: {When you go alone and then you find that you are HIV positive, you could faint or be emotional within people or…yah}

I: Okay… is that the reason why {you haven’t tested?}

P: [laughs] Yah…

I: Okay [high pitched voice]. Uh, how do you think incentives could be used to encourage youth to test for HIV… [clears throat] and access treatment?

P: Well, I think {that cause youth and people are always using phones and all, they can all, they can go test and they can give you airtime or data…or maybe food…after testing}.

I: Okay. So do you think those things {will encourage people to test?}

P: Yaaahhh.

I: Okay [sound of writing]. And what, whats your understanding when we’re talking about the term incentives? Whats your understanding, {what do you understand?}

P: Uh, incentives… {I think that when they talk about incentives, they talk about things that, that encourage} I think.

I: Okay... {Things that} encourage?

P: Yes.

I: Okay… [cough] So {you’re saying things that encourage are, are are...} what types of incentives that youth will value, if you were to give youth those incentives? What types of incentives that should be…to get them tested, to encourage them to test?

P: {They could give them airtime, as I said…data, food and t-shirts…uhm-}

I: {-T-shirts?}

P: Yah.

I: Okay, “dikipa” means t-shirts, {right?}

P: Yah, t-shirts.

I: Okay…

P: {And caps…mhm, yah.}

I: Okay. So as you mentioned t-shirts and caps, what do you think more t-shirts and more {those} caps, what should be written on them?

P: {They could write about} HIV, “know your status” …yah.

I: Okay. So any color, maybe?

P: Any color, black, white…any color it’s fine.

I: Okay. How often do you think these incentives for HIV testing services should be provided?

P: How often?

I: Mhmm…

P: {People that go and get tested for HIV right? Normally they are people, there are people…uh okay. Like when you go to schools, to go and test children, that’s when they should give them those things…}

I: Incentives?

P: Yah.

I: Oh okay. What could be the challenge of providing these incentives for HIV testing services?

P: The challenge?

I: Mhm.

P: {It is that even when people know their status, they will come just to get them}. To get the free caps and all that…

I: Mhm… Okay, those are the challenges?

P: Mhm…

I: Now…what would be the benefits of providing those incentives for HIV testing services?

P: The benefit?

I: Mhmm…

P: {So what will those people benefit, the people who are giving the things?}

I: What will be the benefits for providing, {how is it going to benefit them if we provide} incentives for getting tested for HIV?

P: Yoh. {What will benefit them?}

I: Mhmm…

P: Well {a lot of people will come and get tested when they see the things…}

I: So {a lot of people will be encouraged by incentives}?

P: Mhm…

I: Okay. Please describe to me your thoughts about being contacted via telephone or social media for HIV testing services? What’s your thought about that? Maybe uh uh {if we could call you or write to you on social media about uh, HIV testing services?} Do you think that would be cool?

P: Yah that would be cool cause you don’t have to see people {that you’re talking to, even if you get emotional}, they don’t see you…

I: But remember this is just to inform you about the HIV testing services, do you think you will, if we send maybe a promotional message saying “theres an HIV testing services taking place where and where…”, do you think that would be a good idea to do it if we call you or you see it on social media?

P: No, cause social media like, is just to have fun so {people won’t take it seriously}

I: What about you?

P: {I wouldn’t take it serious…}

I: Even if you we call you?

P: Yah, like {I’m lazy, I’m lazy} [laughs] {to leave where I am and go elsewhere just} because I got a phone call, I wouldn’t go.

I: [laughs] Oh okay. Can you please describe some examples of how you have been informed about HIV testing services?

P: Uhm, {at school, people used to come out to test us for HIV, from the clinic and test us…}

I: Mhmm…

P: [snaps tongue] {they only test those that want to, yah, uhm… The pamphlets from the clinic when you go, sometimes they give out pamphlets about HIV, yah.}

I: So those pamphlets, {do you read them or do you just take them?}

P: {We read them because you will be at the hospital, like at the clinic, also they are looking at you and watching you…}

I: Yeah…

P: {So you’re gonna read them…}

I: So you just read them for, for the fact that you think you might be…?

P: You might be cut; {they are looking at you right?}…Yah.

I: Okay. So {have they ever motivated or encouraged you to get tested} at some point?

P: No. Like I said, {I’m afraid}.

I: Okay [laughs]. Okay. How would you feel about being informed and registering for HIV testing services using your cellphone?

P: Registered?

I: How would you feel maybe {if I informed you} about HIV testing services or {if I registered you} so that you can come and get tested for HIV via cellphone?

P: Nah, it wont be, it won’t, it won’t, it wouldn’t be cool because {things coming from my phone}, I don’t take them seriously.

I: You don’t take them serious?

P: Mhm.

I: Why is that?

P: {I don’t know} like, a phone is just… {It’s just} something that I have fun {with}, {the things that are on my phone, I don’t take them serious.}

I: So don’t you think reading a, a message…a HIV testing message won’t be fun?

P: Uhmm… {I, I don’t know} [laughs].

I: [laughs]

P: Like, yah I would do it but then, it would be fun but {I wouldn’t pay more attention}

I: So which fun, maybe where we can, which fun do you think we can use to make it more interesting so that we can really learn from it or maybe you can take that call…? What do you think we can do to improve…?

P: Yah I think, maybe {if it’s a call and not a message, that will be better…}

I: Okay…

P: {Cause you’re definitely gonna answer the phone, right?}

I: Okay…

P: Yah, so… {and then you’re gonna listen, you won’t just hang up the phone}, that would be rude.

I: Oh okay. That’s great. How could cellphones be used to inform youth of HIV testing services?

P: Cellphones? Well they could call them {and} inform them…about HIV and {going to get} tested. [pause] Texting…and also texting, yah.

I: Okay. Please describe any challenges that the youth might experience if they are contacted on their cellphones for the HIV testing services? {What challenges are there that you think…the youth may experience} when they get contacted about HIV testing services?

P: Challenge?

I: Mhm…

P: {That they could face?} Uhm, lemme see. [pause]

I: Any challenge.

P: {They could not trust those people because cellphones these days, there are people that prank you right?}

I: Mhmm…

P: {So you could think that maybe they are trying to steal/kidnap me or something like that…so they could not trust these people}

I: Mhmm okay. Please describe the benefits of contacting youth on their cellphones for HIV testing services, {isn’t it} you’ve already told me about the challenges?

P: Yah.

I: So what are the benefits? What will be the benefits?

P: Of consulting them?

I: Of contacting them on their cellphones to…letting them, letting them now about HIV testing services?

P: The benefit? Well, it will benefit them in a way cause, uhm… {it will save them time to maybe go to the clinic, yah all your energy, plus its hot, I would be lazy}

I: Oho…so you wouldn’t go to the clinic just to ask about HIV?

P: No, more especially when its hot. No.

I: [laughs] So this, uh, this way of, of texting or calling you will be more convenient for you?

P: Yah it will.

I: Okay. In your opinion what types of social media should be used to contact youth for HIV testing services?

P: Uhh, we can use Whatsapp.

I: Okay.

P: Uhm, [pause] we can actually, again we can invite them to like the page {of} HIV {on} Facebook.

I: There’s Facebook. Mhmm…what else social media?

P: Uhm, Twitter.

I: Okay. How do you think youth will access all these social media networks?

P: Yoh. Youth {is always busy on their phones} so obviously they will just see uh, {them} on Whatsapp 24/7, {they are always on} Whatsapp, Twitter, Facebook; they’re always online so…yah.

I: Okay. So what could, what do you think could be the challenges?

P: {The} challenges of?

I: {The} challenges of using social media to contact youth for HIV testing services?

P: Mhmm, the challenges?

I: Mhmm… [page turns]

P: [pause] Uh, {you could run out of} data, airtime…yah.

I: Okay. So you’ll need airtime and data to access this...?

P: Mhmm…

I: So what if they were, they were free? Do you think youth will uh, open Facebook page to access HIV testing services? Just to read if it was free?

P: Uhm, if it was free to open?

I: Mhm.

P: Eish, youth ok? [laughs]

I: [laughs]

P: Yah, some of them, not…maybe 15 uh no. 30% would.

I: Okay. So in that sense, you saying data is more important as an incentive so that youth can reach these social media networks?

P: Yah.

I: Okay. Its good. And besides the challenges then, what are the benefits of using social media to contact youth for HIV testing services?

P: The benefits?

I: Mhm.

P: In other words, you’re trying to say what will the, what will…-

I: -What will, what will benefit the youth if we post those kinds, uh, that information about testing, about HIV testing services…especially for youth maybe?

P: Uhh. It will help them to [pause] [tongue snaps], to know more about HIV and…also to tell others.

I: Mhmm…

P: Yah. And again it will encourage them to come and test.

I: Ohh. How do you think your parents or your legal guardian would feel about you receiving information about HIV testing on your cellphone or social media?

P: I think they would be happy cause some of the parents are not free {to talk to their children about things such as HIV} and all that.

I: Mhmm…

P: Yah. They will feel that social media is good {because it will do the work for them} [laughs]

I: Okay. That’s a good one. So they won’t have a problem, maybe they-

P: {-They could even buy you data}, like…yah.

I: To be on that page more often?

P: Mhm.

I: That’s great to hear. So [sigh] how do you think uh, the… {How will it motivate you that thing}, maybe your parents buying you data to be on the page; {how will it motivate you to end up testing yourself?}

P: {If you have data, you will go and read right? And to see that actually testing for HIV} is not such a bad thing.

I: Mhmm…

P: {Then you’re gonna end up wanting to know your status}.

I: Mhmm…okay so you need, all you need to get tested is encouragement?

P: Yah.

I: And maybe a little bit of incentive?

P: Yah. [laughs] Yah.

I: Lets probe more into incentives, do you think these incentives can, it’s the only thing…are the only things that will make you to get tested? Or do you think, do you have any more uh, opinions about incentives, you want to add maybe to the ones that you just said?

P: Uh we, we can actually give them anything. I don’t know, like…

I: Maybe let’s specify those “anythings”?

P: Those anythings…

I: Yah.

P: Yah. Also stationery.

I: Stationery… Mhmm…

P: Mhm. Some could, okay…actually {at school you could give them uniforms}

I: Okay.

P: And also…-

I: -Uniforms? Yes.

P: Yes, uniforms, the vouchers…

I: Okay, what kind of vouchers?

P: Mhmm, the stipend; does that make…uh, yah.

I: Stipend?

P: Yah, stipend.

I: Can you explain stipend? What kind of stipend?

P: Like…mhmm… {You can bribe them a little with money, you see?} [laughs] Not really bribing {but} yah…

I: Okay so do you think money could be an incentive?

P: Yah. Everyone will…-

I:-Its not bribing right? Its incentive.

P: Yah. [laughs] Its incentive.

I: Okay [turns page] uhh, can you tell me about, any other suggestion that you may have which could encourage youth to get tested? Any other thoughts, suggestions…?

P: Mhm… {There could be containers where they play music and people would be dancing but then it would also be a testing of HIV}.

I: So entertainment could help?

P: Yah entertainment.

I: Okay.

P: Sport campaigns, yah {even} sport campaigns, talent shows…yah.

I: So do you think youth will be interested in those sports campaigns or talent shows?

P: Yah, more especially {when they have celebrities}

I: Okay.

P: Yah, they could get interested.

I: Why specifically celebrities? How about we bring a doctor or nurse, what do you think-?

P: No {those people aren’t popular, right?} so {when there are celebrities is popular, popular…people know them}

I: Mhmm…so these popular people will make you, will convince you in a way to get tested?

P: Yah.

I: Okay. So do you want them to test as well…at that point, maybe {you guys can come after?} So that you can see how –important it is?

P: -Yah. {If a celebrity can do it, why can’t I do it?}

I: Okay… Which is good. Any other thoughts?

P: Any other thoughts? No, I think that’s all.

I: Maybe on incentive or how we can improve HIV testing services?

P: How can you improve them?

I: Mhm.

P: [snaps tongue] Uh, {you can play a talent show} …uh yah. Yah.

I: Do you think all these, uh HIV testing services are accessible to you, are they youth friendly?

P: No, not all of them. Yah, not all of them.

I: So, what do you want to see? What do, what would you like to see maybe uh, a youth friendly service, HIV testing services should look like? What do, what comes to your mind?

P: Uhm… {you need to have [snaps tongue] music, yoh youth and music…} number one.

I: Okay.

P: [laughs] {when theres music, its what attracts them}

I: Oh so you think music will be, music will make it, the HIV testing services more friendlier?

P: Mhm.

I: Okay.

P: {And food}.

I: Food?

P: Yah.

I: Okay [laughs]. Entertainment goes with food.

P: Yah.

I: That’s what you are saying…

P: Yah [laughs], that’s what I’m saying.

I: Okay. Great. Is that all or maybe you have final thoughts or suggestion about HIV testing services and youth incentives?

P: Mhm. No, uh, no yah. They should be in a public place {where people pass by a lot so that they can see}

I: Oh yah. Yah,

P: More especially {uh, what can I say? Have you seen where they sell bunny chows, how full of people it is?}

I: Yah.

P: Yeah {so it could be there or close to where they sell bunny chows.}

I: Oh, it should be more like on public places like malls…type shops? Okay

P: Mhm.

I: Okay. Any other thoughts?

P: Nah, I’m out of thoughts.

I: [laughs] Okay. Well thank you for taking you time to, to have a conversation with us ok?

P: Yah.

I: End time is 16:21.
