# Supplementary material for: Exploring perceptions of low risk behaviour and drivers to test for HIV among South African youth
Source: PLoS One. 2021 Jan 22;16(1):e0245542. doi: 10.1371/journal.pone.0245542 (PMC7822253; doi:10.1371/journal.pone.0245542)
Supplement: S1 File — (ZIP) [file pone.0245542.s001.zip › S1_File_Anonymised Transcripts/YA01-014-KM Translation_QC2_TM.docx]

Full Participant ID: YA01-014-KM

Participant Type: In-depth Interview

Location: Winnie Mandela

Date: 23 August 2018

Start time:

Primary interview language: IsiZulu (English, Setwana)

Name of Facilitator/Interviewer: Wellington Maruma

Name of Note Taker:

Name of Transcriber: Ornate Masuku

Length of recording: 27:24

Label Key

I = Interviewer

P = Participant

N = Notetaker

{ } = Indicates that details were changed or pseudonyms were used to anonymise data

xxx = words were omitted to anonymise data

- = breaking into a sentence by the next speaker

… = pause or drawn out words

[ ] = indicates noise made, e.g. [laugh], [sigh], [pause]

[inaudible segment] = Unclear section of the recording

?Mulenga Clinic?, ?P3? = questionable text or doubt as to what was said or who said it

I: In-Depth interview the date is the 23^rd^ of August 2018, participant ID is A01-014-KM. The participant is female aged 15 at {XXX} (Name of place where interview took place)and the interviewer is {XXX} (interviewer name).Uumh thank you so much for being part of this interview for agreeing to be part of this interview. Do you allow me to record this interview?

P: Yes

I: Ok thank you, can you tell me what HIV is? What comes to mind when you think of HIV?

P: Ok HIV is a disease; I cannot say that it’s a disease that kills but it’s a disease that will be in your blood. You can treat it so that it won’t kill you

I: Uumh and then what else can you tell me about HIV?

P: Uumh

I: Like how can it be transmitted?

P: You can get it through the blood of someone, yea

I: Uumh

P: Or a boy can sleep with a girl, yea.

I: Any other way, which you know?

P: I don’t know maybe you can be born with it depending if your parent has it or not

I: Uumh

P: Yea

I: Ok and then how does one become at risk of acquiring HIV?

P: How they can acquire HIV?

I: Uuumh

P: Yho! Maybe

I: You said sleeping around puts you at risk of acquiring HIV, what else?

P: No I don’t know

I: You don’t know?

P: Uumh

I: Personally have you ever been in a situation where you felt you were at risk of HIV, of getting HIV?

P: Uumh uumh no never

I: Never?

P: Uumh ummh

I: Ok you only know that HIV is transmitted through sex only?

P: Eehm, yea through sex and touching blood of an HIV infected person but I don’t think my parents have it

I: Ok have you personally touched someone’s blood?

P: No I am scared of blood, I wouldn’t touch it

I: Ok so you said, have you ever tested for HIV before?

P: No

I: Why not?

P: Eish I don’t like needles you see, and I don’t even think I could be HIV positive I don’t see myself in that position

I: Uumh

P: I don’t remember my mother going to the clinic to get treatment or my dad I don’t remember so I doubt there is a way that I could be HIV positive

I: Uumh ok and you don’t think you said you are not the type to get HIV right, is that what you are saying maybe I misunderstood you?

P: It’s possible but I don’t think so

I: How come?

P: For now, I don’t think so

I: Why?

P: Isn’t for now I am not misbehaving if I was misbehaving maybe I would be vulnerable since you can get it in different ways

I: Uumh

P: Uumh

I: Ok and then what do you know about HIV testing services here or at the clinic or in Tembisa or maybe have you heard about testing services of HIV?

P: Uumh uumh I have never

I: You have never?

P: Yea never

I: Ok, ok at school no one has ever come to test for HIV?

P: Eh! I don’t remember

I: Ok what about at the clinic, you don’t know that they test there?

P: Yea I know

I: Ok and where else?

P: No, I only know that people are tested at the clinic?

I: Ok, have you ever heard of other places where you get access to HIV testing services or it’s only at the clinic?

P: Yea I only know of the clinic

I: Ok, what do you think is good about testing here at the clinic? What’s the good thing about testing here at the clinic?

P: It is nice to know your status and your standard and you know where you stand, you also get to prepare for what’s ahead? So if you don’t know there might be a danger ahead

I: Ok, why do you think it’s important for one to know their status?

P: Because you don’t know what lies ahead, so you need to know hence the need to go test that’s what I think

I: Ok why haven’t you tested then?

P: [laughs] Yho!

I: Because you say it’s important but you haven’t tested

P: Eish! Aah! Eh those nurses will be shouting at me, you know how things are at the clinic. If they won’t shout I might consider going but then again there is also the injection

I: Do you think the nurses at the clinic are annoying when people come for testing? Have you maybe heard of that situation happening?

P: No I have never heard but

I: Why do you say so then?

P: You will never know, how the mood of the person you find will be, you might find one unhappy or they are just having a bad day so they might just end up angry, yea

I: Ok and then when you hear incentives what comes to mind?

P: Uumh let’s say you want to talk to the youth about maybe HIV and you offer them something if they come and test for HIV

I: Uumh

P: T-shirts, caps and bottles things like that

I: Ok so u mentioned t-shirts, bottles and?

P: Caps

I: Ok, what else? What do you think can make the youth come test for HIV?

P: Maybe it can be bags, yea. Uumh what else, ok yea that’s all I can think of

I: Ok

P: And maybe food, people of {XXX} (Name of place)love food

I: Ok food

P: Yea I think that’s it

I: What else?

P: Eish I don’t know, yea those things that I have mentioned [laughs]

I: What?

P: Bottles

I: Ok what else?

P: Lunch tin

I: Lunch tin?

P: Yea

I: Ok

P: Uumh I don’t know

I: Uumh

P: Those small bags that are used to store lunch tins one can take to school or office

I: Ok so you mentioned bottles, t-shirt right?

P: Yea t-shirt

I: Caps bags, uumh food

P: Uumh

I: Bottles, lunchbox. Out of everything which one would make you come and test? Like let’s say I give you an option for you to only choose one

P: I would choose a t-shirt

I: T-shirt?

P: Yea, sometimes its necessary people know where you are coming from

I: Uumh

P: They ask you where did you get this thing when you are wearing it someone will ask where you got it from, what did you learn from there? What do they teach there? If you are wearing it clever people will see that you did a good thing and they could also get encouraged to do the same thing you did

I: Uumh ok, this t-shirt

P: Uumh

I: What will be written on it? Like what will it be like? Using your imagination what would look like?

P: There is no need for it to be nice

I: Ok

P: It just needs a name

I: What name would that be?

P: Any name that will help the youth in terms of HIV, something like “Go test”. With that name clever people will be happy to go test so that they also receive the incentives like t-shirts and they also want to be asked these questions.

I: Ok and then if I remove the t-shirt what would be the next thing you would choose

P: Uumh eish a cap

I: Caps?

P: Yea but those can only be given in summer they are not needed in winter, yea

I: Ok

P: Yea in summer caps are ok. Also bags, school kids would appreciate those and others will even ask where you got it from and what do people do there, yea?

I: Uumh

P: If they ask then you explain to them, if they are also interested they can also go check and by doing so they will be involved in the program

I: Ok, like by providing these things to the youth like the 15-24 years or whatever, what do you think are the challenges with this?

P: Challenges?

I: Uumh

P: What kind of challenges?

I: I don’t know you tell me [giggles]

P: Uumh eish

I: Do you think people will come only for these things not to get tested?

P: Yea that’s possible they could just come to get t-shirts and the other things but it’s needed that a person not only comes for those things but for the knowledge they will get

I: Ok, so what can we do to encourage the youth to come even if we won’t give them these things? And they just voluntarily come

P: Without these things isn’t?

I: Uumh

P: By the way you express yourself to the people and by the way you speak and explain to them everything.

I: Ok and then how often do you think we must give them these things?

P: It is not necessary that you give them many times. Just once is enough

I: Ok

P: This is because the person will know that they don’t need to repeatedly get these things as others also need to get. The others need to get also so that they also spread the word to others that ask

I: Uumh, but don’t you think the problem with giving them once a person will come to test for HIV only once and next time what will motivate them to come again

P: It’s the statement that you said and the way that you explained they can be able to face problems they might encounter

I: Uumh, so you think if we give them once is enough?

P: Yea

I: Ok and then uumh and then you spoke of the challenges of giving people these things

P: Uumh

I: So that they come for HIV testing or for accessing HIV treatment, and the benefits?

P: It’s that you can know where you stand

I: Uumh uumh

P: What level you are at, you know that if you wish for a certain thing you can go and get help of this type at this certain place

I: Uumh ok and any other thing that you can think of?

P: Aah no that’s it

I: That’s all?

P: Yea

I: Ok so you have never tested before

P: Yea

I: So uumh information about HIV and testing how would you want to get it?

P: I personally I don’t need to be given anything so that I go and get tested. You would just need to talk to me and explain everything that’s happening I will be ok

I: Uumh

P: Uumh

I: Ok and then tell me what you think about this

P: Uumh

I: So say people maybe from Aurum, from Department of Health or whatever they contact you via the phone or through social media or any other thing that they tell you about HIV testing services how would you feel?

P: They just tell me?

I: Yea they tell you maybe that test, go get tested how would you feel? What are your thoughts on that?

P: I would prefer that it would just be me and the other person just the two of us

I: Face to face

P: Face to face I won’t like something that I receive on my cell phone. There are problems with receiving news or information from social media

I: What kind of problems please explain?

P: Uh I don’t know where to start or say, but you cannot trust what people say through the phones

I: Uumh

P: So it’s important that when you speak to someone you speak face to face and they explain everything to you

I: Ok

P: Uumh

I: So you prefer to get this information face to face

P: Yea

I: Given by who?

P: Mmh?

I: Ok so who’s telling you this information?

P: Anyone it doesn’t matter who that person

I: Even though it’s your friend?

P: Ha aah! I don’t want a friend

I: Cause you said anyone

P: Anyone expect a friend since they know nothing, I can maybe take their advice but not everything because they don’t have all the knowledge. So I think it would be better if there was an adult with knowledge of this

I: Who do you want maybe your mother?

P: No, not my mother she doesn’t know anything

I: Uumh cause

P: I want someone with knowledge of this thing, like they went to school and studied for what they are talking about

I: Ok so like you said you prefer face to face than social media right

P: Yea

I: Ok and then what do you think, so you would also not feel good about being informed via cell phone via sms not even social media

P: If I have met the person before it will be ok, but if I have never spoken to them and I don’t even know who they are I will not give them the time of day

I: Ok let’s say the sms is from Aurum, let’s say we take phone numbers of everyone at school and

P: Uumh

I: Then we send maybe information about go get tested this is important to know your status. Then you get this sms on your phone

P: You would have said your name when we met for the first time so if I know your name and you are the one that explained about the certain thing so I won’t have a problem because I would know who you are

I: And then if you don’t know who it from?

P: I will ask first

I: No let’s say for example it’s from the department of health an HIV testing something information

P: Eish I don’t thin…

I: You don’t know these people but you trust these ones because they are from department of health right

P: What if they just wrote Department of Health? You cannot trust people they can just randomly write this

I: Ok so cell phones how do you think we can use cell phones to get information to the youth?

P: They will get what?

I: Maybe for someone who does like face to face. Isn’t you prefer face to face?

P: Uumh

I: And someone else wants to get information through facebook, social media sms or what not

P: Uumh

I: How do you think we can use cell phones?

P: We can use them to connect with them, most kids love cell phones it would be a better way but for me that isn’t the right way

I: Ok is that the only reason why you don’t want social media cause people might post wrong things or inaccurate information

P: Yea that is the reason

I: Uumh ok

P: Yea

I: But then someone can talk to you face and speak incorrect things

P: If they speak incorrect things I can tell them that that is false information

I: How would you know since we are teaching you these things?

P: Aah aah no! Even at school they talk about such topics, and if information is incorrect you can feel that this is wrong

I: Ok so say, let’s say you didn’t have this problem with social media right, what social media do you think we could use to reach the youth?

P: Which ones?

I: Uumh

P: Yho! Which ones?

I: Uumh

P: … Social media that connects people?

I: Uumh

P: Cell phones facebook

I: Uumh

P: Uumh facebook, instagram, twitter uumh yea

I: Ok and how do you think we could use facebook to give youth information about HIV testing services? Like give me an example how we would use it to distribute information

P: You need people that you have helped before so they can show those who don’t know what really the program is about

I: Ummh

P: So you need people that you can show and then you can add whatever else you want

I: Ok you think facebook will be effective in reaching people?

P: Yea a lot of people love facebook hey

I: Uumh

P: Yea they would appreciate it

I: Ok the other social media platforms instagram and twitter how we can use those to connect with the youth

P: I don’t use those I only use facebook

I: Ok so you said the challenges of using facebook and social media people will post incorrect information

P: Yea

I: And then what are the other challenges you can think of?

P: …Maybe that other wont appreciate this program

I: Uumh

P: They won’t like that you post about HIV and say go test so some might be offended by the post

I: Ok thank you. And then so let’s say we spoke to your mother

P: Yea

I: Do you think she will be happy that you are getting messages Department of health or whatever?

P: Yea

I: On your phone about HIV testing services, it’s good to know your status, it’s important to adhere to treatment do you think she will be happy seeing those types of messages?

P: Uumh uumh she won’t be happy, she prefers that you sit her down and explain everything about your program

I: Uumh

P: So she won’t be happy cause if she sees something on the phone she will automatically assume you are doing naughty things. So she will appreciate being sat down and be told about all these things

I: Ok so why do you think your mom won’t be happy that you are getting these messages

P: She doesn’t trust people she prefers someone to come face her, so she won’t trust that social media cause she knows no one there they can randomly write Department of health. So she wants you to sit her down and say I want to do this to your daughter

I: Ok so what do you think we can do to encourage the youth your age to come to the clinic or maybe test, any suggestion

P: Like you said

I: Uumh

P: T-shirts, caps, bags yea

I: What else?

P: Talking to them the youth prefer that someone talks to them rather than just giving them something. They prefer that you talk to them and explain things, for example in my class some people were not just happy that Aurum is coming because of the food. of course some just wanted the food but some others just wanted to acquire knowledge

I: Uumh ok so you think talking to them will be more important

P: Yea

I: So maybe at school right?

P: Uumh

I: Ok but what about those that are not at school? Not everyone goes to school

P: Yea

I: So how can we reach those ones?

P: You can reach them by talking to them, you can also speak to them, when you see someone idle you can approach them and tell them about this thing. School kids are talkative when they get home they will tell their mother that these people came to school and told us about this and this. If the mom is a gossip they can tell their neighbour and that neighbour tells someone else and the cycle of information can keep extending

I: Uumh ok talking, and you don’t think maybe those we cannot reach we can maybe reach them through the phone maybe

P: Yea you can also reach them that way

I: Through sms or those social media platforms you mentioned

P: Yea through social media

I: So social media would be better than talking face to face? Which one will be better for you because you said you prefer face to face but now you are saying we can reach more people through social media?

P: I don’t mean for me but for others social media would be more preferable. I personally prefer that someone talks to me face to face but others because they like things they will be ok with being reached via social media

I: Uumh ok. And then let’s say you agree to get these messages on your phone

P: Uumh

I: What kind of messages would you want to get?

P: …That HIV message will also be ok but it will have to be from someone I personally know only. If you are to send me that will be ok cause I know you, but if someone I don’t know sends it I would just ignore it

I: Uumh ok. So let’s go back to the incentives you mentioned bottles, t-shirts, caps and all that

P: Uumh

I: So uumh can you explain to me with regards to food, what kind of food will be served?

P: … Healthy food that is nutritious for the body, not junk like chips but food that is good for the body. If maybe we give them an example and say this is the nutritious body building food, I think that’s ok

I: Ok give me and=example

P: Sho! Example ….uumh aai

I: Let’s say I came now and say after testing I will give you this kind of food would it be pap and thing? Is it just pap or any other thing?

P: There’s a need that there are fruits and vegetables also

I: Uumh

P: Even though some people might not like it but it’s the type of food the body

I: Ok and then besides talking to the youth about testing for HIV, what else can be done to encourage them to test for HIV? Do you think there is there any other thing?

P: Uumh uumh

I: Ok

P: I am not sure about that

I: And you don’t think I there are challenges with that

P: With what?

I: With just talking to the youth. Some their parents talk to them but still the child refuses to go and test

P: No you can scare them a bit and say if you don’t test these will be the consequences and you will see the person will go test.

I: Ok thank you we are almost at the end of our interview

P: Ok

I: So uumh we are almost done with our interview, thank you so much n. The time is 4: 29 4:30 actually. Thank you so much

P: Sure

End time: 4: 30
