# Supplementary material for: Exploring perceptions of low risk behaviour and drivers to test for HIV among South African youth
Source: PLoS One. 2021 Jan 22;16(1):e0245542. doi: 10.1371/journal.pone.0245542 (PMC7822253; doi:10.1371/journal.pone.0245542)
Supplement: S1 File — (ZIP) [file pone.0245542.s001.zip › S1_File_Anonymised Transcripts/YA01-015-CS Translation_QC2_TM.docx]

Full Participant ID: YA01-015-CS

Participant Type: In-depth interview

Location: Winnie Mandela

Date: 23 August 2018

Start time:

Primary interview language: IsiZulu (English)

Name of Facilitator/Interviewer: Wellington Maruma

Name of Note Taker:

Name of Transcriber: Ornate Masuku

Length of recording: 35:00

Label Key

I = Interviewer

P = Participant

N = Notetaker

{ } = Indicates that details were changed or pseudonyms were used to anonymise data

xxx = words were omitted to anonymise data

- = breaking into a sentence by the next speaker

… = pause or drawn out words

[ ] = indicates noise made, e.g. [laugh], [sigh], [pause]

[inaudible segment] = Unclear section of the recording

?Mulenga Clinic?, ?P3? = questionable text or doubt as to what was said or who said it

I: In-depth interview, the date is uumh the 23^rd^ of August 2018. The participant is female aged 15 with participant identifier number A01-015-CS, and the interviewer is {XXX} (interviewer name). Ummh thank you so much for agreeing to be part of this interview, do you allow me to record this?

P: Yes

I: Ok, so can you tell me what do you think of, what do you think HIV is?

P: Ummh I think that HIV is a disease that is not good cause if you have sex with your boyfriend without a condom there is a chance that you might get HIV. So you must go to the clinic they will give you information then also give you ARV’s so that you can live life healthy.

I: Uumh ok so you mentioned that you get infected let’s say if you have sex without a condom

P: Ehem

I: Are there other ways you know of? Just relax its fine

P: [Chuckles] Ok, if you have sex without a condom, you can either get HIV or STI’s, things like that.

I: Uumh

P: So there will be a need of a doctor so that they can help you with things like that

I: So are there any other ways you know of?

P: It is only this way, I don’t know any other way.

I: ok and have you ever been in a situation where you felt you were at risk of HIV?

P: Ummh eeh eeh

I: Never?

P: Yea

I: Serious?

P: Serious

I: Ok, you look like you want to say something

P: Eeh eeh

I: So what testing services do you know of around the community?

P: [Sighs heavily] Oh my God! I don’t know shame

I: Uumh

P: Cause I’m no yea...

I: Ok but you know the clinic offers testing services?

P: Yea

I: And where else?

P: Here and there if I am not wrong

I: Ok, no I mean like a place where they test for HIV

P: Oh! Its either they go from house to house or you come to the clinic

I: Uumh, and have you been exposed to the clinic or people came to your house?

P: Uumh here not at the clinic

I: At home?

P: Yea

I: So you have seen them before?

P: Yes

I: Ok so they came to your house?

P: They were going around testing people that didn’t mind, but I didn’t test

I: Why didn’t you test?

P: My problem is I am scared of needles and I don’t like injections. Plus my blood eish

I: What about your blood?

P: It’s not a lot its little cause I don’t drink water or eat a lot of things so yea

I: Ummh, ok so you are scared of your blood being drawn?

P: Yea

I: Ok and any other reason why you didn’t get tested?

P: [giggles] The thing is I have never been intimate with a boy; yea I have never had sex

I: Ummh

P: And I don’t share a spoon with anyone

I: Uumh ok so you think HIV can be transmitted through sharing spoons?

P: Yea they have said something like that

I: Who?

P: My grandmother the one I stay with

I: Ummh

P: Yea

I: Ok what else like how else can you get HIV?

P: Uuumh

I: You said through sex without condom and what else?

P: Uumh I think through lips

I: Ok

P: Yea I think so but I am not sure

I: Don’t they teach you at Stepping Stones?

P: No… yea serious

I: Ok so we spoke of people who came to your house to test you guy’s right

P: Yea

I: To test for HIV, so can you tell me your experience when you came to the house. What happened? What did they do? Experience the whole thing

P: Ok, they go there and greeted, they then said that my sister then agreed to be tested. They said that if you go and test at the clinic you must not allow them to prickle you with a testing kit that is already open but they must open it in front of you then go ahead and prickle you.

I: ok

P: Then they said after two she must come and collect her results to find out if she is negative or those types of things

I: Ummh

P: Yea

I: Ok so what are the good things about these people coming to your house to test for HIV? It’s a good thing or its not?

P: Eish! It’s ok that they test at the house, so that if I am positive only my grandmother will know cause here eish they are always talking

I: Uumh

P: Yea they talk too much

I: Ok, so you think testing at home is better than coming here to the clinic?

P: Yea cause here at the clinic they talk too much and say bad things you see

I: Ummh

P: So it’s fine if they come to the house

I: Ok and that is also the reason why you don’t go to the clinic?

P: Yes

I: Cause they talk too much

P: Yea and they are very judgemental, they pretend as if they have never been sick or had a disease yea so

I: Ummh uumh, so what do you think we can do to encourage the youth to come and test

P: …I think there should be a room that deals with just, HIV and one does not need to pass by other rooms

I: Ummh

P: Cause people are very judgemental. There must be a room specifically designed for HIV, so that when you come to test for HIV you go straight there. Yea maybe be better than them doing house visits

I: Ok, so you mentioned coming to the clinic nurses may be talking too much and they might disclose people’s statuses maybe.

P: Yea

I: So what else do you think is negative about testing at the clinic?

P: Ummh something negative about testing at the clinic?

I: Ummh

P: It’s that if they test and find that you are positive they will judge you before they tell you the results. So at least at home if they tell you that you are positive, your mom will be there and accept your status but at the clinic they can go on and on before they share the results

I: Ummh

P: Yea

I: Ok and then the, so personally where to you prefer to test?

P: I prefer home rather than the clinic

I: So if people come to your house to test would you want them to come again to test you?

P: Aah eh

I: Where else would you test?

P: Ummh its ok if they test me at home

I: Ummh

P: Yea but I won’t test again; if I find out that I am negative I will not re-test, yea

I: Ummh, ok so what would make you test every three months or six months

P: Ummh there’s nothing

I: Ok, so when we speak of incentives what comes to mind? Maybe explain to me what incentives are according to you?

P: According to me?

I: Ummh

P: Eish, according to me you can give them something, so that thing can encourage them to come to the clinic and the thing can pressurize them to come test.

I: Ummh so what is that something?

P: Uumh I think cap or t-shirts or yea

I: Cap and t-shirt only?

P: Uumh and a bottle only

I: Ok and why do you think these things would be important for you, we are talking about you personally

P: Yea

I: So let’s say they come to your house and say a cap, t-shirt or a bottle. Why do you think these things are important for you?

P: …Ummh cause people will be pressurized cause they know if they go test they will be given something. If you give me something for getting tested, people around me will have questions and ask if that place where you tested are they judgemental or not. If they are not they would go that’s what’s important for me

I: Uumh ok them uumh so let’s say cap, t-shirt ok bottle isn’t. Which one of these would you rank first, if I was to say choose one that you want us to give you when you come test which one would it be?

P: Ummh I think you can give me a t-shirt

I: Ummh

P: So that the others can see that this person got a t-shirt, it’s important so that when the other person asks where you got it from; you say at the clinic, they ask what were you doing there? I went to test for HIV

I: Ummh How is this one? What about it will make people come and go get tested? Is it a normal t-shirt like mine?

P: Ummh lets say it will be similar to yours I think that’s fine

I: Ummh

P: People will be interested because it’s nice, plus they will love it its nice, and they can grab their friends and say let’s go test

I: Ummh and then the cap how will it look like?

P: Uumh, I cap maybe I think it can be black let’s say

I: Ummh

P: And then it can have this

I: What badge?

P: This one

I: The one for {XXX} (name of an organization)

P: Yea

I: Why {XXX} (name of an organization)?

P: [laughs] Cause uumh cause you told us a lot about HIV and testing you see things like that

I: Ummh

P: Yea I think it will be ok

I: So you think if people come and give you t-shirts from Aurum

P: Yea they will be interested

I: To test?

P: Yea

I: Ok and then the bottle?

P: I think red

I: Red bottle?

P: Yea

I: Just plain red? Is anything written on it?

P: Ummh maybe they can be written {XXX} (name of an organization)

I: Ummh ok, and then is there any other thing you can think of? Maybe bottles, caps, t-shirts anything else?

P: Aah eehm I don’t have anything to add

I: You don’t? Ok let’s say we don’t give you any of these what do you think the challenges will be?

P: I honestly don’t know, aah eehm I don’t know

I: Do you think we would get the same amount of people coming to test?

P: No

I: Why do you think so?

P: Because they will think that if we go to the clinic they will judge us, they will also shout at us and talk a lot of things and pass a lot of judgement as if they don’t know anyone living with HIV or like they don’t get sick

I: Ummh

P: But if there are t-shirt, bottles and cap they will come

I: Ummh, but even though these things are there will still be people that pass judgement at the clinic either way, so do you think

P: I don’t think they will judge you

I: Why?

P: The most people out here are very sick shame serious, but if there is something that they are given for testing they are more likely to be interested and encourage each other lets go get tested. At least if there is an HIV room only it would be better

I: Ummh ok. So caps, t-shirts and bottle how often do you think?

P: They must be given?

I: Must they give them once after they test?

P: After you test, and every time you come and test you must be given something

I: Ummh ok, why everytime?

P: So that people

I: What if we give them only once

P: Once?

I: Uummh

P: They will give up and not come and test again, like they will have the mind that the last time I got this and that time nothing, why must I come again

I: Ummh so you think that people come to the clinic to test cause of these things that they get? What if these things are not there?

P: If these things are not there only a few will come test to know their status otherwise most will be encouraged by these things

I: Ummh

P: Yea

I: Ok, did you say you have a phone?

P: Yea I have it

I: Ummh ok how do you think we can use the phone to make sure that the youth with phones gets information about HIV testing’

P: Ummh facebook, uumh twitter, whatsapp, instagram, yea

I: Ummh how can we use all these things to get people to go testing HIV?

P: Ummh I think we can write then post on instagram or facebook so that people can see the information then read then they can become interested in this thing

I: Ummh ok

P: Yea

I: And then you mentioned social media you said facebook, integral, whatsapp, twitter

P: Yea

I: Out of the four which one do you think is the important one that’s the most effective one?

P: It’s Instagram

I: Why do you think so?

P: Because most of the people have instagram and they see a lot of things on Instagram, there are only a few that don’t have it. And then if you get your information then you post it on Instagram then you get a lot of people

I: Ummh

P: Yea can get a lot of people interested in everything you post

I: Ok and then on this Instagram like what kind of messages would we posting?

P: Mum like eemh like write about HIV yea, that what is HIV, what kind of disease it is and so on

I: Ummh ummh

P: After they receive the message some will agree and then others will disagree

I: and then let’s say instagram is not there don’t you think face book. Don’t you think we could do the same thing on face book?

P: Face book maybe few

I: Mum

P: Maybe few people but the most they are on instagram

I: Ummh

P: Few are on facebook maybe fifty or more than

I: And then, so let’s say for example instagram who would be posting there?

P: Maybe it’s you or me you can write and then we can post so that people can get a lot of information HIV

I: Uummh

P: Yea so that others get information and can go and test

I: So you think you and your friends maybe let’s say maybe your friend posts on instagram and says “guys it’s important to know your status and things like that” do you think that would encourage you to go test? When you see it

P: If I see it, if it makes sense yea I will go and get tested. Like write my own information then I post

I: Ummh, what do you mean makes sense?

P: If she writes information that is straight, yea you see if the information is straight about HIV that won’t have yea it will be ok

I: Ummh

P: Yea then I would be interested

I: And then how would you feel about, let’s say someone tell you that HIV testing services are offered here at the clinic and you have to register with your phone how would you feel?

P: Uummh…maybe I will feel ok, cause my name will be there and I will see others get tested and I will also be encouraged to test. But I cannot go by what someone says if I don’t want I don’t want

I: Ok, so and then you mentioned that maybe we can use social media on the phone whatsapp and stuff

P: Yea

I: How can we use the phone to reach people?

P: …I think you can post pills ARV’s then write all the information.

I: But that also social media

P: Yea

I: I mean without social media how can we use the phone?

P: Uuumh…I think it would be ok if people go from house to house and come and talk about HIV like all the information about HIV

I: Uumh ok so you think people that go from house to house would be better than using a cell phone?

P: Yea those that go from house to house are ok because the most they can be found at home and can be tested yea I think that’s fine

I: Ok, so and then the challenges of, like people that don’t have the experience of being contacted by cell phone what are those? Since we know that not everyone has a phone

P: Ummh

I: So how do we reach those people?

P: Those without phones?

I: Yea

P: Ummh I don’t know, I don’t want to lie there I don’t know

I: Uuumh

P: At least using a phone you can connect with most people, eish I don’t know

I: We want to hear from you, not everyone has a phone

P: Yea

I: And then we need to make sure we educate people about HIV testing right, so information testing services how do we get it to people without cell phones?

P: …The way I see this

I: Ummh,

P: I think it would be better that someone tells them about HIV. Ok then the person can tell them all the information about HIV that is complete and makes sense, so after that one can make a decision to go get tested

I: Uumh

P: That’s what I think

I: And then for those with phones what do you think is the good thing about phones?

P: Maybe you can call them, or maybe talk on whatsapp or facebook

I: But then what’s good about it? What’s the good thing about it?

P: Eish! What’s good about phone?

I: Ummh, contacting someone using a phone to tell them about HIV testing services instead of maybe word for word [laughs] I mean face to face [phone vibrates]

P: I think the good thing I think you can call them and tell them about HIV. But it’s all the same a person can answer and not really listen but at least if its face to face its better

I: Ummh

P: Yea

I: So you think face to face will be more

P: Yea but it also has disadvantages, because you can tell someone something and they won’t really listen

I: When you say face to face that is between you and someone from the clinic or between you and your mother or your father talking about HIV testing services. Who is this face to face person that you speak of? Who are you talking to?

P: Ummh maybe someone from here at the clinic so that I get information about HIV so that, and if they have their testing kit they can test me there and there then

I: Ok, so you spoke of facebook, instagram, whatsapp and twitter right

P: Ummh

I: What other social media platforms can we use? Do you know of any?

P: Internet

I: Ok

P: …Yea internet

I: Uuumh

P: On the internet you can get a lot of people

I: Ok, then let’s say you have a phone right

P: Ummh

I: And then we contact you regarding HIV testing services let’s say I send you an sms

P: Uumh

I: And say “it's important to know your status, uumh its important to adhere to treatment if you are HIV positive ABCD & E regarding HIV testing services.” Your mother or someone you stay with how will they fell about it seeing these types of messages on your phone

P: Ummh aah ha! They won’t feel comfortable they will be dissapointed cause if I didn’t tell them they will have this thing that I lied. When she sees those things she will be disappointed she might not talk to me or she will be angry with me

I: Ummh

P: Yea things like that so

I: Why do you think she will be disappointed if she finds health information on your phone?

P: Cause other mothers

I: Uuumh

P: Some mothers cannot understand when you tell them about HIV that mom HIV is this and this. Other mothers are not ok when you tell them about HIV they will ask how old are you that you concern yourself with things like this, and they shout at you. It worse because if you ask what HIV is they will not answer you that are why they feel disappointed.

I: Mhmm

P: Yea

I: So how do you think your mother wants you to get HIV testing services? HIV information testing services if you don’t get it via the phone

P: If I don’t get it via the phone it will be better if someone from the clinic comes home to talk to my mother so that she can understand like the way you did with us yesterday we were able to understand it will be better if we all sit down and talk and discuss about HIV.

I: Ummh

P: Yea it’s ok

I: Ok, so you think using a phone or social media and then what about social media let’s say your mom sees your facebook that someone posted about HIV and the importance so of knowing your status you think she will be disappointed as well?

P: No

I: Why do you think it will be different with facebook?

P: Uuumh I think she won’t be disappointed because she will know that on facebook everyone posts what they feel like and one can learn about HIV but I don’t think she will be disappointed about facebook

I: Uuumh

P: Yea cause only a few people will see my status

I: But let’s say that the message I send on your phone is the same message is the same message sent on facebook it’s saying the same thing so you said your mom will be disappointed if she sees the sms

P: Yea

I: And then she sees the message on facebook, why do you think she will be disappointed with the sms than the one facebook message? How's that? Maybe something that we post on instagram and your mom sees it

P: [sighs heavily] Let’s say disappointed cause I didn’t tell her about HIV and she also didn’t tell me. So I think that she will be disappointed and might get angry, if it’s a mother that has no sympathy but one that is understanding they will understand

I: Ummh but why do you think parents become disappointed when they see information maybe about HIV testing services on their kids phones?

P: Because other mothers don’t want their kids to be affected with HIV and other mothers don’t teach their kids about HIV

I: Ummh

P: They become disappointed and are filled with guilt about why didn’t I tell my child about these things, and they will feel bad seeing these things

I: Ummh, ok and then those suggestions that you came up with that the youth come to the clinic you said maybe have an HIV only site or HIV only room at the clinic. The other you said we give them caps, t-shirts and bottles

P: Yea

I: Then the third one is maybe post on social media or what not. Any other suggestion

P: I don’t have any

I: Uummh

P: Yea only those that I mentioned

I: Like with the HIV only room isn't how do you think it will help? Because people will know that room is for HIV, so how will that help the youth come to the clinic to test

P: It will help if you get that you are positive no one will know besides the person that tested you and if they are a professional they will not judge you

I: Ummh ok do you have any final thoughts you have about the youth, HIV testing, incentive that you mentioned t-shirts, caps, bottles. Is there anything else you want to add?

P: Ummh ummh

I: To cap, t-shirt, bottles

P: No I have nothing to add

I: Ok, so uumh give me an example, ummh you said one thing that’s important to you about incentives, the three that you mentioned caps, t-shirt and bottle is t-shirt.

P: Ummh

I: Let’s say t-shirt are not there, what’s the next thing you said cap right, so take away the t-shirt and cap do you think people will come for bottles only?

P: No

I: Why do you think so?

P: Cause a few people want to see evidence that if I go to the clinic I will get this and bottles are not sufficient to interest people

I: Uuumh what do you think we can give them on top of the bottle?

P: Ummh, no I don’t know. I don’t know

I: For you

P: For me I don’t have anything to say

I: So there’s nothing we can give you so that you come to the clinic and get tested?

P: Ummh no

I: Mmmh

P: …You can come without the other things

I: Like?

P: Bottles, caps and t-shirts

I: Uuumh

P: Others might come without wanting anything but most they want things that are evident like I tested and I got this in return, without including the pills

I: Uumh. You said without pills?

P: Uuumh

I: Please explain what do you mean by that?

P: Uuumh putting the pills aside as we know that those are given for free for them to take so I meant something tangible

I: So you meant besides the pills that they are given at clinic there should be other things they must be given other things ok

P: Oh! God it’s hot

I: Ok sorry we are almost done. Any other suggestions or you are done? Any final thoughts maybe anything you want

P: I have nothing else to add

I: Ok and you have said you have never tested right

P: Yea

I: So are you ever going to get tested maybe ever for HIV?

P: Maybe, yea maybe because people do mistakes, so if I make a mistake then I will come and test

I: Ok so you will only come, you are only going to come if there is a mistake

P: Yea

I: So without that you would not wanna know your status?

P: No I am scared

I: You are only scared of blood

P: Eish I am scared

I: Ok so we are almost at the end of our discussion I just want to thank you for taking part in this interview

P: Ok

I: Ummh the time is 17:29 half past 5

P: Aah half 5

I: Yea, thank you

End time: 17:30
