# Supplementary material for: Exploring perceptions of low risk behaviour and drivers to test for HIV among South African youth
Source: PLoS One. 2021 Jan 22;16(1):e0245542. doi: 10.1371/journal.pone.0245542 (PMC7822253; doi:10.1371/journal.pone.0245542)
Supplement: S1 File — (ZIP) [file pone.0245542.s001.zip › S1_File_Anonymised Transcripts/YA01-016-TT_Translation_QC2_TM.docx]

Full Participant ID: YA01-016-TT

Participant Type: In-depth Interview

Location: Winnie Mandela

Date: 24^th^ August 2018

Start Time: 09:55

Primary interview language: English (Setswana)

Name of Facilitator/Interviewer: Bakang Mosime

Name of Note Taker:

Name of Transcriber: Lineo Matsela

Length of recording: 26:30

Label Key

I = Interviewer

P = Participant

N = Notetaker

{ } = Indicates that details were changed or pseudonyms were used to anonymise data

xxx = Words were omitted to anonymise data

- = Breaking into a sentence by the next speaker

…= Pause or drawn out words

[ ] = Indicates noise made, e.g. [laugh], [sigh], [pause]

[inaudible segment] = Unclear section of the recording

?Mulenga Clinic?, ?P3? = Questionable text or doubt as to what was said or who said it

I: Qualitative interview: PID YA01-016-TT. Participant type: male. Location:

Winnie Mandela Clinic. Date: 24^th^ August 2018. Start time is 09:55. Primary language: English. Interviewer: Bakang Mosime. Do you allow me to record this conversation?

P: Yes.

I: Thank you. Uhm can you describe to me your thoughts about HIV?

P: HIV I think uhm [clears throat] it’s been uhm here, it’s been uhm here around since before the the development. Because we, back then we didn’t have the…the knowledge or the specific word for it.

I: Mmhm

P: That’s why people didn’t know about it.

I: What do you understand about HIV?

P: HIV is uhm, I think it’s a disease.

I: Okay. How can you become infected with HIV?

P: Through sexual intercourse or if you’re helping someone who’s uhm HIV…positive and then you have a cut.

I: Mmhm. What kind of sexual intercourse are we referring to?

P: [Pause] The sexual intercourse I mean uhm having sex with uhm [long pause]

uhm unprotected sex.

I: Okay. Tell me what places a person at risk for H…is highly at risk for getting HIV? (Which places is it that) they are more likely…it’s more likely to eh for one to get infected by HIV?

P: Uhm, I think eh (at the nightclubs)

I: Okay

P: (At parties)

I: Mmhm

P: Yah

I: And how is that so?

P: Because…one as an individual (when they go to a party, they have a) mindset (of what they are going to do). Yah, so, (you will find that if I go to a party I want to have sex with) a certain girl so…

I: Oooh okay.

P: Yah.

I: Unprotected sex?

P: Yah.

I: Okay. Can you tell me about any situation when you felt that you may have been at risk of getting HIV?

P: A situation…

I: Any situation whereby maybe (you thought that) maybe you were more likely to get infected by HIV?

P: [Pause] I think it was when I was, yah, dating two girls.

I: You were dating…?

P: Two girls

I: Okay.

P: Yah.

I: What was happening?

P: Uhm, I think I found out later that the second one was cheating on me so…yah.

I: Ooh so it was the moment you were at, highly at risk?

P: Yah.

I: Okay. And you were having unprotected sex?

P: Mmhm

I: Okay. Can you tell me about HIV testing services that is testing place in your area?

P: [Pause] Uhm.

I: Uhm, any testing services? (Where have you seen) HIV testing services ( in the area where you live)?

P: Uhm, (I haven’t seen any in the area where I live, but) near my school there was…I think, yeah.

I: Okay. (What was it)? (Was it a clinic or)…?

P: They said uhm (they test people) so…

I: Okay. (What do they test for? What were they using)?

P: A test, a test kit. (They had put up a small structure)

I: Okay.

P: Yah.

I: So, where are the HIV testing services for youth usually are delivered? (Where does the youth access those uhm testing services)?

P: (At clinics);

I: Mmhm

P:(At hospitals).

I: Okay.

P: Yah.

I: Can you tell me about your experience accessing these services? Maybe (you’ve gone to test). (Which experiences did you have)? What made you maybe (to withdraw) from getting tested?

P: It’s because I was with my friends so when you come out they want to…they will want to know what is your status.

I: Okay. That’s what made you not to test?

P: Yes.

I: Okay. [Pause]. So was that the only experience of uhm accessing these services or are there any other experiences?

P: No.

I: Okay. In your opinion, what is positive about the current HIV testing services that are available to youth?

[Knock on the door]

P: I think that being near, like near to us [pause] [voices in the background], where we can reach them, like I was…like I said, they were near my school, so I could go and get tested.

[Noise in the background] [Pause]

I: So, was it uhm the positive current HIV testing services that are available to youth?

P: Yah, I think so, yah.

I: When you talking about positive uhm, positive opinions maybe, I’m referring (to: what is positive about the HIV testing services that are…that are nearby for the youth)? (What does the youth gain from that)?

[vibration]

P: Uhm, the youth, the…oh? what they gain?

I: Mmhm…

P: Uhm, not much actually.

I: Not much?

P: Yah.

I: Why are you saying that?

P: Because uhm, if uhm, I want something, then you have something, I have to give you what I have to in order to get what I want.

I: Okay, can you explain more about that?

P: If uhm, like testing services (that are nearby, they can introduce maybe the idea that) if you get tested you’ll get uhm social media data’s then maybe yah.

I: Okay.

P: Yah.

I: So, what will be the negative aspects of the current HIV testing services? (Which negative aspects are there) for testing services that are available to youth?

P: The youth, they are looking for something so if you…you don’t have what they want, they won’t give you what…

I: They won’t get tested?

P: Yah.

I: Okay. [pause] How do you think incentives could be used to encourage youth to test for HIV and access treatment?

P: Uhm, the youth uhm, these days are glued to their phones, that’s why social media data’s comes in. If they say you get tested and then you’ll get this amount of data’s then maybe they will come.

I: Okay. So, by saying that, you think incentive can influence or encourage you to get tested and get their treatment?

P: Yes.

I: Okay. What is your understanding of the word “incentive”?

P: Something that uhm encourages you to do something.

I: Something that encourage you to do something?

P: Yah.

I: Okay. Please describe the types of incentives that youth value, which could encourage them to test HIV or access treatment. The types of incentive. (Isn’t it) you’re ? Agree? it’s something that you give so that one can do something?

P: Yah

I: Okay, so what are this type of incentive? What do you think youth value more?

P: They value social media’s and fashion, clothing. Yah, they’re all about clothing.

I: Can you give me the types maybe of clothing that they will appreciate?

P: T-shirt and…and the caps. Labels…not just uhm an ordinary t-shirt.

I: Oh? Labels?

P: Yes.

I: And what more?

P: And then maybe uhm a ticket to a soccer match to watch their favourite soccer team play.

I: Ticket to a soccer match?

P: Yah.

I: Okay, what more?

P: Uhm, maybe a ticket to see uhm your favourite singer.

I: Okay.

P: Yes.

[pause]

I: Is that all?

P: No, and social media data’s, yah.

I: Data?

P: Yah.

I: Okay. Do you think that will be all?

P: Uhm yah, these days they’re all about that. But uhm, me uhm, through education maybe if they get tested and then if you’re a matriculant and then you’re given a registration fee.

I: Registration fee for…for what?

P: For your choice…. university of your own choice. Yah [pause].

I: Okay.

P: And the parents, if they’re encouraged…actually, if the same encouragement that we get at school we get at home, maybe yah, then they’ll get tested.

I: So, you need encouragement?

P: Yes.

I: What sort of encouragement?

P: From our parents.

I: Mmhm

P: Yes.

[pause]

I: And how often do you think this incentive should be given?

P: I think every, yah, every now and then.

I: Every now and then?

P: Yah, maybe…. yah, after maybe 3 months. Yah, 3 months.

I: Okay.

P: Yah.

I: What could be the challenges of providing this incentive for HIV testing?

P: Challenges…uhm, maybe not having access to, to a certain network. Maybe like me, I don’t have a phone so, if I come here and get tested and then I’m given a voucher of data’s then maybe…yah.

I: Oh, you don’t have, you won’t be having a phone to use that data on. That could be the challenge?

P: Yes.

I: Okay. And what could be the benefits of providing this incentive for HIV testing services?

P: Maybe… yah, they will…they will get uhm more access to social media. And if others they see me wearing a label t-shirt then they like and then they will ask me where did I get it, and then I will tell them: because I got tested then I got this.

I: Okay, what message do you think we should put on the t-shirt, that can attract other youth to get tested?

P: I think “wear your status”.

I: Wear your status?

P: Mmhm

I: Oh. That’s good. And do you think uhm you’ve said all the benefits that you can think of.

P: Yah, sort-of.

I: Okay. Please describe to me your thoughts about being contacted via telephone or social media for HIV testing services.

P: Uhm…

I: Do you think it would be a cool thing to…to get a message giving you information about HIV testing services or the testing services that will be taking place in your area…do you think you can take that call and listen and follow what…[inaudible segment]?

P: Nah. I think it should be through uhm, networks. Like, they should uhm maybe if you start with this uhm testing thing, you should talk to Cell C, MTN and all those network and then they will send the message, if you get tested to your nearest what-what you get this amount of data’s you see? Then…

I: So uhm you won’t have a problem getting a message or a telephone call from uhm someone from the clinic uhm telling you about…

P: A message. A message is…yah, better than call.

I: Okay, so you’re thinking youth will read that message and be interested?

P: Yes.

I: Okay. Can you please describe some examples of how you have been informed about HIV testing services?

P: How I have been informed about HIV services…

I: Mmhm

P: Uhm, as friends we talk so that’s where…yah

I: They would tell you.

P: Yah, they tell about it.

I: Okay. Is that the only way that you’ve been informed?

P: Uhm, and then (pamphlets as well), and then (even people from the clinic) if there is a testing…yah, that is happening nearby, they will tell you: “get tested here”.

I: Okay. Do you read those pamphlets though? As youth, do you read those pamphlets that you’re given?

P: They read when uhm, they think they might be in danger then that’s when they read.

I: Okay. How could you feel about being informed and registering for HIV testing services using your cell phone? (How would you feel if you were informed about HIV testing services, or maybe if you were registered) for uhm coming to get tested for HIV through your phone?

P: Uhm. Yah, I think it’s a good thing because uhm nowadays we are always having our phones everywhere we go so…

I: So, you won’t get maybe intimidated or feel like you’re being exposed?

P: Hmm. Somehow, yah, I would feel like I’m being exposed.

I: But you will appreciate it?

P: Yah, I’d appreciate because yah.

I: Okay. How could cell phone be used to inform youth of HIV testing services? How do you think we should use cell phone to inform youth about their HIV testing service…the HIV testing services that are taking place around their area?

P: Like I said, uhm, if Cell C or a certain network that you’re using then…then send you an SMS saying uhm there is this testing taking place, yah, around this place, maybe if you’re nearby then you’ll go.

I: Okay.

P: Yah.

I: Please describe any challenge that youth might experience if they are contacted on their cell phones for HIV testing services.

P: The challenge?

I: Mm. (If I’d call you and tell you) about uhm HIV testing services, what can be the challenge there?

P: Maybe if (it gets answered by) someone else instead of me, yah.

I: Okay.

P: Yah.

I: So, (you wouldn’t want) that person to hear about that?

P: (To know that)…yah.

I: Okay. And what would be the benefits of contacting youth on their cell phones for HIV testing services? (What benefits are there)?

P: They’re always having their phone so it’s the easy way to get through them so, yah.

I: Okay so youth are always on their phone so...

P: Mm.

I: Okay. In your opinion, what types of other social media should be used to contact youth for HIV testing?

P: Uhm. Yah, Facebook, Instagram, Twitter [Pause]

I: Okay. And how are they going to access these?

P: Uhm because they’re always uhm on their social media’s so that’s when they…

I: Okay.

P: Yah.

I: What are the challenges for using social media to contact youth for HIV testing services? (Which challenges will they have) if maybe we get those kind of promo messages about HIV testing services on social media?

P: Hmm…challenges…

I: Mmhm.

P: I don’t see any challenges.

I: Hm?

P: Yah. I don’t think there will be any challenges.

I: Do you think that they will read those promo’s, they will get into, maybe if there’s a link, they’ll open that link to read?

P: Yah, if I’m curious then yah. Yah, yah, I think they will.

I: Okay. Through the help of the data that they will [inaudible segment].

P: Yah, they will get.

I: Okay. What would be the benefits of using social media to contact youth for HIV testing services?

P: [Pause] Hm.

I: (What are the benefits)? Isn’t it we just spoke about their challenges?

P: Mmhm.

I: And what would be the benefits of using social media to contact?

P: Because they’re always on social media’s. Yah. That’s the benefit. They’re always on social media’s so they’re able to see: okay, there’s this certain thing happening.

I: Maybe (you can explain more about), uhm, (how they will be important to get youth to) come and get tested?

P: [Pause] I think knowing your…your status, it means uhm if you’re HIV positive then you’ll get treatment early.

I: Okay.

P: Yah.

I: So, do you think social media (has) influence and impact on youth…

P: Yah

I: …that much that maybe if you can use it to convey this message to them, they will come?

P: Yah.

I: Okay. How do you think your parents or your legal guardian would feel about receiving information on HIV testing services on your cell phone or social media?

P: My mum…uhm, my mum she’s kind so she doesn’t have a problem with me getting tested.

I: Then if you were to get messages or (promo messages about) HIV testing services in your social media or cell phone…maybe (we call you when you’re with your mum) how do you (think that she’ll react) to that?

P: My uhm my mum will encourage me to do so.

I: Will encourage you…

P: Yah. Yah. Because she believe that the healthier uhm, the healthier me, the happier me.

I: Okay.

P: Yah.

I: Okay. Can you tell me about any other suggestion that you think may have…you may have, [vibration] which would encourage you to test for HIV?

P: Hm. Suggestion…could be… let me see [pause]

I: Any. What do you think? (What can we do here to…to attract the youth to come) for uhm (testing for HIV)? What’s your suggestions, (that) maybe (we didn’t mention) on our conversation?

P: Uhm. [Pause] Hai! So far I think uhm we’ve been mentioned all of them. Oh. Entertainment, sports events, uhm… yah

I: Okay. Okay. So, entertainment, how? What do you think we should do?

P: Maybe if there is a testing site and then you bring a certain celebrity to perform there, then yah, maybe they’ll come. Yah.

I: Oh? So, usually youth they are attached to celebrities.

P: Yah. They’re all about the fame.

I: Oh. So, you just want to see that celebrity come perform there?

P: Mmhm.

I: Okay. So, entertainment and HIV testing services can…can make an uptake in HIV testing services?

P: Yes.

I: Okay. Any other thoughts, maybe…that you may have?

P: [sigh]

I: Suggestions?

P: Maybe the church.

I: Church?

P: Yah.

I: Okay.

P: People…most people they respect, you know, what comes from the church so they value that. Yah.

I: Mm. So, does youth go to church?

P: Yah!

I: Okay.

P: They go.

I: So, from church…from who should they get these messages from? Maybe the pastor or what?

P: Yah, the pastor, maybe.

I: Okay.

P: Yah.

I: Okay. Oh? So, do you think it would be relevant for you as a young person to get tested at church? Maybe if we decide to come to church, have a tent, and ask for you to come test. Do you think that will be relevant, it would be a nice thing?

P: Uhm, no.

I: Mhmm.

P: The church is a place of worship, so if they go there, they want to be free that’s where you get to be free so…

I: So, you don’t think it’d be a good idea?

P: Mmhm.

I: Okay. But it’s okay for the message to be conveyed in the church?

P: Yah.

I: Okay. Uhm, are there any final thoughts you have about youth, HIV testing services or incentives?

P: Uhm, I think you should get tested to know, you know, earlier than to know later when it has changed to another stage maybe into AIDS. Yah.

I: Okay.

P: Because that’s when it’s going to be practical to treat it.

I: [Vibration] So it’s good to get tested at an early age…at an early stage?

P: Yah, so that you get treatment early.

I: Oh, okay. Okay. That’s a good thing. Any other thoughts on incentives?

P: Nah. [pause]

I: Think about something that maybe you forgot to mention earlier on. [pause] Anything.

P: No. So far, I think uhm, I’ve mentioned…yah.

I: Okay. So, you’ve mentioned t-shirts, caps, tickets to soccer match, ticket to festival. During this soccer match and tickets to festival…during these festivals, do you think uhm, there should be someone spreading the word about HIV testing services, how important it is?

P: Uhm. [pause] Yah.

I: Okay.

P: Before, yah.

I: Before the match and before the festival?

P: Yah.

I: Okay. Okay, thanks for having this conversation with us. Are you happy with everything that you’ve said, or you want to add more?

P: No. So far, yah, I’ve said it all.

I: Okay. Thank you once again.

End time:10:22.
