# Supplementary material for: Exploring perceptions of low risk behaviour and drivers to test for HIV among South African youth
Source: PLoS One. 2021 Jan 22;16(1):e0245542. doi: 10.1371/journal.pone.0245542 (PMC7822253; doi:10.1371/journal.pone.0245542)
Supplement: S1 File — (ZIP) [file pone.0245542.s001.zip › S1_File_Anonymised Transcripts/YA01-017-CM Translation_QC2_TM.docx]

Full Participant ID: **YA-01-07-CM**

Participant Type: Male

Location: Winnie Mandela Clinic

Date: 23 August 2018

Start time: 13:05

Primary interview language: English

Name of Facilitator/Interviewer: Bakang Mosime

Name of Note Taker:

Name of Transcriber: Reba Ramoroka

Length of recording: 33:29

Label Key

I = Interviewer

P = Participant

N = Notetaker

{ } = Indicates that details were changed or pseudonyms were used to anonymise data

xxx = words were omitted to anonymise data

- = breaking into a sentence by the next speaker

… = pause or drawn out words

[ ] = indicates noise made, e.g. [laugh], [sigh], [pause]

[inaudible segment] = Unclear section of the recording

?Mulenga Clinic?, ?P3? = questionable text or doubt as to what was said or who said it

I: Do you allow me to record our conversation?

P: Yes…

I: Thank you. Can you kindly describe to me your thoughts about HIV? What do you understand by HIV?

P: By HIV?

I: Mhmm…

P: Uhm, according to my understanding…I think, HIV it’s a virus whereby you get infected through accident. If maybe somebody is got an…is got HIV {and maybe you were in the same taxi as them}, you have an accident and then it will affect you or unprotected sex… Yah. I think…

I: Okay. How can…you become infected with HIV?

P: How can…I become infected with HIV?

I: Mhmm… [turns page]

P: Yah things like unprotected sex, using same toothbrush with someone who is infected…

I: Mhmm…

P: Or…maybe accidentally so when you’re trying to help someone then find out someone is bleeding has HIV and you have a scar on your hand then you are trying to help…then you end up catching HIV.

I: Okay. I heard you {you spoke about} toothbrush, how do you…one gets, get infected through toothbrush?

P: Through toothbrush?

I: Mhmm…

P: {You’ll find that maybe I’m brushing my teeth with a toothbrush} maybe I’m infected, then you now when you brush your teeth sometimes {you bleed on your gums}?

I: Mhmm…

P: So you catch on the toothbrush then someone else comes and uses it…then gets infected.

I: Mhmm…okay. Tell me about places a person is mostly, uh, at risk of getting HIV? Maybe any place you can think of whereby you think that place its more like, [snaps tongue] {it could put you at risk of getting HIV}?

P: HIV?

I: Mhmm…

P: I think mostly {where you could get HIV} its, maybe {where} prostitutes {are}… When you going to the prostitutes everytime and you do have sex then you end up having sex with prostitutes cause other people {you’ll find that they} they don’t prefer using condoms… So they end up getting infected. That’s one place, maybe {at beers or somewhere}.

I: {Where?}

P: Maybe {at the beer places} {XXX} (Name of place)cause thats where I hear people saying they go the most.

I: Okay. Is it the only place you know {where you think you could get HIV from}?

P: Nah…I don’t think it’s the only place.

I: Okay other places?

P: Other places? Uhm, I think HIV {where could you get it?} Lemme see, uhm…like a place, just a place?
I: Yah…any place. Where you think-

P: -Just a place?

I: One can get in danger of getting HIV?

P: Uhh…{XXX} (Name of place), I think there are countries where there are a lot of HIV like {XXX} (Name of place)…I think. Some place in {XXX} (Name of place), I’m not there cause I think thats where they say there’s a great, or a tarven…actually.

I: Okay… Whats happening in a tarven?

P: In a tarven maybe you just dancing then you get drunk, you see a lady then you try to approach her then end up taking her, going to the toilet and then…see? Start having sex with that person then…

I: Mhmm okay, without protection?

P: Yah.

I: Okay…

P: Then you get infected.

I: Okay. Can you tell me about the…any situation when you felt like you may have been at risk of getting HIV?

P: Mhmm, I think it was grade eleven when I was helping my friend.

I: Mhmm…

P: Cause {my friend fell at the stairs} and he was hurt badly so {we didn’t have gloves} at that time.

I: Mhm…

P: So I had to help him cause he was my friend then…yah. But, I don’t think he had HIV cause I would’ve been showing right now.

I: [laughs]

P: I don’t know how [laughs]… Yah.

I: Okay…so since the incident happened, you never had…got, got tested for HIV?

P: No.

I: Okay. So how will you know {whether} he was not HIV and you didn’t get infected at that time?

P: Uhh {what makes me think that I didn’t get infected} is because I didn’t have the symptoms {of HIV}.

I: Ohh…

P: Yeah.

I: Okay. What are those symptoms {of} HIV?

P: I think its…I’m not sure but I think its coughing blood and then starting to be skinny…

I: Mhmm…

P: And then vomiting…some things like that.

I: Okay.

P: Yah.

I: Well can you tell me about the HIV testing services that are taking place in your…in your area?

P: In my area?

I: Mhm. Or community.

P: Yah, yah I think some, some… {some people} there’s these people {that} go {and test you}. They come sometimes {with} those containers and then they tell us to test.

I: [turns page] Okay.

P: Yes.

I: So where are these HIV testing services for youth usually take place? [turns page] Where are they delivered?

P: Where are they delivered?

I: Mhm.

P: Sometimes they are delivered {at} {XXX} (Name of place)…

I: Okay.

P: And then… {The others I’ve seen were near {XXX} (Name of place)}

I: Thank you.

P: Some place near {XXX} (Name of place).

I: Were they you-, uh youth friendly services?

P: Yah I think they were youth friendly services cause they, they were allowing anyone to come and test and then you, you were testing for free and then your result {was given to you} same time.

I: Okay.

P: Yah.

I: Can you tell me about your experience accessing these services, maybe {you once tried going there} what were…what were your experiences? What happened?

P: What happened?

I: Mhm.

P: Uhm, I went there {but I was scared to test}, when it comes to {them injecting me on the finger with an injection} then I felt like they used that injection on someone else and I might get infected. That {that’s what made me…like be scared}.

I: Okay.

P: Yah.

I: Was it bad experience or a good experience?

P: Uuhh I can’t tell {if its} a bad experience or a good experience but for me it was bad.

I: Okay.

P: Cause {I was scared}.

I: In your own opinion {right?}

P: Mhm.

I: What is positive about the current HIV testing services that are available to youth?

P: What are the?

I: {What is positive about} HIV testing services that are offered to youth?

P: Uhm…the positive thing {is} they do it for free, and then they don’t, I don’t think they just go, come to you then {test you} at the same time. They gonna ask you if you are sure you wanna test…

I: Mhm.

P: and then…yeah, they tell you some things before you test {that it is good to test} because of this and this and this. And then, this and this and this {I could actually put which way} cause sometimes {you find that you have HIV and then you are scared of testing} then when you starting to be sick, {you start having AIDS} you know?

I: Mhm.

P: Then {after you have AIDS}, found out its too late for you to… Esp-, especially when you sexual active.

I: Okay.

P: {You find that} its too late for you to test, {the you get ARVs} and then, yeah.

I: Okay. And then you told me about the positive aspects {right?}

P: Mhm.

I: Now what do you think are the negative aspects of current HIV testing services?

P: Sometimes, {as people aren’t the same} so you can test sometimes, after testing, then you find out you are HIV then your life doesn’t be the same anymore cause you know you be living with that thing {of} you have HIV and you feel that you were different from other people.

I: Mhm.

P: Yah.

I: Okay so what do you think {is going to make a person feel like their life is still the same even though they are HIV positive?}

P: I think that one {is when you get treatment}.

I: Mhm.

P: And then most of the people think {that} the don’t wanna tell anyone if they have HIV, they rather grab it inside them until {they die}.

I: Mhm.

P: So…I think the treatment and then living the good life as you can, that will make you feel better. {Make you feel like you don’t have that HIV.}

I: Okay, that’s good. How do you think incentives could be used to encourage youth to test for HIV and access treatment?

P: Uhm…

I: {What do you think are the ways that’s could-}

P: {-That could...}

I: {That you could think of that could encourage youth to test for HIV and to get treatment, which ways?}

P: {Which ways?}

I: Mhm.

P: I think {the ways that could make the youth test for HIV} is for, for their parents to encourage them. To do that first, then…so that {they can follow, they should have a leader} someone that will lead them to do that thing. Cause sometimes to be alone doing something is very bad cause we know {that the kids of today go with peer pressure}, if one of your friends {goes and tests} then maybe you can be inspired to {also go test}.

I: Okay. So do you, do you think incentives can encourage youth to get tested?

P: What?

I: Incentives?

P: What are those…incentives?

I: Uhh, lemme get into the next question. What do you understand by the word incentive?

P: Incentive?

I: Mhm.

P: Incentive, I think is more like gifts…

I: Okay.

P: Is more like gifts. Yah. something that you…give to people. Yah. Maybe as a present. E.G lemme say, you see the {XXX} (Name of cellphone network service provider) promotion?

I: Okay.

P: Yahh. They give people like, things like caps, bottles…if you do something first, if you buy airtime first. And then… Yah.

I: Mhm…

P: Maybe I think, if you get them something in return, they’ll do that.

I: They’ll do that...? Get tested?

P: Yah.

I: Okay. That’s a good one. Please describe the types, like we, we, we explained incentives right?

P: Yah.

I: So give me the type of incentive that youth will value?

P: {What?}

I: Which could encourage them to get tested? {What kind of incentives would the youth like…?} Just to encourage them to get tested?

P: Just to encourage them to get tested? I think we can start by a simple thing like, data. [laughs] You know the youth {love using phones and everything}

I: Okay.

P: So if you can give them data, maybe prize winning…yah, something like a phone. Uh, one of them will win it.

I: Mhmm…

P: The one who gets lucky can win it. Then what again? Uhm…uhm, uhm, uhm maybe some shoes. Maybe shoes, some dope shoes…

I: Dope shoes, meaning?

P: Yahhh. Dope shoes [laughs] or sneakers. Sneakers, design shoes or maybe a nice shirt or a dress. Yeah.

I: Shirt or dress?

P: Yah.

I: Okay. What are others?

P: Then maybe…get them food after the test, yah.

I: Okay. Okay. Is that all?

P: Yah. That’s all.

I: Okay. [tongue snaps] And how often do you think these incentives should be given…to…to those people? To the people who get, who get HIV testing services? How often?

P: How often?

I: Mhm.

P: I think if you do it like…once or twice a month cause giving everyday {even the people who are testing lose.} Like we have to all gain so I think if we do it once or twice a month. Cause {a lot of people come} obviously…trying to test.

I: Okay.

P: Yah.

I: Okay. [turns page] what could be the challenges of providing in this, providing these incentives? What could be the challenge?

P: The challenge {would be that} everybody will want something from that. But maybe its an event that was hosted, like everybody will want something in return, like and then we will…you cannot like provide everyone.

I: Mhm.

P: Cause you can’t provide everyone a pair of shoe or provide anyone pair of dresses so…I think the {those are the challenges that you are going to face} by everyone wants something in return. And then {you find that} someone you give them, maybe you give them data then its like I don’t want data, I want shoes.

I: okay.

P: {Then you go to another one, and the other says, no} I don’t want shoes, I want data. Yah I think that will be…

I: So {how can we address these challenges?} What do you think, how do you think we can address them?

P: Oh {how can we address them?}

I: Mhm.

P: I think we can address those by making people understand, like making the youth understand {about} how does this work.

I: Mhm.

P: Maybe gonna tell them {that} uhm, maybe after any three months, you can come and test. And, what again? Mhm…and then you can say who comes first like, {those that come first,} maybe twenty or nine-teen people that comes first then obviously {all of them will come, then when they come in masses, some wouldn’t know whether they came first or if they didn’t} so it will be a trick to…to…

I: To get them tested.

P: Yah, to get them tested,

I: That’s very good. What would be the benefits? We just talked about the challenges, {right}? What would be the benefits of these incentives?

P: These incentives?

I: Mhm.

P: I think the benefit will be {that} they will feel like {they left with something and also for them in return for testing} maybe you find out that you were negative. You go happy, something like…maybe {you leave with something going back home, you that even though you tested}, then I got something in return for testing so again {it will make it a good thing to them to test}. {That} at least you go something. Yah.

I: Okay…uh, please describe your thoughts about being contacted via telephone or social media for HIV testing services.

P: For HIV?

I: Yah, what are your thoughts being contacted like “hello, YA01 ca you please go to test, uh we having a testing campaign”, HIV testing campaign, what would be your thoughts about that or getting a message on social media everytime like, “get tested at your nearest mall or clinic”, what would be your thoughts?

P: Ohh. My thoughts in that? I think that would be a good idea but still it would be some, something like a bad idea cause when, once you see that thing. You gonna have that pride {of} no, I don’t have HIV…

I: Mhm.

P: See I don’t have HIV, {I’m fine} I don’t wanna go for testing. I know where to test when I want to test so but I, when I say it’s a good idea, I think it’s a good idea because there are people {who want to test but they} live like far away from clinics and everything where they can test so if they post information where to test, when to to test; I think that would be better.

I: Okay.

P: Yah.

I: Thank you. Can you please describe some example of how you have been informed about your HIV testing services?

P: Okay. {The way they told me like} they, first time {right?} Like, they called me first…we were walking past the street.

I: Okay.

P: Then there was two girls and boy then they were calling us… “Hey, come test, come test”.

I: Mhm.

P: Then [pause]… Where again?

I: Is that the only way {they called you…the way you got information?}

P: Nah…Nah.

I: What do you think are other way {to get} information for testing services?

P: Yah. I, I think you can get them through pamphlets…

I: Mhm.

P: And then posters…

I: Mhm…

P: Yah.

I: Does youth read those posters and pamphlets though?

P: Some…

I: Okay

P: Some will read them but some just ignores. Cause {they know that no} we don’t wanna test and something like that but…yah I think-

I: -What…Okay. What do you think would interest them to read those posters and pamphlets? What do you think should be these posters or pamphlets?

P: On those posters?

I: Mhm…

P: I think the posters should be colourful and attractive more like {these posters for} one man show. And {that thing}, maybe if you say uhm, you are going to test and then you {show one celebrity that will be there} on the test. I think they will be attracted {to come}.

I: Okay. How would you feel about being informed and registering for HIV testing services using your cellphone? {How would you feel being informed about} the testing services taking place around your area or {registering you} to come get tested via your phone?

P: Yah, I think that would be better cause your phone is something private and then its something that you can use {to, to…like} to solve {appointments} cause once you {get yourself somewhere else and mabe we are walking with you and they call you, it will be like…you are calling them out that these people neeed to test…they have never tested}. So when it comes to a phone {with a} message...you know how, okay. Its mine then…I can go and go test.

I: And then how could cellphone be used to inform youth about testing services? Which way can we use to, to inform youth about HIV testing services?

P: I think you can open groups through Whatsapp. And then SMSes…

I: Mhm.

P: Twitter, Instagram…yah.

I: Okay…

P: Social media. Yes, actually.

I: Okay. [turns page]

P: I think.

I: So do you think social media is…can influence youth to…get tested?

P: Yeah.

I: Okay. [turns page]

P: Yeah I think it can cause most people of the youth {are always on social media}.

I: Mhm. Okay

P: And then once they grab the information right there, some people obvious they will comment that “oh I’m going to test”

I: Mhm.

P: “I’m going to test”, then I think more of the people will end up testing.

I: Okay.

P: Yah.

I: Please describe to me any challenges that youth might experience if they are contacted on their cellphones for, for HIV testing services?

P: On the what? Repeat the question…

I: Please describe any challenges {please explain to me the challenges that} youth might experience if they get, they get contacted for HIV testing services? {Which challenges are there?}

P: Uhm I think {the challenges that they will have} like, they will ask theirselves like, why…like {when you touch your phone and you see a message same time, you are like…} why? How do they know I’m sexually active? How do they know…you know things like that? They will question theirselves {that why I received them}. Where did they get my numbers? How did they get numbers? So yah…something like that.

I: Okay. And please describe the benefits of contacting youth on their cellphones for HIV testing services. What will be the benefits, {isn’t it} you just talked about the challenges-

P: -Okay the benefit? I think the benefit will be {that, like} what can I say? Mhmm… Yah, the benefits {will be} like as I said like, when you see like {message getting into your phone} sometimes you think oh, these people are trying to help me cause they basically know that I’m sexually active and then I have to go for testing cause they wanna provide this thing for me.

I: Mhm…

P: Yah.

I: Okay and in your own opinion, what types of social media should be used to contact youth for HIV testing services?

P: Facebook, Twitter, Instagram, Whatsapp.

I: Okay. Which one is especially influential?

P: Influential?

I: Its more accessible, lets say which one is more accessible?

P: I think Facebook because {at} Facebook theres free Facebook or Twitter, cause there’s free Twitter.

I: Okay.

P: That they provide it. Yah.

I: Okay.

P: For incase {for those people who don’t have data} can use that to get information too.

I: Okay. Okay. What are other challenges for using social media to contact youth for HIV testing services? What do you think could be the challenges of…-using social media, yah?

P: -Social media? Oh, yah the challenges I think {will be that} some other kids {who are infected} will feel bad cause some other people {you’ll find that they tested them} and you know that we are taking ARVs then once you put that poster then some people will comment like “no, go take ARVs…what, what, what, what”. You know…you see?

I: Mhmm okay.

P: Yah, I think those, that will be the challenges…of children who are infected.

I: Okay but again what will be the benefits?

P: The benefits?

I: Mhm. For using social media to contact youth for HIV testing services?

P: Oh, the benefits {are that} there are other children who are sexually active and then they didn’t find someone to advise about HIV testing and then if you post it on social media, yah some kids will be inspired cause…some kids their parents don’t want them to test for what reasons, I won’t explain.

I: Mhm…

P: Cause I don’t know. Yah.

I: Okay. How do you think your parents or legal guardian would feel about you receiving information on HIV testing services on your cellphone or social media?

P: Mhm, I, I…some…parents are different.

I: Mhm.

P: So some parents will feel bad cause they will feel like this child {has been having sex then went to get tested} then now they are sending her messages {to come and test again} because they know she’s sexually active and something like that.

I: {How about you} How do you think your parents or -legal guardians would feel?

P: -Or legal guardians would feel? Well my parents?

I: Mhm…

P: Uhm, [tongue snaps]. My mother is a street person so I wouldn’t be sure if she would be mad or go…cause she knows that I’m not that sexually active, you know? She knows…

I: Okay.

P: Yah. So she will feel a little bit bad.

I: So you think {that} she will won’t appreciate the fact that you getting this kind of messages or phone call for you get, uh, get HIV testing?

P: I think that will be good cause sometimes you don’t know what happened in life. Maybe, someone {hates you and they spill their blood} cause their infected in a juice {while visiting them} then you drink, get infected… Though you’re not having sex cause {they say} people think HIV {you only get through sex}.

I: Mhm.

P: So it cannot be through sex too, {you’ll find that somebody hated you already there} as I said, {they pour something} then you get infected then {you don’t test} cause not testing because you’re not sexually active so you won’t get HIV.

I: Okay.

P: That would be good.

I: So can you tell me about any other suggestion that you may have which would encourage youth to test for HIV?

P: HIV, yah?

I: Any other suggestions, maybe?

P: Yah…-

I: -That you think can work?

P: That I think can work?

I: To get youth to get tested?

P: I think we gotta sacrifice in some point. Maybe {we can call} like three celebrities or four so that they can come perform for them and advise them cause they are their role models.

I: Okay.

P: Cause {its their role models} obviously, when they speak. Maybe {they would listen to them}.

I: Mhm.

P: Cause sometimes when {our parents talk to us} we just don’t listen but when {it com to some one}, something like a role model {who talks to us saying} you must do the testing and what, what, what, what… {uh telling us a story about} how e get tested then a lotta people, lot of youth will get inspired.

I: Okay.

P: Yah.

I: Other suggestions, maybe that you have?

P: Other suggestions?

I: Mhm. To attract, lets say to attract youth to get HIV testing services to access HIV testing services?

P: Uhm…what can I say? Other suggestions…I think {its to inform them} yeah.

I: Yah.

P: To inform them through social medias and then make posters that, like whether they {do HIV testing} let it be fun.

I: Okay.

P: Like let it be fun, let it {have music}

I: Mhm.

P: And people dancing there just to attract…

I: Okay.

P: Just to attract the youth so that {the can also come} then test.

I: To get tested. What about accessing their treatment, how can we make them uh, adhere to the treatment, get their treatment everytime they have an appointment to get some? How do you think you can attract them?

P: I, I think through their cellphones.

I: Okay. Send reminders maybe?

P: Yah, I think…

I: And what are other ways you can think of?

P: Other ways that I can think of? Uhm, lemme see. Mhmmm… [pause] Hm. This one is difficult but [pause], I think you can go door to door knocking for them, telling them to come and take their treatment.

I: Okay.

P: Yah.

I: Okay. Are there any other final thoughts that you have about youth HIV testing services or incentive? Maybe just to add on what you spoke about regarding youth and HIV testing, incentive? Do you want to add something?

P: Yeah, I think the youth need inspiration from someone, someone that will inspire them hardly. So that they can get tested casue sometimes {where we stay}, we like, like people who’ll advise us.

I: Mhm.

P: Cause as kids {you’ll find that we are sitting and chatting right there} then one of us says I will never go for testing because of this and this and this…

I: Mhm.

P: Then the other person goes like… {we’ll all end up thinking the same thing}.

I: Mhm.

P So if there’s that one person that can inspire the nation to do that.

I: Mhm.

P: I think we need that kind of a person.

I: And…who should be that person?

P: Who should be that person?

I: Mhm. Maybe give me an idea, doctor, a nurse…uhh politician? Who should be that person?

P: The person. Yah I think we need a hero {shame}.

I: What kind of a hero? [laughs]

P: I can be that person [laughs].

I: You can be that person?

P: yeah. [laughs] I think I can be that person that inspires people.

I: What would you say exactly?

P: What will I say?

I: Mhm.

P: Okay. I will get there and I will be like “hi, hi” then I’ll ask the person their name and I’ll the person if they tested for HIV and then if they say no. Then I will tell them like uhm, you must test for HIV because you don’t know what happened in your life because even though you are not sexual active, you must understand that sometimes that HIV doesn’t only come because of…like, having sex but there are many ways to get HIV. Like, maybe you just walking {barefoot} then people were fighting yesterday, somewhere then there’s blood on the floor.

I: Mhm.

P: And then maybe, you get cut by {a bottle} then you step on that blood, then you get infected, then you don’t know cause most of the people think getting HIV is all about sex and which is wrong. Which is not…

I: Mhm, Mhm…Yeah.

P: And then some people are wrong by telling people {that} when they have HIV is because they sleeping around with a lot of people. And then I think that’s one thing that makes us be afraid to go test. Cause when {the results come} and you are positive, you’ll ask yourself “what happened?” Cause I never had sex and everything like that and if we can, we can try to advise people to tell them that HIV is not all about sex, there are accidents. I think that will help us a lot.

I: Which hero would {you} want to come {to encourage} you to get tested?

P: {Encourage?}

I: Mhm. Who do you…who will be that person?

P: I think-

I: {-Who can come to you and tell you} YA017, go get tested?

P: Uhh, I think someone who’s close to me, people who are close to us who cares about us.

I: Okay.

P: And maybe your girlfriend, your friends…your aunts, someone, like someone in your community that has an ability.

I: Okay. Now we’ve come to the end of discussion. Thank you for your participation. If you have any questions, the numbers are there. I showed you the numbers [page turns]. You can call between eight and five o’ clock.

P: From eight, {tomorrow?}

I: If you have any question you didn’t understand or you want to know more about the stud…

P: Okay.

I: You are, feel free to contact us and thank you once again.

P: Okay.

End time: 13:40
