# Supplementary material for: Exploring perceptions of low risk behaviour and drivers to test for HIV among South African youth
Source: PLoS One. 2021 Jan 22;16(1):e0245542. doi: 10.1371/journal.pone.0245542 (PMC7822253; doi:10.1371/journal.pone.0245542)
Supplement: S1 File — (ZIP) [file pone.0245542.s001.zip › S1_File_Anonymised Transcripts/YA01-018-SD Translation_QC2_TM.docx]

Full Participant ID: YA01-018-SD

Participant Type: 15 years-old, female

Location: Winnie Mandela Clinic

Date: 30 August 2018

Start time:

Primary interview language: IsiZulu

Name of Facilitator/Interviewer:

Name of Note Taker:

Name of Transcriber: Nokukhanya Ndinisa

Length of recording: 35:13

Label Key

I = Interviewer

P = Participant

N = Notetaker

{ } = Indicates that details were changed or pseudonyms were used to anonymise data

xxx = words were omitted to anonymise data

- = breaking into a sentence by the next speaker

… = pause or drawn out words

[ ] = indicates noise made, e.g. [laugh], [sigh], [pause]

[inaudible segment] = Unclear section of the recording

?Mulenga Clinic?, ?P3? = questionable text or doubt as to what was said or who said it

I: Thank you so much for allowing us to do this interview. Uhm, do you allow me to record this interview?

P: Yes.

I: Okay, thank you.

P: Thanks.

I: [Clearing throat] Okay. Can you tell me what your thoughts are about HIV? What is HIV according to your thoughts?

P: I think, eh, HIV [pause] I think HIV is the disease that is popular for killing people. But if you take treatment well, it won’t kill you.

I: And then, how do you get infected with HIV?

P: Like what?

I: How do you get infected? How can you get HIV?

P: HIV, maybe you can get it from sleeping with someone with HIV without using a condom or when you touch the blood of someone with HIV-

I: Uh hmm…

P: That is how you get it.

I: And then like… So, have you- [pause] Have you ever been in a situation where you saw that you were at risk of getting HIV?

P: Yes.

I: Can you tell me about it?

P: My friend, but she didn’t have HIV, she had AIDS. So, I was even scared of eating her food cause I thought that when I eat her food, I will get AIDS. So-

I: Hmm…They told you that that’s how you can get infected?

P: I used to think so on my own, then I heard that if I touch her blood, that is when I can get HIV/AIDS.

I: Hmm… Okay, can you tell me about other situations where you felt that you, not your friends were at risk? Have you ever felt that you’re at risk of getting HIV?

P: No.

I: Okay, and… okay and, have you ever tested for HIV before?

P: No, I have not tested.

I: Why haven’t you?

P: Because I don’t think that I have HIV or that I will have it. That’s why I don’t test.

I: Why do you think that you won’t have it?

P: I don’t think so cause I have not slept with a boy and I am scared of touching people’s blood. Uhm… I also trust that my parents don’t have it.

I: Okay, but in the future, do you think that you would want to test for HIV?

P: I will- yes.

I: Huh?

P: Yes, as time goes, I will test.

I: And then, what will make you want to test?

P: Maybe… What will make me want to test for HIV? Maybe if I will sleep with a boy, I will want to test because I don’t trust him.

I: Uhm… Only that?

P: Yes.

I: So, you sleep with someone you don’t trust? [pause] okay… So, do you know any testing services around Winnie maybe, where you stay?

P: The people who- [pause] Yes, there are people who maybe drive around in a car, looking for people who want to test, they go there.

I: So, you only know them or?

P: And the clinic.

I: Okay, so where would you want to test?

P: I would want to test at the clinic because I don’t trust the people who test people in the streets. So, I would test at the clinic.

I: So, do you think that testing at the clinic will be better than testing in the streets?

P: The clinic is better because they have their laws, they are gifted, and they have studied for that. Those in the streets, I don’t know if they studied or not. I can’t trust them, and I don’t know if they want to take my blood somewhere.

I: Okay. So, you’re saying that you’ve never had any experience of testing, whether at a clinic, school or anywhere else?

P: No.

I: Okay. So, if people come to your school-

P: People from the clinic?

I: Yes. Would you want to test?

P: Yes. I would want to test.

I: Why?

P: Cause I trust people from the clinic, I would want to test.

I: Okay, and why do you think it will be important to know your status?

P: [Sigh] Because I must always know if I’m positive or negative.

I: To do what?

P: So that I can take treatment and get help.

I: Okay. So, you spoke about like people… You mentioned the clinic, and-

P: The streets.

I: Streets, yes and school, right? Where you know that they test for HIV.

P: Yes.

I: So, can you tell me, what is the positive thing about testing? So, you’ve already mentioned that you trust the clinic, right?

P: Yes.

I: So, what is positive about testing in the street?

P: Maybe, they test you and then you don’t know where your blood is going or if they are selling it to {XXX} (Reference to specific nationality)or what, I don’t trust them… Or they didn’t study for what they are doing, maybe they can give you wrong answers, saying you are positive or negative.

I: Hmm…

P: I don’t trust them.

I: Okay, so if people come to school to test learners, do you think it’s a good thing for them to come to school than testing in the streets?

P: At the clinic? [pause] I don’t understand.

I: So, you said that people can test at the clinic, in the streets-

P: Yes.

I: And schools, right?

P: Yes.

I: So, I’m asking what is the good thing about testing at school compared to testing in the streets?

P: Oh!

I: Hmm…

P: Testing at school?

I: Hmm…

P: Maybe doctors from the clinic can be sent to schools to test learners cause HIV is all over at school and some children are scared of going to the clinic.

I: Why do you that that they’re scared of going to the clinic?

P: Yes, some are scared cause I’m also scared that people will think that I am going to do an abortion or I’m going to prevent and things like that.

I: Okay, and then [pause] Uhm, one of the negative things you talked about is that people are scared to go to the clinic because of what people will say about them.

P: Yes, what people will say about them if they go to the clinic.

I: Okay, and then the negative thing about testing in the streets? You said you don’t trust them.

P: Oh, testing in the streets? Like what?

I: A wrong thing about testing in the streets?

P: A wrong thing about testing in the streets?

I: You said you don’t trust them, I want another point.

P: Another [pause] Actually, the ones on the street can give you a wrong answer, maybe they will say you are positive and that’s why I don’t trust them because [pause] I’m scared that they will give me a wrong answer.

I: Eh, you said you if you test in the future-

P: Yes.

I: You’d prefer testing in the clinic?

P: Yes.

I: Okay, and then at the clinic you don’t trust- you don’t think that there are people who can-

P: No.

I: Give you a wrong answer?

P: No.

I: Why don’t you-

P: At the clinic, I trust them cause they studied for this thing and its legal. In the street, maybe it is not legal, I don’t know.

I: Okay. So, when you think of incentives, what do you think when I talk about-

P: Incentives?

I: Hmm… What are they?

P: I think it’s the people who test HIV, they go around giving people condoms and pads to girls, if a boy goes to test, they give them condoms but if you are a girl, they give you pads.

I: And then you… What incentives would you want in order for you to come and test at the clinic?

P: I… For the pads.

I: Okay. .? P3?

P: Huh?

I: What else?

P: Yes, pads and condoms.

I: Aha… So, do you think if they were to give you pads, you’d come to test at the clinic?

P: Yes.

I: Why?

P: I don’t understand.

I: So, you think that if you come to the clinic and someone tells you that if you test for HIV, they’ll give you pads, would that make you come and test for HIV?

P: Yes.

I: Why? I want to know why pads and not anything else.

P: Pads cause that’s what we use… Uhm, pads are right.

I: And anything else?

P: And condoms are right cause some may have sex without condom cause they don’t have them or else.

I: Hmm… Okay, but then condoms, like we’ve been giving them to a lot of people and still we don’t see a lot of people coming to test, especially around your age. So, we wanna know, what else can we-

P: Give them?

I: Hmm… What else do you think can work?

P: For people to-

I: Come and test?

P: Come and test? Maybe uhm, money cause people really love money, especially people my size. They can come and test when they say you will get money, so a lot of people can come.

I: How much?

P: Maybe around R50 or R100, people can come. If maybe they say when you test you get food, they’ll come cause they like food too much.

I: So, you think condoms, pads, money and food will work?

P: Yes.

I: Why do you think so?

P: Cause many people really love money and food. Where there is food, there are many people.

I: So, for you, the first thing are pads, right?

P: Yes.

I: So, let’s say there are no pads, there are condoms, food and money. What would you take?

P: I’ll take money.

I: Why?

P: Cause money can buy the pads that I didn’t get. Money can buy a lot of things.

I: Like what?

P: Maybe [cough] Maybe I can save it and on Saturdays, I can buy stuff that I want. I can also buy things that I need, if I need school things, I can buy them.

I: School things like what?

P: When I want books, I can buy them or when I want crayons or glue, I can buy them.

I: Stationery?

P: Yes.

I: So, do you think that, let’s say we give you stationery, not money. Would you still be interested to come?

P: No.

I: Why?

P: I can buy… Money is needed.

I: So, for you, out of the things you’ve mentioned, money is the most important thing?

P: Yes.

I: So, let’s say there is no money.

P: No money?

I: Hmm... What else there or you are thinking of something else?

P: I can come. I can come for pads and condoms.

I: We know that already they are not working, people are not coming for these things and other things, right? So, let’s say money is not an option, condoms and pads are also not an option. What else are you thinking about?

P: What I think can make people come?

I: Yes.

P: Uhm, clothes, maybe like t-shirts [pause] maybe bottles. Just anything. There are many things.

I: So those t-shirts, can you maybe just paint a picture for me on how they look?

P: The-

I: T-shirt, is it just a normal t-shirt, white, blue, green? How are they?

P: Different colours cause some people don’t like being looked down on. Maybe if they get purple t-shirts, some won’t want to be looked down on when they’re wearing them because it can be obvious that they got them from the clinic. I think many different colours, white, blue.

I: Okay, and then the bottles?

P: Normal, beautiful bottles.

I: How are they? Explain to me. [laugh]

P: Maybe {XXX} (Name of company that distributes plastic ware).

I: Okay, let’s say out of all these things, you must choose top three of things to give the youth at your age as incentives so that they can come to test at the clinic. What are the top three you can choose?

P: Food-

I: Food, uhm…

P: Stationery, school things and t-shirt.

I: T-shirts, right?

P: Yes.

I: So, you mentioned eight things. You mentioned condoms for boys, pads for girls, money, food, books, crayons, stationery or t-shirts and bottles, right?

P: Yes.

I: So, you think that like, how often should we be giving these things out? How often, like once, twice, three times or every time they come to the clinic?

P: Every time they come to the clinic. Like every time they come, they get. That can bring in a lot of people.

I: Why do you think that will bring many people?

P: Cause if one comes to test, they will tell others who will also tell others. This will spread, and many people will come.

I: And then what do you think can be the challenges of giving people these things? What are those challenges? The challenges-

P: Of giving these things?

I: Yes.

P: So that they come to the clinic?

I: Like, we give you something so that you come to the clinic. So, what are the problems that can come from these things?

P: The problem that can come because of this? Stealing maybe? When people are used to getting food and money from a certain place, they can steal.

I: Steal these things?

P: Yes, they can steal. The stuff- things that they work with and they sell them.

I: Okay, so what are the good things? What do you think are the good things of providing these things so that people can come to the clinic?

P: To test for HIV cause it’s all over and some don’t want to test. Some want to test cause we’re not sure if you’re positive or negative.

I: So, you think that, let’s say one of your friends doesn’t want to come and test at the clinic because they are scared. And then now we tell them that if they come to the clinic, we will give them bottles after testing, do you think that will make them to stop being scared?

P: Yes, but they are afraid of people, so they will continue being scared. So, if they are scared, they must come with someone like me, a friend, so that they are not scared.

I: Okay. So, you get information about HIV/AIDS via {XXX} (Name of a non-profit organization), the after-school programs, right?

P: Yes.

I: So, can you tell me, how else do you think the information about HIV can be accessed?

P: Something else?

I: Yes, since you get it via afterschool sessions?

P: Yes.

I: So, how else do you think you can get it? I mean information about HIV.

P: Maybe I can research on a phone.

I: A phone?

P: Yes, I can get information that way.

I: And what else?

P: At school they teach us and at the clinic.

I: So, when you research using phone, and-

P: Yes.

I: So uhm…[sigh] how else do you think we can use a phone to tell you about HIV testing services?

P: How? Like-

I: How else do you think we can use a phone?

P: Use a phone?

I: How can we use a phone?

P: To tell us about HIV?

I: Yes.

P: Uhm… I think… to research, we can research about HIV.

I: And then how else? We spoke about research-

P: WhatsApp and Google.

I: What else?

P: [pause]

I: And then what do we do with WhatsApp? How would you wanna get information?

P: Yes, you will get phone numbers of people, then ask for information.

I: From who?

P: From people who test or those who teach at {XXX} (Name of a non-profit organization)at school.

I: So, you’re saying WhatsApp, people from {XXX} (Name of a non-profit organization)will get all of your numbers and then-

P: They can give us information via WhatsApp.

I: And then using Google, how?

P: Google, maybe… People from {XXX} (Name of a non-profit organization)can put their things, post on Google, maybe a paragraph about HIV and then when you google, you will get it.

I: Okay, and what do you think about using social media? Do you know social media?

P: Social media?

I: Like Facebook, do you know them?

P: I do.

I: What do you think about using them?

P: Using Facebook? Yes, it’s right cause you can find out about HIV there.

I: And then besides Facebook, WhatsApp and Google, what else can you use on your phone?

P: Uhm… Calling, maybe?

I: Calling who?

P: People from {XXX} (Name of a non-profit organization)or clinic doctors.

I: So, doctors will call everyone?

P: Ha ah! No, I’ll call because I am the one who needs to find out about HIV, I must call.

I: So, let’s say in your class, everyone is like you. They’ve never tested for HIV before, so doctors from clinic must call everyone?

P: So that-

I: They come and test.

P: [pause] Yes.

I: Do you think that’s possible? [laugh]

P: Yes.

I: And how do you think that it’s going to work?

P: Eh... but he-eh! It’s right.

I: Why isn’t it right?

P: Some don’t have phones and they can call their parents and maybe their parents don’t want them to test cause like if they test, they can think a lot of things like maybe they’ve slept with a boy.

I: So, for those without phones, how do you think we can reach them?

P: For the-

I: Maybe for information about HIV testing.

P: It’s right that they come to school to attend and be taught like at {XXX} (Name of a non-profit organization).

I: And then for those who don’t go to school?

P: Don’t go to school-

I: And they don’t have phones, how else do you think we can reach them?

P: By going around the streets and testing them.

I: But you’ve said that you-

P: I don’t trust them, the people who walk around the streets. For those without phones and not at school, I think it’s right for them.

I: And then if they also don’t trust them?

P: They’re supposed to go to the clinic then. Maybe, like every day at 3, people who want can attend at the clinic. Then they can write on a board outside that people can attend at the clinic to get information about HIV.

I: Okay. And then, uhm… You said challenges of using a phone is that other people don’t have it. What are other challenges?

P: Other challenges?

I: Hmm... Problems that can happen when they use phones to contact with people, like you said information won’t reach those without phones. So, what other challenges can you think of?

P: Come house-to-house. Doctors go house-to-house and tell people about HIV.

I: I mean problems that can come from contacting with people using phones or maybe telling them about HIV testing services. So, one of them is that some people don’t have phones. So, even if you send them something via a phone, they won’t get it. So other challenges?

P: Other challenges is that they will get HIV.

I: They will get it?

P: Uh, those without phones, like… maybe uhm…I think they will get HIV because they don’t have information. They don’t have information.

I: Uhm…. And then those ones, how can we reach them?

P: How can we get them to test?

I: Since they don’t have phone, no WhatsApp, no Facebook. So, how can we get them? Like your age peers.

P: Put boards in every township… maybe {XXX} (Name of a non-profit organization)their things written that maybe you can come and attend at the clinic to get information about HIV.

I: Do you think people will come?

P: Yes, they will come. Since they will read that when they walk in the streets, then you will write HIV is now all over, so people must get information. So, they will rush to the clinic.

I: Okay… So [pause] Challenges? Let’s say someone else is not on WhatsApp or Facebook. You say they can get HIV, can you explain to me what you know?

P: They can get HIV because they don’t have information, they don’t care about getting HIV, so they trust other people.

I: Okay. So, what I’m going to ask you now is that do you think that there is any good thing about contacting young people, I mean on their phones. Do you think there’s any good thing about contacting them, maybe via a phone?

P: Yes, it’s a good thing.

I: What are the good things?

P: When you call them, it’s right cause they can come, you can tell them to meet you somewhere to get information about HIV. But sometimes it’s not right because wrong people can call them to come and get information about HIV but they want to kidnap them that way.

I: Hmm… You know what I’m trying to understand?

P: Yeah?

I: You are saying that maybe like someone at the clinic should call these people to come to the clinic? So, where can they get these people?

P: Uhm…

I: Like for you, if I was not at your school, I would not have met you and I wouldn’t have been able to call you to encourage you to come for HIV testing-

P: How would you get me?

I: Yes.

P: Oh! Stand in busy streets and ask for people’s phone numbers and tell them that you are from the clinic to ask them to test at the clinic.

I: Hmm… So, let’s go back to the phones. So, let’s say you receive information about how to use condoms, how to stay safe from HIV and anything that is HIV-related. How do you think your mother would take it? Or your parent? How will your mother or father take it if you get HIV testing information on your phone?

P: They will think that I sleep with a boy.

I: So, you don’t think they will be happy that you get information even if it teaches you?

P: They will be happy cause, yes… I must take care of myself.

I: So why do you think that other parents won’t be happy that their child get information on their phone?

P: Maybe they think that… They think that… that, I don’t know. Please repeat.

I: So, since you say that your parents will be happy that you get information if you had a phone. So, I’m asking that why do you think that other parents won’t be happy that their children get information about HIV.

P: Oh, so those parents don’t like teaching their children. So, maybe they won’t like it when another person teaches their children. Some think it’s not right for children to know about HIV.

I: So, let’s go back to suggestions that you came with about how we can make people to come and to the clinic for HIV testing, right?

P: Hmm…

I: So, one of the things you said we can use… uhm WhatsApp, Google, Facebook. You said we can give boys the condoms, girls’ pads, and all other incentives that you mentioned, right?

P: Yes.

I: And you said, you don’t trust those people in the streets. Can you tell me other suggestions that you have that can work for someone your age to come to the clinic? How else do you think we can get other people to come to the clinic for HIV? On top of giving them condoms.

P: You can tell them that HIV has spread all over. So, they must come and check if they don’t have HIV. If they have it, they can get treatment.

I: Uh-huh… [pause] but don’t you think that’s already been done? Cause everywhere you go, you see boards explaining that HIV does this and this. But still they don’t come. So, from yourself, what would you do so that they come, knowing that the youth likes this and this?

P: [pause] I can follow them to their home, take them to the clinic to test.

I: Like one-by-one?

P: Uhm, I can go house-to-house and get the youth.

I: Will they agree?

P: Yes, since there are things. I can beg them and say that when you are done testing, you get food, money-

I: Do you think they will be happy to know their statuses?

P: Yes, they will be happy to get treatment.

I: Okay. So, we’re almost at the end of our interview. So, I wanna know your final thoughts about the youth, maybe your age. If you have any thoughts about HIV testing, the incentives you spoke about… Anything you wanna add?

P: No. [background noise]

I: So, do you think that HIV is a problem amongst the youth? Or is it only for older people?

P: I-HIV is for every one, even young people can get it.

I: But do you think that the youth realises what a big problem it is? Or that’s why they-

P: They don’t come to test? Maybe they trust other people even anyone they see in the streets, even when they see them maybe fat and think that maybe they think HIV is for thin people.

I: So, is there anything else that you wanna add to what we spoke about?

P: No.

I: I see. We’re almost- actually we’re at the end of our interview.

P: Thank you.

I: I just wanna thank you for being part of the study.

P: Thanks.

End time: 16:24
