# Supplementary material for: Exploring perceptions of low risk behaviour and drivers to test for HIV among South African youth
Source: PLoS One. 2021 Jan 22;16(1):e0245542. doi: 10.1371/journal.pone.0245542 (PMC7822253; doi:10.1371/journal.pone.0245542)
Supplement: S1 File — (ZIP) [file pone.0245542.s001.zip › S1_File_Anonymised Transcripts/YA01-019-LN_Transcription_QC2_TM.docx]

Full Participant ID**:** A01_019_LN

Participant Type: Female 15-17

Location: Winnie Mandela Clinic

Date: 30 August 2018

Start time: 15:45

Primary interview language: English

Name of Facilitator/Interviewer: Bakang Mosima

Name of Note Taker:

Name of Transcriber: Nokukhanya Ndinisa

Length of recording: 25:22

Label Key

I = Interviewer

P = Participant

N = Notetaker

{ } = Indicates that details were changed or pseudonyms were used to anonymise data

xxx = words were omitted to anonymise data

- = breaking into a sentence by the next speaker

… = pause or drawn out words

[ ] = indicates noise made, e.g. [laugh], [sigh], [pause]

[inaudible segment] = Unclear section of the recording

?Mulenga Clinic?, ?P3? = questionable text or doubt as to what was said or who said it

I: Do you allow me to record this conversation?

P: Yes. [background noise]

I: What is your understanding? Describe to me your thoughts about HIV.

P: Uhm… I would say HIV is not a good thing to our- HIV is not a good thing into our country cause like it affects a lot of people and cause a lot of people to have a low self-esteem in them.

I: How can you become infected with HIV?

P: Uhm, others were born with HIV, others are getting HIV with unprotected sex.

I: Okay. Tell me, what places a person at risk of getting HIV? In which places, most of the times, can you end up with HIV?

P: Like age?

I: Places, places. Which places?

P: I don’t understand.

I: Uh, which areas can you find yourself at a high risk of getting HIV?

P: Uhm… Should I mention provinces?

I: No, only mention places that you can tell me about.

P: At {XXX} (Name of place), at {XXX} (Name of place), are they sharp?

I: [Silence] What happens at {XXX} (Name of place)? What were you saying about {XXX} (Name of place)?

P: You said you wanted places that have HIV.

I: Where you can find- Where a person can find themselves feeling that I’m at risk of getting infected with HIV.

P: At a tavern.

I: Okay.

P: Yes.

I: Why at taverns?

P: Cause… Cause like it’s not safe. So, like, there are old people. It can happen that you become drunk too much and then they kidnap you.

I: Okay. Are these the only places you know?

P: [pause] Yes.

I: Okay. Can you tell me about any situation where you felt like you may have been at risk of getting HIV, maybe?

P: Meaning like going around and sleeping with many people?

I: Okay. So, yourself, at what instance? In which instances have you felt that this time I’m at risk of getting HIV, maybe?

P: So far, I’ve never felt like that.

I: Can you tell me about HIV testing services that take place in your community?

P: Okay, {XXX} (Name of place),

I: Okay

P: And next to my home, like the opposite street.

I: What is happening there?

P: They test the youth so that they can know their status.

I: Who is testing there? Like, is it nurses or it’s community members?

P: It’s nurses.

I: So, where are the HIV testing services for youth usually delivered? Where does the youth go to test?

P: Clinic.

I: Which clinics? Which clinics accommodate youth?

P: {XXX} (Name of a private clinic.

I: Okay. Can you tell me about your experience accessing these services? Have you ever been there to test? What happened there? Please explain how your experience was to me.

P: Like, my attention when I go to test, I wanted to know what my status are like so that if pos- I’m negative, so that I can take treatment easy before my time. So- my mom told me that HIV is a very dangerous disease in our community. So that’s why-

I: Did you test?

P: No.

I: Why didn’t you test?

P: Uhm, [pause] I thought it- I thought it was not my time to test because I never sleep with anyone.

I: Okay. When do you think is the right time to get tested for HIV?

P: From 16 years and above.

I: Okay. In your opinion, what is positive about the current HIV testing services that are available to you? What is positive about this whole of putting tents on the streets for people to test?

P: So that they come and test what kind of status they have in their life.

I: Is that the only positive thing?

P: Yes.

I: And what are the negative aspects, maybe?

P: The negative would be like, if like, they don’t know their status, they will be surprised when, like if the time like is almost over for them, they realise that they have HIV on them without knowing.

I: Okay, that’s good. How do you think incentives could be used to encourage youth to test for HIV and access treatment?

P: Uhm, like posters like all over their city so that they can know more.

I: Okay. So, how should incentives be given? [silence] What is- Do you think that incentives- How can we give incentives so that people can access HIV testing? Should we go to their home or maybe they should come to test and then we give them incentives?

P: They should come and test because like going to their homes, we are so many in the city.

I: Okay. So, what is your understanding about the word incentive?

P: Uhm...

I: Anything. Just tell me what you understand.

P: This word like, it means like what are the things we should do to the city so that thing of HIV- the percent of HIV can decrease.

I: Okay, okay. So, please describe the type of incentives that the youth would value them to access treatment or HIV testing services. Which incentives that the youth can value if we give them to get tested or access treatment for HIV?

P: The clothes.

I: What kind of clothes?

P: Like the… the long jeans.

I: Okay.

P: The long jeans and- like they should wear like things that are like uhm, 7 to 8 centimetres long, not three.

I: We’re talking about incentives, right? Incentive you said that it’s something that you can give someone to change behaviour, right?

P: Yes.

I: So what kind of incentives should we give people? What should we give people to test?

P: ?P3?

I: Yes, anything.

P: Clothes, eh… we can support with books-

I: Okay.

P: And food.

I: Food, okay.

P: Hats, caps.

I: Caps?

P: Yes.

I: Okay.

P: Time, [pause] watches.

I: Okay. Are these the only things you are thinking about?

P: Yes. [pause] ?P3? Yes, and airtime.

I: [Silence] Okay. [pause] Uh, please- How often do you think these incentives, should these incentives be given provided? Should be provided for HIV testing services? How often should we give the things you mentioned, like the clothes, books, food? When?

P: Uhm, like today ?P3?

I: Like when? How often? Should we just give them, or we should wait for people to test, then give them or?

P: Yes, they can test first cause if we give them, they will be more interested.

I: Oh, so do you think that these things will make them to come and test?

P: Yes.

I: Okay. [background noise] and what could be the challenges when providing these incentives for HIV testing services?

P: The challenges would be like from people- [ringing cell-phone] people who stay far, they would have to get them transport to come- to fetch them to come here because like it would be far.

I: Okay, and what would be the benefits? What can they benefit? What would be the benefits if we give the community the incentives for HIV testing services?

P: The benefits would be- they would be proud about themselves cause they would be knowing what standard they’re living in life.

I: Okay, that’s good. Please describe me your thoughts about being contacted via telephone or social media for HIV testing services.

P: Eh-

I: Do you think it is a good thing if I have to call you or text you via social media to let you know about HIV testing services?

P: I think it would be good if you call me.

I: Okay, why do you think calling you would be best?

P: Cause like eh… Calling would be best cause I would be understanding more than typing on the phone.

I: Oh, okay. [pause] Can you please describe some examples of how you have been informed about HIV testing services?

P: [silence]

I: Do you understand me?

P: [silence]

I: Can you describe examples- Can you give me examples of how you have been informed about HIV testing services?

P: How did I test?

I: No. How have you received information?

P: Oh like-

I: Did they call you or give you something?

P: No, like- there were like the- people who work in the clinic, in the streets, so they went house-to-house to inform girls about more, like they told us that HIV is the most disease that we have so that they would like to test us if like we have HIV, they asked parents for permission so that they can test us.

I: Oh, and why didn’t you test that time?

P: I tested because I wanted to know-

I: Why didn’t you test since you said that you’ve never tested before?

P: Yes.

I: So why did- didn’t you test? Why didn’t you test, that time?

P: I thought that it was not my time cause like I was very too young.

I: Okay. How would you feel about being informed and registering for HIV testing services using your cell phone? How would you feel if maybe we say there’s a registration method for HIV testing and [pause] How would you feel?

P: I’ll feel [pause]

I: Will you be happy maybe about registering on your cell phone or getting a message saying register for HIV testing services or if we tell you about HIV testing services, would that make you feel comfortable?

P: Yes.

I: Why would you be comfortable?

P: Cause like, I’ll be more- I’ll be like what is HIV actually, cause it seems like it’s a serious disease.

I: How could cell phones be used to inform youth about HIV testing services? How can we use cell phones to alert or to make the youth aware of HIV testing services?

P: [silence] Like social media [door knock]

I: Social media and what?

P: And Facebook.

I: Facebook and what?

P: WhatsApp.

I: WhatsApp and what? Are these the only things?

P: Yes.

I: Please describe challenges that youth might experience if [cough] is they’re contacted on their cell phones. Which challenges do you think that the youth can find we call them to inform them about HIV testing services?

P: The challenges that they can have is that maybe their phone is off, or they left it at home and they’re not around.

I: Okay, since well we talked about challenges, what benefits could that could be?

P: The benefits like would be that they will know more about this HIV, what is actually this HIV.

I: In your own opinion, what social should we use to contact youth about these HIV testing services?

P: Uhm, WhatsApp, Facebook and yes-

I: Do you think that it would be easy for youth to access these social media?

P: Yes.

I: Why are you saying that?

P: Cause like majority of the people in our city, they’re more- they concentrate more on Facebook and WhatsApp

I: Oh, they con- that they stay on Facebook most of the time?

P: Yes

I: Is that all that you can think of, maybe?

P: Yes, cause if it’s not on Facebook, it’s WhatsApp.

I: Okay. So, you said what could the challenges of using social media to contact youth be?

P: The benefits?

I: Hmm.

P: The benefits would be, they would know more about this thing of HIV.

I: And what would be the challenges?

P: The challenges would be, if like they don’t know about it, like they won’t- like they won’t know about what it is, so you find someone with it, but they didn’t have time to know early so that they can take treatment early before.

I: So, do you think eh, social media would help to spread the message for HIV testing services?

P: Yes.

I: Okay. How do you think parents, say your parents or legal guardian would feel about you receiving information about HIV testing services on your cell phone or social media?

P: My parent would feel like- they will be… They won’t be happy, but they will be like, why do I receive message about HIV, maybe I have it. She’ll be more depressed about it.

I: What will depress her?

P: She will ask herself many questions, about maybe- why do I get message about HIV, maybe I go around sleeping with people.

I: Hmm… So, what kind of ?P3? do you think we can do to send these message to your phones but not to scare parents? What do you think we should write or do?

P: Uhm, like when you type, parents must be included in this thing.

I: So, it should be everyone’s message? Not only the youth?

P: Yes.

I: Okay. So, can you tell me about other just suggest- other suggestions that you have which may encourage the youth to test?

P: Like you should have more people that would go into different places and open like groups-

I: Support groups?

P: Yes, support groups so that you can tell them about what is HIV, and those who are interested would also like take place.

I: Okay, other suggestions that you may have, which will encourage youth? As youth, what would make you have interest on this HIV testing?

P: Now it’s our own generation, we must take care of each other so that- We should work together, actually. We must work together to-

I: What other things? What other things can be interesting to the youth so that they can come to a certain place like maybe when we have placed a tent for HIV testing services, what can we do that can be interesting to the youth so that they can come?

P: Like, latest music cause like as youth, we like dancing. So, if we can hear certain music that we like, we will come and then when I get there, I will get an experience.

I Okay, so music will make an impact?

P: Yes.

I: Okay, are there any final thoughts you have about youth, HIV testing services or incentives?

P: No.

I: You don’t want to add maybe any incentives that we should give? Okay, let’s talk about you, personally, what kind of incentive would you value? What is it that we can give you so that you can test?

P: From my side?

I: Yes. You, in your personal.

P: Like, from my side, I think you can give me anything because you’re not forcing me, it depends on me if am I interested or not.

I: So, you won’t come for HIV testing services just for incentives?

P: No.

I: Okay. Thank you very much. Now we’ve come to the end of the discussion and for any information, you can call the number on the form that I’ve shown you. If maybe you want information, you can call our office, or you can call our manager’s cell-phone number from 8 o’clock in the morning, until 5.

End time: 15:12
