# Supplementary material for: Exploring perceptions of low risk behaviour and drivers to test for HIV among South African youth
Source: PLoS One. 2021 Jan 22;16(1):e0245542. doi: 10.1371/journal.pone.0245542 (PMC7822253; doi:10.1371/journal.pone.0245542)
Supplement: S1 File — (ZIP) [file pone.0245542.s001.zip › S1_File_Anonymised Transcripts/YA01-020-KK Transcription_QC2_TM.docx]

Full Participant ID: YA01-020-KK

Participant Type: Male, Age 20

Location: Winnie Mandela Clinic

Date: 31 August 2018

Start time:

Primary interview language: English

Name of Facilitator/Interviewer: Wellington Maruma

Name of Note Taker:

Name of Transcriber: Reba

Length of recording: 32:59

Label Key

I = Interviewer

P = Participant

N = Notetaker

{ } = Indicates that details were changed or pseudonyms were used to anonymise data

xxx = words were omitted to anonymise data

- = breaking into a sentence by the next speaker

… = pause or drawn out words

[ ] = indicates noise made, e.g. [laugh], [sigh], [pause]

[inaudible segment] = Unclear section of the recording

?Mulenga Clinic?, ?P3? = questionable text or doubt as to what was said or who said it

I: Uhm, do you allow me to record this interview?

P: Yes.

I: Okay so we can get started. Thank you.

P: Okay.

I: So can you describe to me what your thoughts are on HIV? What do you think HIV is?

P: Uh, HIV…from my knowledge. It’s a, it’s a disease which…which, uh, {which} it goes to people, it goes through people, it goes to people most of the time sexually.

I: Mhm…

P: But there are other things {which} we are unaware of them {which} can also be of transmission {of} HIV/AIDS.

I: Do you know of those, those things {you’re mentioning}?

P: Cuts, things {that are the same as cuts}.

I: Mhm…

P: Mhm. Yah, that’s the slightest example {that I have}.

I: Mhm. So is that the only way you know, uh…or ways you know, like ways you know a person can get infected with HIV?

P: Mhm.

I: Sexually and through cuts…or do you know of any other ways? Huh?

P: Uh…yah. Uhh…

I: Are those the only ways you know?

P: Yah, that I know. But I know there are more.

I: Mhm.

P: Yah.

I: So what do you think would put a person at risk for…contracting HIV?

P: [exhale] Having unprotected sex and clearing things…such as cuts without protective gloves of some sort.

I: Mhmm…

P: Uhh [pause] uhh, lack of knowledge.

I: How so?

P: Not knowing…how HIV is transmitted between two people.

I: Mhmm…

P: And…not, not being able to understand the root cause of HIV/AIDS.

I: Mhmm… Okay and {you} personally {right}?

P: Yah.

I: Have you ever felt that you were at risk of HIV at some point?

P: Yah.

I: Wanna tell me more about it?

P: Yah. Well there was this girl, {right}? {Where I come from} and she was about, I think by that time she was about twenty-six or twenty-seven, it was 2016. Well basically…from {where I stay} they know her as…can I say the word or…? Uhhh [laughs]…a slut {that’s how they call her}. But {for me} this thing, there was this thing that came over me cause {she’s a person who likes having fun} and then she doesn’t stay {where I stay}. She’s from another place…

I: Mhm…

P: And then she was…sexually active with another friend. Whereby she became interested {in me} and then I took it as it is.

I: Mhm…

P: So at once I had that thought {of no} I might be infected, and it was 2016. And so far {I never took it serious and said I’d go test} or something.

I: Mhm.

P: Because {she’s a person who} I only found out after {that} she does go and test and stuff so {I took it with a black person’s mentality and said that theres nothing wrong}. [laughs]

I: [laughs] Okay, and then like…so {you said} she tests, she tested like a lot?

P: Yah. She usually tests.

I: She tests. And then {you?}

P: {I} have never tested before.

I: You wanna tell me why?

P: Uhhh…like I said people do get away with? Being afraid/fear or it? (04:20) so I’m one of those people. So {I think what if I go there and I find out I am HIV positive} because I’ve been…

I: Mhm…

P: I’ve been doing things even though {I’m in relationship, I’ve been in a relationship for three years} but I’ve been doing things outside my relationship.

I: Mhm… So {what are you afraid of?} Tell me…do you wanna tell me?

P: I think…{just} judging by the things {that I have gone through}, how is it possible that I am not infected? Is it possible {that} I’m infected? Those are my two questions.

I: Mhmm…

P: {It’s what I always ask myself}.

I: Don’t you wanna know though?

P: That’s why {I’m saying that} after this I wann aget tested. Because {I didn’t have that heart of} I’ll go to the clinic {to test} and stuff.

I: Mhmm… So…about, other than {at the clinic}, where else do you {that they test people?}

P: Uhh, hospital…I think.

I: Mhmm… And where else?

P: {XXX} (Name of a non-profit organization), there was this poster {XXX} (Name of a non-profit organization) before, it was {XXX} (Name of a non-profit organization)before and what do they call it? They changed its name…} I don’t recall the name {but} uh, it was closed last year {around our side}, I don’t know if it still operates around South Africa.

I: Mhm…

P: Its just that. {I forgot it’s name}. Yah.

I: Mhm.

P: Mhm. [pause] And it did a lot of things this {XXX} (Name of an organization)and my brother used to work for it…my older brother.

I: mhm.

P: Uhh. Is it…? {Doesn’t the name have…? [pause]}

I: It doesn’t matter.

P: It doesn’t matter? {If I remember it} during the interview, {I’ll tell you}.

I: So, so {at the hospital}, clinic {you spoke about {XXX} (Name of an organization)and stuff right?}

P: Yah, {like {XXX} (Name of a non-profit organization)}…

I: Now where do you think you wanna get tested? {In the streets? People from the streets?}

P: No.

I: Why not?

P: Ah, it wouldn’t be accurate, I, I, I don’t think {they have accuracy of whether I am sick or…uh-uh, from the streets I don’t think I prefer it}. Where it’s done properly.

I: But why {do you think that the ones from the street aren’t} accurate?

P: Cause…I don’t think they have enough resources and stuff. Mhm.

I: Okay. And then {these resources} what do you mean, what type of resources are you referring to?

P: Blood taking resource…such as that, these pricks that you use. {In the streets when they…maybe be, I can’t be sure, it might happen but} sometimes you never know. They can prick you {with something that they have already used with} another person.

I: Mhmm…

P: A different person and you find that that person was actually infected and then {you} were not. But just because that thing {worked on you} and then…you become one.

I: Mhm… Okay. [tongue snaps] So in the case of {youth} right? Age, like your age?

P: Mhmm…

I: Where do think would be the most ideal place for you to get tested for HIV?

P: Clinic.

I: Clinic? Why? Is it because of the accuracy thing?

P: {The nurses. The nurses that you find at the clinic.} But, but in some cases there are {clinics that are, nurses that you find} are rude. Just because you are young…

I: Mhm.

P: But its not, I don’t think its ideal cause they are being rude, they are being caring. Because we as young as we are, we lack knowledge…too much knowledge.

I: Mhm.

P: About understanding HIV, whereas {they} have enough insight. Yeah.

I: Okay. So uhm, do think maybe {when the nurses are rude right?} Do you think that’s something that can be a negative thing {that makes people not come to clinics}?

P: Uhh, for example {where I come from…?}

I: Mhm…

P: {A lot of kids, too much…are afraid of going to the clinic cause of the reaction of the women who are nurses for example, a child is pregnant. She’s afraid of going to the clinic to get tested or to go get help because nurse “this and that”} is going to be rude and stuff {to her}.

I: Mhm…

P: Mhm.

I: Oklay, so…so that is like one of the negative stuff of going to test {at the clinic}?

P: Yes…

I: So what other negative things can you think of?

P: Uhh [sigh], what I’ve heard…?

I: Mhmm…

P: A lot of people {say that there is no privacy, nurses talk too much. You find that when you find that she is annoyed or maybe if she is trying to make an example, she will make an example with someone. Only to find that} there’s someone in the very same clinic {who knows that person that they are talking about}. Yah.

I: Mhmm…

P: Cause that’s what they do most of them.

I: Mhm. Okay so {its} rudeness, lack of privacy…and what else?

P: Yahh… Uh. That is much.

I: You wanna talk about maybe the positives of testing-?

P: The positives?

I: Mhm.

P: Oh, most of the time…every, every, each and every place it’s located at clinic. It’s allocated at clinic {by} government. It wouldn’t mean {that whenever you want to test} that you would have to spend more than R50. That’s, you see…clinic is more accessible than hospital. It’s difficult for goverment {to put a hospiotal where like, in a place that is small}, there’s no access {for a lot of things}.

I: Mhm. Okay. So {you’re saying} because they are closer and more accessible, that’s one of the positives right?

P: Yah.

I: And can you think of any other…positive thing {of testing at a clinic}? Or if any of these things, these other instititutions that you’ve mentioned?

P: Uh {the things that they use} uhhh like, {they have what can I say [laughs], they have} enough resources.

I: Mhm.

P: To do everything…accordingly. Yah.

I: Mhm. Okay so…can we now maybe just talk about incentives? So what do you think, what comes to mind when you think of incentives?

P: I think mobilisation…

I: Mobilisation?

P: Go to schools…going to schools.

I: Mhmm…

P: Going to schools. Not uh, {you see} going to schools {like high schools and secondary schools.} I think {the kids that are there already their heads have a different mindset and} its going to be hard {changing it from where it is and put it} at a better place. So {I} prefer {that, even at primary…not tta you are going to teach them} whats done, {just give them} more insight about what is HIV, how does it get transmitted… {the effects of it, the consequences of it} and stuff…

I: Mhm..

P: {They say eat while its wet in Setswana}, start from…{when you start from a younger age, they grow up with that} insight an knowledge…of whats happening with HIV. I think it would be better.

I: Mhm…

P: And another thing…[tongue snaps] Focus should be more….i know {that} such institutes {like {XXX} (Name of a non-profit organization)and the one I was telling you about?}

I: Mhmm…

P: When they get to…maybe an event, they get, uh, negative attitude from those younger ones. But I think its more accessible because {they like events} way more than they are…if I can say that. So if you can hit it from that part…then, you’ll be more effective.

I: Mhmm…

P: And it is right now.

I: Okay.

P: Uhmm…and through social media. {Things like Whatsapp ususally get things like a long chain message where they discuss something, that is just not important}, you can still {make} sort of information and then {write} it down, {in} Whatsapp its gonna flow. {It will spread} too much.

I: Mhmm…

P: In a short space of time.

I: Mhmmm… Okay so what you’ve given me {is} suggestions that make uhm, youth {to} access HIV testing information, your services…care, the treatment services, right?

P: Yah.

I: But my question was-

P: Incentives?

I: Yah. {What do you think} like, give me your own definition of what incentives are.

P: Something that you get for doing something.

I: Okay. So you think by having events…would…

P: That’s where the term incentives, will be more applicable.

I: So what kind of incentives?

P: Uh, I can still go to {the shirt one, but something that} is being used quite long, you know?

I: Mhm…

P: {Squeeze bottles} have been used and then, incentives {like what?} Uhh…lets say data, internet data. Uhh, uhh…uhh, caps {caps}…

I: Mhmm…

P: Mhmm, uhhh…{bags}, {like a school bag that’s written “{XXX} (Name of a non-profit organization)”} or a name of any institution will be…yah.

I: Mhmm…Okay. So [clears throat] {when you think about, like} okay so lets say {we give you} these things, right? Or {we give the youth these things} to come and get {to come and get tested}, for HIV. What do you think would be the challenges in providing these things?

P: Mhmm… ? Project? [15:06] [inaudible segment]

I: Huh?

P: [pause]…Uhh, supply…yah all those things where {where will they come from?} And then [chewing]…

I: Mhmm…

P: And then how to allocate them…

I: Mhmm. So {how do you think we should allocate them?}

P: Example, you ask questions…when you get to an event then {they give you a} platform. You start, you create games…

I: Mhmmm…

P: {Which will} when you… {things like mind games} when you {answer a question} then you get. Let’s say you get it correctly or you almost get it…and then you are, uh, you are given something in return.

I: Mhm. Okay but does…how does that link to HIV testing? Cause now {its} mind games, maybe? Once another ride thing? [16:10] but then {will people come and test?} After they play those games?

P: Remember that platform {you are given to do what?} It’s a that you’ll use to explain and make people understand…

I: Mhmm…

P: About the importance of testing…the importance of knowing your status.

I: Mhmm…

P: And the importance of uh, what can I say? {They say uh, they say…} what is better than cure? Uh…prevention is better than cure…yah.

I: Mhmm… so do you think that those are the type of messages you’d want printed maybe {on your T-shirts?}

P: Yah.

I: Okay.

P: Or caps…somewhere.

I: Okay. Paint a picture for me, {right?} How would this, uhm…pic- maybe a shirt, how would it look like? Something that would entice {you}…to come and get tested. Is it juts a plain white T-shirt or…?

P: Have a, you have a example? {The} golf shirt {that you are wearing}.

I: Mhmm…

P: Its, its bright...its something that you can wear anytime and go anywhere {with it}. So you can print it at the back, a message concerning HIV/AIDS {at the back, you print it}

I: Mhmm.

P: Yah. I think yah.

I: Okay. And then these bottles {what about them?}

P: {On the bottles?}

I: Mhm.

P: You can still do the same thing. But with different messages, lets say for {the bottles}…use a different message. For the golf shirts, a different message…{on the bags} a different message. {But message that is combines with HIV/AIDS, it teaches}…

I: Mhmm…{give, give me a little example maybe.}

P: Know your status.

I: Mhm…

P: {It is} It’s a short message {that will fit where? On a squeeze bottle}

I: Mhmm…

P: Short and easy, yah.

I: Okay. So do you think that will work for someone who, like you or is afraid of knowing their status? Cause already a lot of people are afraid to know their status so even if you give them something…they’ll still be reluctant.

P: Remember after, that’s where we go back to that plot {that you get when we get to the events}, you change…work on changing the mindset {of people on HIV/AIDS} Cause most of the people {think that HIV/AIDS is a death sentence whereas its not. So I think its gonna work.

I: Mhmm…

P: From my point of view.

I: Mhmm… So {you} mentioned some of the challenges which, which, like for providing these incentives which could be…you said budget, supply, we gonna be funding them. Uh {the allocation of it} right?

P: Yah.

I: So can you maybe just take me through the benefits, of maybe providing these incentives that you’ve mentioned? What benefits do you think there are?

P: Mindset {of} this youth will be change. Even if, evenif it doesn’t mean completely change but they would have a better insight and knowledge of whats HIV/AIDS. How to deal with it, how to get tested and stuff.

I: Mhmm... Okay any other benefits that you can think of?

P: Uhhh, I’m done [laughs]

I: Okay [laughs] its fine. Okay so I’ll take you back a few minutes away, you’ve spoken about uhm, using social media like whatsapp right?

P: Yah…Whatsapp, Facebook, Twitter…

I: Okay. You said Twitter…

P: Uhhh, Instagram. That’s where you can encourage, you can even create short videos…

I: Okay. Take me through that.

P: Short videos, uhh…lets say you explain [tongue snaps], uh you explain…what can I say? {to explain} how knowing your status can be of benefit to you, than not knowing…cause remember {when you don’t know, you’ll sit there not knowing whereas you have it} and at a later stage {whereby} it is full blown. Then that’s when you start going to the clinic and then its going to be harder than it would’ve been if {you had began with it} from the start.

I: Mhmm… So how [clears throat] So uh, {you uses these social medias on your phone, right?}

P: Yah.

I: So how else do you think we can use, phone…to get these uh, maybe information about HIV testing and treatment services to the youth?

P: Mhmm… {we could send} through videos.

I: Mhmm

P: And then [snaps tongue] and we can create short or long messages explaning…{but} for long messages, some are just gonna recors, uh, they just gonna pass it. Short messages…straight to the point messages.

I: Mhmm…

P: Yah.

I: So these messages would be delivered through, mhmm [snaps tongue] through…?

P: Through {that thing}, we can…see that platform from Facebook, thats where they gonna move from Facebook to Whatsapp. Uhh, Twitter…because Facebook now is…its big.

I: Mhm.

P: It has more followers and especially those youth. Because its free…

I: Mhmm… So you think of the, uh, social media platforms that you mentioned…Whatsapp, Facebook, Twitter, Instagram and to think Facebook would be the right…?

P: Be the, the umbrella. Yah, where it should start.

I: Okay,

P: Yah.

I: And then?

P: Mhmm…

I: The next one would be?

P: From Facebook its gonna move towards that, that’s what I know.

I: Mhmm…

P: From whatsapp, its gonna move to Twitter. Cause if you, if you {if you look at it properly}, Facebook and Whatsapp are one in the same thing right now.

I: Mhm. Okay…so what do you think would be the challenges in using these social medias to, to attract {youth}?

P: Challenges {will be most of the youth that don’t have} cellphones.

I: Mhm…

P: Uhm, which is now {in, in, in…in today’s life} its…I can say its kinda rare {to find a child without a phone}, even a five-year-old {you find them holding a phone.} She’s or he’s on Facebook or Whatsapp, not knowing {what they are doing} …

I: Mhmm…

P: Yah.

I: Okay. And so for those that don’t have {phones, right?} Let’s think about them for, for a minute.

P: That’s where mobilising comes.

I: Okay. So {you said} we mobilise through schools…

P: Schools uh, events or…soccer matches and stuff.

I: Okay. Uhm so, so lets…so how would you feel, {right?} If, lets say you were being informed or you have to register for HIV testing maybe through services, like uh, uh, register for HIV testing services maybe using your cellphone? Maybe therewas a, some sort of an app or…?

P: That would be easier. Yah.

I: Okay.

P: Very easy. Because some of the people {look at things like} okay I’m going to the clinic, {that} clinic is placed in a centre of {XXX} (Name of a place). Surely it will be full as hell. So I can’t afford to lose that time {and go stand in a line} and stuff. So {when you book} through the internet, {you know when you get there} you just go, where you have to go. And then you do what you came to do.

I: Mhm… Is it something that would appeal to you, personally?

P: Sorry?

I: Is that something that would make you interested?

P: Yah. I think because {you see}, for example {me?}

I: Mhmm…

P: {The job that I do} I’m a very busy person, when I’m at home, I rest. When I, when I get up…go to work, during the weekends, I go for debriefings around {XXX} (Name of a place) so {I don’t have a lot of time to maybe go to the clinic. When I knock off its late, the closes at around 5 o’ clock}.

I: Mhm.

P: And Saturday I’m not here, {I’m in} {XXX} (Name of a place), doing other stuff.

I: Mhm. And {what do you think the benefits are for using apps, Facebook} what what? What do you think?

P: {They will be} more applicable.

I: For youth?

P: For youth, yes.

I: Okay.

P: {Cause other people are afraid of being seen, its like people will that I went to the clinic.}

I: Yah.

P: {I’m going to test and then they have that mindset of ah, they are going to think that I am sick.} Whereas that’s not an issue, {you} are looking out for yourself, for your own health.

I: Mhm. Okay so let’s say you received uhm, information about HIV testing or anything HIV related right?

P: Yah.

I: Through your phone, how do you think your parents would feel about that or whoever is looking…or your and-, your guardian?

P: {Remember there are}, I can say {there are parents who still hold that old mindset, i’m not sure what they called it, they called it “Phamokate” (Term used by locals to refer to HIV/AIDS) or some sort of, yah. So even them}, I think they still need to be taught…{about} what is HIV/AIDS. And that HIV/AIDS its not a death sentence, it’s a disease which is, which can be managed accordingly. Yah, through treatment {that you get from where? The clinic. Yah

I: Mhm… Okay but {you} personally, do you think your parents would be happy or…they wot be happy about you receiving-?

P: -They will be happy because they know {that} okay, this one is looking out for his health.

I: Mhm.

P: We don’t have to worry {about him} even though we know {he is always out at night, he is always will girls} she-, he knows {that}, whats gonna happen if {he doesn’y do this} and whats gonna lead to.

I: Mhm. Okay and why do you think {other parents}, they don’t want their kids receiving HIV information, you did mention {that} some of them, {they want to be taught more about HIV} …

P: Mhm.

I: And still have that mindset of, HIV {is} “Phamokate” (Term used by locals to refer to HIV/AIDS)…

P: Yah…

I: It’s a deadly disease or whatever, so why do you think some parents would not feel good that, would not be alright that their kids are receiving…?

P: Cause {they think that, that thing…where dies it lead them?} To being sex slaves, to being more committed to sex and being committed to boys. Whereas {that’s not true}. {Me} I think {with my head, the more you block a child} to go out and explore things is the more {you kill them.} Cause by the time {they go out} you’ll know whats gonna happen. {Where you blocked for them, lets say you lock them up for five years, they go out with a chance and sneak out} then {they come back with that thing, one day} so that’s how I take it.

I: Mhmm… Okay thanks. But you’ve mentione quite a lot of uhm, suggestions that would like encourage youth to come and get tested for HIV, one of them being uhm, mobilisation, need to mobilise…-

P: -Go to the youth.

I: Go to the youth, have events uh, where the youth hangout.

P: Mhm.

I: Maybe through social media and provide incentives such as shirts, bottles, data…caps and all those kinda things. Do you have any more suggestions that you wanna add to that list?

P: Mhmm… Uh..uh, uh…Suggestions?

I: Huh?

P: Any suggstions that I had?

I: Yeah.

P: To the list?

I: Yeah.

P: Lets see…. [pause] Uhm…{the, the nurses from the clinic} because most of the young, the youth {are afraid of them.} I think {they} should be taught even though, even though I know that they already been taught how to handle such situations but {it shouldn’t be that when a child comes to test} and then {they become, what I can I say, they become rude towards them} just because {he/she couldn’t follow} the rules and protocols to avoid being infected. They should console, comfort her {and show them that, no} this is not the end of it. From here we gonna work {like this and this} until {you become this way}….

I: Mhm. Okay so we almost at the end of our interview, do you have any final thoguhts that you have maybe {about the youth}…HIV? Testing servives, any final thoguhts? Anything that you wanna add over and above what you’ve said?

I: I think {our government} should me, should be like… {it should be more} participating.

I: Mhm.

P: Like {XXX} (Name of a non-profit organization), I understand it’s not a, it’s a non-govermental organisation {right?}

I: Mhm.

P: Goverment should put in {a hand}. Than {what is is doing} now. Because they are, I can say they are some places that I know…{whereby} they go more than, lets say they go more than 30km to access clinic. Some of the places that I know from {the North West side} especially {XXX} (Name of a province)and {XXX} (Name of a province), {XXX} (Name of a province)…

I: Mhm.

P: Mhm. So I think government should be more participating than its already been.

I: Mhm. So {the issue of} accessing the clinic, do you think {its also} an issue that is like, the reason why a lot of people are not coming to get tested {too}?

P: {If you look at} lets say maybe you to the internet and research places such as {XXX} (Name of a province), {XXX} (Name of a province), {XXX} (Name of a province)…you’d find that uh, HIV mhmm, what do they say…HIV…?

I: Prevalence?

P: Prevalence. Its higher than {the, the} places {such as} {XXX} (Name of a province)and then {XXX} (Name of a province), not {XXX} (Name of a province) – {XXX} (Name of a province){and…} those that have attention {of government such as {XXX} (Name of a province)…}

I: Mhm.

P: {In} {XXX} (Name of a province), I can say youth {of {XXX} (Name of a province) have access to everything} but they are not using it the way they should use it. So access, access {that the youth in {XXX} (Name of a province)has, if it could spread} to our communities and provinces. I think all will be well.

I: Mhm. Maybe you’re already thinking of an extra one you wanna add to the list? [laughs]

P: [laughs] Nah…

I: {That’s all?}

P: Yah.

I: Okay.

P: I think I said what needed to be said.

I: Mhm. Okay. We’ve come to the end of our discussion, uhm, thank you so much for being part of this, participating.

End time: 13:00
