# Supplementary material for: Exploring perceptions of low risk behaviour and drivers to test for HIV among South African youth
Source: PLoS One. 2021 Jan 22;16(1):e0245542. doi: 10.1371/journal.pone.0245542 (PMC7822253; doi:10.1371/journal.pone.0245542)
Supplement: S1 File — (ZIP) [file pone.0245542.s001.zip › S1_File_Anonymised Transcripts/YA02-031-NM_03102018 TRANSLATION by Nokukhanya_QC2_TM.docx]

Full Participant ID: YA02-031-NM

Participant Type: Female, 20 years old

Location: Jerry Moloi Library

Date: 30 October 2018

Start time: 10:47

Primary interview language: IsiZulu

Name of Facilitator/Interviewer: Wellington Maruma

Name of Note Taker:

Name of Transcriber: Nokukhanya Ndinisa

Length of recording: 28:30

Label Key

I = Interviewer

P = Participant

N = Notetaker

{ } = Indicates that details were changed or pseudonyms were used to anonymise data

xxx = words were omitted to anonymise data

- = breaking into a sentence by the next speaker

… = pause or drawn out words

[ ] = indicates noise made, e.g. [laugh], [sigh], [pause]

[inaudible segment] = Unclear section of the recording

?Mulenga Clinic?, ?P3? = questionable text or doubt as to what was said or who said it

I: Thank you so much for being part of this interview. Do you allow me to record this interview?

P: Yes.

I: Okay, thanks. Can you tell me your thoughts about HIV? What do you think HIV is?

P: HIV is a virus that we live with. It’s not a virus that should scare people because you can live with it, drink pills and live forever. You are just like everyone, but you just have HIV.

I: Uhm, do you know how it can infect you?

P: In many ways

I: Can you tell me about them?

P: Maybe unprotected sex and blood if someone is injured.

I: Okay. Do you know about- Can you maybe just tell me about places where you think a person can be at risk of getting HIV?

P: Eish… In many places. People meet in taverns, have sex without thinking because they are both drunk and don’t know each other’s statutes. You can get it that way.

I: Uhm, do you know taverns only-

P: No, not only taverns. There are many places but taverns mostly because they are drunk, and they don’t think.

I: So, you personally, can you tell me, have you ever been in situation where you felt like you were at risk?

P: Hmm.

I: Can you tell me about it?

P: I was also once in this situation where we went to a tavern, left and came back with regrets in the morning because after two weeks I had a lot of thoughts or that I have this thing because I didn’t know this person and he doesn’t know me. So, I became scared.

I: And then what did you do about it?

P: Nothing. I was scared of going to the clinic because if I find that I have it, what am I going to do? I’m scared of many things and even now I have not done anything.

I: Like, what were you scared of so much?

P: What if I find that I have it? If I find that I have it, what am I going to do? I thought that I will live a painful life, knowing that I have this.

I: And then you said you feared knowing your status, right?

P: I’m scared.

I: So, there- Do you think that there’s anything that we can do to make sure that you’re not as fearful to know your status, maybe?

P: Like if I can find someone to sit down and talk to me, if I find that I have this disease cause on my own, there is no one I can talk to if I have that I have it. Everyone I stay with at home would judge me.

I: Why do you think that they would judge you if have HIV?

P: I normally see with our relatives. If there was some event and my family just them, when we come back, they will talk about how this is finishing them. So, I think they will say the same things about me.

I: I get you. So, what do you think can be done about this whole thing of judgements?

P: If we find people who are supportive.

I: How?

P: People that you can talk to without judging you. They would encourage you in many things and that you will still live, just like everybody.

I: And then here at {XXX} (Name of a clinic)or {XXX} (Name of a clinic), right?

P: Hmm.

I: Do you know of any places where they test?

P: There are tents where they test everywhere on the streets.

I: And then why haven’t you gone for those? What’s wrong or what’s right about them?

P: They are right. The tents are right.

I: What’s right about them?

P: Because my sister- I once went there with my sister, she was going to test. They tested her and wrote her name. They found that she has HIV negative. So, that’s why I’m saying they are right.

I: And then what about testing at the clinic? For you, do you think that you would go?

P: To the clinic?

I: Hmm…

P: I can go to the clinic, it’s fine but no-

I: [laugh] Tell me

P: Those nurses talk a lot.

I: What are they saying?

P: They complain a lot. You are so you, why are you testing? What make you think about testing? You are rushing for adulthood things! Things like that.

I: And then what do you think can be done about the people from clinic, the sisters who are judgemental?

P: These sisters must just encourage us to test. So, even if you find that you’re HIV positive, they must be alright, they shouldn’t tell you that you are already positive, at a young age. They must tell you that you be right, and other things.

I: So, your clinic experience, has it always been like that? Not only for HIV, maybe for any other service.

P: No, other services are right.

I: They’re right?

P: Uhm, or maybe it depends on the sisters.

I: Hmm…

P: I think that it depends on the sisters. Some are just rude, others are right.

I: And, do you think are they only like that to young or everyone else basically?

P: Everyone.

I: Everyone?

P: Yeah.

I: Okay. So, those are like the negative things of-

P: Clinic?

I: Yeah. So, what do you think- are there any positive things of testing at the clinic?

P: Otherwise it’s right, you know.

I: Can you tell me more about-

P: You can test and find that maybe you’re positive, maybe they’ll tell you about how long so that you can get pills and be fine.

I: Hmm… Okay. So… So… So, the incentives- What would make you to go and test now that you’ve never tested?

P: That can make to go and test?

I: Hmm… Is there anything that would encourage you?

P: [silence]

I: Is there anything that would encourage you? Cause you said that you’re scared of knowing your status, right?

P: Hmm…

I: Is there anything that you can do that would make you to want to test?

P: If they say after testing, there will be people who will talk to you and advise you about what to do next and what will happen, it will be better. If I tested negative, they would tell me when to come back and how I should conduct myself. If I find that I’m positive, they would tell me that it’s not the end of the world, I can still live like everyone else even if my blood has, you see? If there are these people at the clinic, you won’t think that if you find that you are positive, the nurses will say and too young and positive because I like things

I: So, you think the clinics are not that positive to talk about this?

P: They are not.

I: They are not?

P: Hmm…

I: Okay. And- So, when you think of the word incentive, what do you think?

P: [Pause] The word incentive?

I: Yeah. In your own words, what do you think about it?

P: That after testing they will say that you will get something like caps, t-shirts or keyholders, and things like that.

I: And then you said caps, t-shirts and keyholders?

P: Hmm.

I: So, what else would you like to get maybe after testing as an incentive?

P: [Silence]

I: Huh?

P: I can’t think of anything.

I: Nothing? So, you’re saying if I were to give you a t-shirt and a cap, you would come and get tested?

P: Hmm.

I: [Laugh] Would that work though?

P: Huh-uh.

I: What would?

P: Many things.

I: I want those many things. Anything in your mind?

P: If they say after testing you get maybe food, cold drinks, money, things like that, maybe free phone. I think many people would go but I would also go if they give us money or food after testing.

I: What kind of food?

P: Cooked food that they would dish out for us to eat.

I: So, cooked food?

P: Yeah.

I: Okay. So, like and then let’s say they give you that food and all these other things that you’ve mentioned. Like how often would you wanna get them? Do you wanna get them like once or every time you test?

P: Every time I test.

I: And then how often would that be?

P: After three months.

I: And you think that would encourage you to come and get tested?

P: Hmm... Me and other people.

I: Why do you think like food, money?

P: Because when we pass by the testing tents, many people always say ah we will test and get nothing in return. We can’t just test for nothing.

I: So, you think people would come if we give them something?

P: Hmm… Cause when we pass by, we normally say we won’t get anything. Then we just pass.

I: So, do you think that’s maybe the reason why people don’t test?

P: They think that you will test but get nothing in return. So, you will just live in pain forever, after finding that you’re positive. Others say I’ll see the day I get slimmer or the day I feel sick, then I’ll go. For now, I will not get anything in return.

I: And what do you think of events? Maybe we have events here at {XXX} (Name of a place), where we have like someone famous come, and talk about HIV. Do you think that it’s something that can also help?

P: That can also help.

I: How?

P: This person will arrive and speak to encourage people to test, and other things.

I: What kind of a person? Give me an example. Who would you want?

P: Actors from like {XXX} (Name of a local TV show).

I: And then like what would that person say?

P: They must just come and encourage people to know themselves, things like that.

I: And then do you think- So you said, uhm, if maybe we didn’t give people these incentives like t-shirts, keyholders, people wouldn’t come, right?

P: Hmm.

I: Those are like the negative things about maybe the challenges about providing incentives. What other challenges do you think there are, in giving people these to expect them to come and get tested?

P: [Pause] As you’ve said that maybe if a celebrity that people only see on TV can come. So, if they say this person will be in that place, people would go. After that person has spoken, there would be tents where people can go and test.

I: Uhm… Okay. So, other benefits of giving people incentives? What do you think they are? Cause you said that if we give people, we’ll have a lot of people coming. What other benefits can you think of?

P: [Silence] I can’t think of any.

I: Nothing?

P: Hmm…

I: So, uhm… You said you have a phone, right?

P: Hmm…

I: I see here? P? How do you think we can use them to maybe get information about HIV out there or how do you get information about HIV?

P: I get it from pamphlets at the clinic. I get them there and read.

I: So, how do you think we can use phones?

P: Facebook. There can be a page where such things are posted, cause many people stay on Facebook. So, maybe there can be a page where people talk about it, where people share their opinions.

I: Like what types of things will be discussed there? Can you give me an example of a topic that you wanna discuss on that page?

P: We can discuss reasons why people are scare of testing and things that can make them test.

I: And then for you? Since you haven’t tested, how would you feel if [clearing throat] we use this Facebook page? So, let’s say you get an invite that says do you wanna get tested and maybe you must click on the link and then it shows you the nearest clinic. Would that be something that you would be interested in?

P: Hmm…

I: Why?

P: Ey, Testing? I can test but not here at {XXX} (Name of a clinic). Never!

I: Okay, tell me why?

P: Those nurses here… the sisters just… No! They talk a lot.

I: So, where would you test?

P: In any clinic but not here at {XXX} (Name of a clinic).

I: But what if it’s the same?

P: If they’re the same, it will be a problem.

I: So, why- Is it only just because they talk a lot or there’s something else?

P: It’s that they talk a lot and being scared.

I: And being scared, right?

P: Hmm… Many people are scared that yoh- If I test and find that I’m positive. Many people are scared.

I: And, remind me again, how do you think we can get over that fear of testing?

P: If we get support from people. If they say that there’s a room with people that can give you advices after testing. Maybe if you test negative, they also advise you, you see?

I: Hmm…

P: If you test positive, they must advise you. Not that after testing nurses ask you why makes you test at a young age because that’s what they ask. They ask what makes you test, what do you know at such a young age.

I: So, back to the phones subject. Talking about Facebook, maybe we create a page, is there any other social media platform that you think we can use, cause you’ve mentioned Facebook as a platform.

P: And WhatsApp but WhatsApp- you can also create a group chat, if you have people’s contacts and just chat, ask if they have tested and their experience, what happened and what caused them to test.

I: So, you’re saying this WhatsApp group would be for you and your friends or?

P: Me and my friends. Maybe my friends can add other friends and they would also add others and so-on.

I: But you said at the beginning of the interview that people talk, like maybe your family. So, what if you added someone who knows your family. Don’t you think they will talk?

P: They will talk.

I: So, how do you think we can- Do you think WhatsApp is the best?

P: Huh-uh, WhatsApp. That’s why I mentioned Facebook.

I: So, you think Facebook is better that WhatsApp?

P: Hmm…

I: And then any other social media platforms, maybe?

P: [Silence]

I: Facebook and WhatsApp only?

P: ?P3?

I: So, you think phones can only be used on Facebook and WhatsApp to get information out there?

P: Hmm…

I: What do you think about being contacted directly? Like people from the clinic calling you? Maybe to remind you that you have to come and test or something.

P: People will say hah, why is this person following me and asking me to test? Even myself, I’d ask why is this person on my case.

I: So, you wouldn’t want that?

P: Hmm…

I: So, what do you think challenges of using social media platforms are?

P: [Pause] Challenges?

I: Hmm…

P: [Silence]

I: Take your time.

P: [Silence]

I: Huh?

P: I can’t think of any.

I: Okay. So, let me say for example, I don’t have a phone and then there’s these WhatsApp groups that are going around and they are talking about HIV testing information, treatment and all these type of things. That’s a challenge already because I don’t have a phone. Which means I won’t get this information, right?

P: Hmm…

I: So, what other challenges do you think there are?

P: [Pause] I can’t think of any.

I: And then benefits of using social media?

P: Because many people can join that page on Facebook. When they are just chilling, they can just log in to see what’s happening.

I: But yourself, do you think that it is important for people to know their status?

P: Hmm…

I: Why do you think so?

P: To know themselves because others test only when they are sick. So, it’s right to go when you are still well so that if you’re positive, you can get pills, and be alright cause others drink alcohol and others stop taking pills when they feel better and start regaining their body shapes.

I: So, you’ve mentioned something interesting, that your parents or someone that you live with, would not really appreciate it if maybe you were to come back home, HIV positive. Why do you think that is?

P: I don’t know.

I: You don’t know? Okay. So, if you were to receive a message, let’s say on Facebook, and you’re reading information about HIV and stuff. What do you think your parents would feel about that?

P: They would wonder and ask me what is happening. Then maybe I’d tell them that I want to know information. They would ask if I just want to know. Then they would think a lot of things.

I: And how do you think can encourage them to support you, if you wanna read maybe about HIV on Facebook?

P: [Silence]

I: If they find that you have a page about HIV on Facebook or group or something similar.

P: [Pause] I don’t know.

I: But why do you think parents don’t want their children to know information about HIV?

P: They… I don’t think that they don’t want them to know, you see? I am saying that if I test and find that I am positive. When I come back home and tell them that I went to a clinic and found that I’m HIV positive. They would say a lot of things

I: Like what?

P: They would say a lot of things. Like I said, if we went to a family event or something, when we come back they always comment about someone who is getting finished by this virus.

I: Okay. And then- So, maybe you’ve mentioned like maybe one of the suggestions that you’ve mentioned was maybe to get actors, maybe from {XXX} (Name of a local TV show) to come to an event or something, to speak to you guys about HIV and maybe offer food, which is cooked, right?

P: Hmm…

I: Maybe keyholders, caps, t-shirts, money, you said free phones, right? Is there any suggestion you’d like to give? Maybe what we can do to make people to come and test at the clinic.

P: Besides bringing a celebrity?

I: Anything else you’re thinking?

P: [Silence]

I: Huh?

P: [Silence]

I: Cause you did mention quite a few suggestions. I just wanna know if you have maybe others.

P: I don’t have any.

I: So, you think if we just give people food and all these things, keyholders, caps, t-shirts and then actors come. Do you think they’ll come?

P: Hmm… I think so. Many can come. As I’ve said that some ask what they will get in return because they can just test without getting anything.

I: So, you’re saying that people want to get something in return if they go somewhere, maybe if you wanna get tested?

P: Hmm.

I: So, you’re saying that caps and t-shirts are some things that would work specifically for you or you think that others would like something else?

P: Since others say you don’t get anything in return, maybe if someone says let’s go and get caps or keyholders, others would go and maybe others won’t. They would say you are just testing, what are you getting in return? I’d go the day I see that I’m really sick.

I: Okay. And do you have any final thoughts about the youth, HIV testing and these incentives? How do you think we can- for example, you said that t-shirts, caps and all these things- how do you think we should- you said after every three months, we must hand them out, right?

P: Hmm…

I: Do you think we should give out everything at once? Or what’s the most important thing for you? Like what is the number one thing for you?

P: For me, it would be caps and food.

I: And then the next thing?

P: For me? It’s just the two.

I: Would they work?

P: Hmm.

I: But already there are people who are giving out caps in the streets.

P: Huh-uh. There aren’t.

I: There aren’t?

P: They are not there. [Laugh] or maybe I have not seen them.

I: Yeah. [pause] So, the most important thing for you is food and caps?

P: Hmm.

I: And then, any final thoughts? Any final suggestions on this? Anything that you wanna add maybe?

P: Huh-uh.

I: So, we’re almost at the end of our interview and… [background noise] Thank you so much for being part of this interview. Thanks.

End time: 11:16
