# Supplementary material for: Exploring perceptions of low risk behaviour and drivers to test for HIV among South African youth
Source: PLoS One. 2021 Jan 22;16(1):e0245542. doi: 10.1371/journal.pone.0245542 (PMC7822253; doi:10.1371/journal.pone.0245542)
Supplement: S1 File — (ZIP) [file pone.0245542.s001.zip › S1_File_Anonymised Transcripts/YA02-032-GN (Female18-24 years) Translation_QC2_TM.docx]

Full Participant ID: YA02-032-GN

Participant Type: In-depth Interview

Location: Jerry Moloi Library

Date: 3 October 2018

Start time:

Primary interview language: English and IsiZulu

Name of Facilitator/Interviewer: Wellington Maruma

Name of Note Taker:

Name of Transcriber: Ornate Masuku

Length of recording: 36:00

Label Key

I = Interviewer

P = Participant

N = Notetaker

{ } = Indicates that details were changed or pseudonyms were used to anonymise data

xxx = words were omitted to anonymise data

- = breaking into a sentence by the next speaker

… = pause or drawn out words

[ ] = indicates noise made, e.g. [laugh], [sigh], [pause]

[inaudible segment] = Unclear section of the recording

?Mulenga Clinic?, ?P3? = questionable text or doubt as to what was said or who said it

I: Qualitative interview the date is the 3^rd^ of October 2018, the participant is aged 19 female the participant PID is YA02-032-GN. The location is {XXX} (Name of interview location)and the primary language for the interview will be English and IsiZulu. Ummh thank you so much for being part of this interview, do you allow me to record this interview?

P: Yes I do

I: Ok thank you. Can you maybe just tell me what you think HIV is? What are your thoughts on HIV?

P: Ummh, HIV is part of when you go to test, but me I don’t go to test yea

I: Ummh

P: HIV is about a disease, so someone having the disease and HIV is part of like when you have HIV you are a person, if you have HIV don’t be a person who does not have life, you do have life. HIV ummh you are still a person, HIV comes as a substance of having it yea ok.

I: [sneezing] Excuse me, so you mentioned that you don’t test for HIV right.

P: Yes

I: Can you tell me maybe what your reasons are?

P: I have never tested for HIV because I have never encountered danger like to go to test. I have never had this thing to go and test.

I: Ummh, like what?

P: Like changes to my body like maybe sores around my body yea

I: Ummh

P: Yea I am still the person that I am.

I: Ummh, so like what do you mean when you say changes to the body?

P: Like to be not the person that you usually are, like maybe see changes in you somewhere some how

I: Ummh

P: Yea

I: So you say you have never been in danger right?

P: Yea

I: So that danger can you explain to me what that danger is?

P: Like danger?

I: Ummh

P: I have never slept without using I am always using protection

I: Ummh

P: Yea all the time yea

I: Ok so you said you have never slept without like had unprotected sex right?

P: Yes

I: So how else do you think a person can get HIV?

P: A person can get HIV by not using the condom, always sleeping without protection condom. Always you must always use condom if you do sexual sex, always be careful when someone has blood or is hurt yea.

I: Ummh

P: You must use gloves when you have touching something someone you must use gloves, you must always check that you use everyday protection. Ummh and what else? Yea that’s it.

I: Ummh, so you have you ever been in a situation where you felt like you were exposed to HIV?

P: No

I: Never?

P: Never

I: Ok tell me more about that

P: Ok isn’t as people when we go testing, I think that a lot of people would come to test if we would do a thing like after testing we will give you a voucher, like give them cell phone. Like have groups on social media in those groups like as people we have phones but some are using the older one. If you give someone something after testing like cell phones so that we can socialise on social media and be able to create a group. After we create the group it must be used as a platform to encourage each other after someone has, if someone has tested and is negative they go to that group if you are positive another group. In the negative group we encourage each other as negative people on how we move forward like how we can prevent ourselves from getting this virus, and those in the positive group would be encouraged to go to the clinic to take their treatment, and eating good food that has vitamins, those that are positive will be given a piece of land to start a garden so that they eat fresh food, no alcohol, no sleeping without condom they always use the protection those that have HIV. Then those that are negative they must go to clinic every three months and check. We must also be given a garden where we can get food and we must go to the clinic and help those who are HIV positive.

I: Ummh

P: Yea

I: So you mentioned that you would get tested if maybe they give you a voucher

P: Voucher or something

I: Ummh I cell phone ummh you mentioned cell phone, food

P: Yea so that we can attract people

I: Oh ok

P: Like they can come and get tested

I: Ummh, so do you think that if we don’t give these things they won’t come?

P: No like they won’t come

I: Why do you think so?

P: Like in the past they used to give them caps

I: Ummh

P: And t-shirts written {XXX} (Name of a non-profit organization), so they saw giving these as an important thing like giving this i.e. caps and so on, so if we give them cell phones and vouchers. Aren’t cell phones we can use them for social media and have a group that we will use so that we are in touch and communicate? On that social media we talk to those that are positive like how we can help each other and those that are negative how do we help each other and we say we can help each other in such a way

I: Ummh so

P: T-shirts people can buy for themselves because they are cheaper caps they can buy for themselves but cell phones they won’t

I: So you are saying they don’t want t-shirt and caps?

P: Yea they are not wanted in my own opinion

I: Ummh, so you think vouchers, so these vouchers will be from how much and where would you spend the voucher money?

P: It can be a voucher from R350 upward

I: R350?

P: Yea R350 to upwards

I: Ok and then what would you buy using that voucher?

P: Ok, in those vouchers isn’t a phone a cell phone maybe the price starts from R400 yea a cell phone so that people are able to go on whatsapp and others have twitter and face book since we will create groups in these platforms. Also on that voucher will be accompanied with t-shirts by size 30 and we will look for a suitable name like the same way when you come for this interview.

I: Ummh

P: Yea on that voucher you also get what you call it these what you call these written our unique name like the what is the name of the company the {XXX} (Name of a non-profit organization)so we print those and caps then also get shoes.

I: Ummh

P: Yea shoes

I: So you can use the voucher to buy shoes?

P: Shoes, cap and t-shirts.

I: Ummh, but I thought you said t-shirts and caps aren’t wanted

P: Like Aaai isn’t their ones where written {XXX} (Name of a non-profit organization)but we can come up with our own name and boast with them

I: Oh ok! So you come up with your own name as friends or what?

P: By our company

I: You’re Company?

P: Yea

I: Ok

P: Like your company they can write it here

I: Ok, so you are saying if we were to give you t-shirts with our company logo

P: And money

I: Money? How much money

P: These days money rules everything

I: How much?

P: R400 after testing, everyone will come to test since money runs everything these days. Even though I can say everyone will come test even those who are positive will come test because they need the money.

I: Ummh ok so money and everything that you mentioned how often do you think they should get it, give people?

P: Ok money I can say as of now there are people with no money and they don’t work

I: Ummh

P: So this person who does not work will say yho! Let me go and test and see, even if you are positive you can come because there is money there.

I: Ummh

P: So in terms of phones people don’t have cell phone ummh. On top of that there is a t-shirt its printed please looks

I: Ummh

P: Yea

I: So you mentioned in the group ok

P: Ummh

I: You would have to encourage each other about how to eat the right kind of food, garden like maybe plant fresh garden and what not. So do you think giving people will encourage people to come?

P: Yea yes because some don’t have food and are on the treatment and most times before you take your pills one must eat first. So like we can plant vegetables and we then buy them other veggies and fruits always they must have juice 100% they must not drink alcohol like they can fully recover.

I: Ummh, so let’s say we would give you food what kind of food would you want?

P: Ok vegetables

I: Ok

P: Carbohydrates and fruits and fats and yea

I: Ummh, so I am trying just list all the things that you have mentioned just to, so you said Money, vouchers, cell phones, t-shirts and caps, you said shoes as well and then food. So why do you think like even if we give these things why do you think people still don’t want to test for HIV?

P: Ok, if like we test people without giving them something they will not come

I: Why?

P: Because of you are just testing and don’t get something, like people of nowadays work with something how you attract them to do something, you attract someone by saying come and test and I will give something

I: Ummh so don’t you think that’s ignorance?

P: Yea but people always want to benefit from doing something

I: They don’t want to know their status

P: Ummh

I: Why do you think that is though wouldn’t you want to test so that if you are positive you get help?

P: I have never been met with danger to my life

I: Danger to your life how?

P: I have never been raped, always I use protection when I am going to have intercourse

I: Ummh, so you are saying that because you have never been in danger

P: Ummh

I: Like you have never been raped and you have never had unprotected sex, then you feel like you don’t need to go and get tested?

P: Ummmh

I: But then again you also mentioned that you can get HIV through blood

P: Yea but I have never touched someone’s blood when the person was hurt and I had cut.

I: So you will only get tested if you have that danger?

P: Ummh

I: Why?

P: Because as I have said I have never been exposed to danger like find someone hurt and I help them and we exchanged blood and every day I use protection always… Oh! And I have never exchanged blood to blood and always I use protection. And those who are testing like they are helping themselves because of they get help if you tested and found that you are HIV like they give you counselling that when you have HIV you must eat this kind of food and help you cope with your status that it’s not the first time you are not the only one with HIV and you get help at the clinic.

I: Ummh, and around here at {XXX} (Name of a clinic)or {XXX} (Name of a clinic) this whole section is there a place where you would feel more comfortable to go and get tested?

P: Here at {XXX} (Name of a place) no people are too judgemental

I: Ummh

P: When you test near where you stay or fetch treatment ha! The thing is that there are lines for specific things like a line treatment, a line for those bringing kids instead of this specific line they must just have one line for everything. Like there must only be one line so that no one assumes what you are at the clinic for. With having different lines people will already know that you are there for that’s why most people don’t go to clinics for this reason. If I was to test I would test far from where I stay.

I: Because they are judgemental??

P: Ummh yea they are very judgemental

I: And then how do you think we can stop the whole thing of judging?

P: Like?

I: You mentioned in the clinic we should not put

P: Talk ummh

I: So we should not put in different lines but there must be only one line

P: Yea like so that we are all equal

I: Ummh

P: Only when you enter the doctor’s room can you talk to the doctor and say I am here for this and the doctor will understand your situation and not pass judgement. Also at the clinic there are different files for different people like there are green, white, and brown files so I think that we should get files of the same colour. Because different files people already know you are going where

I: Ummh

P: Ummh

I: Ok

P: Yea

I: Ok, so you have mentioned that maybe if we give people vouchers and all these things right

P: Ummh

I: And maybe have people in the clinic in the same queue and then have the same files right

P: Ummh

I: So what other suggestions do you think there are there to encourage someone like you to come to the clinic without feeling judged and everything

P: Ok we can go around the neighbourhood announcing that at {XXX} (Name of a clinic) on this day we are having a meeting there we have a meeting then we talk like ok guys today’s meeting everyone will now get a green file all of us it does not matter what you are suffering from

I: Ummh

P: And everyone will get into the same room without knowing what this doctor or doctor deals with what but people will like always talk.

I: Ummh

P: Ummh

I: So you are saying through community meetings? Then someone will come and tell you that?

P: Ummh

I: This is how things would be going at the clinic, do you think that community meeting could also be an opportunity maybe to have an event to invite the youth to test; do you think that would work?

P: Yea it will work

I: Ok

P: We will get vouchers isn’t we have that meeting and after the meeting we have a meeting as the youth and we are told that today after testing you are given caps like from my company I made a name, company, caps or money. You know that after testing there are a number of options to choose from like one would want money the other cell phone and this and that.

I: Ummh

P: So as the youth this will make us very happy that today after testing we will get money, what do you want, what do you want?

I: Ummh

P: Yea

I: Ok

P: And also have a party

I: A party? Ok

P: An event

I: Event ok

P: Ummh, we plan that on this day as the youth we will have a party and people will be very happy

I: Do you think the youth loves parties?

P: Yho! They love parties too much

I: Ummh

P: If you are to tell them about a party they will be very happy, and say most definitely we will be there

I: And then who will like be addressing you like at the party?

P: Like there will be a person

I: What kind of person?

P: Like someone like you so you come from your company and you tell us, like we can start with something like a talk about living with HIV and giving each other advice like it’s not end of life you must go to clinic and test take the R the ARV’s treatment then you will be ok. Then at 8 o’clock set alarm and we start having fun

I: [giggles]

P: Then if you are negative ok guys always use protection, if someone is hurt having blood to blood try help but only using gloves you can help that person

I: Ummh, so this event

P: Ummh

I: How would you feel let’s say some celebrity comes and then would be the one taking you guys through the information on HIV testing? Do you think that would work?

P: Yea it will work like we will announce that on this day we will call {XXX} (Name of a South Africa musician) and other celebrities like {XXX} (Name of a famous US musician)like people would be excited for that. We can say the party is from 12 o’clock until later everyone will come drinks are there, the entrance is free and everyone will appreciate free entry, drinks, food, toilet everything ummh.

I: Ummh, what kind of food?

P: Food like meat, wors, boerwors and meat like meat and chicken, like it will depend on you if you want pap with red meat or wors or you want chicken

I: Ummh

P: And then for gravy we do Sunday food everyone will appreciate it.

I: Ummh, so ummh these things that you have mentioned vouchers, cell phones and all these things ok we call them incentives.

P: Ummh

I: Ok, so what other like do you think there are challenges with giving people these things?

P: Challenges?

I: Ummh

P: There can be there

I: Can you take me through them?

P: Ok challenges that we could maybe face as the youth, as youth we are faced with challenges that maybe you meet a guy and maybe the guy lets you down when you come to test you can get a voucher and you can use it to console yourself and you know in the coming days there is an event. So as the youth we appreciate if we test and get something in return like I said voucher and maybe start a sport

I: Sports?

P: Yea

I: How do you mean?

P: Like there are people, isn’t they say that if you have HIV you must exercise

I: Ummh

P: Yea, so you can start netball, soccer, rugby, table tennis team. There can be games played with other areas and whoever wins gets a medal. Like the youth like things that will attract them for example when schools close host here at the library poetry sessions, read stories and maybe challenge each other…I forget it like poems, read stories and we see how you read and the reading can be done in different languages like English, Sesotho and Sepedi.

I: Ummh

P: Yea, then we maybe do a modelling competition like “Indomi” (refers to a local competition) like Miss {XXX} (Surname of a democratic president of SA) and we do a contest, yea I think that would work

I: Ummh interesting, so you are saying that if we have events, sports competitions that would be something to encourage the youth to come

P: Ummmh

I: Ok, that’s interesting so any other suggestion that you can think of?

P: Ok as you guys have come today you gave us information as the youth then we also gave you information about how the clinics are. You also explained to us how one must treat themselves as a person

I: Ummh

P: Yea

I: Ok and then ummh let me take you back to like 5 or 7 minutes ago when you mentioned we can maybe use face book as a

P: As a group

I: Ummh as a group, so do you know maybe other ways in which we can reach out to the youth any other social media platforms?

P: Instagram

I: Ummh

P: Twitter, instagram and twitter ummh. Like gather people’s numbers then we create a group on whatsapp ummh

I: Ummh ok and on that group would you, like who would be the moderator you or someone else who would be managing the conversation?

P: I can manage

I: Ok, but then it would have to be someone who is knowledgeable about HIV

P: Yea

I: Ok

P: Isn’t I will learn from others then I will create a group name and include all these people ummh

I: Ok, and then that group will be among you and your friends or anyone?

P: Anyone yea

I: Ok and the twitter how can we use it?

P: We can use twitter isn’t we can get peoples numbers then create a group there too. After creating this group we discuss everything and will include everyone whether negative or positive and not show the status of anyone positive or negative.

I: Mum

P: So that we can communicate properly

I: Ummmh and the same way we used twitter and create groups

I: Ummh, do you know how instagram works?

P: Ummh ummh, I know it here and there but you post something and then people comment on whatever you posted.

I: What kind of things would you post?

P: I can announce that I have an event the event will have a theme our company will help us and we maybe wear t-shirts and pants that are black and black shoes that will be our theme

I: Ummh

P: That way people will comment asking about the time of the event and you reply and you let them know that the entrance is free and you know people love free things so free drinks, there is nothing you buy everything will be on me.

I: Ummh, ok so what are the challenges of like using social media l what do you think they are?

P: Like sometimes it’s not right to use these like twitter

I: Why?

P: Because while we are serious there might be other people who might not be so serious and say the wrong things sometimes a phone is a bad thing sometimes it’s a good thing cause you can easily connect with someone

I: Ummh

P: Sometimes eish it’s not right sometimes it’s right

I: Ok and what are the good things about a phone?

P: Like a phone is helpful as I said we will create a group a group will help make communication and reach easy, we share ideas and the messages reach people quick, when we are about to host an event we post about it.

I: So you mentioned that one can use a phone maybe use social media right?

P: Ummh

I: So what other ways can we use the phone?

P: We can always not use phones but we use laptops

I: Ok how?

P: Like isn’t a laptop you can download things like facebook and still communicate

I: Ummh, but I am saying phone how else do you think we can use them? Is it only social media or there’s others? How about maybe I call you to say come and test do you think that would work?

P: Ummh, yea it will work

I: Like it will work why?

P: Like I can phone you maybe and say come to the library at 9 o’clock and here at the library we give you something after you test you can get a cell phone and you will choose what you want cell phone, t-shirts or money. So everyone will come like you post something and people will send you a call back and you call them.

I: Ummh a call back? Ok that’s interesting, ok and then ummh…So you have mentioned challenges with social media, so let’s say for example let’s say I do have a phone but I don’t have social media how else do you think you can contact me and give me information that’s on social media instagram twitter and whatsapp?

P: Isn’t we will use phones there are phones smart phones and there are ones that are older models those that use the older models will send a call me back and I call them back but if it is you just go to the facebook group.

I: Ummh

P: Isn’t you would have come to test and you have the option of getting a phone and joining the group

I: Ummh so let’s say that we post information everything about HIV on this instagram twitter and whatsapp right, how do you think your parents will feel?

P: Parents will still be ok, as a parent you obviously know your child like their HIV status, if they are negative you preach protection and if you are you positive you can encourage them that go to the clinic or go water the garden and get food

I: Ok, why do you think other parents they don’t feel good when they see their kids go through HIV testing information?

P: Ok often times our parents warn of these things and we never listen they say don’t drink, don’t go out at night and once you contract the disease they start saying I told you not to go out at night and your drinking habits and you did not listen they tell us all these but we never listen

I: Ummh, so how do you think we can get over that like parents like who do not want their children to learn about HIV?

P: Like you know us as the youth

I: Ummh

P: We must have a meeting among ourselves and tell each other to stop going to taverns and going out at night when they reprimand us we must listen a teenager must be a teenager and a parent a parent

I: Ummh so do you think these taverns and going out at night are some of the things that put people at risk of HIV?

P: Yea isn’t like a guy like a guy will go to the tarven if you are a girl you would go to the tavern too, some ladies go to these places with no money and men keep buying you beers as the night ends you go home with the guys to pay for all the beers with sex, most guys are the ones that give woman AIDS cause a person can tell you that they do not want a condom and the girl insists on it some guys tear the condom, you guys continue with intercourse and since you are drunk you do not feel anything. Others when they are coming from these places they are raped or some leave the house to go to the shops at night while there are gangsters and they end up being raped.

I: Ummh

P: Ummh

I: Mmmh, ok

P: Also if you are dating someone your age you understand each other but older people are different and it’s not easy to negotiate condom use with them

I: So do you think sometimes the risk of HIV is dating someone who is older?

P: Ummh older and always being at the tavern

I: Ummh

P: We won’t have alcohol we will have juice and drinks no alcohol

I: Ok interesting, so do you have any other suggestions we are almost at the end of our interview do you have any suggestions? Over and above everything that you mentioned do you have anything you want to say?

P: Like I want to say that as the youth we must take care of ourselves and be an example to the young ones so that they learn from us and respect each other.

I: Ummh, do you have any final thoughts about the youth, incentives those incentives that you mentioned, voucher, cell phones, shoes caps, money, t-shirts and cap is there anything else you want to add maybe?

P: Ummh can add…ummh its fine

I: Its fine?

P: Yea

I: You are happy with the list?

P: Yea I am happy

I: We are actually at the end

P: Then we will add

I: Ok no problem

P: Then we will add if you are in school doing Matric if you have no money we will take you to University like continue with school

I: Sort of like I bursary?

P: Yea I bursary ummh and we will buy food and pay for your accommodation. Those seeking work and have Matric and you don’t feel like University and maybe you want to join {XXX} (Name of a local radio station) ok so we will help with learners and licence.

I: Learners and licence?

P: Yea

I: Ok

P: Ummh

I: So like learners and licence would be for? I guess obviously you have to get it once right?

P: Ummh

I: So let’s say that was the only thing to be offered do you think if we give learner’s do you think they will come back if that is the only thing we give them?

P: Yea they can come

I: Come back?

P: Yea learners is the most important thing

I: Ummh

P: Yea

I: Ok do you have any final thing before we close off?

P: [giggles] Ummmh as youth 15 to 17 years like we host a party for them we organize a jumping castle, swimming pool… Yea

I: Ummh

P: Ummh

I: And then at this party

P: And then we use an open space and host a party that everyone who is bored can come to park don’t go to boys.

I: Ummh

P: Ummh

I: Ummh and then that will be the place that we will be doing HIV testing

P: Yes

I: Do you think they will come?

P: They will come

I: Ummh

P: Yea, since the entrance is free

I: Ummh ok anything else you want to say before we close off?

P: Ummh thank you for coming to {XXX} (Name of an interview setting)

I: Thank you so much for being part of our interview

P: Ok

I: The time is 12:05, Thank you so much

P: Ok

End time: 12:05
