# Supplementary material for: Exploring perceptions of low risk behaviour and drivers to test for HIV among South African youth
Source: PLoS One. 2021 Jan 22;16(1):e0245542. doi: 10.1371/journal.pone.0245542 (PMC7822253; doi:10.1371/journal.pone.0245542)
Supplement: S1 File — (ZIP) [file pone.0245542.s001.zip › S1_File_Anonymised Transcripts/YA02-033-NM FINAL TRANSLATION by Nokukhanya_QC2_TM.docx]

Full Participant ID: YA02-033-NM

Participant Type: Female

Location: Daveyton Main Clinic

Date: 03 October 2018

Start time: 10:55

Primary interview language: English

Name of Facilitator/Interviewer: Bakang Mosime

Name of Note Taker:

Name of Transcriber: Nokukhanya Ndinisa

Length of recording: 37:23

Label Key

I = Interviewer

P = Participant

N = Notetaker

{ } = Indicates that details were changed or pseudonyms were used to anonymise data

xxx = words were omitted to anonymise data

- = breaking into a sentence by the next speaker

… = pause or drawn out words

[ ] = indicates noise made, e.g. [laugh], [sigh], [pause]

[inaudible segment] = Unclear section of the recording

?Mulenga Clinic?, ?P3? = questionable text or doubt as to what was said or who said it

I: Do you allow me to record this conversation?

P: Yes.

I: Okay. Can you please describe your thoughts about HIV? Explain to me your thoughts about HIV. What do you understand by HIV?

P: Like, I don’t really know much about HIV, but uhm [pause] I never tested for HIV, cause I think… I think if I test for HIV it will bring results that I didn’t expect, you see? So, uhm, I don’t think it’s a good idea to test if you don’t understand much about HIV.

I: So, you need more education about HIV?

P: Yes. I think [pause]

I: Okay. Do you maybe know how one can get infected by HIV? Do you know HIV is transmitted from one person to the other?

P: Not really.

I: You don’t know anything?

P: Yes.

I: So, can you tell me what places do you feel like a person is mostly at risk of getting HIV?

P: Uhm… Like you mean [pause]

I: Places? Any places that you think maybe uhm, when a person is there, he or she is more likely to get HIV at?

P: Like there are many places where you can get HIV. For example, you can get HIV from accidents, like touching other’s blood without covering your hands.

I: Other places?

P: Like having sex without protection can get HIV.

I: Okay.

P: Uhm.

I: And what other places do you think a person is more likely to get HIV at?

P: Like partying, eating drugs, uhm…

I: Any places again?

P: I don’t have.

I: You don’t have any? Okay. That’s good. Can you tell me about any situation you felt like that you may have been at risk of getting HIV?

P: Like?

I: Any situation where you felt like maybe this time I’m at risk of having HIV?

P: Like… Sometimes if I’m having sex without protection, I feel that of having HIV.

I: Okay. Is that the only situation where you felt like you were at risk?

P: Yeah

I: Can you tell me about the HIV testing services that take place in your community?

P: Yeah, they have tents, like and clinic.

I: Okay Clinics?

P: Yes.

I: So, at the tents, who are testing HIV?

P: Like… I won’t say but they are not nurses.

I: Okay. Have you ever maybe tried to go and get tested?

P: No, I don’t feel comfortable.

I: You don’t feel comfortable?

P: Yes.

I: What makes you not to feel comfortable?

P: I think it’s better if they use their nurses to test you, you see? Like if- like if you won’t feel comfortable if I know you, then I must test you and know your results, you understand?

I: So, you basically prefer someone who-

P: Yeah.

I: Okay. So, these HIV testing services, where do they normally take place?

P: Anywhere. At schools’ gates-

I: At school’s gates?

P: So, at the school, do they test everybody like any age, or they just choose certain age maybe?

P: They choose.

I: So, from which age?

P: Eighteen.

I: Okay. What about the fifteen to the seventeen? Do they test them?

P: I don’t know.

I: Okay. So, in your opinion, what is positive about the HIV testing services that are available to youth?

P: Please help me understand.

I: What is positive about these HIV testing services? What do you think is the impact on the community?

P: Like it-

I: Help how?

P: It’s helping because they encourage you to go and test instead of standing in lines at the clinic, you see? They help a lot because you don’t have to go to packed clinics, you see?

I: Hmm… So, you think these people with gazebos in the community help to… to reduce crowd from clinic?

P: Eh… Uhm…

I: The people you spoke about in tents-

P: Hmm.

I: Do they assist the government clinics to- for in terms of number?

P: Yeah, I think-

I: Like to reduce crowding in clinics?

P: Hmm, yeah.

I: Okay. So, do- Maybe, think most people prefer HIV testing services in the community than the clinic?

P: Yes. Like our clinic, it’s not- their service is not right, you see? So, it’s better if they put their tents there, you see? At the clinic, they don’t normally- They don’t care, they don’t attend patients immediately. Like many kids died because of that service, lack of service.

I: Service where? At the clinic?

P: Yeah.

I: Okay. So, you gave me the positive aspects about HIV testing services in your community.

P: Hmm.

I: So, what do you think are the negative aspects of the current HIV testing services that are available to youth?

P: Please help me understand.

I: Uhm, what is negative about HIV testing services amongst youth?

P: Uhm…

I: You told me about the positive things. You said they have help and eliminate the crowd from clinics.

P: Hmm.

I: So, what’s negative about it?

P: Eh… On that side, I don’t think it’s a good idea to test on the streets, cause they don’t get counselling after testing. If I test and get that I’m positive, I could do something wrong about myself or I could do something, you see? So, I don’t think it’s a good idea. If it’s- If it continues, like they have to counsel those that people-

I: Who test?

P: Yes, people who test.

I: Okay. So, if maybe we were to incentives to people to maybe get HIV testing services or access treatment... Uhm, [pause] Do you these incentives could encourage people to get tested?

P: Yeah. I think…

I: Do you think they’ll be encouraged to come and get tested?

P: Yes. They- I must say things that-

I: So, what do you understand about the word incentive? What do you understand?

P: Like I understand that what will they get if they if they get tested, right?

I: Hmm.

P: I think that if you produce sanitary towels for them, they will get tested.

I: So, [pause] what types of incentives do you think youth would value to get- to access HIV testing services and treatment?

P: Huh?

I: What kind of incentive do you think youth will value?

P: Like, sanitary pads.

I: Sanitary pads for females?

P: Yeah.

I: Okay. And for sanitary pads, any brand maybe that you think of?

P: Maybe Always.

I: Okay. And what other incentives we can give? Give me other incentives you can think of.

P: Maybe if they come for testing, there should be lunches, maybe give them sandwich and juice for the youth.

I: Okay. Other incentives?

P: Uhm, t-shits are right but they’re not enough.

I: T-shirts?

P: Hmm.

I: Why are they not enough?

P: Uhm, like if- if you call people to come and get tested for t-shirts only, they will be lazy to come but if you tell them about sanitary pads, like some other girls don’t afford to buy those sanitary pads.

I: What other incentive can you think of? You gave me sanitary pads, lunch, sandwich and juice, t-shirts and then what other things can you think of?

P: Uhm… I don’t have any. [Pause] Maybe phones?

I: Cell phones?

P: Yeah.

I: What kind of phones? Smart phones or just phones?

P: Smart phones. We’re talking about the youth, right?

I: [laugh] Okay. So, youth likes smart phones?

P: Yeah.

I: Okay, what else?

P: Airtime.

I: Okay. So, what does youth do with smart phones?

P: Like… I think it’s help them for like they’re schoolwork like they tell us to go to the internet, some of us even our parents, we don’t have smart phone. It’s cost us to go next door and ask for that particular phone in order to get information that I’m needing.

I: So, how do you get access to information on a smart phone?

P: Like through internet.

I: Free internet? Is it free internet?

P: No. We use airtime or data.

I: Okay. So, do you think data can help, if we were to give data to young people, it will help them?

P: Yeah.

I: So, data can fall under incentive?

P: Yeah. Maybe and free Wi-Fi.

I: That’s great. And how often do you think these incentives for HIV services should be provided? How often do you think we should give these things?

P: Like if they come to that particular tent after testing, they get that particular phone or sanitary pads.

I: Only when testing?

P: Yeah.

I: Okay. What could be the challenges of providing these incentives for HIV testing services?

P: Like they- it’s going to have challenges because, uhm, we live in a township. Okay, people will know obviously that if people test for HIV, they get cell phones, they get sanitary pads. I think after getting that particular phone, drug addicts will take that phone from that person. So, I think that after testing, you have my address, you have like my details, I think it’s better if they deliver at home.

I: Okay. What do you think about testing at home? Do you think if we were to come and test you at home would be convenient for you?

P: Yeah, it’s a good idea, cause I will feel more comfortable cause sometimes if I have parents that are not right. Some parents don’t know how to hold their children’s secret, some other parents know how to insult you as her or his child.

I: Okay. So, some parents insult their own children?

P: Yes, and others won’t feel comfortable to test at their homes.

I: So, what kind of insult do parents usually give their child? Do you maybe of any?

P: They will say yeah, you get what you wanted. What were you thinking having sex without condom? Like sometimes they don’t know where did you get that HIV.

I: In that case, do you parents lack education when coming to HIV testing services or just HIV?

P: Like-

I: Do you think parents are not well-educated about HIV?

P: Yeah. I think us as well, we don’t have education about HIV.

I: Okay. So, how do you think we can educate parents and young people about HIV?

P: Like-

I: What do you think we should do as health workers?

P: I think you should open maybe like, like support group. Open something or hire people and enter door-to-door, educate people about HIV, make them understand about HIV, how it works.

I: And for this education program that we must implement, door-to-door campaign or support groups, do you think it should be parents only or parents and young people or should we divide them?

P: Like you can- as the support group, you can divide parents and children as well, but if you if you go door-to-door, you can tell them as a family and make them understand as a family. You don’t say their statuses, right? You educate them about HIV in order to them go to get tested.

I: Don’t you think that can cause conflict maybe when a young person is sitting with parents, teaching them about HIV, maybe giving them an opinion. Don’t you think that the parents will get-

P: Like our parents are not the same. I can’t say yes, I can’t say no, but as my opinion, I think it’s a good idea cause our parents get more educated and understanding about HIV. Like for example, if I go and get tested and find my results being positive, I can tell my parents because I now know that they’re educated about HIV. Like I don’t have that fear no more.

I: So, what is your fear about telling your parents about your HIV status?

P: Yoh! [sigh] I never get tested but they will get frustrated. They won’t treat me the same, you see?

I: As before?

P: As before because they don’t know anything about HIV. They don’t know how to treat someone with HIV. Maybe they will divide my drinking cups, divide my spoon or maybe something. They won’t feel comfortable.

I: So, do your parent speak about HIV, sexual stuff, reproductive?

P: No.

I: They don’t?

P: They don’t know about HIV, they hear people talking about HIV, then they-

I: So, they don’t talk to you at home about HIV?

P: No.

I: Why do you think they don’t talk? Why do you think they’re afraid of talking to you about these things?

P: I don’t think they’re comfortable because they don’t have education about HIV.

I: Oh, that’s great, okay. And what could be the benefits of providing these incentives for HIV testing services?

P: Like, our you will benefit, maybe if they give phones, the youth will benefit freedom. What can I say? It will reduce the work of going to internet cafés and others will benefits sanitary pads, those who don’t have them.

I: So, they will help, right?

P: Yeah. A lot.

I: So, if the parent will ask, where did you get this phone from, what are you going to explain to them?

P: I think if you are planning to do this, you need to educate people about HIV so that I can come with those sanitary pads at home, as I’ve said I was testing for HIV and so-on and so-on.

I: So, you will tell them before you go and get tested. Is that what you are saying?

P: Yes, because they won’t have problem because they now know about HIV, they now know how to protect yourself and you must know your status.

I: Okay. So, can maybe you describe to me your thoughts about being contacted maybe via telephone or social media about HIV testing services?

P: I don’t think it’s a good idea though cause sharing my status on social media is uncomfortable and its unco- what?

I: Confidential?

P: It’s not confidential sharing your status with other people like it’s right to talk to a stranger but not over cell phone cause what will I say?

I: So, here we are talking about HIV services, maybe just informing about the HIV testing services at the corner here

P: Oh, yeah it’s right.

I: Do you think calling you or posting it on social media could be right for you to-

P: Yeah, it’s a good idea but others don’t have cell phones as you know.

I: Can you describe some examples of how you’ve been informed about HIV testing services around?

P: I’ve never been informed about HIV testing services and I know nothing about HIV but what I’m talking is in my understanding about HIV.

I: Okay. How do you feel about being informed and registering for HIV testing services using your cell phone?

P: Like-

I: How would you feel if maybe I was to call you and tell you about the HIV testing services or ask you to register for getting tested for HIV via a cell phone? How would that make you feel?

P: Like… I have to test, I would feel nervous because I never tested before but its right.

I: Can you explain maybe why do you think it’s right?

P: It’s a good idea because some people don’t feel comfortable entering on that particular tent and ask for testing. So, it’s better to contact or send posts. Especially us, youth, going to test at the clinic, other nurses will say you are having sex already! Like they don’t understand that maybe you had an accident and got injured. They don’t ask before, they judge first, without knowing.

I: So, other nurses are judging when they come to get tested, especially young people.

P: Yeah, even if you go there just to prevent for pregnancy. Huh you are having sex at eighteen years? So early! Like they will say such things even if you get teenage pregnancy, they will insult you before they help you.

I: So, what do you think of youth-friendly service clinic? Do you know about them?

P: No.

I: Youth-friendly clinics are those clinics that specialise with young people. They have a section for youth. Do you think maybe if there were to be a clinic for youth-friendly services, youth will go there to get tested, maybe? Or do family-planning?

P: I think that maybe it will help, cause I think they were going to feel comfortable because it’s about youth, you won’t get an adult woman who will insult you before assisting you.

I: So, how could cell phones to inform youth about HIV testing services?

P: Maybe send messages or calling

I: SMS and calling?

P: Hmm.

I: Any platform?

P: Or have their WhatsApp group, where they invite and educate and talk.

I: Any other way in which cell phones can be used?

P: Maybe have Facebook, post flyers on Facebook.

I: So, what message do you think will trigger young people when posted on Facebook? What kind of message? Maybe give me an example of the message, what should we maybe write there?

P: Like uhm… uhm… My opinion?

I: Yes.

P: Like eh, even if I say it in IsiZulu?

I: Yeah.

P: Like we help our youth to test and want them to know their status. Like they will get this and this, after testing. I think that will make them come and test.

I: Do you think that youth reads these promotion messages?

P: Yes, I think- Not everyone is on Facebook, not everyone is on WhatsApp as I said before, but those who have Facebook and WhatsApp, when you scroll and get that poster, you will view and want to know what they are talking about.

I: Can you describe any challenges that youth might face when they are contacted via cell phone for HIV testing services?

P: Uhm, others will be nervous to come and test like it’s better to invite them in personal.

I: Okay. So, other than challenges, what do you think might be the benefits of contacting youth via cell phone?

P: Saving time.

I: Saving time?

P: Hmm.

I: Okay… what challenges do you think there would be if you were to contact youth and informing them about HIV testing services? What challenges using social media.

P: We are not all the same. Maybe others will- if they get that number or call from you guys, others will insult, others will ignore. Like with {XXX} (Name of non-profit organization), many people are playing with that number.

I: Okay.

P: Those are the challenges you’re going to face.

I: And what could be the benefits of using social media to contact youth for HIV testing services?

P: Uhm, you will benefit to save time and resources. Like send SMS or call instead of taking out transport to go to {XXX} (Name of place), maybe by knock-off time, you wouldn’t have covered everyone. So, it’s better to SMS or call.

I: So, do you think youth are more active on phones, social media and?

P: Yes.

I: They spend more time there.

P: Yes.

I: So, in your own experience, how do you think parents or legal guardian will feel about you receiving information about HIV services on your phone?

P: On that, it will help children who are afraid of parents knowing information about HIV because my phone is my privacy. So, I don’t think they will go through my phone and check messages or WhatsApp.

I: Okay. So, by getting these messages, it will help a lot?

P: Hmm.

I: So, can you tell me any other suggestions which you may have that would work to get young people tested for HIV?

P: [Silence]

I: Any suggestions that you have which will encourage youth to test?

P: I suggest that you bring up {XXX} (Name of non-profit organization)again and take it to schools during LO period to educate learners about HIV and encourage them to go and get tested.

I: Okay. Are there any final thoughts that you have about youth, HIV testing services or incentive? Any final thoughts?

P: Uhm, I think it’s a good idea to encourage our youth to go and test because even me, I don’t know my status, but I wish to go and get tested because I don’t know more about HIV, I am scared. It’s a good idea to educate people about HIV, to educate our youth about HIV.

I: So, where do you think this education can take place? Where can we educate people for HIV? Do you think we should go to schools or?

P: Like you can go to schools, you can open your own support groups, you can go to primaries for those fifteen and those fourteen.

I: Okay. Thank you very much. We’ve come to the end of our discussion. If you have any questions, you can call the number on your consent form from 8 to 5 o’clock. And thanks again.

End time: 11:25
