# Supplementary material for: Exploring perceptions of low risk behaviour and drivers to test for HIV among South African youth
Source: PLoS One. 2021 Jan 22;16(1):e0245542. doi: 10.1371/journal.pone.0245542 (PMC7822253; doi:10.1371/journal.pone.0245542)
Supplement: S1 File — (ZIP) [file pone.0245542.s001.zip › S1_File_Anonymised Transcripts/YA03-026-LK Translation_QC2_TM.docx]

Full Participant ID: YA03-026-LK

Participant Type: Male, aged 23

Location: Phillip Moyo, Daveyton

Date: 12 September 2018

Start time: 10:05

Primary interview language:

Name of Facilitator/Interviewer: Willington Maruma

Name of Note Taker:

Name of Transcriber: Nokukhanya Ndinisa

Length of recording: 33:05

Label Key

I = Interviewer

P = Participant

N = Notetaker

{ } = Indicates that details were changed or pseudonyms were used to anonymise data

xxx = words were omitted to anonymise data

- = breaking into a sentence by the next speaker

… = pause or drawn out words

[ ] = indicates noise made, e.g. [laugh], [sigh], [pause]

[inaudible segment] = Unclear section of the recording

?Mulenga Clinic?, ?P3? = questionable text or doubt as to what was said or who said it

I: Thank you so much for being part of this interview. Do you allow me to record this interview?

P: Yes, I do.

I: Okay, thank you so much. Can you tell me what your thoughts are on HIV? What do you think HIV is?

P: Eh, HIV is a disease that kills like it has no cure. They say it has no cure and like it infects in different ways.

I: How?

P: Eh… It happens that your friend or someone gets injured and gets a cut and then suddenly you help them, not knowing their HIV status, you see? You help them without anything, you touch them. Okay, they get help, but their blood enters your pores, all the way. So, like you don’t know and then it happens that you sleep with your partner, you see? Then when you sleep, they also get this thing. It’s something that is continuously.

I: So, you said the cuts and being sexually active, right?

P: Yes.

I: Do you know any other ways that a person can get uhm… this HIV disease? Any other way?

P: Any other way? [pause] Well, eish… ?P3?

I: Hmm…

P: Maybe I can say that- It can happen that let’s say maybe I take something like a condom like that you were using with someone else, and left it there, you get me? Then it happens that I touch it maybe while cleaning. Let’s say we are using the same room, you left it like that and I’m cleaning after you. This thing of HIV, if ever you touch it, the not deal with it early, by taking time to check it, it can spread. It has many symptoms.

I: Okay and then those symptoms, do you know any?

P: Well, I can say that it changes the way you feel. Like let’s say if ever it’s hot, you start feeling cold and hot when it’s cold, then you start sweating. Others cough a lot.

I: Hmm… Okay and then have you ever been in a situation where you felt like you are at risk of contracting HIV?

P: I did once.

I: Okay. Tell me about it.

P: Well, I can say that one of my exes like- I thought it was HIV but it wasn’t, I just had drop, do you get me? Yes, I went there like with a shock like I was scared of going to test at the clinic, but I saw that something is wrong with me. I got there and tested. They said no, you actually have this and I become alright.

I: And then you said you were scared of- to go to the doctor?

P: Yes.

I: Why were you scared of testing?

P: Yoh! Ey… Like it was the first time, you see? I felt like why I am like this. If ever you know the symptoms, when people talk about them and the next thing you see them happening to you. Like I had shock and told myself that I have this thing. So, I was scared of going, like I was scared to find out whether I have this thing or not.

I: So, you were going to test for the Drop, right?

P: Yes.

I: And you were told that it’s not actually HIV?

P: Yes.

I: Okay. You said you went to a doctor. Was it at a hospital or clinic?

P: It was a clinic.

I: Clinic?

P: Yes. {XXX} (Name of a clinic)

I: Oh, {XXX} (Name of a clinic). Okay, do you think the HIV testing services are alright at {XXX} (Name of a clinic)clinic? Or is there anything you’d change? How?

P: Uhm, I don’t know, dude cause they said I must come back after months. So, I wouldn’t say they are 100% sure about their mission.

I: So, since you have not tested for HIV, would you go to {XXX} (Name of a clinic)clinic to test or you’d go to another clinic? Or is there any other place where you’d go for HIV testing services?

P: Yes, I prefer going to the far east not here at the clinic because like when you walk out, you see people that know you. Eish! But even though it remains as a secret but walking out to people that know you- when you’re going to test, you come back with a facial expression, do you get me? If ever you find that you are positive, you won’t be happy or smile, you will walk out feeling sad, you see? Then you find people who know you, looking at you as you walk out from that room. Do you get me? This becomes somehow and leads to other things.

I: So, concerning confidentiality and things like that at the clinic, right?

P: Yes.

I: What do you think can be done about that situation at the clinic? About maybe people talking about other people’s statuses or any other thing. What do you think can be done? Do you have any suggestions?

P: Yoooh! Eish... Suggestions, ok?

I: Hmm…

P: Let me see [clearing throat] Maybe they could shift it cause where it is now, that office is not more secretive, so you get me? Like as they are building, maybe they must have another office space that will be more secretive. Maybe where you will have to turn on a corner or two, then go to the back cause right now you just have to enter and take a left. So, there are doctors for kids, next to the HIV what-what around the corner. And just imagine a queue for kids and walking out traumatized in front of the mothers and staff.

I: And any other thing?

P: Uhm… Just… I can just say that this program of yours makes people aware about their safety. It must be successful. Many people must be made to stay alert. Okay, some people already have the information, but it’s not the full information so that they can do the right things.

I: And then this information, how can we- like how can we get it to someone your age? Like a youth or adult or adolescent? How do you want to receive information about HIV?

P: Yes, you see people my age really like social media. So, if ever such programs have some social media or groups. [vibrating cell phone] If ever there are people who I want to encourage, I can add them then convince them to join the group. Let’s say if ever I take it slow and you trust me, I add you to the group so that you may find encouragement to talk to someone from the organisation about your own point of view. Cause like you won’t talk on the group but if ever you find encouragement within the group, when there is something you want to take off your chest, you can talk to people who work there, maybe you’ll be sharp. Cause yooh… Here at {XXX} (Name of place)mostly girls kill themselves and we don’t know why. So, we must also fight that, besides HIV.

I: Okay. So, you said like social media, right?

P: Yes.

I: Would you like to receive information via social media?

P: Yes.

I: Social media like how? Which platforms? Since there’s Facebook, there’s what-what-

P: Could be WhatsApp cause Facebook is not a good thing.

I: Why isn’t it a good thing?

P: [Laugh] Like Facebook has a lot of things cause you can make a mistake and think you are writing somewhere but you are posting in a wrong place, it could be WhatsApp cause it’s directly to the person.

I: You said WhatsApp could be like a group, right?

P: Yes.

I: And then who would the administrator of that group? Is it you amongst your friends or it’s the clinic?

P: I can say that if ever there are people from the clinic you may like this thing, they can become the admins, you see? And then if ever like me, I want to add two people, I will consult admin that I have so-and-so, and then add them.

I: So, admin would be, maybe like you?

P: Yes.

I: I get it, I get it. And then- so you’ve mentioned social media like WhatsApp and Facebook. What other platforms that you think would work in relaying information about HIV testing?

P: Eeeh… Social media?

I: Hmmm…

P: Eh, there are many social media things, Instagram and others. We must just join social medias, not only Facebook and WhatsApp. Different programs, different admins.

I: Okay, now that we’ve talked about how to use WhatsApp, would you use Instagram to relay the HIV information on Instagram? Like how? What kind of posts would be on Instagram and entice you?

P: Eh… What can I say? Youth likes more attractive things. So, let’s say like here, let’s say there’s a special deal. We host, maybe at the park, there is sound, we play music, give-aways, people come, maybe our group members the t-shirts and encourage people to come. Then there are photos and videos, then they are posted so that people can see that there’s something happening, and if you are interested, then you take a part.

I: Okay. So, you said in these special events there will be entertainment and stuff, you said give-aways –

P: Yes. T-shirts and other things.

I: Are those the type of things that would encourage you to come and get tested? Those kind of give-aways? T-shirts? If they were to be given away, if they say come and get tested, you will get a free t-shirt. Is that something that would encourage you? Those t-shits?

P: Eish, some can be attracted but eish… The problem is that today’s youth likes things that are very high. They are more choosy-

I: What do you think would work for them? Something we can give them.

P: Eish… Maybe [pause] not something that can drug them. What can I say?

I: What about food?

P: Yes. Food. Then offer them drinks, maybe cans.

I: And then this food, what would it look like? What type of food would you like to be served at these special events?

P: I can say that if possible, it can be sausages and rolls, maybe and some slices.

I: So, issue about social media is that there are people who don’t have phones or access to internet. So, how do you think we can reach them with HIV information?

P: Well, that means that like me, let’s say that I have joined, and I must encourage people and not keep information to myself but share it so that others can have information, to join and help others. We must all participate. Let’s say I fetch people who don’t know from the streets or family.

I: So, using these WhatsApp, Facebook and Instagram use data.

P: Sure.

I: Do you think that data could encourage people to come and test? Let’s say if you come and test, we give you 10MB or something like that. Instead of a t-shirt, data.

P: Yes, it could work.

I: Why do you think it could work?

P: Because that’s why since social media attracts people because like let me make an example. {XXX} (Name of cellphone network service provider)} was a first to make WhatsApp data what-what and then people changed, used it for social media and then {XXX} (Name of cellphone network service provider)said you can buy data with R15, that is 1Gigabyte for the whole month and then other people changed to use {XXX} (Name of cellphone network service provider)because it has one what-what. So, they really like something that will make them stay on social media no matter what. They just follow like-

I: Okay. So, t-shirts, food and drinks like cans and data, which one you- which one would encourage you to come and get tested for HIV? If we were to say, we’ll only give you one thing from all of those things.

P: With me, actually it can be the data.

I: Okay, do you have any other ideas that maybe, maybe you have any other ideas about t-shirts, data, maybe you wanna add something more?

P: Something more?

I: Hmm…

P: [sigh]

I: Maybe something that you’re already thinking of?

P: Well. I’ve ran out of options, you know. [pause] But the events can encourage many people, like making posters and put them on social media, paste other on the streets, showing that on this date, this will be happening. Because like when something like in the government is going to happen, we just see it happening, like a tender, without any announcement. Maybe people don’t attend, they think that maybe it’s just something because they don’t have information. So, if ever we have events, we must post, in the streets, hospitals, police station, taverns, shops that sell food. Just posting anywhere because when you post, you encourage others to come and get information.

I: Okay. So, let’s say we give you all these things, the give-aways, drinks, data and do events, what could be the challenges? What do you think are the challenges of giving you guys these things so that you can come and get tested?

P: Challenge?

I: Hmm…

P: Well, eish… [background noise] Some people like those who gave us parents’ excuse, maybe some don’t trust us, they need more effort. I don’t know how to encourage them but I’ll find a way. Besides that one, {XXX} (Name of a friend)because her mother said no in front of me.

I: And them uhm… Like you said challenges would be to encourage people to participate in these things, give-aways? So, if you tell people that at what-what there will be this special event about HIV, they won’t believe you and that’s a challenge as well. Is that what you’re saying or?

P: That won’t be a problem because we’ll be having posters, they can also see.

I: What are the good things of giving these things? Like, what are the benefits?

P: Benefits? I’d say, like if ever we are having an event whereby someone from an organisation and there are people. From these people, I’d say there’s a percentage separating those we can encourage to come. Then there are others but if ever we have the understanding and others also have and understanding. It depends on how a person’s understanding is, but there can be benefits if ever we give them information to go forward, eventually they can come and help moving forward.

I: So, let’s go back to the social media issue, right? So, obviously, to use social media, you need a tablet or a phone or device, and a lot people have phones, you also have a phone, right?

P: Yes.

I: So, how else do you think we can use cell phones to get information out there, despite- I mean besides social media?

P: Without social media?

I: Hmm…

P: I’d say there’s a- create a website whereby we post our things there, like we post where we gonna be, next time like how to find us, like post information. When you have something to post like encouragement or inspiration. Like write there, then you post it on the website, then you post it there to show other people.

I: Give me an example of a post you’d like to see. You said, you’d like to see encouragement or inspiration-

P: Hmm…

I: Give me an example of the inspirational posts you’d like to see on these websites. Something that would encourage you personally.

P: I’d say, these words that my pastor likes to say, like it’s a slogan of our church that failure is not an option. If ever you fail to believe in yourself, no-one will. Something like that because if you fail yourself, no-one could help you go forward.

I: I like that. So, you’ve mentioned that- actually, I did allude to this earlier, maybe some people don’t have cell phones and it could be a challenge in contacting them, right? Can you maybe think of other challenges?

P: If ever someone doesn’t have a phone, like we could meet here. Tell the person the information that since they don’t have a phone, I can take them there, if they are interested, but I know that they can be interested and then they will tell us how to communicate with them, maybe through their parents. Children mostly get the pressure from parents to concentrate more on their books. Do you get me? That’s why they say no phone, but you can communicate with parents because they want what is right for their kids. They won’t have any problems. They will even be happy if like you consult them before the child.

I: So, the parents issue, like you said that sometimes the problem is that the parents don’t want the kids to participate in these kinds of activities because there is no enough information for them, right?

P: Yes.

I: How do you think your parents would feel if you were to receive HIV testing information on your phone?

P: Ah… They won’t think anything. Like my mom, my mom is like a friend of mine. She loved where I work. It’s not the first time being part of an organisation.

I: What do you think the problem is? Maybe some parents don’t want their kids to be receiving information about HIV on their phones?

P: I’d say for today, from what I saw, she was listening to the FM radio there, that there are people meeting other people and then they take them there. Like that wrong information from people. So, what should they do- Like if ever, they don’t trust us?

I: Hmm…

P: We can go, or I can go but she won’t trust me still I’m like her child. But if ever someone like you enters, to assist and talk to her. You see, in a community, if one mother can understand, she can help others to also understand and tell their kids and other mothers to make this big.

I: So, you said that the reason they don’t want their kids to be a part of these things like HIV activations is because sometimes they’re receiving wrong information or don’t believe it or something like that?

P: Yes. Yes!

I: Okay, I get you. Okay, so do you- [cough] So, you’ve mentioned a lot of suggestions, actually that could help improve the HIV testing, right?

P: Yes.

I: One of them is that the rooms in the clinic should be secretive and to have programs that will make people aware, where we give people information, and then you spoke about special events that should be held in the community, uh, where there could be give-aways, issues of uh, and maybe relaying information on social media, right?

P: Hmm…

I: So, are there any suggestion that you can think of that you can maybe improve HIV services maybe amongst young people, over and above what you’ve mentioned?

P: Other organisations.

I: What should other organisations do? Suggestions? I’m looking for suggestions that you can think of that can improve HIV testing services for young people. So, one of them that you’ve mentioned is to have rooms that are secretive because there’s concerns about confidentiality, right?

P: Yes.

I: So, another one, you mentioned that if we could provide- have special events, where we give-aways things like t-shirts, food, drinks. And maybe at these events we take photos and post them on social media platforms like Instagram. Those are some of the suggestions that you came up with.

P: Yes.

I: So, I’m asking if you can come up with other suggestions.

P: Eh… Uhm, I’m out.

I: Okay. So, we’re almost done with our interview, is there anything else you’d like to add about HIV, youth, the give-aways that you mentioned. Do you wanna maybe add?

P: I could add, ok?

I: Hmm…

P: I could add cause like even schools, cause like schools, I could say like {XXX} (Name of a school){here, we could go there with a post and make a thing for an hour or 30 minutes for them and give them information and see. Others could love the information, give it away and come but others- you know how school kids are. But if ever there will be give-aways at school, not outside, they would take the caps and t-shirts there, for their time.

I: So, do you think t-shirts and caps would work at schools?

P: Yes.

I: Or do you think anything else would work maybe?

P: I’m 100% sure about t-shirts and caps at schools-

I: Yeah.

P: But let me think. [pause] Uhmm… maybe offer them a sandwich or something.

I: Okay, so-

P: At schools, we go with our flag there and post and give them information. And test them too! Those interested would come and test. After we talked to them, after we meet and talk, those interested can come.

I: So, do you have any final thoughts before we wrap up our discussion? Do you have anything else you wanna say, maybe if you might have forgotten to say?

P: [laugh] Hah, no it’s fine.

I: Thank you so much for being part of our interview. Thank you so much, we’ve come to the end of our interview.

P: Thank you too.

End time: 10:38
