# Supplementary material for: Exploring perceptions of low risk behaviour and drivers to test for HIV among South African youth
Source: PLoS One. 2021 Jan 22;16(1):e0245542. doi: 10.1371/journal.pone.0245542 (PMC7822253; doi:10.1371/journal.pone.0245542)
Supplement: S1 File — (ZIP) [file pone.0245542.s001.zip › S1_File_Anonymised Transcripts/YA03-027-BN_Translation_QC2_TM.docx]

Full Participant ID: YA03-027-NB

Participant Type: Qualitative Interview

Location: Philip Moyo Clinic

Date: 12 September 2018

Start time: 10:06

Primary interview language: English

Name of Facilitator/Interviewer:

Name of Note Taker:

Name of Transcriber: Lineo Matsela

Length of recording: 28:37

Label Key

I = Interviewer

P = Participant

N = Notetaker

{ } = Indicates that details were changed or pseudonyms were used to anonymise data

xxx = words were omitted to anonymise data

- = breaking into a sentence by the next speaker

… = pause or drawn out words

[ ] = indicates noise made, e.g. [laugh], [sigh], [pause]

[inaudible segment] = Unclear section of the recording

?Mulenga Clinic?, ?P3? = questionable text or doubt as to what was said or who said it

I: Qualitative interview. Date: 12 September 2018. Participant ID: YA03-027-NB. Location: {XXX} (Name of interview setting). Participant type: male. Time started: 10:06. So, do you allow me to record this conversation?

P: Yes.

I: Okay. Thank you. Please describe to me your thoughts about…on HIV. What do you think about HIV?

P: Uhm, I think it’s a disease which is causing people to die. Some of them are suffocating because of it.

I: Okay. Suffocating, how?

P: Like uhm, some of them they don’t take their…their medicine so, some of them they don’t do, thus they have the disease. They’re still sick.

I: Okay.

P: Yah.

I: You mean they’re suffering from the disease?

P: Yes.

I: Okay. How can one become infected by HIV?

P: I think through unsafe sex or rape. Some of them are born with the HIV.

I: Mmhm. Okay. Tell me about the places you think people are most likely at risk of getting HIV.

P: Uhm, it can happens on the streets, even at home or at taverns.

I: Okay. How can it happen at home?

P: Uhm, at home it can happen through unsafe sex, uhm, without knowing. Then uhm…at the streets people can be raped.

I: Okay.

P: Yah.

I: And at the tavern how does it happen?

P: They also can be raped or…[inaudible segment]

I: Mmhm. Okay. Is that the only places you think a person is highly at risk of contracting HIV maybe?

P: Yah. I think so.

I: Okay. Can you tell me about any situation when you felt…where you felt like you may have been at risk of getting HIV?

P: Uhm, where I used uhm a bandage to cover my wound.

I: No, I mean like yourself, personally. Can you tell me about a situation where you felt like you may have been likely to get HIV?

P: Like uhm…[pause]

I: When you felt like this time I thought like I was in a risk of getting HIV.

P: Oh. Ah. I was having unsafe sex. So, the condom…like I was using the condom but it bursted. So, I haven’t got the chance to change it. Yah.

I: Okay.

P: Yah.

[pause]

I: You didn’t get the chance to change it?

P: Yah.

I: Okay. Is that the only situation where you felt you were…

P: Yah.

I: Okay. So, can you tell me about the HIV testing services that are taking place in your area?

P: Uhm, they are taking place, but uhm, I think, uhm…young people are not interested because of the things they are not doing to attract them so that they can come and listen to what they are here to tell them about.

I: Okay. What things…what are those things you are referring to?

P: Like their pamphlets that they are using, they are not colourful enough. And if they’re there in the corners, they just sit there, they don’t move around, maybe alerting people about what they are here about you see. Yah.

I: So, these testing services, where are they taking place exactly in the community?

P: Uhm, they…they stay at the corners; where it is busy.

I: Okay.

P: But still, they don’t move around.

I: Okay. Where are the HIV testing services for youth usually take place?

P: Uhm, {XXX} (Name of a clinic).

I: Mmhm. {XXX} (Name of a clinic)Clinic.

P: Yah.

I: Okay.

P: Uhm, then {XXX} (Name of a local restaurant).

I: Okay. What is {XXX} (Name of a local restaurant)?

P: It’s a restaurant.

I: Okay. So, what’s happening at {XXX} (Name of a local restaurant)? How does it have testing services?

P: They just put a…tent. Then, those things which are written “HIV test”, you see?

I: Mmhm.

P: Yah.

I: Okay. So can you tell me about your experience trying to access or accessing these services?

P: Eish.

I: Have you tried accessing those services since or you haven’t tested?

P: I have! But, eish! [laugh]

I: Yeah?

P: I have but…I…I have been before.

I: You’ve tried to test before? And what happened?

P: Eish! I got there, I was afraid, I thought I have the disease but I don’t have it. So, ah! I’m not sure what I’m going to test.

I: Okay. So you didn’t test?

P: Yah.

I: Okay. So, how was the experience? What made you to withdraw from testing? What made you not to go and get tested on the day?

P: Eish! I saw someone who has this disease, uhm, suffocating in a bad manner…in a bad way.

I: Was suffering?

P: Yes, in the…at the clinic.

I: Oh? It was on that day that you wanted to test?

P: Yah.

I: Okay. So, in your own opinion, what is the positive about current HIV testing services that are available to youth?

P: Like uhm…can you explain more?

I: Okay. What do you think is positive about those people who are placing tents at the corners? Yah. What are the positive aspects of them having those testing station in your corners?

P: I think they want to…uhm, alert people so that they know about their status.

I: Do you think it is, uhm, helpful for youth?

P: Yes, it is but, not always.

I: Why are you saying that?

P: Because there are thing that they are not doing to alert the youth.

I: What kind of things are those?

P: Like uhm, maybe, uhm, having music when they are at the corners, you see? Or moving around, entertaining people but in a…like maybe with their programme that they’re having you see? But they must add some entertainment for the youth.

I: So, you think entertainment can draw youth…

P: Attract. Yes.

I: Okay. To come and get tested?

P: Yes.

I: Okay. And what are the negative aspects? What do you think is negative that they are doing that uhm…on the current testing services? (Isn’t it) we just spoke about the positive ones. Yah.

P: Uhm, the people who don’t know about the programme they think they’re just there to, to to waste their time. So, if they they have to…they must uhm…put more effort to what they’re doing so people can understand exactly what they’re here for.

I: And what will be those efforts? Can you maybe share with me the efforts you’d like to see?

P: Like I said, they are not moving around there, alerting people. They’re just sitting there. If you want to get tested, you go there, if you don’t want you just pass. So if maybe they were handing a…pamphlet around, you see? Sharing the information to people. I think people would be interested.

I: You said something about the pamphlets that they are not colourful. How do you think they should look?

P: Uhm, any colours but they must not be formal colours, you see? Because uhm some young adults don’t like formal colours you see?

I: What are formal colours?

P: Like (like) whites, black.

I: Okay.

P: Yah.

I: Okay, so maybe use different colours like red, yellow?

P: Yah, at least.

I: Do you think youth will read those that are colourful?

P: Yes. I think so.

I: Okay. So, when you say they’re not putting more effort they should go around informing people, do you think when you say informing people do you mean by door-to-door campaign…?

P: No, on the streets.

I: Okay.

P: Like people are passing, they don’t know what is happening, they just see uhm… what do you call those things which are written uhm “HIV”? Those they put aside?

I: The gazebo’s?

P: Yah. They just see those things only. They don’t know what they’re here for, so they must give to some people? Some brief? So that they can come.

I: With a little bit of entertainment on site?

P: Yes.

I: Okay. So, how do you think incentive could be in use to encourage youth to test for HIV and access treatment?

P: [pause] Eish. Like can you give me uhm…

I: How do you think uhm, giving incentive to people uhm should be…could be in use to encourage youth to get tested or access treatment?

P: Uhm those people are here to alert the youth, so if the youth is alert, they’re going to do the testing you see? Yah. I think without alerting them some of them won’t be doing the testing.

I: Okay. So, they should alert them about the incentive that they’re giving out?

P: Yah.

I: Okay. Do you think it will work in order for them to access treatment as well?

P: Yes, I think it will.

I: Okay. What do you understand by the word “incentive”?

P: Uhm… I think are those groups who are here to uhm…to alert people about the certain thing which are happening around.

I: You mean incentives right?

P: Yah.

I: Okay. Maybe we can discuss incentives (right)? Incentives are things that maybe you can give a person to change attitude or maybe do something like when you’re getting tested and you get something.

P: Yah.

I: Okay. So, can you describe the types of incentives that youth uhm, value, which could encourage them to test for HIV or access treatment?

P: Uhm, I think uhm colourful pamphlets.

I: Colourful pamphlets.

P: Or social media. Like uhm, things like uhm, (like)…an ad you see on maybe internet talking about the HIV. Yah.

I: Okay. So, colourful pamphlets and social media? Can we talk about maybe t-shirts, caps, food…what do you think about those?

P: Yah. T-shirts, yah. They can do it. She can supply them t-shirts, maybe uhm if uhm you see the other wearing the t-shirt and ask: “what is going on with the t-shirt?”, then she’ll explain more about it.

I: Okay.

P: Then one will be interested to…to try and get tested.

I: Besides t-shirts, what other incentives do you think can attract young people?

P: Uhm, I think maybe uhm, CD players.

I: CD players?

P: Yah.

I: Okay.

P: Things like that.

I: Any other?

P: Let me see. Ah! That’s all.

I: What about food?

P: Uhm, food? Ah! I don’t think food will do it.

I: Why don’t you think food will do it?

P: Because a person will take that food and eat then, no one will see that you gave them something. So, if you give them a t-shirt it’s going to last and it’s going to show some people.

I: Okay.

P: Yah.

I: So, when you’re talking about food, uhm, I’m not only referring to cooked food (right), the one you’re eating now. If I were to give uhm a hamper, yah, would they be interested in testing?

P: Uhm, they would be interested in the food, not the testing, you see? So, things like food, ah, I don’t think will work.

I: You don’t think food could be part of uhm…form of incentive?

P: Yah. But if they’re on the [inaudible segment], they must be given some food to eat you see?

I: So, cooked food?

P: Yah.

I: A plate of food?

P: Yah.

I: Not a hamper?

P: No.

I: Oh. If it was to be a hamper, what do you think youth would be interested getting in a hamper?

P: Uhm, maybe uhm…cosmetics.

I: Okay. Cosmetic.

P: Yah. Maybe a…sweet like chocolate, sweets.

I: Sweets. Are those the only incentive you can think about or you want to add more?

P: Uhm, no.

I: Okay. How often do you think this incentive should be given to people during HIV testing services?

P: Uhm, they must be given always after someone is tested.

I: Always? Okay. What could be the challenge of providing this incentive for HIV testing?

P: Can you repeat the question?

I: What could be the challenges of providing these incentives for HIV testing services?

P: Like challenges?

I: Mmhm.

P: Who are going to face those challenges? You or..?

I: What could be the challenges of giving out these incentives?

P: Uhm…maybe...maybe one person will come for more but has come before you see?

I: Mmhm.

P: Yah.

I: So, they will repeat themselves?

P: Yah. Try make money out of it. That is why I don’t agree with the food pack.

I: Okay.

P: Yah.

I: So, with the t-shirt you don’t think it can happen?

P: Yah. I don’t think it will happen. No one will buy a t-shirt which is written “HIV” you see?

I: Oh? So that t-shirt must be written something?

P: Yes.

I: What should it look like?

P: It should have colours but uhm…maybe big words written about uhm maybe HIV or whatever.

I: Okay. Okay. So, what do you think will be the benefits of providing these incentives for HIV testing services?

P: More people will come and get tested.

I: More people will come and get tested?

P: Yah.

I: Okay. Uhm, are those only the benefits that could be?

P: I think so.

I: Okay.

P: Because if more people are coming, which means more people are tested.

I: Okay. Please describe to me the thoughts about being contacted via telephone or social media for HIV testing services.

P: Uhm, to some people it can be offensive. To some can be painful because uhm, some they’re not aware or carrying the symptoms or what is going on about uhm HIV. Yah.

I: How do you think it would be offensive?

P: Uhm, maybe someone has the HIV disease. Maybe he thinks that maybe somebody is trying to insult her or…him or her.

I: Okay. So, how do you think uhm being informed via telephone, social media for HIV testing should be like?

P: Again?

I: How can we use telephone to inform without insulting or offending anyone…someone should be like?

P: I think a best way to use messages or WhatsApp. Don’t call people.

I: Ooh. So, we can use…

P: Social media.

I: Okay.

P: Or messages? (indeed)?

I: Okay. Can you describe some examples of how you have been informed about HIV testing services?

P: Uhm, I was given a pamphlet.

I: Okay. You were given only a pamphlet?

P: Yah.

I: Nothing else other than pamphlets?

P: No.

I: Okay. How could…how would you feel about being informed and registering for HIV testing services using your cell phone?

P: Uhm, it will be easy because uhm, I will save money. Yah.

I: So, it will be easy for you to…if you get registered for HIV testing via cell phone?

P: Yah.

I: It won’t be offensive or anyhow?

P: Yah.

I: So, it will be a good thing?

P: Sure.

I: Okay. How could cell phones be used to inform youth of HIV testing services?

P: To inform? How could they be used?

I: Mmhm.

P: Uhm, they can uhm, maybe uhm have some ad on the internet.

I: App?

P: Ad.

I: Okay.

P: Yah. If people are…maybe [inaudible segment] searching something then it appears on the top. Yah.

I: Okay. So, this ad should be accessible free or be with a use of data or some WiFI?

P: Uhm if you…it must be free, obvious.

I: Okay. Why do you think it must be free?

P: Uhm because uhm alerting people for their lives must be not? Wasting? Some money.

I: Mmhm. Okay. So, don’t you think if maybe we were offering data to young people they could uhm access these sites on the internet?

P: Yah. It could be that.

I: So, data can be part of the incentive?

P: Yah.

I: Do you think so?

P: I think so.

I: Okay. [pause] Please describe any challenges that youth might experience if they are contacted on their cell phones or for testing HIV…for HIV testing services.

P: Eish. Uhm, some youth uhm, have anger issues, you see?

I: Mmhm.

P: If maybe she or he already knows that uhm he’s infected, it could uhm make him or do something that will regret about you see?

I: Mmhm.

P: Maybe somebody calls you and tells you that you must test for your status then, but you already know about it and they’re not taking you well in your heart you see? Yah.

I: So it can be a challenge?

P: Yah.

I: Okay. Please describe the benefits of contacting youth on their cell phones for HIV testing. (Isn’t it) you just told me about the challenges?

P: Yah.

I: What could be the benefits?

P: They don’t have to move around for the information. It will easily come to them.

I: Oh?

P: Yah.

I: How easy?

P: Through phone calls (right?) or social media.

I: Okay. So, youth are usually on cell phones and social media?

P: Yes.

I: Okay. In your opinion, what types of other social media should be used to contact youth for HIV testing services?

P: Like WhatsApp, Facebook.

I: Okay. WhatsApp, Facebook.

P: Yah. (Like) Twitter (hey?).

I: Twitter?

P: Yah.

I: With the help of the data that they get as incentive right?

P: Yah.

I: Don’t you think they’ll use this, uhm, this data for some other things other than information?

P: No. if they were given to use it to…to access information, they will going to use it for it.

I: Okay. And what other challenges for using social media to contact youth for HIV testing services? What could be the challenges of using these social media platforms for HIV testing services?

P: Uhm some people uhm they will think that these are scams.

I: Ooohh.

P: Yah.

I: Okay. So, what could be the benefits of using social media to contact youth for HIV testing services?

P: It is easy for them to get the information without uhm, maybe traveling to the clinic and ask about it.

I: Okay. So, you said some people will think they are scams. So how do you think it should be put on social media so that people may not think that way?

P: You see it mustn’t involve uhm some…something like you’re going to get a reward like for maybe being asked some questions actually. You can reward someone if maybe is here already, but not through the internet or the cell phone.

I: Okay.

P: They will think that maybe it is some sort of a scam. Or maybe ask for their numbers or ID on the cell phones. You must ask them face-to-face.

I: Okay. So, on social media maybe how would you put the message? Maybe if you can phrase a message for me.

P: Maybe I can say uhm “let’s meet and talk about things like HIV which are causing people to…yah”.

I: Mmhm. That’s great. How do you think your parent or legal guardian will feel about you receiving information on testing uhm HIV testing services on your cell phone or social media?

P: Oh Uhm, he or she will be relieved because uhm there’s no parent who wants to talk with his uhm daughter or son about things like HIV. Especially if uhm, they’re infected. They’re afraid of talking about it.

I: So, parents don’t talk to their children?

P: Ah, not all of them. Some they do.

I: And in these instances, your parents, are you talking with your parents about HIV?

P: Ah, no, no.

I: You don’t?

P: [laugh] No I don’t.

I: Okay. Are they strict when it comes to talking about uhm HIV, sexual…

P: Yah. They’re strict.

I: Okay. So, can you tell me uhm…about any other suggestion that you may have which would encourage youth to test? Any suggestion that we haven’t mentioned maybe, but you have in mind you think it could work for young people to get tested?

P: I think social media.

I: Social media?

P: Yah.

I: Okay. Any other suggestions?

P: Eish! I don’t know what to say.

I: If you were maybe…you were to encourage, let’s say you, were to encourage youth of {XXX} (Name of place)section to come and get tested, what would you do? How will you attract them to come and get tested?

P: Uhm, I would simply play some music around.

I: Okay.

P: Yah. Go around telling people about what I’m hosting. Then if they’re coming then I’m going to explain more about it.

I: Okay.

P: Yah. Some youth uhm like music.

I: Okay.

P: Yah.

I: Is youth interested only in music or are there any other codes maybe that we can use to attract them besides music?

P: Maybe uhm…hiring a celebrity. Just one. You see? Yah. They will be interested to see what he or she is doing there, then will come.

I: Okay.

P: Yah.

I: So, the celebrity must come on what kind of event? The music event or…?

P: Any!

I: Any event?

P: Any.

I: I wanted to know those kinds of events that you can think of.

P: [laugh]

I: You just talked about entertainment. There are any entertainments maybe? Let’s try to explore “entertainment”.

P: Maybe [inaudible segment] something like an event but it is about uhm, alerting people.

I: Okay.

P: Or sports. Things like sports.

I: Oh sports events?

P: Yah.

I: Okay. Do you think youth are active on sports?

P: Yah. They’re very active in sports.

I: Okay.

P: Yah.

I: And the services must be at the sports ground?

P: Yah. Soccer fields, yah.

I: Okay. Are there any final thoughts you have about HIV testing incentives or about youth? Any final thoughts?

P: Uhm, I think, uhm people should be informed about the HIV. They must not stop giving them those things which are alerting them. They must carry on.

I: Those colourful pamphlets?

P: Yah.

I: What do you think of the banners? like when we’re having festivals, there are banners all over the streets. Do you think youth will read uhm HIV testing service message on a banner like they read with the festivals or what?

P: Yah. They can.

I: Okay. Now we’ve come to the end of our discussions. Thank you for your participation. If you have any questions like I explained to you on the informed consent form, there are numbers you can call anytime between 8am and 5pm.

P: Okay.

I: Yah. Thank you very much.

Time ended is 10:40.
