# Supplementary material for: Exploring perceptions of low risk behaviour and drivers to test for HIV among South African youth
Source: PLoS One. 2021 Jan 22;16(1):e0245542. doi: 10.1371/journal.pone.0245542 (PMC7822253; doi:10.1371/journal.pone.0245542)
Supplement: S1 File — (ZIP) [file pone.0245542.s001.zip › S1_File_Anonymised Transcripts/YA03-028-ML Transcription_QC2_TM.docx]

Full Participant ID: YA03-028-ML

Participant Type: 19 years old, male

Location:

Date: 12 September 2018

Start time: 12:22

Primary interview language: English

Name of Facilitator/Interviewer: Wellingthon Maruma

Name of Note Taker:

Name of Transcriber: Nokukhanya Ndinisa

Length of recording: 34:45

Label Key

I = Interviewer

P = Participant

N = Notetaker

{ } = Indicates that details were changed or pseudonyms were used to anonymise data

xxx = words were omitted to anonymise data

- = breaking into a sentence by the next speaker

… = pause or drawn out words

[ ] = indicates noise made, e.g. [laugh], [sigh], [pause]

[inaudible segment] = Unclear section of the recording

?Mulenga Clinic?, ?P3? = questionable text or doubt as to what was said or who said it

I: Thank you so much for being part of this interview. Do you allow me to record this interview?

P: Yes, I do allow you.

I: Okay, thank you so much. Can you tell me what your thoughts are on HIV? [pause] What you think HIV is? Do you know how its- uhm, you can get infected? Do you know what HIV is?

P: Uhm, I know HIV is a sickness which can be transmitted like via sex or if like there’s a blood transfusion between two people, with the one infected and the one not infected. That’s what I know HIV is.

I: Uh-huh, And- So... Have you ever been in a situation where you felt at risk of HIV, maybe of contracting HIV in your life?

P: Yes.

I: Can you tell me about it?

P: Recently, I had unprotected sex and I started having weird things on my body so that started being worried. So, that is the only like scenario where I felt like I was highly exposed.

I: Did you feel like this would be a point or something that will make you feel like you should go and get tested maybe?

P: Yeah, I felt that need but when I like- I did go to the clinic but they only told me it’s an allergy. So, I wasn’t tested, at all.

I: Did you ask them why you don’t wanna get tested?

P: No. Testing is actually very scary, so I think this is the main reason why I didn’t wanna test

I: Why is it so scary about getting tested?

P: You know what- The word HIV, to people, to us, it’s so scary. To us, we are- actually me, I’m scared to know, do I have it or not. That’s what I’m scared of.

I: Do you think there would ever be a time in your life where you overcome this fear and get tested?

P: Yeah, I think-

I: And then what do you think will make you get tested?

P: It’s actually my health, because I know if I don’t get tested I’m actually going to get very sick, as I’ve seen other people getting sick in front of me.

I: Uhm, Okay. You said that your health is important, right?

P: Yes.

I: But you still have not tested?

P: Yes.

I: Okay, so… I just want you to help me get through this… You said that your health is important, so-

P: Yes.

I: So, do you think that knowing your HIV status is important as well?

P: Yeah, it is important, I think it is.

I: Why?

P: So that if I am affected- infected, I can get medicine as soon as possible. Actually, the knowledge that I know is that it starts from HIV, then AIDS. So, to prevent it from going to AIDS, I have to be on medication.

I: Why do you think a lot of young people don’t access like HIV testing services? They don’t want to know their HIV statuses.

P: The word HIV is scary and that’s actually why we don’t wanna test.

I: And what do you would make you guys get tested?

P: Actually, it’s knowing that if you don’t test, what would happen. If you get more knowledge, maybe you could be more interested and worry about your health.

I: What kind of information could this be?

P: Like the impact of HIV, if you’re infected what will happen. If we know that if you don’t test, this is what will happen and where will it end. Then I think some choice may arise-

I: So, how- Let’s say that the lack of information is maybe the reason people don’t want to get tested-

P: Yeah.

I: Is that what you’re saying?

P: Yes.

I: Okay. So, say that we were to give information about HIV testing services-

P: Yes.

I: How would you want to receive that information?

P: Actually like, from a person I trust, maybe at home but it should be something like we have to be told about ARV’s, we have to be told that if you’re infected and ignorant about it, where will it end because people are scared of the word HIV, they’re not scare of it. So, of we actually know what is HIV, what will it do to your body if you don’t actually like treat it.

I: Okay. So, besides having someone that you trust, telling you this information about HIV. How else would you like to get information? I mean there’s different routes like pamphlets, TV. How else do you want to get HIV testing services information?

P: I think via social media because that’s what young people are mostly exposed to.

I: Social media, how? Maybe just explain.

P: WhatsApp groups of some sort.

I: And then what would happen there?

P: People would be taught about HIV and what it does to people and how you would have to treat it, given safety precautions and how to treat it, actually

I: Okay with social media- Is WhatsApp the only social media platform?

P: For me, WhatsApp is the most effective one-

I: Okay, and what about the others- how others- what others- I mean, do you know any others that could?

P: That could be used? [pause] Uhm, no. There aren’t any.

I: What about Facebook? You don’t think we could use Facebook for this type of information?

P: I don’t think so.

I: Why not? Why don’t you think Facebook is a good social media platform?

P: Facebook is crowded. Actually, when I say WhatsApp, it could be a private group. Actually, not a private group, it should be public so that people can get exposed to it.

I: By public, what do you mean?

P: Like the link should be everywhere for people when they want to get information, they should just join this group and be able to ask questions about HIV.

I: And on these groups, I know WhatsApps have like admins and people who actually own the group. So, who would you like to get information from in these groups? Would it be like amongst your friends? Would it be maybe-

P: No, actually-

I: That’s what I wanna hear.

P: It would actually be someone that has knowledge cause if I get from my friend, it’s only an opinion, it’s not a fact. What we’re looking for is facts.

I: Hmm, and this knowledgeable person would be like who? Give me an example, someone from the clinic?

P: Yeah, someone from the clinic. Someone who would give us appropriate answers to our questions.

I: Okay. Can you maybe just tell me about your understanding about the word incentives? The word incentive, word does it mean to you?

P: Incentive?

I: Hmm…

P: I’m not familiar with that word.

I: You’re not familiar with that word? [pause] So, say that you’ve heard of these {XXX} (Name of a non-profit organization) programs or these HIV testing platforms where they would maybe give a t-shirt for you to come and test?

P: Yes, like these Soul Buddies’ group.

I: Soul Buddies’ group, right? So, a t-shirt would be something like an incentive.

P: Yes.

I: So, what I would like to find out from you is what kind of incentives would encourage you to come and get tested for HIV seeing that you are not tested for HIV before?

P: A t-shirt cannot. Let’s say-

I: Why not? Why not a t-shirt?

P: Because I have my own. Actually, a t-shirt can never overcome the fear that I have. So, I think, yoh, a pair of sneakers. Something valuable like yeah.

I: Valuable like sneakers, what else? So, you’re saying if they were to tell you like if we give you sneakers if you get tested [cell phone vibration] Then you would get tested. Is it something that would make you want to get tested?

P: Yeah and if those sneakers were not going to be written HIV-HIV, you know people run away from the name HIV so yeah. If you would give me sneakers written HIV-what-what, then [laugh] I wouldn’t come.

I: Then what would they look like?

P: Just random sneakers.

I: What else? So, you’re saying t-shirts won’t work for you?

P: For me, t-shirts and caps won’t work.

I: So, sneakers and?

P: Voucher, maybe.

I: A voucher?

P: Yes.

I: Where would you use this voucher maybe? Or how much would this voucher be worth?

P: Maybe-

I: And then where would you use this voucher? Would it be something you can use everywhere or certain shops?

P: I could say everywhere.

I: And then what would you purchase with this voucher, personally with this R50 voucher?

P: Personally, I’d buy- I’ll probably just purchase anything that I need, which can be maybe airtime.

I: And then if airtime was given to you, as well? Let’s say we give you airtime, would that work?

P: That would be effective, very effective.

I: Why do you think so?

P: Actually, young people like social media. So, I see social media as a bait. So, airtime actually gives you access to the actual social media. So, yeah.

I: Okay. So, what about maybe food? Is it something that could encourage you to come and get tested?

P: No, for me, it’s a no.

I: Okay. Why not?

P: Ah, food… I have food at home so [laugh] I don’t think-

I: And then what about those who don’t have food at home and maybe they can’t even take their medication because they don’t have food? Do you think by providing food, it’s something that would encourage them to come and test?

P: Yes, it would. It’s a need for them, actually.

I: So, you’re saying that for you, a R50 voucher, airtime and sneakers would be-

P: Yes.

I: Would be something that would encourage you to get tested?

P: Yes.

I: And do you have anything in mind?

P: Regarding what?

I: Regarding these incentives?

P: No, I got nothing.

I: So, what do you think the challenge would be regarding providing these incentives? If we were to give you your sneakers, for example, right?

P: Yes.

I: What would be the challenge if we were to give you sneakers to come and get tested?

P: It would have to get the sneakers after you have tested, not before.

I: And what would the challenge with that be? Do you think people would come for the sneakers only, not because they wanna know their status?

P: Yeah, people would come for sneakers, but it would be helpful because people would already know about their statuses and once they know, they’d have to take action.

I: So, you’re saying that the benefit would be that people would already know their status?

P: Yes.

I: And they would be more knowledgeable on how to manage-

P: Yes, cause once you know, especially if you’re positive, you would want to know more to sustain your health.

I: Okay. Uhm, and the challenges would be that people would be coming for the sneakers?

P: Yeah, they would only be coming for the sneakers.

I: And what other challenges do you think of?

P: Uh, I don’t think there’s another challenge I can think of.

I: Okay, so… You spoke about the use of social media, right?

P: Yes.

I: Like WhatsApp groups and you were against the idea of using Facebook cause like its crowded, right?

P: Yes.

I: So, obviously to use social media you would need like tablet, phone or digital computer-

P: Yes.

I: How else do you think we can use those to relay information to the youth about HIV testing.

P: [Silence] It’s already there. The only thing I can think of is numbers to call, they’re already there.

I: Take me through it.

P: We already have numbers like if you wanna call, they’re already there.

I: I’m not following. Are you saying that we can use cell phones to call people?

P: Yeah but I don’t think that people can actually call. They will only call when they know that they are positive but if you don’t know if you’re positive, you’re not worried. So, it’s obvious, you won’t call, unless there’s something that you that alerts you that should call.

I: So, let’s go back to the incentives that you mentioned, right? The sneakers, the voucher and the airtime, right?

P: Yes.

I: How often do you think these things should be given out? Is it once or everything you get tested? How often do you think they should be given?

P: I think once because it would be a challenge. People would be coming every day, today, tomorrow like so that they could get more and more.

I: But don’t you think that the challenge would be that someone else might not want to come again because they are only given once. Two from now they would not want to come and get tested because they-

P: Because they already have? Yeah. Okay, my view is that the person should like get- Uhm… how long is this period? They say its six days or six weeks?

I: Three months.

P: Maybe they should be given within four months. Let’s say you are given today and then again after four months. Not today, tomorrow, daily. If you come again within the four months, you should not be given but if you come after these four months you should be given.

I: So, you’re saying once, every three months?

P: Hmm…

I: Okay. So, you’ve mentioned, uhm… ways that you can use, right? Like cell phones, social media and you said we could call the patients? I didn’t understand that. My question was else can we use cell phones to get the information out there? Or would you wanna be contacted about HIV testing services?

P: No, I wouldn’t.

I: Why not?

P: No, it would just be weird. I wouldn’t like talking about it. Like people that are unaware, especially if you don’t know who’s listening when you’re talking over the phone, but if you’re typing you already know that it’s confidential, you know that it’s your own information.

I: But it’s not about calling, what about someone texting you?

P: That would work.

I: Tell me why that would work for you personally?

P: Oh, you’re talking about me? It won’t be verbal. If it’s a text, I can keep it a secret but if it’s verbal, I don’t know who’s listening.

I: Why do you feel the need to keep it a secret?

P: Your status is a secret these days, so automatically it would have to be a secret.

I: So, you’re saying that even if your mother would know that you’re receiving some HIV information on your phone, would you wanna keep it a secret from them as well?

P: No, I wouldn’t.

I: How do you think they would feel if you were to receive any HIV testing information on your phone? It doesn’t have to be your status, but maybe information about HIV testing services is important. How do you think they would feel?

P: Uh, they would feel… I don’t how they would feel but it wouldn’t be nice.

I: You don’t think they’d allow that?

P: No, they wouldn’t.

I: Why not?

P: It would be better if it’s something that you subscribe into.

I: Tell me more.

P: If you subscribe into it, not that you just get a message randomly. If you subscribe, if there is a WhatsApp group, you’ll have to join. Yeah, that’s- You know that if I join, I’ll be getting these messages. So, not just like, random messages, random messages.

I: Yeah, so my question is- so let’s say your parents know that you’re on these WhatsApp groups and you’re receiving this information on HIV testing or anything related to HIV. How do you think they would take it?

P: [Silence]

I: So, it’s not like an SMS coming through or a WhatsApp group. Let’s say I’m your mother and I know that you’re receiving information regarding HIV. How do you think that would make you feel?

P: It depends on the mother.

I: Tell me about your parents.

P: My parents… Okay, my parents would think that I’m already HIV positive, cause like, even now, I don’t tell my parents that I’m sexually active. For me, that’s shameful, actually. So now, going from that and jumping to information about HIV would be a big step.

I: And what do you think a lot of parents are not really receptive about their kids receiving information about HIV? You spoke about fear, is it?

P: Yes, I did.

I: You’re scared. Do you think their parents are scared as well?

P: Yes.

I: Why do you think they don’t want you to associate with anything HIV-related?

P: I could say that they’re also scared because there couldn’t be any other reason besides being scared.

I: But why do you think they’re scared?

P: They’re scared that you could actually be positive. There is this notion that what you don’t know, won’t kill you. We, as people hide against that and which is actually not the case.

I: So, uhm… Let’s go back a bit. Which clinic did you attend where they told you that you might have an allergy?

P: ?P3? Clinic but it’s in KZN.

I: How was your experience when you went there?

P: They asked me that do I wanna- When I entered, one of the nurses was looking at me and he said it was a rash and they he asked me if I was HIV positive or not, I said I don’t know. He asked me if I will like to get tested, then I said no, I only came so that they can remove what was on my neck.

I: So, she asked if you wanted an HIV test and you refused?

P: Yes.

I: Okay, is there anything that would have made you to say yes to getting tested? Maybe if she had offered you something there.

P: Because I was scared-

I: Even if they provided those sneakers that you spoke about?

P: The sneakers would only be effective if I’m sitting at home and thinking about what if I get tested and if I get tested, I’ll get the sneakers. That’s when the sneakers would be effective but now because I’m scared-

I: But do you think you’ll ever overcome this fear?

P: Yes, I will.

I: When would that be?

P: Like I said, having more knowledge. Like right now, like for me, I already have that knowledge that now I should get tested.

I: Okay, so you do think that it is important to know your status?

P: You only get concerned if you know the results of getting tested. If there is this notion that what you don’t know won’t kill you, that’s why we are behind.

I: Okay. So, do you think there is any positive thing about the current HIV testing services in South Africa? Like the clinic that you went to, do you think there is any positive thing about the services that they provide with regards to HIV testing services?

P: Yes, I do.

I: Take me through the. The good things?

P: They like… like- I could say that the age that they- they don’t have any age where they say you don’t get tested. Everyone is allowed to get tested, only if you give your consent.

I: Im listening. What about the negatives? Do you have any negative experiences or that you’ve heard of?

P: The negative like I’ve experienced negatives. I was entering, and they were saying like- I don’t know if they were being sarcastic or not but they had these assumptions that since I have rash, I’m already HIV positive

I: So, you’re saying that the clinic staff sometimes assumes people and that would deter people from testing as well because there is already an assumption that people are-

P: Yes. You would be scared because uhm, if I’ve got something weird, I’m HIV positive, without testing me.

I: Okay. So, all the things that you’ve mentioned, like the people making assumptions and-

P: Yes.

I: So, I wanna hear from you, the suggestions. What suggestions do you have that you can come up with?

P: With accordance to what?

I: All the negative things that you’ve spoke about. So, you’ve mentioned like the staff are giving- like assuming people are HIV positive and staff. Uh, some parents will not allow their kids to receive HIV testing information. So those were some the negative things?

P: Yes

I: So, I want suggestions of how to overcome the negative things.

P: Maybe if the parents also get educated so that they can know as much as the youth can be taught on the same wavelength and know… With accordance to the staff, I can’t think of any.

I: So, you said, providing information. That was one of the suggestions, right?

P: Yes.

I: So, you spoke about information, you spoke about social media, you spoke about incentives like sneakers, airtime and food… and yeah, right?

P: Yes.

I: You are suggesting that we just give people information about HIV?

P: Yes.

I: So, is that enough? Do you think that would solve everything? Just giving people information? Cause I mean that has been done. So, I wanna hear from you, what other creative ways can make sure that people get the information they need about HIV testing services or improve the HIV testing services in our health centres?

P: It’s very hard to get consent from a person. Actually, you can’t force a person to get tested. Those are the only things that I can think of instead- cause there was this clinic where they told me that they can’t help me if I don’t get tested because they don’t know my health condition.

I: Say that again.

P: They said that I have to get tested first so that they can know my health condition, so that they can know what they are dealing with.

I: So, you’re saying that because clinics would want to know your status, it is not something that you would want because they’re helping you? I’m not following.

P: [laugh] I’m actually saying that it be effective if they tell you that before they help you, you would have no choice but to-

I: Okay, I get you. So, you’re saying-

P: But you wouldn’t be verbally forcing the person. The person would actually force themselves because at the end of the day, it is important to-

I: Let me see if I’m getting this straight. So, you’re saying if I’m coming to the clinic and I’m sick with ABC, right?

P: Yeah.

I: The clinics should force me to get an HIV test?

P: No, not force you but they would tell you to raise an alert that this is actually serious. At the end of the day, they have to get your consent, using thing this of saying before we help you, we have to know everything about you. That could be effective too.

I: Hmm… I hear you. So, you’re saying that the only suggestion that you have is giving information to parents by teaching them about HIV? And giving sneakers and social media, is that the only suggestions you can think of?

P: Here’s an idea. Like there could be a room-to-room, people getting from houses to houses, teaching people. That could also be effective, because when we use social media, not everyone is on social media. Like those who don’t have cell phones, it means that won’t reach the information. So, if we go house-to-house, everyone will be exposed to the information.

I: Okay. So, we’re almost at the end of our interview. Do you have any final thoughts about youth, HIV testing services or the incentives that you mentioned? Maybe you wanna add in the incentives that you just thought of now, over and about the sneakers, the vouchers, the airtime?

P: No, there isn’t.

I: And your final thoughts about HIV testing?

P: [silence]

I: What are your final thoughts about HIV testing services?

P: I don’t get you.

I: Is it important to test for HIV?

P: Yes.

I: What your thoughts on HIV testing? What are your final thoughts?

P: Okay, my thoughts about HIV testing?

I: Hmm…

P: It is important for everyone to test because it’s our health, so we have to know. As I said, if we actually have the knowledge, we would be actually encouraged to know about our healths, not the other person or anybody else. Even the statistics are saying that being HIV- actually, I can’t say HIV because HIV is really like taking people. Whether you know about your status or you don’t know. Still, it’s going to affect you.

I: And do you think this lack of knowledge is only among the youth or? Cause you’ve emphasized this lack of knowledge many times. Is it only amongst the youth or even the older people?

P: Even the older people. Yes, like I said, some parents don’t like talking about these things not because they don’t like them but because they have no knowledge to pass into their kids. If all of us could be exposed to this knowledge

I: And the you said that this knowledge can be passed through house-to-house, WhatsApp groups, right?

P: Yes.

I: Do you have other ways in which the information can be passed through? What other ways? Cause you did say that you don’t like Facebook. How else can we- do you have another way?

P: I can’t say vouchers and those leaflets because I’ve seen them being torn. Even I, if you give it to me, I have no interest but if it’s in my cell phone, I’ve got interest and only if they give it like an interesting topic, like an eye-catching topic.

I: Give me an idea of these topics.

P: They shouldn’t start with like blissful things. The topic should be like head-on, like the effects of not knowing your status. Actually, everyone already has fear, so when you like, this topic will actually attract you more, since like I don’t know my status. Then I would get interested to find knowledge but these leaflets, aah- I’ve seen them been torn.

I: And you saying through phone, would only be on WhatsApp?

P: Yes, all I can think of now is the digital platform. Otherwise these leaflets, papers, posters, they’ve been used but even I don’t pay attention to them.

I: So, we’ve come to the end of our discussion and I just wanna thank you for your participation. Uhm, if you have any questions about our study, you are more than welcome to contact me. I’ve given you my number. Thank you so much for your participation.

P: Thanks.

End time: 12:57
