# Supplementary material for: Exploring perceptions of low risk behaviour and drivers to test for HIV among South African youth
Source: PLoS One. 2021 Jan 22;16(1):e0245542. doi: 10.1371/journal.pone.0245542 (PMC7822253; doi:10.1371/journal.pone.0245542)
Supplement: S2 File — (DOCX) [file pone.0245542.s002.docx]

**COREQ (COnsolidated criteria for REporting Qualitative research) Checklist**

A checklist of items that should be included in reports of qualitative research. You must report the page number in your manuscript where you consider each of the items listed in this checklist. If you have not included this information, either revise your manuscript accordingly before submitting or note N/A.

| **Topic** | **Item No.** | **Guide Questions/Description** | **Reported on Page No.** |
| --- | --- | --- | --- |
| **Domain 1: Research team and reflexivity** |  |  |  |
| *Personal characteristics* |  |  |  |
| Interviewer/facilitator | 1 | Which author/s conducted the interview or focus group?  Chetty-Makkan and Maruma | 24 |
| Credentials | 2 | What were the researcher’s credentials? Authors had  PHD, MD, MSC and MPH degrees | 11 |
| Occupation | 3 | What was their occupation at the time of the study?  Authors were part of the research management and data collection team | 24 |
| Gender | 4 | Was the researcher male or female?  Researchers were both male and female | 11 |
| Experience and training | 5 | What experience or training did the researcher have?  All authors had experience in conducting studies that involve qualitative data collection and analysis | 24 |
| *Relationship with participants* |  |  |  |
| Relationship established | 6 | Was a relationship established prior to study commencement? There was no pre-existing relationship with participants prior to study commencements, participants were only first contacted via telephone to set up appointments for interviews | 6 |
| Participant knowledge of the interviewer | 7 | What did the participants know about the researcher? e.g. goals, reasons for doing the research  Participants understood the goals and purpose of the study as discussed during the informed consent process. | 6 |
| Interviewer characteristics | 8 | What characteristics were reported about the interviewer/facilitator? e.g. Bias, assumptions, reasons and interests in the research topic  The interviewers were experienced and trained in data collection. They did not disclose any personal preferences and maintained an objective perspective during the interview process. | 7 |
| **Domain 2: Study design** |  |  |  |
| *Theoretical framework* |  |  |  |
| Methodological orientation and Theory | 9 | What methodological orientation was stated to underpin the study? e.g.  grounded theory, discourse analysis, ethnography, phenomenology, content analysis  Thematic analysis using deductive and inductive approaches | 10 |
| *Participant selection* |  |  |  |
| Sampling | 10 | How were participants selected? e.g. purposive, convenience, consecutive, snowball  We used a word-of-mouth technique were people that took part in the study referred their friends | 6 |
| Method of approach | 11 | How were participants approached? e.g. face-to-face, telephone, mail, email  Participants were approached via telephone and face-face | 6 |
| Sample size | 12 | How many participants were in the study?  IDI: n=25; FGD: n=4 | 7 |
| Non-participation | 13 | How many people refused to participate or dropped out? Reasons? No one refused participation. Three participants (10.7%) for the IDI were excluded due to reporting they tested for HIV. We had limited screening data on those who were recruited for the FGDs | 7, 22 |
| *Setting* |  |  |  |
| Setting of data collection | 14 | Where was the data collected? e.g. home, clinic, workplace  Study data were collected at clinic settings | 5-6 |
| Presence of nonparticipants | 15 | Was anyone else present besides the participants and researchers?  For FGDs, a note taker was present with researcher and participants during sessions. For the IDI, it was only the researcher and a participant | 7 |
| Description of sample | 16 | What are the important characteristics of the sample? e.g. demographic data, date  In total there were 25 participants for the IDIs; where 14 were females and 11 males. For the 15 to 17 years old category (females = 6; males = 5) and the 18 to 24 years old category (females = 8; males = 6) were enrolled. A total of 12 participants indicated that they were currently in a relationship; 13 were sexually active; 23 reported they had high school education and 2 had tertiary education and 16 reported that they had cellphones ( refer to Table 1). We had four FGDs; where participants were divided according to two age categories (15 to 17 years and 18 to 24 years) which were also gender specific. In total 18 participants participated in the FGDs. | 7 |
| *Data collection* |  |  | v |
| Interview guide | 17 | Were questions, prompts, guides provided by the authors? Was it pilot tested?  Interview guides were provided. Probes were reviewed and adapted during the course of the study. During debriefing sessions, research staff made suggestions on additional probes to include for example type of incentives, and frequency of receiving the incentive. | 7 |
| Repeat interviews | 18 | Were repeat interviews carried out? If yes, how many?  No, there were no repeat interviews | 6 |
| Audio/visual recording | 19 | Did the research use audio or visual recording to collect the data?  Audio recording was used | 6 |
| Field notes | 20 | Were field notes made during and/or after the interview or focus group?  Yes, field notes were made during focus group and IDI sessions | 10 |
| Duration | 21 | What was the duration of the interviews or focus group?  IDI lasted approximately 45 minutes to an hour per interview and FGD took approximately 1-2 hours per session. | 7 |
| Data saturation | 22 | Was data saturation discussed? Yes | 6 |
| Transcripts returned | 23 | Were transcripts returned to participants for comment and/or correction?  No, transcripts were not returned to participants for comment and/or correction. | 6 |
| **Topic** | **Item No.** | **Guide Questions/Description** | **Reported on Page No.** |
| **Domain 3: analysis and findings** |  |  |  |
| *Data analysis* |  |  |  |
| Number of data coders | 24 | How many data coders coded the data?  Three coders (CMC, WM and TM) | 11 |
| Description of the coding tree | 25 | Did authors provide a description of the coding tree?  Yes, an iterative approach was used to develop the codebook and index the transcripts from which categories, themes and patterns developed from the data. The codebook was discussed at a round table meeting where new codes were suggested. The authors agreed to particular codes. Codes that lacked intercoder reliability were dropped from the analysis. The codebook was revised by authors and the themes finalised which are displayed as direct quotes | 11 |
| Derivation of themes | 26 | Were themes identified in advance or derived from the data?  Themes were derived from data (inductive), some categories were identified (refer to IDI and FGD guide) | 11 |
| Software | 27 | What software, if applicable, was used to manage the data?  QSR Nvivo 10 | 10 |
| Participant checking | 28 | Did participants provide feedback on the findings?  No, participants did not provide feedback | 11 |
| *Reporting* |  |  |  |
| Quotations presented | 29 | Were participant quotations presented to illustrate the themes/findings?  Was each quotation identified? e.g. participant number  Yes, participants quotations were presented to illustrate findings with identified quotes | 13-19 |
| Data and findings consistent | 30 | Was there consistency between the data presented and the findings?  Yes | 13-19 |
| Clarity of major themes | 31 | Were major themes clearly presented in the findings?  Yes | 13-19 |
| Clarity of minor themes | 32 | Is there a description of diverse cases or discussion of minor themes? Yes, there were attributes identified to illustrate the findings. | 13, 14 and 16 |

Developed from: Tong A, Sainsbury P, Craig J. Consolidated criteria for reporting qualitative research (COREQ): a 32-item checklist for interviews and focus groups. *International Journal for Quality in Health Care*. 2007. Volume 19, Number 6: pp. 349 – 357

**Once you have completed this checklist, please save a copy and upload it as part of your submission. DO NOT** **include this checklist as part of the main manuscript document. It must be uploaded as a separate file.**
